# Regioselective Syntheses of Bis(indazolyl)methane Isomers: Controlling Kinetics and Thermodynamics via Tunable Non-Innocent Amines

**DOI:** 10.1021/acsomega.5c08094

**Published:** 2025-11-14

**Authors:** María Álvarez-Sánchez, Margarita Gómez, Carina Santos Hurtado, Jean Ngouné, Eleuterio Álvarez, Agustín Galindo, Claudio Pettinari

**Affiliations:** † Instituto de Investigaciones Químicas (IIQ), Spanish National Research Council (CSIC), University of Sevilla (US), Avda. Américo Vespucio 49, Isla de la Cartuja, 41092 Sevilla, Spain; ‡ Chemistry Interdisciplinary Project (ChIP), School of Pharmacy, University of Camerino, Via Madonna delle Carceri, 62032 Camerino MC, Italy; § Departamento de Química Inorgánica, University of Sevilla (US), Aptdo. 1203, 41071 Sevilla, Spain

## Abstract

The selective synthesis of regioisomers from ambident N-heterocycles remains a challenge in organic chemistry. We report a general and modular method for the regioselective syntheses of bis­(indazolyl)­methane isomers, in which the outcome is controlled by the nature of the base. Specifically, we employed structurally diverse amines as noninnocent bases, whose steric and electronic properties—particularly their p*K*_a_H and ability to act as methylene carriers or activators—play a decisive role in directing product distribution. By fine-tuning the amine structure, we achieved selective access to symmetrical and unsymmetrical isomers under mild, one-step conditions, without intermediate isolation. The use of amines over conventional inorganic bases was essential to enable both chemo- and regioselective control, while minimizing overactivation or competing pathways. Experimental findings were supported by DFT calculations that rationalize the observed selectivity through differential activation energies and intermediate stabilities. The methodology accommodates both classical methylenating agents (e.g., CH_2_Br_2_) and in situ generated ammonium-based donors. All compounds were fully characterized, and key products were confirmed by single-crystal X-ray diffraction (SCXRD). This strategy highlights the utility of noninnocent amines as tunable reagents for regioselective transformations of ambident nucleophiles, with broad potential applications in ligand design, supramolecular chemistry, and heterocyclic synthesis.

## Graphical abstract

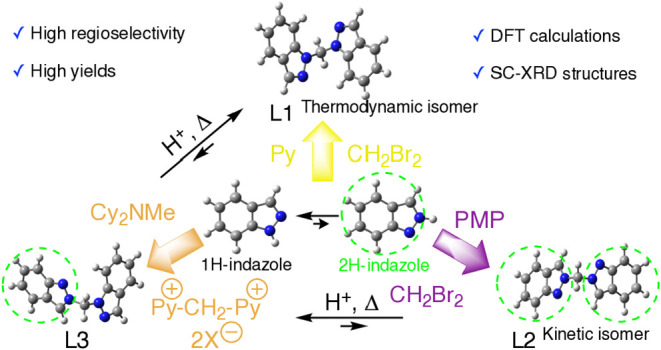

## Introduction

Bis­(indazolyl)­methane ligands (BINDMs) have recently emerged as promising scaffolds in coordination chemistry, owing to their modular N,N-donor architecture and ability to generate well-defined metal complexes for catalysis and biomedical applications. Their structural similarity to bis­(pyrazolyl)­methane and the presence of nonequivalent nitrogen atoms make them especially appealing for tunable ligand design. However, the selective synthesis of specific BINDM regioisomers remains a significant synthetic challenge due to competing alkylation pathways.

Bis­(pyrazolyl)­alkanes [R_2_C­(pzx)_2_], first reported by Trofimenko^1^ have long been studied as ligands in catalysis and biomimetic systems.^2–5^ In particular, the isostructural BINDMs have shown promise in Rh- and Cu-mediated C–H functionalization, and metal–organic frameworks.^6,7^ Recent reports also link these scaffolds to antibacterial and anticancer applications.^8–10^

Several years ago, we turned our attention to BINDMs, recognizing their potential in biologically relevant metal coordination and catalysis.^11–14^ We previously reported that the regioisomers di­(1H-indazol-1-yl)­methane and di­(2H-indazol-2-yl)­methane exhibit distinct coordination modes toward Zn­(II), Cd­(II), and Hg­(II),^15^ as well as Group 11 and 9–10 metals.^16–18^

Despite their relevance, the synthesis of pure regioisomeric BINDMs is often hindered by low selectivity during N-alkylation of indazole, especially in the presence of strong bases.^19,20^ Both N1- and N2-alkylation pathways are accessible, and mixtures of regioisomers are typically obtained, as first described by Juliá et al. in 1982 under phase-transfer conditions Scheme 1.^21^ These mixtures consist of three possible isomers: di­(1H-indazol-1-yl)­methane (**L1**), di­(2H-indazol-2-yl)­methane (**L2**), and the nonsymmetrical (1H-indazol-1-yl)­(2H-indazol-2-yl)­methane (**L3**). Their structural similarity makes their chromatographic separation particularly challenging, and the unselective nature of the classical methodology limits access to pure BINDMs for targeted applications.

**Scheme 1:**
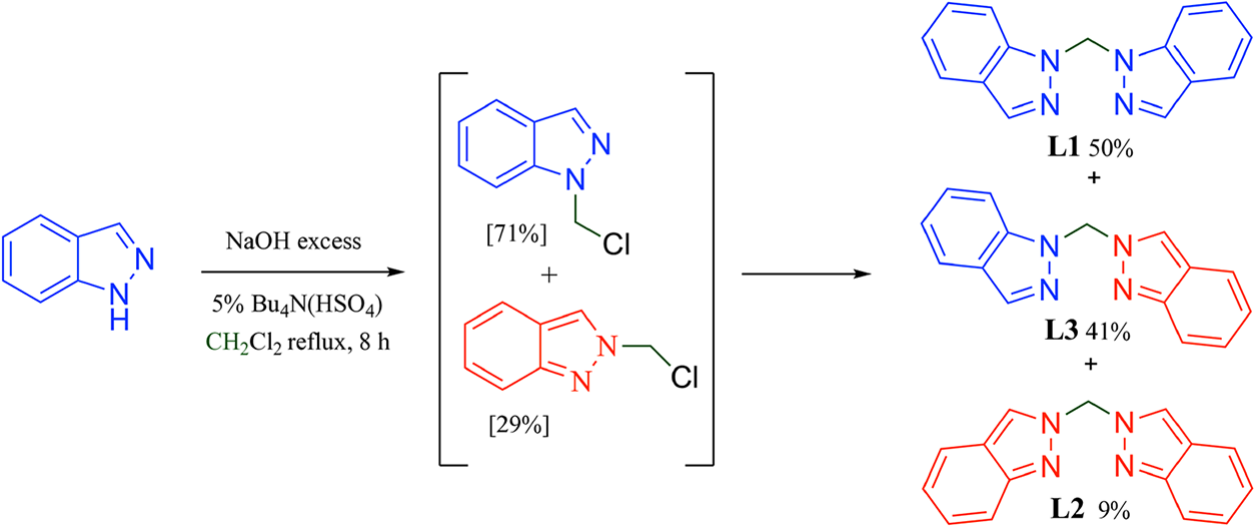
Phase-Transfer Strong Base Mediated One-step Synthesis of Bis­(indazole)­methane Isomers **L1**, **L2**, and **L3** Reported in 1982

Unlike imidazole and pyrazole, indazole has two nonequivalent nitrogen atoms, leading to regioselectivity that is highly sensitive to reaction conditions. Parameters such as base strength, solvent, and temperature play a decisive role in isomer distribution.^22,23^

Although several strategies have been developed to selectively obtain di­(1H-indazol-1-yl)­methane (**L1**), including acid-mediated rearrangements and 3d-metal salt-catalyzed one-pot protocols (Scheme 2, parts (a) and (b)),^24–27^ these methods are typically limited to this isomer exclusively and offer poor control over isomer distribution. As shown in Scheme 2, parts (a) to (c), the thermodynamic isomer **L1** ^28^ is often favored under elevated temperatures or acidic conditions.

**Scheme 2:**
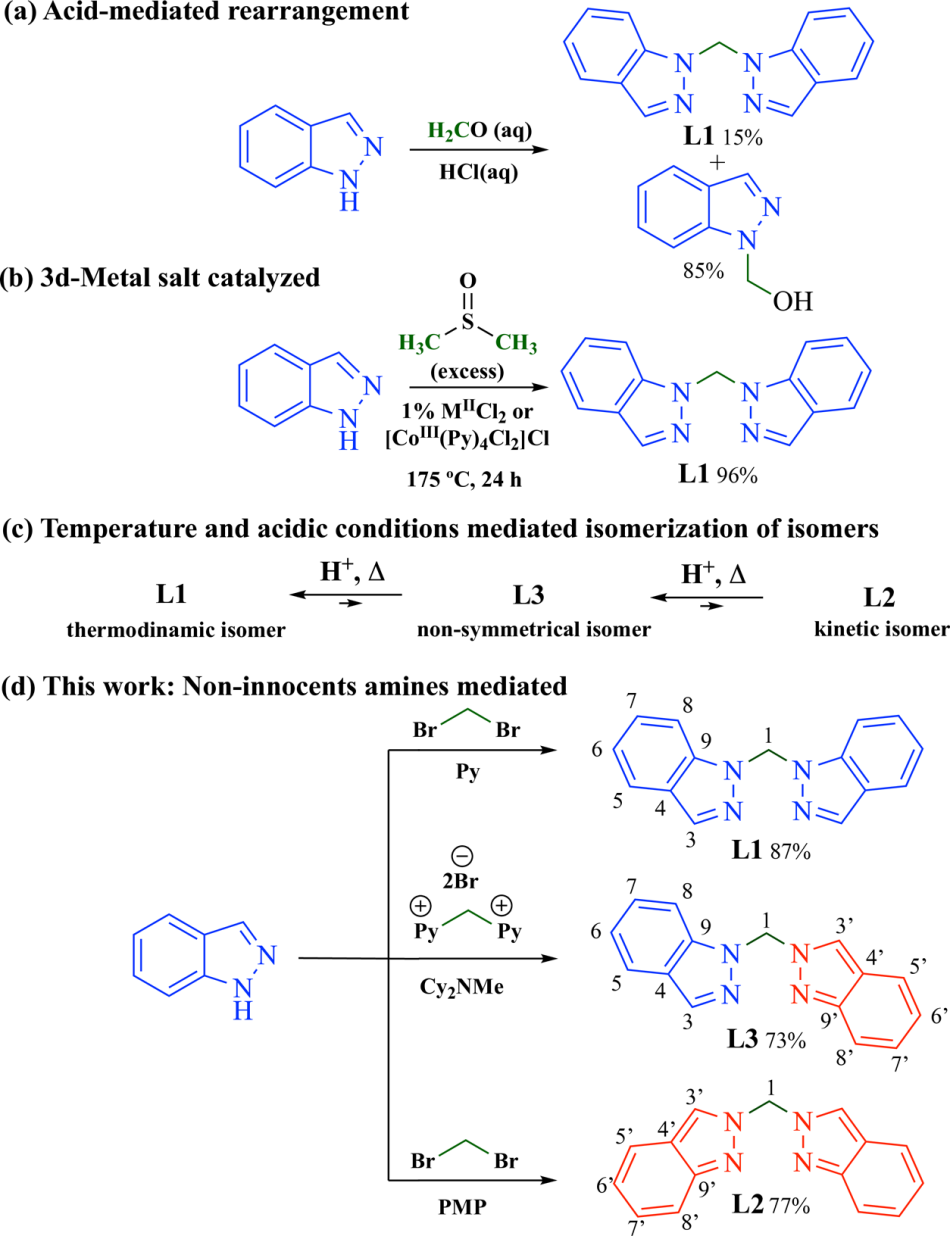
(a, b) Previously Reported Pathways for the Selective Formation of **L1**. (c) Schematic Representation of the Relative Isomerization Tendencies of BINDM Isomers **L1**–**L3**, Showing **L1** as the Thermodynamic Product and **L2**/**L3** as Kinetically Accessible Intermediates. (d) Present Strategy Developed in This Work, Based on Tunable Non-innocent Amines

In this work (Scheme 2, part (d)), we introduce a rational strategy for the regioselective synthesis of BINDM isomers by carefully selecting noninnocent amines. By tuning base structure and p*K*_a_H, the outcome can be directed toward a desired isomer, as supported by experimental evidence and DFT calculations. This concise overview contrasts our approach with earlier reports (paths 1–3) and clearly positions the present work as a complementary, generalizable methodology.

## Results and Discussion

### Preliminary Synthesis and Isolation of Bis­(indazolyl)­methane Isomers

As previously mentioned, we have used the two symmetrical regioisomers of bis­(indazolyl)­methane (**L1** and **L2**) as N,N-bidentate ligands in coordination complexes with Group 9 to 12 metal salts.^15,16,18^ These ligands were synthesized from indazole and CH_2_Br_2_ in DMF using NaH as a strong ionizing base at room temperature. The isomeric ratio of **L1**, **L2**, and **L3** determined by ^1^H NMR was consistent with previous results obtained using CH_2_Cl_2_ and NaOH under phase-transfer conditions.^21^ This consistency supports the formation of delocalized indazolide anions in solution, which is key in explaining the nonselective outcome under these basic conditions.

The isolation and purification of these isomers by chromatography proved challenging due to their close polarity and structural similarity. Nonetheless, a low-pressure column chromatography protocol was optimized to efficiently separate **L1** (first eluted) and **L2** (last eluted). The nonsymmetrical isomer **L3** eluted between them but remained contaminated with unreacted indazole.

To purify **L3**, the mixture was acetylated with Ac_2_O/Et_3_N in CH_2_Cl_2_, catalyzed by 5% DMPA, affording a 3:2 mixture of indazole acetate regioisomers: **1-acetyl-1H-indazole** (an oil previously described) and **2-acetyl-2H-indazole** (a novel crystalline solid that readily isomerizes in solution to **1-acetyl-1H-indazole**).^29–31^ Their structures were confirmed by NMR and X-ray crystallography. These acetylated derivatives allowed indirect purification of **L3** by chromatography, which eluted last under the same conditions.

Further details of the purification, including Scheme S2, Figure S2, and Table S1, are provided in the Supporting Information. Although not strictly required, the regioselective acetylation (Table S1) was also included as complementary information, since it revealed a previously undescribed indazole derivative and allowed us to compare the regioselectivity of acetylation under different conditions (Table S1), which may be of interest to readers.

Considering the relative Gibbs free energy differences (Δ*G*) between the three bis­(indazolyl)­methane isomers (see DFT study below) and prior reports on indazole alkylation reactions in the absence of strong bases,^22,23,32^ we hypothesized that judicious selection of the amine base— taking into account its p*K*_a_H, nucleophilicity, and steric profile—could impact the regioisomers distribution. In particular, we envisioned that certain amines may act as noninnocent species, not only deprotonating indazole but also transiently stabilizing specific transition states or intermediates via hydrogen bonding or ion-pairing effects. By pairing the methylene source with structurally distinct bases, we aimed to modulate the outcome of kinetic vs thermodynamic control, enabling regioselective access to a desired isomer and improving its isolated yield.

### Regioselective Syntheses of Bis­(indazolyl)­methane Isomers

Among the methylenating agents screened, dibromomethane (CH_2_Br_2_) was chosen as the preferred methylene donor due to its moderate volatility and clean reactivity. Dichloromethane was excluded because of its low boiling point, while diiodomethane showed decomposition tendencies under the applied conditions.

In addition, methylenating ammonium salts derived from pyridine, 3-methylpyridine, and DABCO (Figure 1) were synthesized and fully characterized (see Supporting Information). Their use was motivated by two considerations: first, to modulate steric effects in the transition states by comparing aromatic versus saturated systems (e.g., pyridine vs 3-methylpyridine and sp^2^ vs sp^3^ donors such as DABCO); and second, to introduce a secondary amine component of different basicity that could compete with the primary base. This competition was expected to generate trace acidity during the reaction, thereby favoring the selective formation of the nonsymmetric isomer **L3**, which occupies an intermediate position in stability among the three bis­(indazolyl)­methane isomers.

**Figure 1:**
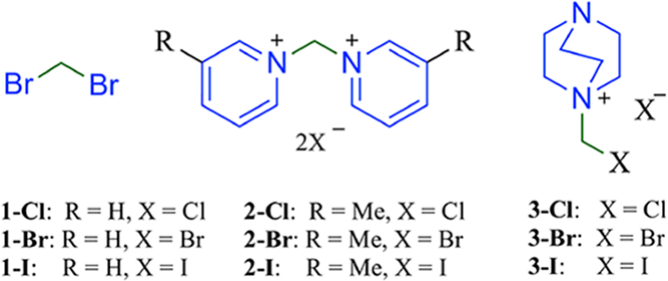
Methylenating agents employed in this study.

Figure 2 shows the p*K*_a_H values of their conjugate acids—ammonium ions for aliphatic amines, pyridinium ions for pyridines, and quaternary ammonium salts for DABCO derivatives. This parameter provides an indirect but reliable measure of their basic strength, deprotonation capacity, and potential role as noninnocent bases. Unlike p*K*_a_, which refers to the acidity of the free base, p*K*_a_H reflects the Brønsted basicity relevant under our reaction conditions.

**Figure 2:**
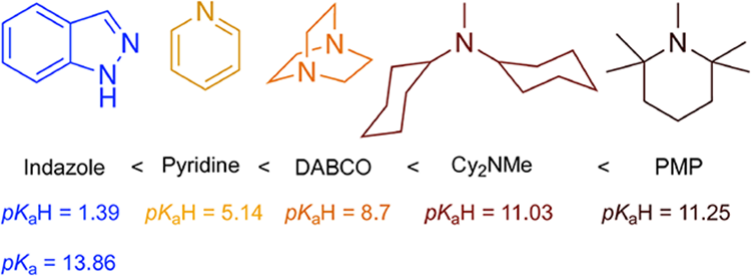
Relative basicity of the amines used, expressed as p*K*_a_H values of their conjugate acids (ammonium, pyridinium, or quaternary ammonium ions). The p*K*_a_ of indazole is included for comparison.

Unlike classical ionizing bases such as NaH or NaOH, which generate strongly basic anions in solution, the amines employed in this study act as neutral proton acceptors. These bases were selected not only for their effectiveness as strong proton scavengers, but also for their steric profiles, which can modulate transition states and influence selectivity toward the kinetic product **L2**. Their dual role as proton acceptors and sterically demanding agents underpins their designation as noninnocent bases.

To identify optimal conditions for regioselective isomer formation, each reaction was monitored by ^1^H NMR to quantify **L1**, **L2**, **L3**, and unreacted indazole. Most entries in Table 1 reflect analytical-scale screenings, with relative isomer distributions determined by signal integration (for more details see Supporting Information, Figure S1 and data). Only selected entries were scaled up to isolate individual isomers, and in those cases, isolated yields are provided in parentheses in Table 1 (explained in the footnotes).

**Table 1:**
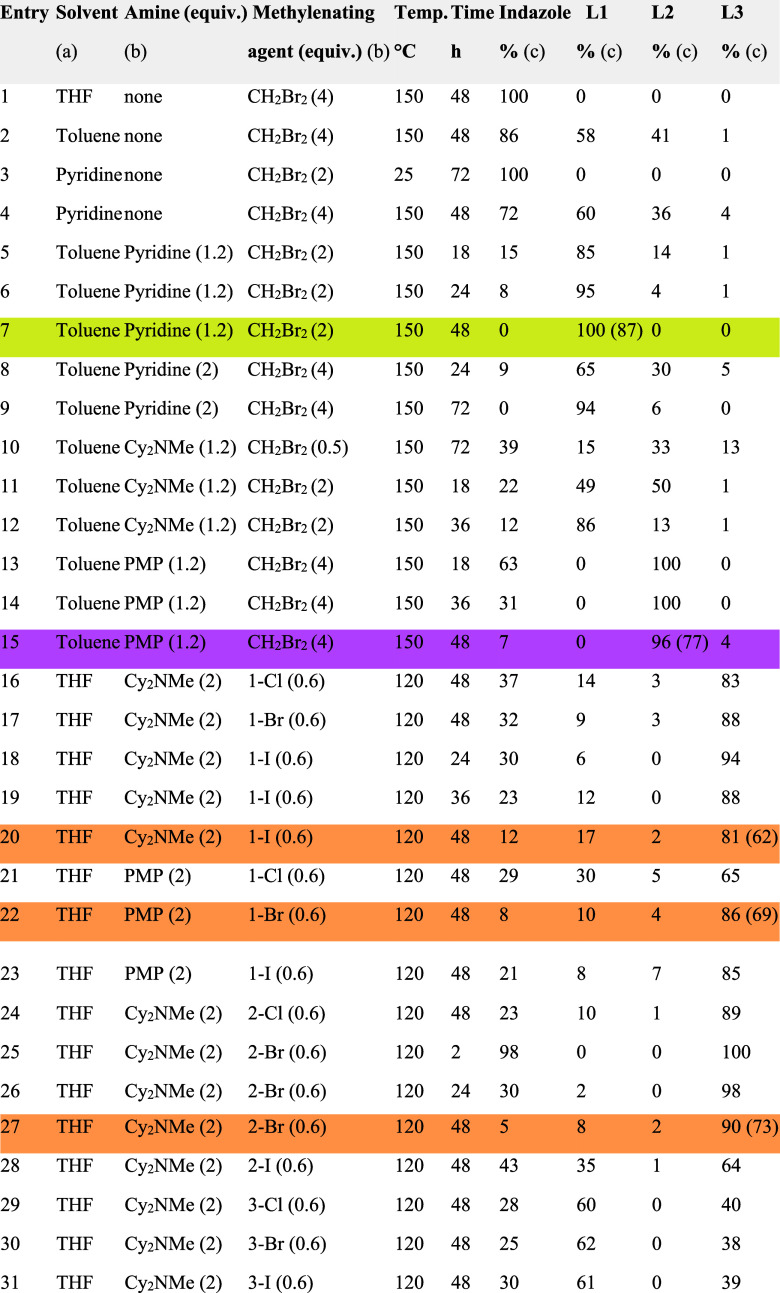
Summary of Key Experiments Conducted to Optimize Reaction Conditions for the Regioselective Synthesis of a Specific Bis­(indazolyl)­methane Isomer^a^

a Reaction conditions: (a) Indazole (0.5 mmol), solvent (2.0 mL), amine, methylenating agent, sealed Fisher-Porter vessel, under N_2_. (b) Number of equivalents relative to indazole. (c) Relative percentages were determined by integrating a known, isolated singlet signal for each compound in the ^1^H NMR spectrum (Figure S1, please see the Supporting Information). The relative amount of unreacted indazole was calculated by comparing the integration of its characteristic 1H NMR signals (Figure S2, please see the Supporting Information) with the total signal corresponding to the bis­(indazolyl)­methane isomers. Percentages in parentheses refer to the isolated yield of the pure isomer after chromatographic purification upon scaling up the reaction (×40).

When no base was added (entries 1–4), reaction progress was slow or negligible. The use of **pyridine** as an external base (entries 5–9), characterized by its low p*K*_a_H (≈5.2) and minimal steric hindrance, yielded high selectivity toward the thermodynamic isomer **L1**. Entry 7, scaled-up, provided **L1** in 87% isolated yield, indicating that **pyridine** not only acted as a mild base but also facilitated thermodynamic equilibration through transient acid-mediated isomerization.

In contrast, strongly basic and sterically hindered amines such as **Cy**_**2**_ **NMe** and **PMP** (entries 10–15) displayed strikingly different behavior. Although both have high p*K*_a_H (>10), **PMP**—more hindered and non-nucleophilic—selectively yielded the kinetic product **L2**, likely due to its ability to sequester HBr, thus preventing trace acid-catalyzed isomerization. This supports its role as a noninnocent base, beyond simple deprotonation.

In entries 16–31, we explored quaternary methylenating salts (**1-X**, **2-X**, **3-X**) combined with **Cy**_**2**_ **NMe** or **PMP** in THF. Under these more polar conditions, a shift in product distribution was observed. The major product shifted toward **L3**, particularly when less basic ammonium salts were combined with bulkier tertiary amines. **L3**, possessing intermediate thermodynamic stability, formed directly under these conditions without apparent isomerization to **L1** or **L2**.

Across all experiments, the combination of methylene donor, base structure, **p** ***K***_**a**_ **H**, and steric factors proved crucial in directing product selectivity. Notably, entries 27, 22, and 20 provided the best conditions for **L3** formation, each scaled-up to yield 73, 69, and 62%, respectively. The use of **Cy**_**2**_ **NMe** and **PMP** alongside halomethylated pyridinium species allowed tight control over regioselectivity and minimized unreacted starting material, facilitating purification.

### Crystallography

Crystals suitable for SCXRD analysis of the **L1**, **L2** and **L3** isomers of bis­(indazolyl)­methane were obtained by dissolving the compounds in dichloromethane and allowing *n*-hexane to diffuse slowly into the solution within connected, sealed vials.

The resulting crystals of **L1** and **L3** were colorless and plate-like, whereas **L2** formed irregular, colorless blocks. The crystal structures reveal both similarities and differences in symmetry among the isomers. All three bis­(indazolyl)­methane isomers crystallize in monoclinic systems. However, **L1** (space group: *C*2, with two symmetrically independent half-molecules in the asymmetric unit) and **L3** (space group: *P*2_1_) crystallize in achiral, yet Sohncke-type space groups. In contrast, **L2** (space group: *P*2_1_/c) crystallizes in a centrosymmetric space group. Both **L2** and **L3** contain a single molecule in the asymmetric unit. The key crystallographic and refinement data for the structures of **L1**, **L2**, and **L3** are provided in the Supporting Information summarized in Table S12.

Figure 3 presents the ORTEP^33^ crystal structures of the three bis­(indazolyl)­methane isomers, **L1**, **L2**, and **L3**. To enable a clear structural comparison between the isomers, the structures are depicted from two perspectives: a frontal view along the N–CH_2_–N axis bridging the two indazole rings, and a planar view relative to this axis. Additionally, the intercentroid distances and angles between the pyrazole rings are provided for each isomer. The bond lengths and angles observed in these crystal structures are consistent with those found in the parent indazole, as well as with the previously reported crystal structure of the **L1** isomer.^27^ Furthermore, these experimental values closely match the theoretical geometric parameters predicted for all three isomers. These comparisons are thoroughly detailed and illustrated in Figure S26 (see the Supporting Information).

**Figure 3:**
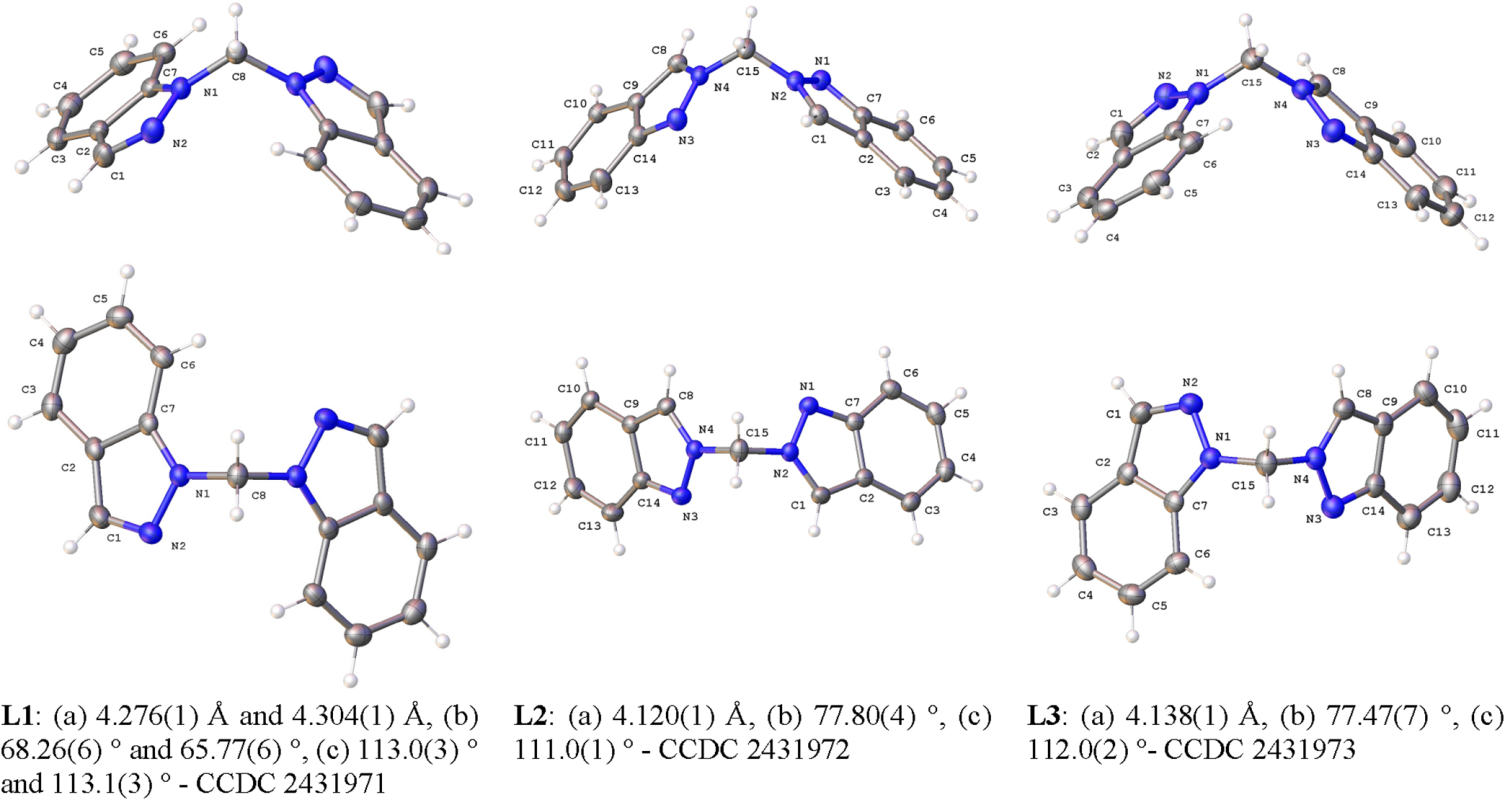
ORTEP representations of the crystal structures of bis­(indazolyl)­methane isomers **L1**, **L2**, and **L3**, with ellipsoids shown at 50% probability. (a) Distance between the centroids of the two pyrazole rings in indazoles. (b) Angle between the planes of the two indazoles rings. (c) Angle between the N–CH_2_–N bridging atoms. All values were calculated using least-squares analysis in SHELXL.

The perspective projections of the BINDM isomers (Figure 4) were generated from the CIF files obtained in this work using the ToposPro (5.5.3.1) program package.^34^ These visualizations highlight the characteristic packing motifs and intermolecular interactions for each crystalline isomer. The crystal structure of the symmetric bis­(indazolyl)­methane isomer, **L1**, features extended face-to-face π-stacking interactions along the crystallographic *b*-axis. These involve aromatic π–π interactions between neighboring indazole rings, with centroid-centroid distances between rings measuring approximately 4.100 Å (Figure 4a).

**Figure 4:**
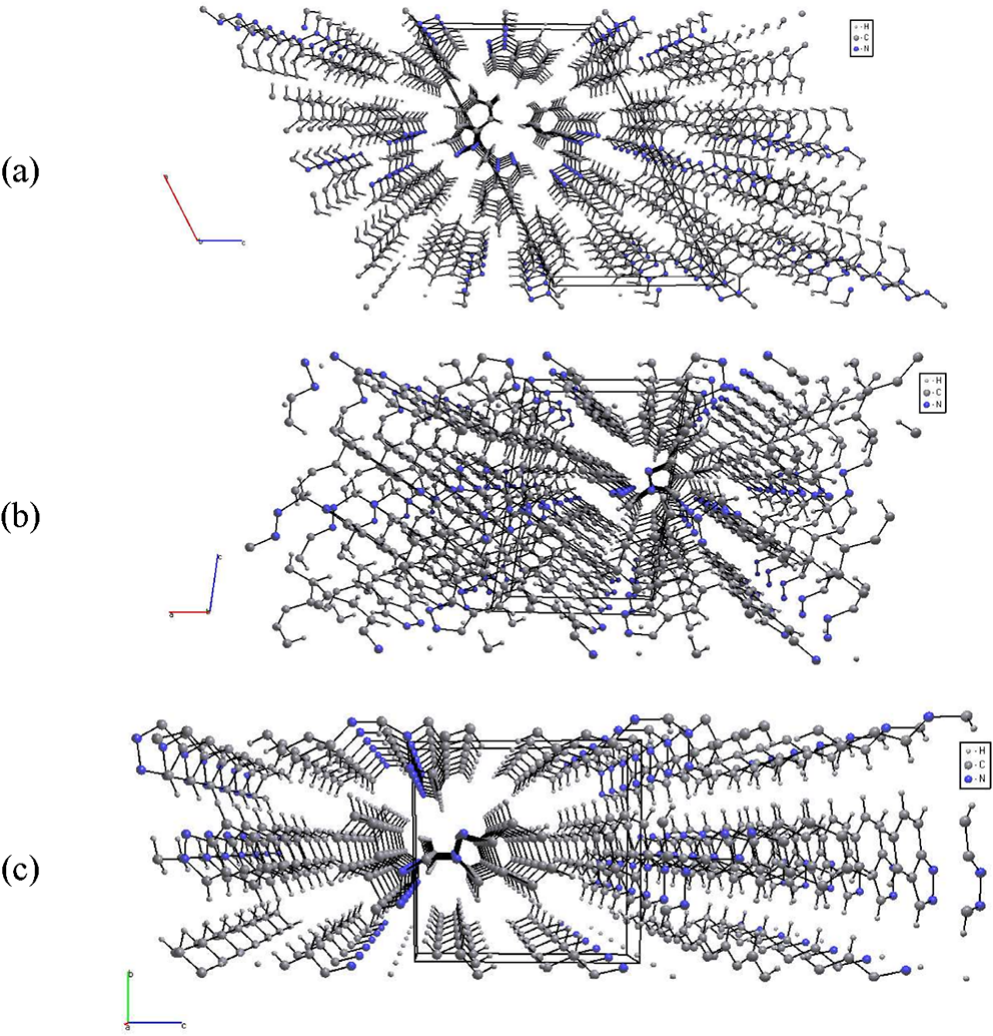
Perspective projections of the crystal packing of BINDM isomers: (a) **L1** viewed along the crystallographic *b*-axis; (b) **L2** viewed along the *b*-axis; (c) **L3** viewed along the *a*-axis.

In contrast, the crystal structure of the symmetric isomer **L2** exhibits more intricate packing arrangement. The molecules form alternating layers in which adjacent indazolyl groups display both edge-to-face interactions (between the centroid of one phenyl-pyrazolyl ring and a C–H hydrogen atom of a neighboring ring) and face-to-face π-stacking between antiparallel indazolyl moieties along the *b*-axis, with separations of about 2.717 Å and 3.647 Å, respectively (Figure 4b).

Finally, the crystal structure of the nonsymmetric isomer L3 extends via parallel face-to-face π-stacking along the *a-axis*, with ring centroid separations of approximately 4.506 Å (Figure 4c).

### Theoretical Study of Mechanism Selectivity

#### Structures of Compounds L1-L3

The three isomers of bis­(indazolyl)­methane, **L1**-**L3**, were optimized without symmetry restrictions at the B3LYP/6–311+G** level of theory and the resulting structures are shown in Figure 5.

**Figure 5:**
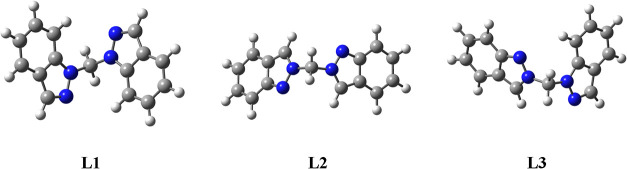
Optimized structures of compounds **L1**-**L3**.

The optimized geometries closely match the experimental results, particularly considering that the calculations were conducted for gas-phase molecules. The localization of the π system within the rings, as clearly observed in the experimental structures, is well described by calculations. The only notable discrepancy is the slight overestimation of the C–C bond length at the ring junction, while the remaining distances align within 0.02 Å. The N–CH_2_–N bond angle is consistently reproduced across all isomers, remaining close to 112° experimentally and approximately 113° in the optimized structures. A comparison of the selected computed and experimental geometric parameters for the three isomers is provided in Figure S26 (see the Supporting Information). From an energetic perspective, the **L1** isomer is the most stable (thermodynamic isomer). However, the **L3** and **L2** isomers are only slightly destabilized, with relative Gibbs free energy (Δ*G*) differences of 4.5 and 9.6 kcal·mol^–1^, respectively.

#### Preliminary Analysis of the Mechanism

The synthesis of compounds **L1-L3** proceeds through a two-step process. First, CH_2_Br_2_ reacts with one equivalent of indazole. In the second step, the resulting bromomethylindazole intermediate interacts with a second equivalent of indazole. To explain the bonding of the −CH_2_– fragment at the N2 atom of indazole (as seen in compounds **L2** and **L3**), it is necessary to consider the transformation of **1H-indazole** to **2H-indazole** before it attacks CH_2_Br_2_, assuming the reaction occurs without a deprotonating base. Although the energies of indazole, substituted indazoles, and their tautomers have been previously explored theoretically,^35–39^ to our knowledge their isomerization process has not been previously analyzed. For nonsubstituted indazole, a transition state (**TS**) was identified where **1H-indazole** converts to the **2H-indazole** through a hydrogen shift involving concomitant electronic redistribution within the aromatic indazole moiety (**TS**_**Ind**_ in Figure 6). In this **TS** the hydrogen atom is in the molecular plane, with distances of 1.209 Å to **N2** and 1.282 Å to **N1**, and an N–H–N angle of 71.6°. This TS lies 50.0 kcal·mol^–1^ higher in energy than **1H-indazole**, which is consistent with the absence of isomerization to **2H-indazole** at room temperature. The calculated energy difference between the **1H-** and **2H-indazole** tautomers (5.1 kcal·mol^–1^) is consistent with previous theoretical studies by others authors.^36^

**Figure 6:**
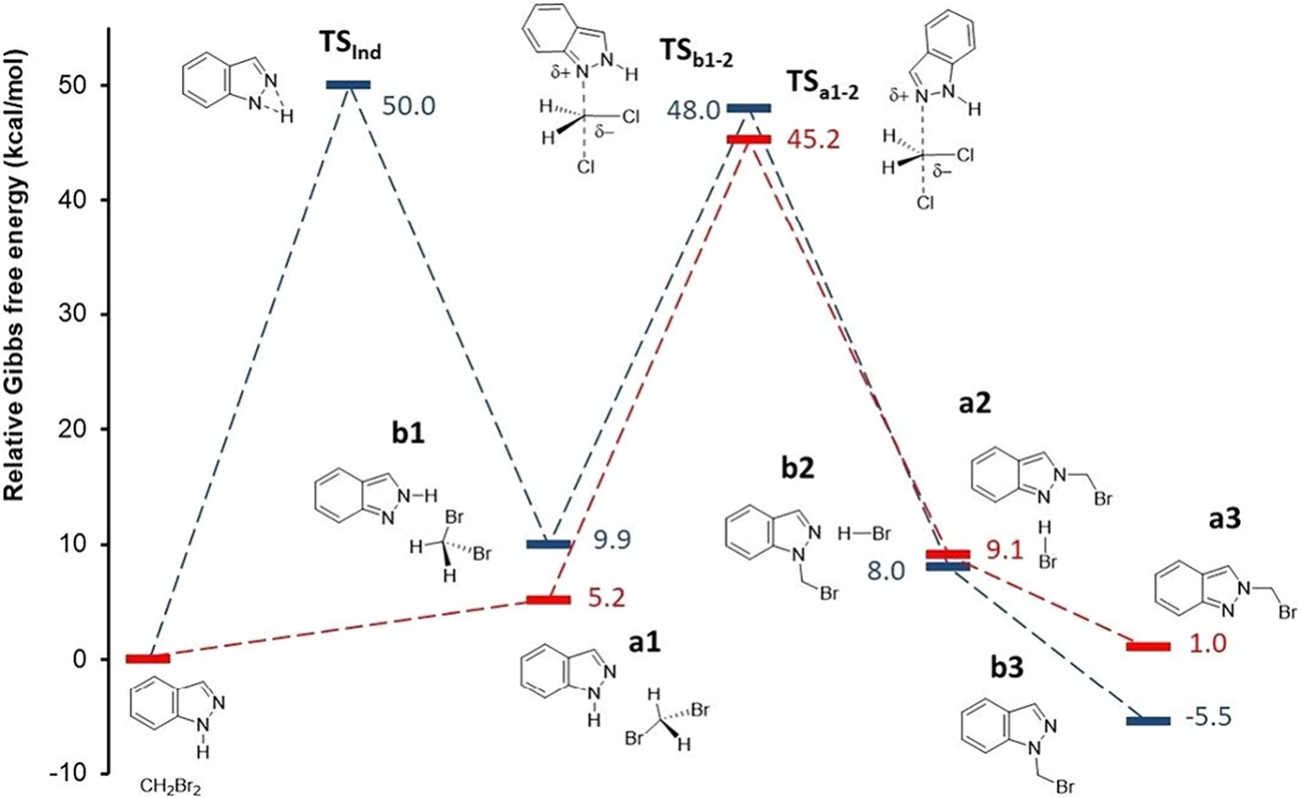
Relative energy profile for the formation of intermediates **1-(bromomethyl)-1H-indazole** (blue) and **2-(bromomethyl)-2H-indazole** (red) from the reaction of CH_2_Br_2_ with **1H-indazole** in the absence of base.

To determine the operative mechanism in this reaction, two possible pathways for the first step were examined: the interaction of CH_2_Br_2_ with **1H-indazole** and with **2H-indazole**, after isomerization. In the absence of a deprotonating base, both a concerted pathway and an S_N_2 mechanism were considered. For each route, two transition states for **1H-** and **2H-indazole** were identified (Scheme 3). The S_N_2 pathway’s relative Δ*G* energy was significantly lower than that of the concerted pathway, leading to the selection of the S_N_2 mechanism for further investigation in this transformation.

**Scheme 3:**
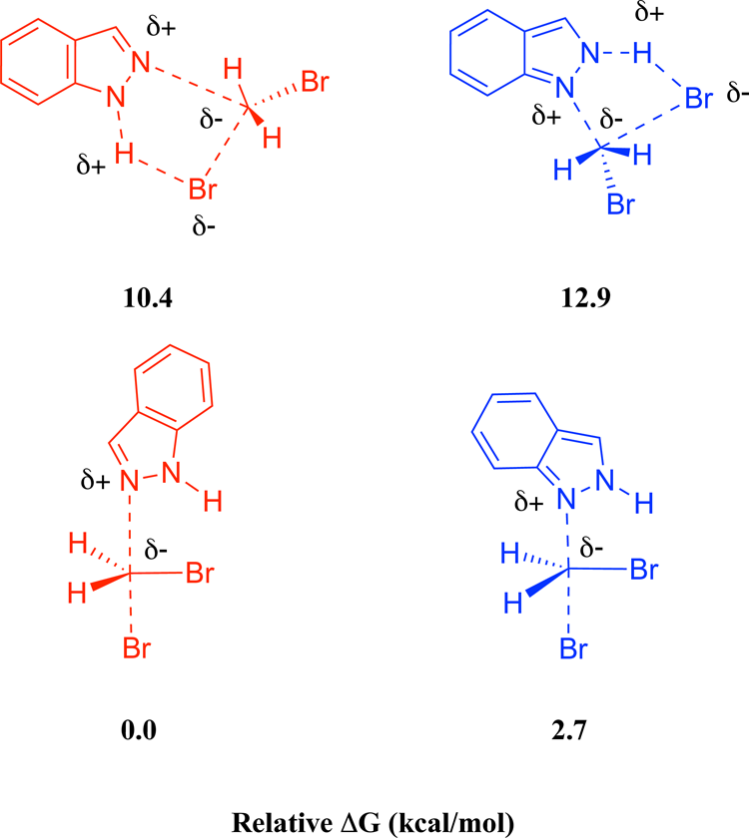
Comparison of the Relative Energies of the Transition States Located for Two Alternative Mechanisms — Concerted (**Top**) and S_N_2 (**Bottom**) — in the Interaction of CH_2_Br_2_ with **1H-** and **2H-indazole**

The selectivity arises from the use of a base that does not deprotonate **1H-indazole**. If the indazolide anion were formed, there would be no energetic difference between the two transition states corresponding to the S_N_2 attack of indazolide on CH_2_Br_2_ through the **N1** or **N2** atoms (Scheme 4). This would lead to a lack of selectivity, as experimentally observed when strong bases such as NaOH or NaH were used.^22^

**Scheme 4:**
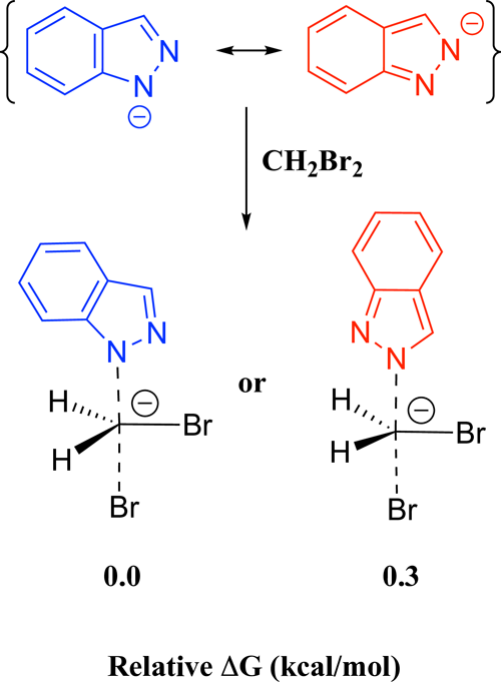
Comparison of the Relative Energies for the Transition States of the Reaction of Indazolide with CH_2_Br_2_: via the **N1** (left) or **N2** (right) Atoms

The reaction profile for forming **1-(bromomethyl)-1H-indazole** and **2-(bromomethyl)-2H-indazole** intermediates was first analyzed in the absence of a base (Figure 6). While **1-(bromomethyl)-1H-indazole** is the thermodynamic isomer, its formation requires overcoming two successive Δ*G* barriers of 50.0 and 38.1 kcal·mol^–1^, the first corresponding to indazole isomerization through **TS**_**Ind**_. In contrast, for the **2-(bromomethyl)-2H-indazole**, only a single Δ*G* barrier of 40.0 kcal·mol^–1^ (**TS**_**a1–2**_) is present, making it the kinetic isomer, though its formation is endergonic (1.3 kcal·mol-1). This result aligns with the lack of reactivity observed between **1H-indazole** and CH_2_Br_2_ in the absence of a base and corroborates previous experimental selectivity toward the kinetic intermediate.^22^ As discussed, the selectivity of the reaction is influenced by the choice of base. Therefore, the formation of these intermediates was analyzed in the presence of bases such as **pyridine** (**py**), **PMP**, and **NMeCy**_**2**_. The isomerization of **1H-indazole** to **2H-indazole** is favored in the presence of a base, significantly reducing the **TS**_**Ind**_ barrier (e.g., 21.1 kcal·mol^–1^ with **NMeCy**_**2**_ versus 50.0 kcal·mol^–1^ without base, Figure S27, see the Supporting Information). The formation of the compound **L1** from the **1-(bromomethyl)-1H-indazole** intermediate in the absence of an added base also involves the isomerization of **1H-indazole** via **TS**_**Ind**_, making **L1** the most kinetically hindered compound due to two additional barriers of 50.0 and 29.5 kcal·mol^–1^ (**TS**_**Ind**_ and **TS**_**b4–5**_ in Figure 7). Conversely, when the reaction starts from the **2-(bromomethyl)-**2H-indazole intermediate, the interaction with 1Hindazole and 2H-indazole produces compounds **L2** and **L3**, respectively (Figure 8). Again, indazole isomerization hinders the formation of **L3**, with barriers of 50.0 and 29.6 kcal·mol-1 (**TS**_**Ind**_ and **TS**_**b6–7**_, respectively), while **L2** is the preferred kinetic isomer in this second step of the reaction (only a barrier of 34.9 kcal·mol^–1^ through **TSa**_**4–5**_).

**Figure 7:**
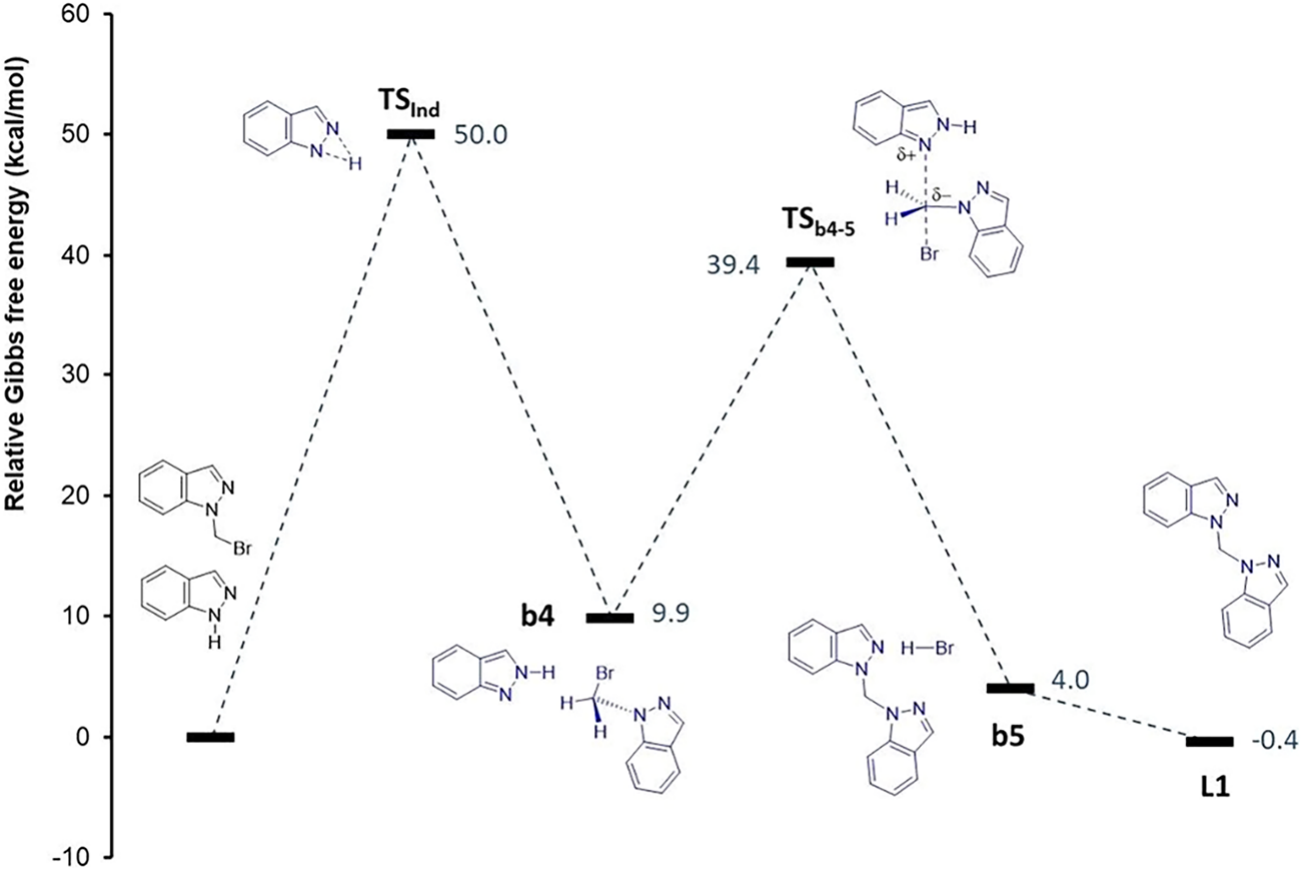
Relative energy profile for the formation of the compound **L1** from the reaction of the **1-(bromomethyl)-1H-indazole** intermediate with **1H-indazole** in the absence of base.

**Figure 8:**
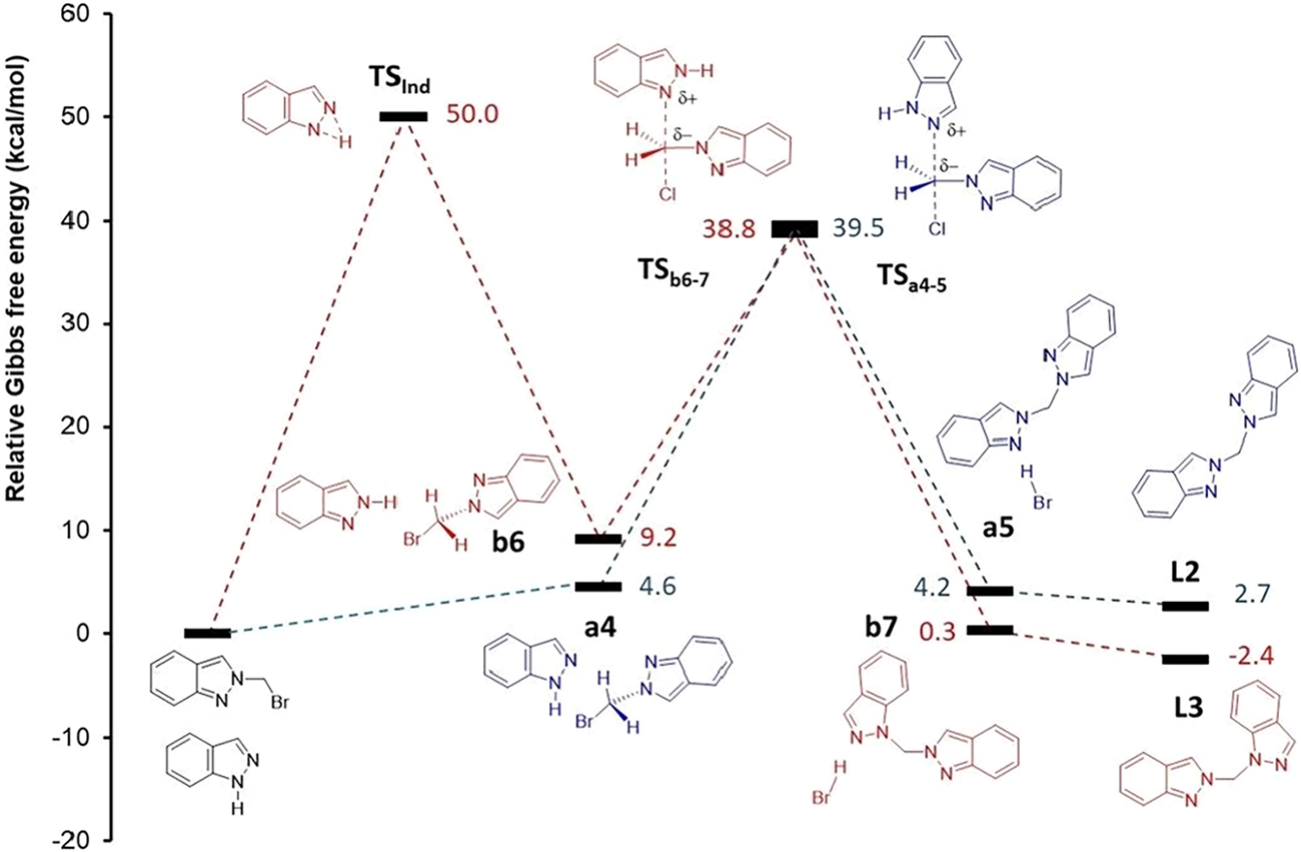
Relative energy profile for the formation of compounds **L2** (blue) and **L3** (red) from the reaction of the **2-(bromomethyl)-2H-indazole** intermediate with **1H-indazole** and **2H-indazole**, respectively, in the absence of base.

#### Proposed Mechanism for L2 Formation

Compound **L2** was selectively formed in the reaction of **1H-indazole** with CH_2_Br_2_ in the presence of the base **PMP**. Based on the previous preliminary analysis, **L2** is identified as the kinetic isomer, and thus, shorter reaction times are necessary for its formation compared to **L1**. The complete mechanism (Scheme S3, see the Supporting Information) was analyzed with the inclusion of **PMP** in the calculations, and its energetic profile is shown in Figure 9. The first transition state, **TS**_**a1–2(PMP)**_, corresponds to the nucleophilic attack of indazole on the CH_2_Br_2_ molecule. In this transition, the **N2** atom of the indazole approaches the carbon atom of CH_2_Br_2_ (1.963 Å), simultaneously elongating one of the C–Br bonds (2.681 Å).

**Figure 9:**
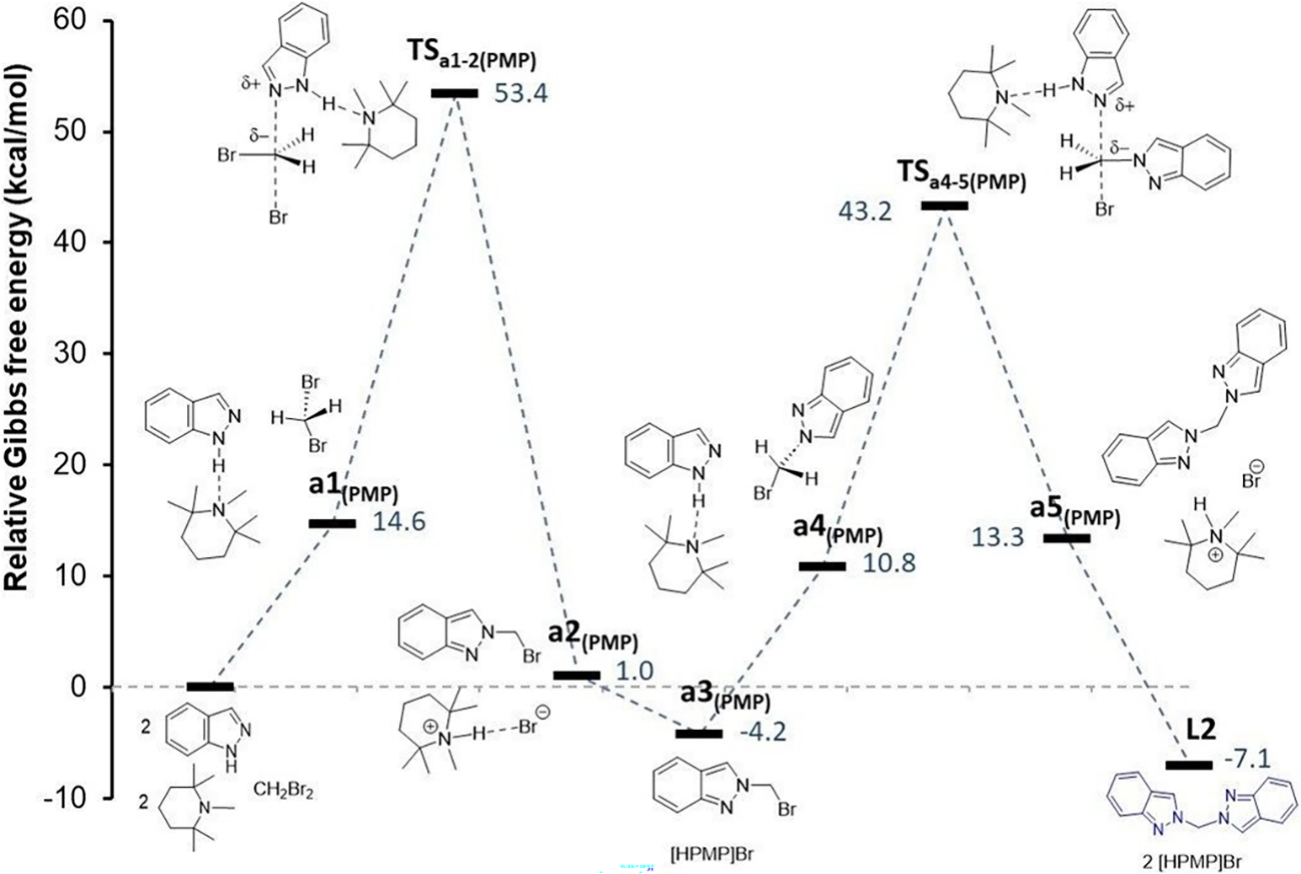
Relative energy profile for the formation of **L2** from the reaction of CH_2_Br_2_ with **1H-indazole** in the presence of the **PMP** base.

Indazole is stabilized by **PMP** via a hydrogen bond (**N1–H** ^**...**^ **N**_**(PMP)**_ at 1.987 Å). This transition state has a Δ*G* energy difference of 38.8 kcal·mol^–1^ with respect to **a1**_**(PMP)**_. The next intermediate **a2**_**(PMP)**_, corresponds to **2-(bromomethyl)-2H-indazole** plus the **[HPMP]­Br** salt. This intermediate subsequently yields **2-(bromomethyl)-2H-indazole**, **a3**_**(PMP)**_, after elimination of the **[HPMP]­Br** salt that precipitates in the reaction medium.

The second nucleophilic attack of indazole occurs on this intermediate, forming the second transition state, **TS**_**a4–5(PMP)**_, with a Δ*G* difference of 32.4 kcal·mol^–1^ relative to **a4**_**(PMP)**_. In this case, the **N2** atom of the second indazole molecule approaches the carbon atom of **2-(bromomethyl)-2H-indazole** (1.946 Å), while the C–Br bond elongates (2.776 Å). The second indazole is also stabilized by **PMP** through a hydrogen bond (**N1–H** ^**...**^ **N**_**(PMP)**_ at 1.984 Å). This transition state leads to an adduct of compound **L2** with **[HPMP]** ^**+**^, **a5**_**(PMP)**_, characterized by a weak **N1** ^**...**^ **H–N**_**(PMP)**_ interaction (2.246 Å). After the elimination of **[HPMP]­Br**, compound **L2** is formed with a relative Δ*G* energy of −7.1 kcal·mol^–1^.

The selectivity for **L2** over the other isomers arises from two main factors, as shown in Figure S28 (see the Supporting Information), which compare the relative energy profiles for the formation of the intermediates **1-(bromomethyl)-1H-indazole** and **2-(bromomethyl)-2H-indazole** from the reaction of CH_2_Br_2_ with **1H-indazole** in the presence of **PMP**. The first factor, as mentioned earlier, is the absence of indazole isomerization on the pathway to **L2**. The second factor is the higher relative energy calculated for **TS**_**b1–2(PMP)**_, the transition state analogous to **TS**_**a1–2(PMP)**_ but with **2H-indazole** instead of **1H-indazole**. The same destabilization is observed in the transition state for the second attack of **2H-indazole** on **2-(bromomethyl)-2H-indazole** in the presence of **PMP**.

#### Proposed Mechanism for L1 Formation

Based on the preliminary analysis of the reaction profile in the absence of base, the synthesis of **L1** would require high temperature and long reaction times, primarily due to the isomerization of indazole and higher transition state barriers, in agreement with the experimental results. The complete mechanism for the formation of **L1** in the presence of **pyridine** was calculated (Scheme S4, see the Supporting Information), and its energy

profile is shown in Figure S29 (see the Supporting Information). However, in this profile, selectivity toward **L1** is not observed, as some of the transition states calculated in the presence of **pyridine** favor the formation of the **L2** isomer.

In fact, mixtures of **L1**-**L3** compounds were experimentally observed when the reaction was conducted with shorter reaction times. The **L2** or **L3** formed during this period can be converted into **L1** by simply heating the reaction mixture under the reaction conditions. The isomerization of **L2** or **L3** isomers to **L1** only occurred when small amounts of acid were present. Pyridine plays an additional role in this reaction, as the pyridinium cation produced during the process is sufficiently acidic to facilitate the isomerization under these conditions. This isomerization process has been theoretically analyzed (Figure 10).

**Figure 10:**
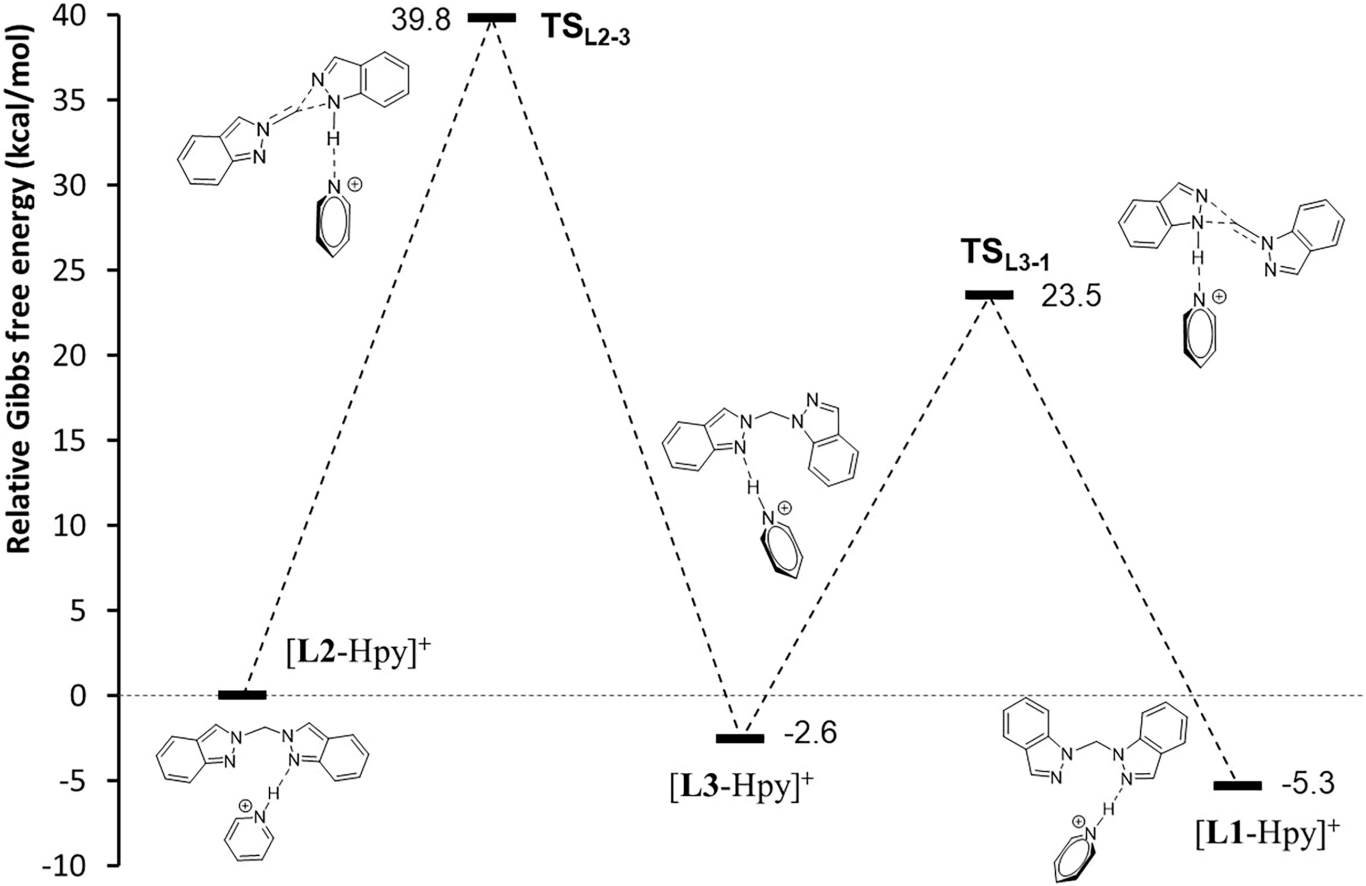
Relative energy profile for the isomerization process toward compound **L1** from its isomers **L2** and **L3** in the presence of **[Hpy]** ^**+**^ cation.

Starting from the less stable isomer **L2**, which forms a 1:1 adduct with the **[Hpy]** ^**+**^ cation, the conversion to **L3** occurs via the transition state **TS**_**L2–3**_, involving a intramolecular shift of the **2-methylene-2H-indazolium** group. The energy barrier for this transformation is 39.8 kcal·mol^–1^. In this transition state, the C–N bond distance of the methylene group in the **2-methylene-2H-indazolium** fragment shortens to 1.302 Å (compared to 1.447 Å in **[L2**-Hpy**]** ^**+**^), while the C–N separations to the **N1** and **N2** atoms of the indazole fragment lengthen to 2.793 Å and 2.676 Å, respectively. Additionally, the hydrogen atom of pyridinium shifts toward indazole, reducing the N–H bond length to 1.041 Å (from 1.699 Å in **[L2**-Hpy**]** ^**+**^).

Subsequently, **[L3**-Hpy**]** ^**+**^ isomerizes to **[L1**-Hpy**]** ^**+**^, the most stable isomer, through the transition state **TS**_**L3–1**_. This transition also involves an intramolecular-shift of the **1-methylene-1H-indazolium** group, with a lower energy barrier of 26.1 kcal·mol^–1^ (Figure 10). In this case, the C–N bond of the methylene group in the **1-methylene-1H-indazolium** fragment shortens to 1.290 Å, while the C–N separations to the **N1** and **N2** atoms of the indazole fragment lengthen to 2.955 and 3.047 Å, respectively. The H atom of pyridinium moves closer to indazole, with an N–H bond length of 1.036 Å. Significantly higher energy barriers were calculated for this isomerization process in the absence of acid, as shown in Figure S30 (see the Supporting Information).

#### Proposed Mechanism for L3 Formation

After screening various reagents and reaction conditions, it was concluded that compound **L3** can be selectively prepared through the reaction of **[CH**_**2**_ **py**_**2**_ **]­Br**_**2**_ with **1H-indazole** in the presence of the **NMeCy**_**2**_ base. Notably, compound **L3** is neither the kinetic nor thermodynamic isomer. Consequently, the proposed mechanism differs from the preliminary analysis mentioned earlier, although it still requires the inclusion of the **NMeCy**_**2**_ molecule in the calculations (Scheme S5, see the Supporting Information).

In this profile (Figure 11), to maintain charge neutrality in the computational model, the **[CH**_**2**_ **py**_**2**_ **]** ^**2+**^ cation was used instead **[CH**_**2**_ **py**_**2**_ **]­Br**_**2**_ species. The first step is the exergonic reaction between **[CH**_**2**_ **py**_**2**_ **]** ^**2+**^ and **NMeCy**_**2**_, which produces the dication **[CH**_**2**_ **py­(NMeCy**_**2**_ **)]** ^**2+**^ (**L3b**). This reaction occurs through the transition state **TS**_**L3a‑b**_, with an energy barrier of 18.9 kcal·mol^–1^, where the **NMeCy**_**2**_ molecule moves toward the carbon atom of **[CH**_**2**_ **py**_**2**_ **]** ^**2+**^ (at 2.268 Å) while one of the pyridine groups moves away (with a **C–py** bond distance of 2.084 Å).

**Figure 11:**
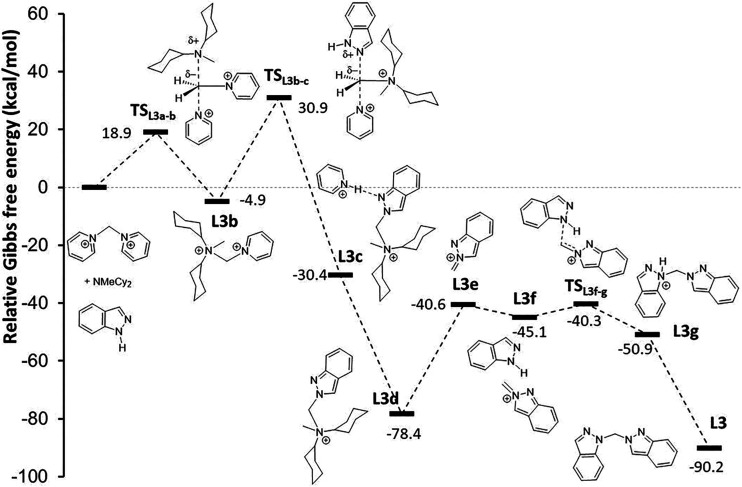
Relative energy profile for the formation of **L3** from the reaction of **[CH**_**2**_ **py**_**2**_]^**2+**^ with **1H-indazole** in the presence of the **NMeCy**_**2**_ base.

The resulting dication intermediate then undergoes a nucleophilic attack by **1H-indazole**. This step is characterized by the transition state **TS**_**L3b‑c**_, which lies 35.8 kcal·mol^–1^ higher than the dication intermediate **L3b**. This transition state leads to the formation of **L3c**, identified as **N-((2H-indazol-2-yl)­methyl)-N-cyclohexyl-** *N* **-methylcyclohexanaminium**, which interacts with the pyridinium cation (with a **py–H** ^**...**^ **N** distance of 1.884 Å). The elimination of pyridinium as bromide salt produces the intermediate **L3d**. Subsequently, the dissociation of **NMeCy**_**2**_ forms the **2-methylene-2H-indazolium** cation (**L3e**).

Following this, **L3e** interacts with a second indazole molecule (**L3f**), initiating a nucleophilic attack by **1H-indazole** through the transition state **TS**_**L3f‑g**_, which has a small energy barrier. This step results in the formation of **1-((2H-indazol-2-yl)­methyl)-1H-indazol-1-ium** (**L3g**). The potential interaction between **L3e** and the **N2** atom of indazole would involve a transition state with an energy which is 12.9 kcal·mol^–1^ higher than **TS**_**L3f‑g**_, thereby preventing the formation of the **L2** isomer. Finally, the proton is removed by the **NMeCy**_**2**_ base, yielding compound **L3** in an exergonic process.

## Conclusions

This study presents an efficient and regioselective synthetic strategy that enables the controlled preparation of each of the three bis­(indazolyl)­methane isomers in a single operational step. The methodology is underpinned by a comprehensive understanding of the chemical reactivity, supported by both experimental observations and computational insights.

A key finding is the decisive role of the selected amines, whose steric and electronic properties modulate the reaction pathway. These noninnocent amines influence both the thermodynamic and kinetic landscapes of the process, offering a handle to fine-tune the selectivity. Additionally, the nature of the methylene transfer agent, in combination with the base, was found to be equally critical in directing the reaction toward a desired regioisomer.

The successful isolation and crystallographic characterization of all three regioisomers provided unambiguous structural confirmation of the products, further validating the synthetic methodology and the proposed structure–reactivity relationships.

Altogether, this work introduces a rational and versatile approach for regioselective synthesis in heterocyclic chemistry. Beyond its immediate relevance to indazole chemistry, the strategy holds broad potential for guiding the selective construction of complex organic scaffolds relevant to coordination chemistry, material science, and pharmaceutical development.

## Experimental Section

### Overview

A concise description of the general methods and some representative procedures are provided here for completeness; full experimental details, complete characterization data (^1^H, ^13^C, 2D NMR, FTIR, ESI-MS, elemental analysis), crystallographic information, and additional procedures are compiled in the Supporting Information.

### General

All procedures and manipulations were performed under a dry, oxygen-free nitrogen atmosphere using standard Schlenk or glovebox techniques, unless explicitly stated otherwise. Solvents were distilled under nitrogen using the following drying agents: sodium/benzophenone ketyl for diethyl ether (Et_2_O) and tetrahydrofuran (THF); sodium for pentane and toluene; and calcium hydride (CaH_2_) for dichloromethane (CH_2_Cl_2_) and acetonitrile (CH_3_CN). All solvents were degassed prior to use. Solution nuclear magnetic resonance (NMR) spectra were recorded using Bruker DRX-400 (400 MHz), AVANCE III/ASCEND 400R (400 MHz), and AVANCE III (500 MHz) instruments at the IIQ. ^1^H and ^13^C chemical shifts were referenced to residual signals of the deuterated solvents, with all data reported in parts per million (ppm) downfield from tetramethylsilane (Me_4_Si). Coupling constants (*J* values) are given in Hertz (Hz). The following abbreviations are used to designate multiplicities: **s** = singlet, **d** = doublet, **t** = triplet, **q** = quartet, **quin** = quintuplet, **sext** = sextet, **sep** = septet, **m** = multiplet, **br** = broad, **dd** = double–doublet, ddd = double–double–doublet. Fourier-transform infrared spectra (FTIR) were recorded using a Bruker Tensor 27 spectrometer (IIQ). The following abbreviations denote the relative transmittance intensity of the observed peaks, with the vertical axis representing transmittance and the horizontal axis showing frequencies as wavenumbers (cm^–1^): **vs** = very strong, **s** = strong, **m** = medium, and **w** = weak. As the novel compounds described in this manuscript do not contain functional groups (such as alcohols or acids) that typically produce broad FTIR peaks, all peak widths mentioned below refer to those considered sharp. Mass spectrometry analysis (MS) with an electrospray ionization (ESI) source, as well as elemental analyses, were carry out by the Instrumentation Services at IIQ (Mass Spectrometry and Analytical Services) using a Bruker Esquire 6000 Ion Trap Mass Spectrometer or a Bruker AmaZon SL Ion Trap LC/MS instrument, and a LECO True-Spec CHNS elemental analyzer, respectively. Single-crystal X-ray diffraction data were collected using a Bruker-Nonius X8 Apex-II diffractometer (IIQ) or a Bruker-AXS D8 QUEST ECO diffractometer (IIQ).

General method used for the syntheses of bis­(indazolyl)­methane isomers as detailed in Table 1. The experiments were conducted in a sealed Fischer–Porter vessel equipped with a pressure gauge and subjected to magnetic stirring. One equivalent of anhydrous indazole was dissolved in the appropriate volume of anhydrous, degassed solvent and introduced into the vessel at room temperature under a nitrogen atmosphere. To this solution, the required volume of the selected amine was added, followed by the apprpriate equivalents of the methylenating agent. Once the vessel was sealed, the mixture was stirred at the specified temperature for the designated time, as detailed in Table 1. The progress of the reaction was monitored by cooling the Fischer–Porter vessel to room temperature and sampling aliquots that were as representative as possible of the reaction mixture, which was often heterogeneous due to the formation of ammonium salts. After hydrolyzing the sample with distilled water, extracting with dichloromethane, and drying over anhydrous Na_2_SO_4_, the solvent was removed under reduced pressure using a rotary evaporator. The reaction progress was assessed through ^1^H NMR analysis, which indicated the presence of unreacted indazole and the ratio of isomers of bis­(indazolyl)­methane formed (See Figure S1 in the Supporting Information for comparative analysis of the 1H NMR spectra).

### Regioselective Synthesis of Di­(1H-indazol-1-yl)­methane

(**L1**). A solution of 2.36 g (20 mmol) of dry indazole in 80 mL of anhydrous toluene was prepared in a 250 mL Fischer–Porter flask under an inert atmosphere of nitrogen or argon, followed by the addition of 3.9 mL (48 mmol) of dry **pyridine**. After magnetic stirring for 5 min at room temperature, 5.6 mL (80 mmol) of CH_2_Br_2_ was added. The flask was then sealed, and the reaction mixture, maintained under an inert atmosphere with continuous stirring, was heated at 150 °C for 48 h. After cooling the Fischer–Porter vessel to room temperature, the reaction was quenched by the addition of 100 mL of distilled water and extracted with three 100 mL portions of ethyl acetate. The combined organic layers were washed with two 50 mL portions of brine, dried over anhydrous Na_2_SO_4_, filtered, and concentrated under reduced pressure using a rotary evaporator. The resulting residue was purified by flash chromatography using a 1:10 mixture of diethyl ether and *n*-hexane as eluent (see Section 1.3, page S9 of the Supporting Information for further details), affording 2.16 g of purified **L1** (87% yield). **Mp**: 150.2 °C. **Elem. Anal. Calcd** (%) for C_15_H_12_N_4_: C, 72.56; H, 4.87; N, 22.57. **Found**: C, 72.13; H, 4.87; N, 22.55. **FTIR** (KBr, cm^–1^): 3061 (m), 1617 (w), 1499 (m), 1463 (m), 1439 (w), 1418 (w), 1361 (s), 1280 (m), 1205 (s), 1005 (w), 934 (m), 909 (m), 828 (m), 761 (m), 736 (s). ^**1**^ **H NMR** (500 MHz, CDCl_3_): d = 8.01 (d, *J* = 0.9 Hz, 2H, H3), 7.83 (dtd, *J* = 8.4, 0.9, 0.7 Hz, 2H, H8), 7.67 (dt, *J* = 8.0, 0.9 Hz, 2H, H5), 7.40 (ddd, *J* = 8.4, 7.4, 0.9 Hz, 2H, H7) 7.15 (ddd, *J* = 8.0, 7.4, 0.7 Hz, 2H, H6) 6.90 (s, 2H, H1). ^**13**^ **C NMR** (125 MHz, CDCl_3_): d = 139.62 (C9), 134.61 (C3), 127.20 (C7), 124.90 (C4), 121.60 (C6), 121.13 (C5), 110.18 (C8), 61.76 (C1). **MS** (ESI +, *m*/*z*): [M + Na] ^+^: 271.1, [2 M + Na] ^+^: 519.1.

### Regioselective Synthesis of Di­(2H-indazol-2-yl)­methane

(**L2**). A solution of 2.36 g (20 mmol) of dry indazole in 80 mL of anhydrous toluene was prepared in a 250 mL Fischer–Porter flask under an inert atmosphere of nitrogen or argon, followed by the addition of 8.7 mL (48 mmol) of dry **PMP**. After magnetic stirring for 5 min at room temperature, 11.22 mL (160 mmol) of CH_2_Br_2_ was added. The flask was then sealed, and the reaction mixture, maintained under the inert atmosphere with constant stirring, was heated at 150 °C for 48 h. After cooling the Fischer–Porter vessel to room temperature, the reaction was quenched by the addition of 100 mL of distilled water and extracted with three 100 mL portions of ethyl acetate. The combined organic layers were washed with two 50 mL portions of brine, dried over anhydrous Na_2_SO_4_, filtered, and concentrated under reduced pressure using a rotary evaporator. The resulting residue was purified by flash chromatography, eluting with a 1:3 mixture of diethyl ether and *n*-hexane (see Section 1.3, page S10 of the Supporting Information for further details), affording 1.9 g of purified **L2** (77% yield). **Mp**: 179 °C. **Elem. Anal**. Calcd (%) for C_15_H_12_N_4_: C, 72.56; H, 4.87; N, 22.57. Found: C, 72.00; H, 4.90; N, 22.00. **FTIR** (**KBr**, cm^–1^): 2974 (w), 2290 (w), 1946 (m), 1919 (m), 1820 (s), 1794 (m), 1708 (vs), 1626 (w), 1561 (vs), 1514 (vs), 1470 (m), 1372 (m), 1326 (m), 1286 (m), 1240 (m), 1204 (m), 1134 (m), 1013 (m), 990 (m), 976 (m), 952 (m), 909­(m). ^**1**^ **H NMR** (**500 MHz, CDCl**_**3**_): d = 8.27 (dd, *J* = 0.3, 0.2 Hz, 2H, H3′), 7.69 (dtd, *J* = 8.8, 0.8, 0.1 Hz, 2H, H8′), 7.61 (ddt, *J* = 7.0, 0.8, 0.2 Hz, 2H, H5′), 7.29 (ddd, *J* = 8.8, 7.2, 0.8 Hz, 2H, H7′) 7.07 (ddd, *J* = 7.2, 7.0, 0.8 Hz, 2H, H6′), 6.85 (s, 2H, H1). ^**13**^ **C NMR** (**125 MHz, CDCl**_**3**_): d = 149.69 (C9′), 127.23 (C7′), 123.80 (C3′), 122.75 (C6′), 122.50 (C4′), 120.73 (C5′), 117.88 (C8′), 68.98 (C1). **MS** (ESI+, *m*/*z*): 249.1 [M + H^+^], 271.1 [M + Na^+^], 288.3 [M + K^+^], 519.1 [2 M + Na^+^].

### Regioselective Synthesis of (1H-indazol-1-yl)­(2H-indazol-2-yl)­methane (L3)

A total of 4.80 g (24 mmol) of [(C_6_H_7_N)_2_CH_2_]­Br_2_ (**2-Br**) was mixed with 70 mL of anhydrous tetrahydrofuran (THF), followed by the addition of 17.1 mL (80 mmol) of dry **Cy**_**2**_ **NMe**. The mixture was prepared in a 250 mL Fischer–Porter flask under an inert atmosphere of nitrogen or argon. After magnetic stirring for 5 min at room temperature, a solution of 2.36 g (20 mmol) of dry indazole in 10 mL of THF was added. The flask was then sealed, and the reaction mixture, under the inert atmosphere and constant stirring, was heated at 120 °C for 48 h. After cooling the Fischer–Porter vessel to room temperature, the reaction was quenched by the addition of 100 mL of distilled water and extracted with three 100 mL portions of ethyl acetate. The combined organic layers were washed with two 50 mL portions of brine, dried over anhydrous Na_2_SO_4_, filtered, and concentrated under reduced pressure using a rotary evaporator. The resulting residue was purified by flash chromatography, eluting with a 1:5 mixture of diethyl ether and *n*-hexane (see Section 1.3, page S10 of the Supporting Information for further details), affording 1.8 g of purified **L3** (73% yield). **Mp**: 117 °C. **Elem. Anal. Calcd**. (%) for C_15_H_12_N_4_: C, 72.56; H, 4.87; N, 22.57. **Found**: C, 72.56; H, 4.91; N, 22.44. **FTIR** (KBr, cm^–1^): 3101 (m), 3058 (m), 2928 (s), 2852 (m), 1939 (w), 1629 (m), 1618 (m), 1517 (s), 1503 (s), 1468 (s), 1435 (s), 1412 (m), 1388 (m), 1364 (w), 1317 (m), 1284 (m), 1245 (w), 1231 (w), 1173 (m), 1151 (m), 1135 (s), 1115 (s), 1069 (m), 1006 (w), 958 (s), 941 (m), 910 (m), 872 (m), 847 (m), 831 (m), 782 (s), 771 (s), 763 (m), 746 (m), 710 (s). ^**1**^ **H NMR** (500 MHz, CDCl_3_): d = 8.08 (d, J = 0.8 Hz, 1H, H3), 8.06 (d, J = 0.7 Hz, 1H, H3́), 7.77 (dq, J = 8.5, 0.8 Hz, 1H, H8), 7.71 (dt, J = 6.3, 0.8 Hz, 1H, H5), 7.70 (dq, J = 7.5, 0.7 Hz, 1H, H8′), 7.57 (dt, J = 7.5, 0.7 Hz, 1H, H5́), 7.44 (ddd, J = 8.5, 7.5, 0.8 Hz, 1H, H7), 7.25 (ddd, J = 9.0, 7.5, 0.7 Hz, 1H, H7′), 7.19 (ddd, J = 7.5, 6.3, 0.8 Hz, 1H, H6), 7.04 (ddd, J = 9.0, 7.5, 0.7 Hz, 1H, 6′), 6.89 (s, 2H, H1). ^**13**^ **C NMR** (125 MHz, CDCl_3_): d = 149.05 (C9́), 139.86 (C9), 135.84 (C3), 127.66 (C7), 126.70 (C7́), 124.99 (C4), 122.60 (C3́), 122.52 (C4́), 122.42 (C6′), 122.01 (C6), 121.32 (C5), 120.57 (C5́), 117.99 (C8′), 109.84 (C8), 64.68 (C1). **MS** (ESI+, *m*/*z*): 249.1 [M + H^+^], 271.1 [M + Na^+^].

### Crystallographic Data

A summary of the crystallographic structure refinement data for the 14 crystalline compounds discussed in this article is provided in Tables S3–S13 of the Supporting Information. Crystals of suitable size for X-ray diffraction analysis were coated with dry perfluoropolyether and mounted on glass fibers, then positioned on the goniometer head under a cold nitrogen stream (T = 193 K). Data were collected using either a Bruker-Nonius X8 Apex-II diffractometer equipped with a CCD area detector or a Bruker-AXS D8 QUEST ECO diffractometer equipped with a PHOTON II area detector. Monochromatic Mo Kα radiation (λ = 0.71073 Å) was employed, and data were acquired through ω and φ scans with a step width of 0.5°. Data reduction was performed using the SAINT software, and absorption corrections were applied using the multiscan method (SADABS), both integrated within Bruker’s APEX5 ^40^ crystallographic software suite. Structure solution was achieved using intrinsic phasing (SHELXT),^41^ also included in the APEX5 package, and refinement was conducted against all *F* ^2^ data by full-matrix least-squares methods (SHELXL-2018/3),^42^ minimizing *w*[*F*_0_^2^ *-F*_c_^2^],^2^ with the aid of the OLEX2–1.5 ^33^ crystallographic software package. All non-hydrogen atoms were refined anisotropically, whereas hydrogen atoms were placed in calculated positions and refined using a riding model with isotropic displacement parameters. Crystallographic data for the compounds reported herein have been deposited with the Cambridge Crystallographic Data Centre (CCDC) under the following accession numbers: CCDC 2431961 (**1-Cl**); 2431962 (form **a**) and 2431963 (form **b**) [**1-Br**, two crystalline polymorphs]; 2431964 (**1-I**); 2431965 (**2-Cl**); 2431966 (**2-Br**); 2431967 (**2-I**); 2431968 (**3-Cl**); 2431969 (**3-Br**); 2431970 (**3-I**); 2431971 (**L1**); 2431972 (**L2**); 2431973 (**L3**); and 2431974 (**Ind–N2-Ac**). These data are available free of charge from the CCDC via www.ccdc.cam.ac.uk/structures

### Theoretical Study

The electronic structure and geometries of all compounds were calculated using density functional theory (DFT) at the **B3LYP** level,^43,44^ with the 6–311+G** basis set for all atoms. Molecular geometries of all model complexes were optimized without symmetry constraints. Frequency calculations were performed at the same level of theory to identify all of the stationary points as either **transition states** (one imaginary frequency) or **minima** (zero imaginary frequencies) and to provide the thermal correction to free energies at **298.15 K** and **1 atm**. In some cases, a structure was considered a minimum despite a very low imaginary frequency (<10 cm^–1^), possibly due to the use of an insufficiently large integration grid.^45,46^ For certain cases, solution-phase **SCF** energies for intermediates and transition states were obtained via **single point calculation** on the gas-phase optimized structure using the **PCM solvation model** in toluene or THF.^47^ In these cases, the **Gibbs free energies in solution** were estimated using the equation G_solv_ = *E*_solv_ + (*G*_gas_ – *E*_gas_). Including solvent effects in DFT calculations using the **PCM model** for various reaction mechanisms investigated for us and others did not significantly alter the energetic reaction profile (1–3 kcal·mol^–1^ deviations between gas-phase and solvent-phase free energies).^48–50^ Therefore, most calculations were performed in the gas phase, and an in-solution description was not attempted. The energy profiles are presented in terms of **relative free energies** derived from thermochemical analysis. DFT calculations were carry out using the Gaussian 09 software suite.^51^ The coordinates of all optimized compounds and other energy profiles are reported in the Table S14 of the Supporting Information.

## Supplementary Material





## Notes

Experimental procedures; analytical and spectroscopic data including 1H, 13C, and 2D NMR spectra; FTIR spectra; ESI-MS and elemental analyses; complete crystallographic information (data collection, refinement details, and structural figures); theoretical calculation diagrams and cartesian coordinates of optimized compounds (PDF)
Crystallographic information files of the crystalline compounds described in this work (CIF)

## #

Institut für Chemie, Humboldt-Universität zu Berlin, Brook-Taylor-Straße 2, 12489 Berlin, Germany

## ⊥

Research Unit of Noxious Chemistry and Environmental Engineering (RUNOCHEE), Department of Chemistry, Faculty of Science, University of Dschang, P.O. Box 67, Dschang, Cameroon.
