# Supplementary material for: Regioselective Syntheses of Bis(indazolyl)methane Isomers: Controlling Kinetics and Thermodynamics via Tunable Non-Innocent Amines
Source: ACS Omega. 2025 Nov 14;10(46):56240–54. doi: 10.1021/acsomega.5c08094 (PMC12658713; doi:10.1021/acsomega.5c08094)
Supplement: Supplementary file 1 [file ao5c08094_si_001.pdf]

Supporting Information

Regioselective Syntheses of Bis(indazolyl)methane Isomers: Controlling Kinetics and Thermodynamics via Tunable Non-Innocent Amines

María Álvarez-Sánchez,[a‡§] *Margarita Gómez,*[a‡] *Carina Santos Hurtado,*[a] *Jean Ngouné,*[b∥] *Eleuterio Álvarez,*[a]* *Agustín Galindo,*[c]* *and Claudio Pettinari,*[b]*

[a] Instituto de Investigaciones Químicas (IIQ), Spanish National Research Council (CSIC), University of Sevilla (US), Avda. Américo Vespucio 49, Isla de la Cartuja, 41092 Sevilla, Spain

[b] Chemistry Interdisciplinary Project (ChIP), School of Pharmacy, University of Camerino, Via Madonna delle Carceri, 62032 Camerino MC, Italy

[c] Departamento de Química Inorgánica, University of Sevilla (US), Aptdo. 1203, 41071 Sevilla, Spain

[‡] M.A-S. and M.G. contributed equally to this work.

[§, ∥] Present Addresses are listed before References section in the main manuscript.

Table of Contents

1 Experimental Procedures (Supporting Information) *S2*

1.1 General Information **S2**

1.2 Synthetic Procedures: Mono- and Bis-Quaternary Ammonium Salts [1] **S3**

1.3 Synthetic Procedures: Mixture of Bis(indazolyl)methane Isomers; Isolation and Characterization **S7**

1.4 Regioselective acetylation of indazole **S11**

1.5 Regioselective Syntheses of BINDM isomers on Demand **S15**

2 Crystallographic Data *S16*

3 NMR Spectra *S42*

3.1 NMR spectra of di(1H-indazol-1-yl)methane (*L1*) **S42**

3.2 NMR spectra of di(2H-indazol-2-yl)methane (*L2*) **S45**

3.3 NMR spectra of (1H-indazol-1-yl)(2H-indazol-2-yl)methane (*L3*) **S48**

3.4 NMR spectra of Indazole acetate isomers **S51**

3.5 NMR spectra of mono- and bis quaternary ammonium salts **S57**

4 Theoretical Study *S77*

4.1 Index of figures and schemes corresponding to the mechanism described in the main manuscript: **S77**

4.2 Table S14. Coordinates of the optimized compounds. **S83**

5 References (SI) *S127*

1 Experimental Procedures (Supporting Information)

This document provides complete experimental procedures and full characterization data; a concise Experimental Section is included in the main manuscript.

1.1 General Information

All procedures and manipulations were performed under a dry, oxygen-free nitrogen atmosphere using standard Schlenk or glovebox techniques, unless explicitly stated otherwise. Solvents were distilled under nitrogen using the following drying agents: sodium/benzophenone ketyl for diethyl ether (Et_2O) and tetrahydrofuran (THF); sodium for pentane and toluene; and calcium hydride (CaH_2) for dichloromethane (CH_2Cl_2) and acetonitrile (CH_3CN). All solvents were degassed prior to use.

Solution nuclear magnetic resonance (NMR) spectra were recorded using Bruker DRX-400 (400 MHz), AVANCE III/ASCEND 400R (400 MHz), and AVANCE III (500 MHz) instruments at the IIQ. ^1H and ^{13}C chemical shifts were referenced to residual signals of the deuterated solvents, with all data reported

in parts per million (ppm) downfield from tetramethylsilane (Me_4Si). Coupling constants (J values) are given in Hertz (Hz). The following abbreviations are used to designate multiplicities: **s** = singlet, **d** = doublet, **t** = triplet, **q** = quartet, **quin** = quintuplet, **sext** = sextet, **sep** = septet, **m** = multiplet, **br** = broad, **dd** = double-doublet, ddd = double-double-doublet.

Fourier-transform infrared spectra (FTIR) were recorded using a Bruker Tensor 27 spectrometer (IIQ). The following abbreviations denote the relative transmittance intensity of the observed peaks, with the vertical axis representing transmittance and the horizontal axis showing frequencies as wavenumbers (cm^{-1}): **vs** = very strong, **s** = strong, **m** = medium, and **w** = weak. As the novel compounds described in this manuscript do not contain functional groups (such as alcohols or acids) that typically produce broad FTIR peaks, all peak widths mentioned below refer to those considered sharp.

Mass spectrometry analysis (MS) with an electrospray ionization (ESI) source, as well as elemental analyses, were carry out by the Instrumentation Services at IIQ (Mass Spectrometry and Analytical Services) using a Bruker Esquire 6000 Ion Trap Mass Spectrometer or a Bruker AmaZon SL Ion Trap LC/MS instrument, and a LECO True-Spec CHNS elemental analyzer, respectively.

Single-crystal X-ray diffraction data were collected using a Bruker-Nonius X8 Apex-II diffractometer (IIQ) or a Bruker-AXS D8 QUEST ECO diffractometer (IIQ).

1.2 Synthetic Procedures: Mono- and Bis-Quaternary Ammonium Salts[1]

1.2.1 Synthesis of 1,1'-methylenedi(pyridin-1-ium) dichloride (1-Cl)[2–4]

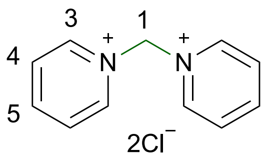

A solution of pyridine (15 mL, 100 mmol) in toluene (20 mL) and CH_2Cl_2 (3.2 mL, 50 mmol) was placed in a sealed Fisher-Porter tube under an argon atmosphere and stirred for 18 hours at 120 °C. After this period, the resulting solid was filtered, washed with hexane (4 × 10 mL), and dried under vacuum, affording a white solid in 73% yield.

Mp: 232 °C.

Elem. Anal. Calcd. (%) for $C_{11}H_{12}Cl_2N_2$: C, 54.34; H, 4.98; N, 11.52; **Found**: C, 56.27; H, 5.12; N, 10.94.

FTIR (KBr, cm^{-1}): 3136 (w), 3096 (w), 3031 (s), 3004 (s), 2875 (m), 2098 (w), 1634 (vs), 1583 (w), 1497 (vs), 1361 (w), 1301 (m), 1242 (w), 1227 (w), 1185 (vs), 1022 (w), 845 (w), 834 (m), 834 (m), 785 (s), 772 (s), 696 (s), 678 (m), 514 (s).

^{1}H NMR (500 MHz, MeOD): δ = 9.57 (d, 6.0 Hz, 4H, H3), 8.88 (t, 7.9 Hz, 2H, H5), 8.36 (t, 7.8 Hz, 4H, H4), 7.53 (s, 2H, H1).

^{13}C NMR (125 MHz, MeOD): δ = 149.63 (C5), 145.99 (C3), 129.20 (C4), 77.70 (C1).

MS (ESI+ m/z): 86 $[M/2]^{2+}$.

1.2.2 Synthesis of 1,1'-methylenedi(pyridin-1-ium) dibromide (1-Br)[5,6]

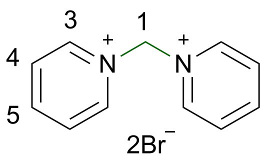

A solution of the corresponding pyridine (15 mL, 100 mmol) and CH_2Br_2 (3.5 mL, 50 mmol) was stirred for 18 hours at room temperature. After this period, the resulting solid was filtered, washed with hexane (4 × 10 mL), and dried under vacuum, yielding a white solid in 69% yield.

Mp: 254 °C.

Elem. Anal. Calcd. (%) for $C_{11}H_{12}Br_2N_2$: C, 39.79; H, 3.64; N, 8.44. **Found**: C, 39.66; H, 3.93; N, 8.41.

FTIR (KBr, cm^{-1}): 3132 (w), 3090 (w), 3069 (w), 3029 (s), 3006 (s), 2873 (m), 2044 (w), 1632 (vs), 1581 (w), 1495 (vs), 1440 (w), 1364 (w), 1299 (m), 1237 (w), 1223 (w), 1184 (vs), 1022 (m), 970 (w), 843 (w), 832 (m), 832 (m), 782 (s), 769 (s), 693 (s), 675 (s), 560 (s).

^{1}H NMR (500 MHz, MeOD): δ = 9.54 (d, 5.8 Hz, 4H, H3), 8.88 (t, 7.8 Hz, 2H, H5), 8.36 (t, 7.2 Hz, 4H, H4), 7.51 (s, 2H, H1).

^{13}C NMR (125 MHz, MeOD): δ = 149.43 (C5), 145.58 (C3), 129.13 (C4), 77.56 (C1).

MS (ESI+, m/z): 86 $[M/2]^{2+}$.

1.2.3 Synthesis of 1,1'-methylenedi(pyridin-1-ium) diiodide (1-I)[7,8]

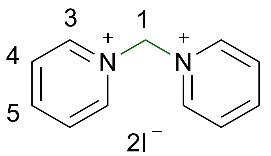

A solution of the corresponding pyridine (15 mL, 100 mmol) and CH_2I_2 (4 mL, 50 mmol) was stirred for 18 hours at room temperature. After this period, the resulting yellow solid was filtered, washed with hexane (4 × 10 mL), and dried under vacuum, affording the product in a 65 % yield.

M.p. 229 °C.

Elem. Anal. Calcd. (%) for $C_{11}H_{12}I_2N_2$: C, 31.01; H, 2.84; N, 6.58.

Found: C, 30.87; H, 3.30; N, 6.54.

FTIR (KBr, cm^{-1}): 3126 (w), 3077 (w), 3029 (s), 2865 (w), 1984 (w), 1624 (vs), 1579 (m), 1548 (w), 1491 (vs), 1434 (w), 1367 (w), 1294 (m), 1231 (w), 1180 (vs), 1021 (w), 962 (w), 840 (w), 830 (w), 777 (m), 766(s), 689 (s), 671 (m), 558 (m), 524 (m).

^{1}H NMR (500 MHz, MeOD): δ = 9.49 (d, 6.5 Hz, 4H, H3), 8.86 (t, 2H, H5), 8.36 (t, 7.5 Hz, 4H, H4), 7.46 (s, 2H, H1).

^{13}C NMR (125 MHz, $(CD_3)_2SO$): δ = 149.23 (C5), 146.21 (C3), 129.24 (C4), 77.08 (C1).

MS (ESI+, m/z): 86 $[M/2]^{2+}$

1.2.4 Synthesis of 1,1'-methylenedi(3-methylpyridin-1-ium) dichloride (2-Cl)

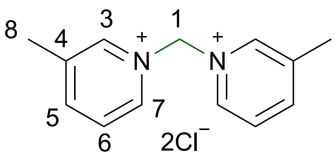

A solution of 3-picoline (1.98 mL, 20 mmol) in acetone (10 mL) and CH_2Cl_2 (0.65 mL, 10 mmol) was added to a sealed Fisher-Porter tube under an argon atmosphere. The solution was then heated to 100 °C for 2 days. After this period, the resulting white solid was filtered, washed with hexane (4 × 10 mL), and dried under vacuum, yielding the product in a 58% yield.

Mp: 248 °C.

Elem. Anal. Calcd. (%) for $C_{13}H_{16}Cl_2N_2$: C, 57.58; H, 5.95; N, 10.33. **Found**: C, 57.40; H, 6.05; N, 10.32.

FTIR (KBr, cm^{-1}): 3021 (s), 2935 (s), 2856 (w), 2066 (w), 1811 (w), 1627 (vs), 1589 (w), 1506 (vs), 1441 (m), 1376 (w), 1305 (s), 1252 (s), 1205 (s), 1165 (s), 1045 (w), 1025 (m), 937 (s), 921 (m), 839 (m), 824 (m), 768 (s), 752 (s), 702 (s), 676 (s), 634 (w).

^{1}**H NMR** (500 MHz, MeOD): δ = 9.42 (s, 2H, H3), 9.35 (d, 6.0 Hz, 2H, H7), 8.67 (d, 7.9 Hz, 2H, H6), 8.19 (t, 7.0 Hz, 2H, H5), 7.40 (s, 2H, H1), 2.65 (s, 6H, H8).

^{13}**C NMR** (125 MHz, MeOD): δ = 151.04 (C5), 146.41 (C3), 144.08 (C7), 142.59 (C4), 129.62 (C6), 78.68 (C1), 18.59 (C8).

MS (ESI+, m/z): 100 $[M/2]^{2+}$.

1.2.5 Synthesis of 1,1'-methylenedi(3-methylpyridin-1-ium) dibromide (2-Br)

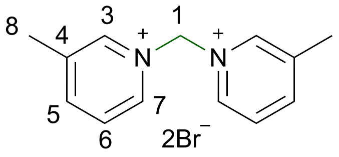

A solution of 3-picoline (1.98 mL, 20 mmol) in acetone (10 mL) and CH_2Br_2 (0.700 mL, 10 mmol) was added to a sealed Fisher-Porter tube under an argon atmosphere. The solution was heated to 120 °C for 18 h. After this period, the resulting solid was filtered, washed with hexane (4 × 10 mL), and dried under vacuum, affording a white solid in a 60% yield.

M.p. 262 °C.

Elem. Anal. Calcd. (%) for $C_{13}H_{16}Br_2N_2$: C, 43.36; H, 4.48; N, 7.78. **Found:** C, 43.44; H, 4.53; N, 7.72.

FTIR (KBr, cm^{-1}): 3022 (s), 2934 (s), 2056 (w), 1813 (w), 1628 (vs), 1589 (m), 1503 (vs), 1474 (m), 1441 (m), 1378 (m), 1350 (w), 1327 (w), 1305 (s), 1252 (s), 1222 (m), 1206 (m), 1171 (s), 1157 (s), 1138 (w), 1047 (w), 1019 (w), 932 (m), 840 (w), 824 (w), 810 (w), 766 (s), 752 (m), 701 (s), 671 (s), 631 (w).

^{1}**H NMR** (500 MHz, MeOD): δ = 9.36 (s, 2H, H3), 9.29 (d, 5.6 Hz, 2H, H7), 8.67 (d, 8.0 Hz, 2H, H5), 8.19 (t, 6.6 Hz, 2H, H6), 7.34 (s, 2H, H1), 2.65 (s, 6H, H8).

^{13}**C NMR** (125 MHz, MeOD): δ = 151.06 (C5), 146.46 (C3), 144.06 (C7), 142.53 (C4), 129.63 (C6), 78.38 (C1), 18.72 (C8).

MS (ESI+ m/z): 100 $[M/2]^{2+}$

1.2.6 Synthesis of 1,1'-methylenedi(3-methylpyridin-1-ium) diiodide (2-I)

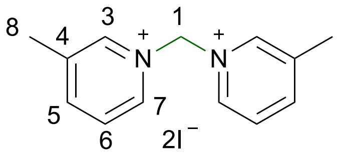

A solution of 3-picoline (1.98 mL, 20 mmol) in acetone (10mL) and CH_2I_2 (0.810 mL, 10 mmol) was added into to a sealed Fisher-Porter tube under an argon atmosphere. The solution was then heated to 120 °C for 18 hours. After this period, the resulting solid was filtered, washed with hexane (4 × 10 mL), and dried under vacuum. The product was obtained as a white solid with a 78% yield.

Mp: 240 °C.

Elem. Anal. Calcd. (%) for $C_{13}H_{16}I_2N_2$: C, 34.39; H, 3.55; N, 6.17. **Found:** C, 34.28; H, 3.50; N, 6.13.

FTIR (KBr, cm^{-1}): 3029 (vs), 2931 (s), 1984 (w), 1796 (w), 1627 (vs), 1587 (m), 1502 (vs), 1470 (m), 1437 (w), 1379 (m), 1347 (w), 1304 (s), 1252 (s), 1217 (m), 1203 (m), 1167 (vs), 1135 (vs), 1045 (w), 1019 (w), 1002 (w), 931 (m), 890 (w), 831 (m), 806 (w), 763 (vs), 752 (m), 697 (s), 669 (s), 630 (m).

^{1}**H NMR** (500 MHz, MeOD): δ = 9.38 (s, 2H, H3), 9.30 (d, 6.4 Hz, 2H, H7), 8.66 (d, 8.0 Hz, 2H, H5), 8.20 (t, 7.1 Hz, 2H, H6), 7.34 (s, 2H, H1), 2.66 (s, 6H, H8).

^{13}**C NMR** (125 MHz, MeOD): δ = 151.20 (C5), 146.41 (C3), 144.00 (C7), 142.68 (C4), 129.75 (C6), 78.66 (C1), 18.79 (C8).

MS (ESI+, m/z): 100 $[M/2]^{2+}$.

1.2.7 Synthesis of 1-(chloromethyl)-4-aza-1-azonia bicyclo[2.2.2]octane chloride (3-Cl)[9,10]

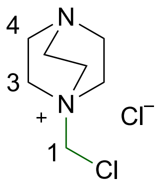

To a solution of DABCO (1.15 g, 10 mmol) in acetone (40 mL), CH_2Cl_2 (0.62 mL, 10 mmol) was added, and the mixture was stirred for 2 days at room temperature. After this period, the resulting solid was filtered and dried under an inert atmosphere under vacuum conditions, as the product is a deliquescent solid. Before complete drying, some crystals were collected and characterized by single-crystal X-ray diffraction (SC-XRD). The product was obtained as a white solid with a 67% yield.

Mp: 150-160 °C.

Elem. Anal. Calcd. (%) for $C_7H_{14}Cl_2N_2$: C, 42.66; H, 7.16; N, 14.21; **Found**: C, 42.22; H, 8.10; N, 14.07. **FTIR** (ATR, cm^{-1}): 3416 (s), 3375 (s), 3266 (m), 3007 (m), 1638 (m), 1461 (m), 1396 (w), 1362 (m), 1324 (w), 1093 (s), 1052 (s), 984 (m), 949 (w), 901 (m), 840 (s), 780 (s), 684 (m), 617 (vs), 565 (w), 533 (vs), 512 (s), 428 (m), 351 (w), 288 (w).

^{1}H NMR (400 MHz, MeOD): δ = 5.25 (s, 2H, H1), 3.54 (t, 7.2 Hz, 6H, H3), 3.28 (t, 7.2 Hz, 6H, H4).

^{13}C NMR (100 MHz, MeOD): δ = 69.27 (C1), 52.57 (C3), 45.87 (C4).

MS (ESI+, m/z): 161, 163 $[M – (Cl)]^+$.

1.2.8 Synthesis of 1-(bromomethyl)-4-aza-1-azonia bicyclo[2.2.2]octane bromide (3-Br)[11,12]

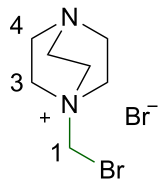

To a solution of the DABCO (1.15 g, 10 mmol) in acetone (40 mL), CH_2Br_2 (0.7 mL, 10 mmol) was added and the mixture was stirred for 2 days at room temperature. After this period, the resulting solid was filtered, washed with hexane (4 × 10 mL) and dried under vacuum conditions. The product was obtained as a white solid with a 71% yield.

Mp: 170-175 °C.

Elem. Anal. Calcd. (%) for $C_7H_{14}Br_2N_2$: C, 29.40; H, 4.93; N, 9.79; **Found**: C, 29.19; H, 5.60; N, 9.73.

FTIR (KBr, cm^{-1}): 2996 (s), 2301 (w), 1491 (s), 1464 (vs), 1437 (vs), 1350 (vs), 1199 (w), 1181 (w), 1094 (vs), 1055 (vs), 1003 (m), 987 (s), 902 (s), 868 (s), 839 (vs), 792 (s), 707 (s), 667 (s).

^{1}H NMR (500 MHz, MeOD): δ = 5.22 (s, 2H, H1), 3.54 (t, 7.2 Hz, 6H, H3), 3.27 (t, 7.2 Hz, 6H, H4).

^{13}C NMR (125 MHz, MeOD): δ = 57.58 (C1), 53.49 (C3), 46.06 (C4).

MS (ESI+, m/z): 205, 207 $[M – (Br)]^+$.

1.2.9 Synthesis of 1-(iodomethyl)-4-aza-1-azonia bicyclo[2.2.2]octane iodide (3-I)[13]

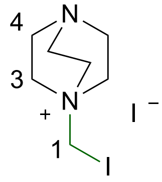

To a solution of the DABCO (1.15 g, 10 mmol) in acetone (40 mL), CH_2I_2 (0.8 mL, 10 mmol) was added, and the mixture was stirred for 2 days at room temperature. After this period, the resulting solid was filtered, washed with hexane (4 × 10 mL), and dried under vacuum conditions. The product was obtained as a white solid with an 83% yield.

Mp: 150-160 °C.

Elem. Anal. Calcd. (%) for $C_7H_{14}I_2N_2$: C, 22.12; H, 3.71; N, 7.37; **Found**: C, 22.01; H, 4.22; N, 7.33.

FTIR (KBr, cm^{-1}): 1456 (w), 1436 (s), 1424 (w), 1419 (w), 1368 (vs), 1255 (m), 1183 (w) , 1178 (w), 1088 (s), 1053 (s), 996 (w), 986 (s), 942 (w), 902 (m), 834 (s), 813 (m), 796 (m), 694 (s), 622 (m), 567 (w), 529 (s), 523 (m), 425 (w), 283 (s), 280 (s), 269 (s).

^{1}H NMR (500 MHz, MeOD): δ = 5.14 (s, 2H, H1), 3.47 (t, 7.4 Hz, 6H, H3), 3.24 (t, 7.4 Hz, 6H, H4).

^{13}C NMR (125 MHz, MeOD): δ = 54.95 (C3), 46.31 (C4), 31.34 (C1).

MS (ESI+, m/z): 253, 254 $[M – (I)]^+$.

1.3 Synthetic Procedures: Mixture of Bis(indazolyl)methane Isomers; Isolation and Characterization

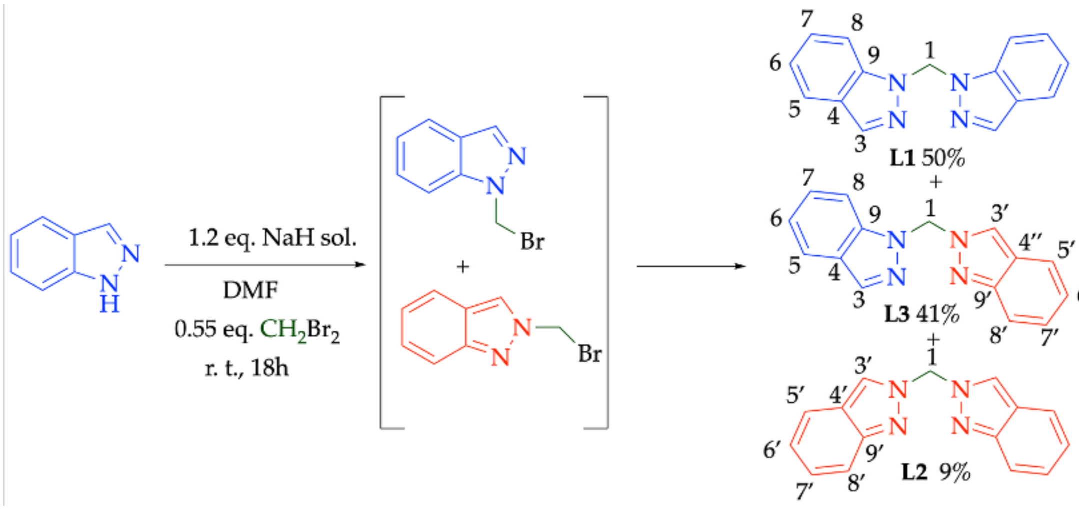

Scheme S1. One-pot synthesis of bis(indazolyl)methane isomers **L1**, **L2**, and **L3** using an ionizing base

In a 100 ml round-bottom flask, 606.3 mg (24 mmol) of 95% NaH (previously washed with dry pentane and stored in a glovebox) was added under an inert atmosphere and stirred magnetically with 10 mL of dry DMF. To this suspension, a solution of 2.4 g (20 mmol) of indazole in 30 mL of DMF was added, followed by the addition of 772 µL (11 mmol) of CH_2Br_2. The reaction mixture was stirred at room temperature for 18 hours. After this time, the mixture was hydrolyzed with 20 mL of a saturated NH_4Cl solution, diluted with 100 mL of distilled water, and extracted with diethyl ether (6 x 50 mL). The combined ether layers were washed with distilled water to remove any residual DMF, followed by a wash with brine. The solution was then dried over anhydrous Na_2SO_4, filtered, and concentrated under vacuum.

The isomeric ratio of bis(indazolyl)methane products (Scheme S1) was determined from an aliquot of the crude reaction mixture, concentrated under vacuum. This ratio was calculated by integration non-overlapping **^{1}H NMR** (500 MHz, CDCl3) signals specific to each isomer. The protons selected for analysis were H3 for **L1**, H3' for **L2**, and both H3 and H3' for **L3**, appearing as a broad singlet or a doublet due to long-range coupling with the aromatic protons H8 (**L1**), H8'(**L2**), and H8 and H8'(**L3**), respectively (Figure

S1). Based on integration of these signals, the isomer distribution of bis(indazolyl)methane isomers was found to be:

- δ 8.01 ppm, H3 (**L1**, 52%)
- δ 8.08 ppm, H3 and δ 8.06 ppm, H3' (**L3**, 41%)
- δ 8.27 ppm, H3’ (**L2**, 7%).

For reference, the isolated indazole’s H3 appears at δ: 8.14 ppm in the ^{1}**H NMR** spectrum. This isomer ratio can also be estimated by integrating the methylene bridge signal for each isomer, although this method is less precise due to signal overlap. The methylene proton signals appear at:

- δ: 6.90 ppm, 2H1 (**L1**)
- δ: 6.89 ppm, 2H1 (**L3**)

δ: 6.85 ppm, 2H1 (**L2**)

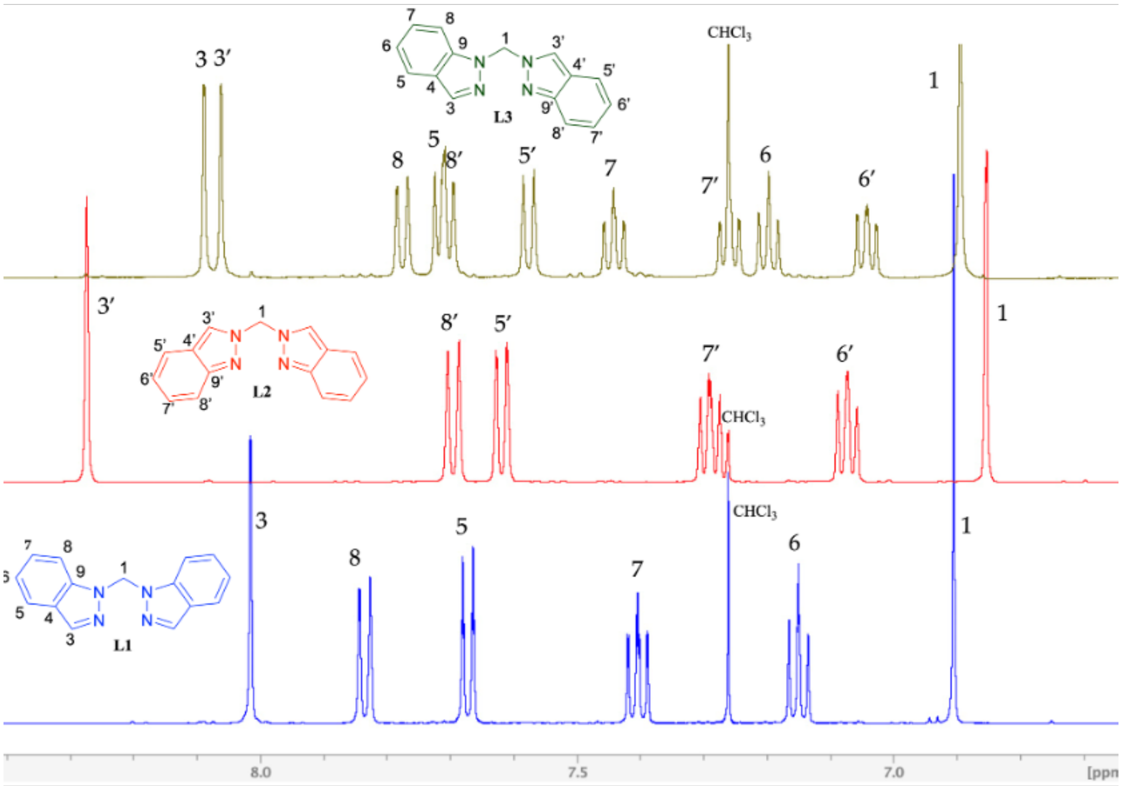

Figure S1. Comparative analysis of ^{1}H NMR (500 MHz, $CDCl_3$) spectra of di(1H-indazol-1-yl)methane (**L1**, blue spectrum), di(2H-indazol-2-yl)methane(**L2**, red spectrum) and (1H-indazol-1-yl)(2H-indazol-2-yl)methane (**L3**, green spectrum) isomers.

The crude compound, a whitish syrup, was purified by flash chromatography under nitrogen pressure (0.3-0.5 bar) on silica gel 60 (particle size 0.040-0.063 mm, ASTM mesh 230-400), using a gradient of diethyl ether in *n*-hexane with increasing polarity (1:10, 1:5, 1:3, and 1:1).

TLC analysis (Scharlau SiO2/Alu plates with a UV254 indicator; eluent: diethyl ether/*n*-hexane, 1:1) showed the following elution order: **L1** (R_f = 0.70), **L3** (with minor indazole contamination, R_f = 0.50), and finally **L2** (R_f = 0.30).

The chromatographic fraction containing **L3**, which was contaminated with unreacted indazole and difficult to purify, was acetylated using a slight excess of acetic anhydride and triethylamine, catalyzed by 5% DMAP[14] in dichloromethane at room temperature **(**Scheme S2**)**. Under these conditions, indazole produces a mixture of a kinetic isomer (**Ind-N2-Ac)**, and a thermodynamic isomer (**Ind-N1-Ac),** as observed by monitoring the reaction through aliquots analyzed by ^{1}H NMR. Over time, at room temperature, this isomer gradually converts into the thermodynamic form **Ind-N1-Ac** (see theoretical calculations below).

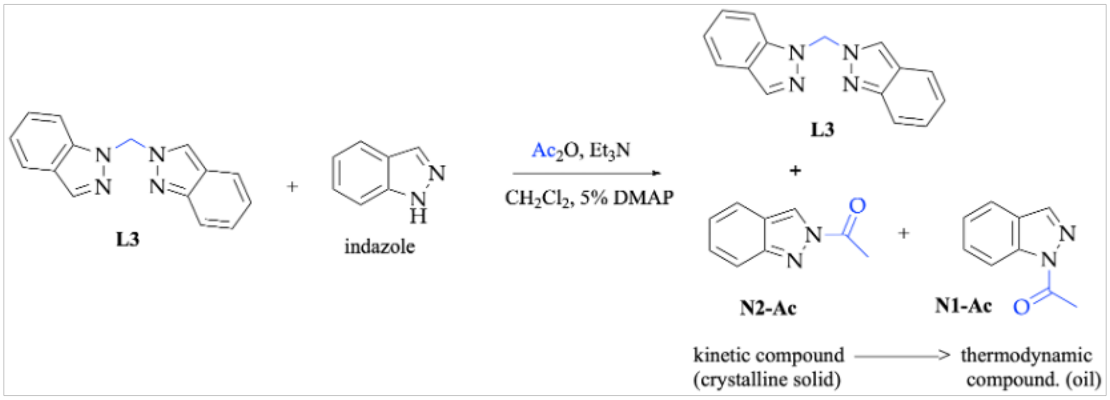

Scheme S2. The acetylation of indazole produces a mixture of acetates with a ratio of:
Ind-N2-Ac/Ind-N1-Ac = **2**:**3**

Flash chromatography of the **L3** mixture, containing indazole acetates, enabled the separation and purification of this bis(indazolyl)methane isomer. Indazole acetates, being less polar, eluted first using an diethyl ether/*n*-hexane (1:10) solvent system, followed by a 1:1 mixture to elute **L3**.

After concentrating the indazole acetate mixture in a rotary evaporator following chromatography, 0.36 g of the mixture was obtained, corresponding to 11,2% of the initial amount of indazole used. Avoiding heating or exposure to acids during the purification of the isomeric acetate, its spectroscopically characterization was achieved, revealing an **Ind-N2-Ac**/ **Ind-N1-Ac** ratio of **2:3**. To prevent isomerization of the bis(indazolyl)methane isomers, as well as indirectly of the indazole acetates (though this was not the primary objective), chromatography was always performed using basic silica, which was prepared by pretreatment with aqueous NaOH, followed by drying in an oven. The three isolated and purified bis(indazolyl)methane isomers were fully characterized by both spectroscopic techniques and single-crystal X-ray diffraction (SC-XRD).

1.3.1 Characterization data of di(1H-indazol-1-yl)methane (L1)

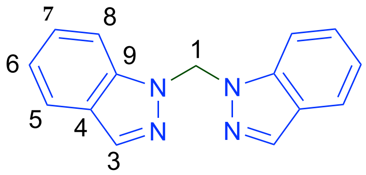

By collecting the initial fractions from chromatography, corresponding to the **L1** isomer as identified by TLC, 1,09 g of a white solid was obtained, yielding 43.9% based on the initial indazole used as the starting material

Mp: 150.2 °C.

Elem. Anal. Calcd. (%) for $C_{15}H_{12}N_4$: C, 72.56; H, 4.87; N, 22.57. **Found**: C, 72.13; H, 4.87; N, 22.55.

FTIR (KBr, cm^{-1}): 3061 (m), 1617 (w), 1499 (m), 1463 (m), 1439 (w), 1418 (w), 1361 (s), 1280 (m), 1205 (s), 1005 (w), 934 (m), 909 (m), 828 (m), 761 (m), 736 (s).

^{1}H NMR (500 MHz, $CDCl_3$): d = 8.01 (d, *J* = 0.9 Hz, 2H, H3), 7.83 (dtd, *J* = 8.4, 0.9, 0.7 Hz, 2H, H8), 7.67 (dt, *J* = 8.0, 0.9 Hz, 2H, H5), 7.40 (ddd, *J* = 8.4, 7.4, 0.9 Hz, 2H, H7) 7.15 (ddd, *J* = 8.0, 7.4, 0.7 Hz, 2H, H6) 6.90 (s, 2H, H1).

^{13}C NMR (125 MHz, $CDCl_3$): d = 139.62 (C9), 134.61 (C3), 127.20 (C7), 124.90 (C4), 121.60 (C6), 121.13 (C5), 110.18 (C8), 61.76 (C1).

MS (ESI +, m/z): $[M + Na]^+$: 271.1, $[2M + Na]^+$: 519.1.

1.3.2 Characterization data of di(2H-indazol-2-yl)methane (L2)

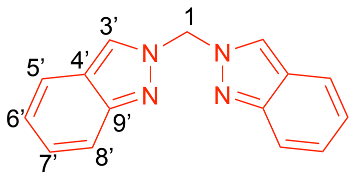

By collecting the final fractions from the chromatography, corresponding to the **L2** isomer identified by TLC, 140 mg of a white solid was obtained, yielding 5.2% based on the initial indazole used as the starting material.

Mp: 179 °C.

Elem. Anal. Calcd. (%) for $C_{15}H_{12}N_4$: C, 72.56; H, 4.87; N, 22.57. **Found:** C, 72.00; H, 4.90; N, 22.00.

FTIR (KBr, cm^{-1}): 2974 w), 2290 w), 1946 (m), 1919 (m), 1820 (s), 1794 (m), 1708 (vs), 1626 (w), 1561 (vs), 1514 (vs), 1470 (m), 1372 (m), 1326 (m), 1286 (m), 1240 (m), 1204 (m), 1134 (m), 1013 (m), 990 (m), 976 (m), 952 (m), 909(m).

^{1}H NMR (500 MHz, $CDCl_3$): d = 8.27 (dd, *J* = 0.3, 0.2 Hz, 2H, H3'), 7.69 (dtd, *J* = 8.8, 0.8, 0.1 Hz, 2H, H8'), 7.61 (ddt, *J* = 7.0, 0.8, 0.2 Hz, 2H, H5'), 7.29 (ddd, *J* = 8.8, 7.2, 0.8 Hz, 2H, H7') 7.07 (ddd, *J* = 7.2, 7.0, 0.8 Hz, 2H, H6'), 6.85 (s, 2H, H1).

^{13}C NMR (125 MHz, $CDCl_3$): d = 149.69 (C9'), 127.23 (C7'), 123.80 (C3'), 122.75 (C6'), 122.50 (C4'), 120.73 (C5'), 117.88 (C8'), 68.98 (C1).

MS (ESI+, m/z): 249.1 $[M + H^+]$, 271.1 $[M + Na^+]$, 288.3 $[M + K^+]$, 519.1 $[2M + Na^+]$.

1.3.3 Characterization data of (1H-indazol-1-yl)(2H-indazol-2-yl)methane (L3)

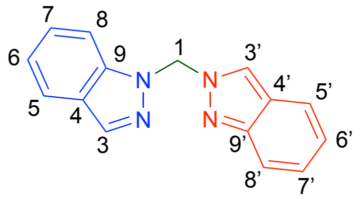

After chromatographing the **L3** mixture containing the indazole acetates, the final chromatographic fractions corresponding to the **L3** isomer, as identified by TLC, were collected. This yielded 0.8 g of a white solid, corresponding to a 37% yield based on the initial indazole used as the starting material.

Mp: 117 °C.

Elem. Anal. Calcd. (%) for $C_{15}H_{12}N_4$: C, 72.56; H, 4.87; N, 22.57. **Found**: C, 72.56; H, 4.91; N, 22.44.

FTIR (KBr, cm^{-1}): 3101 (m), 3058 (m), 2928 (s), 2852 (m), 1939 (w), 1629 (m), 1618 (m), 1517 (s), 1503 (s), 1468 (s), 1435 (s), 1412 (m), 1388 (m), 1364 (w), 1317 (m), 1284 (m), 1245 (w), 1231 (w), 1173 (m), 1151 (m), 1135 (s), 1115 (s), 1069 (m), 1006 (w), 958 (s), 941 (m), 910 (m), 872 (m), 847 (m), 831 (m), 782 (s), 771 (s), 763 (m), 746 (m), 710 (s).

^{1}**H NMR** (500 MHz, $CDCl_3$): d = 8.08 (d, J = 0.8 Hz, 1H, H3), 8.06 (d, J = 0.7 Hz, 1H, H3′), 7.77 (dq, J = 8.5, 0.8 Hz, 1H, H8), 7.71 (dt, J = 6.3, 0.8 Hz, 1H, H5), 7.70 (dq, J = 7.5, 0.7 Hz, 1H, H8’), 7.57 (dt, J = 7.5, 0.7 Hz, 1H, H5′), 7.44 (ddd, J = 8.5, 7.5, 0.8 Hz, 1H, H7), 7.25 (ddd, J = 9.0, 7.5, 0.7 Hz, 1H, H7’), 7.19 (ddd, J = 7.5, 6.3, 0.8 Hz, 1H, H6), 7.04 (ddd, J = 9.0, 7.5, 0.7 Hz, 1H, 6’), 6.89 (s, 2H, H1).

^{13}**C NMR** (125 MHz, $CDCl_3$): d = 149.05 (C9′), 139.86 (C9), 135.84 (C3), 127.66 (C7), 126.70 (C7′), 124.99 (C4), 122.60 (C3′), 122.52 (C4′), 122.42 (C6’), 122.01 (C6), 121.32 (C5), 120.57 (C5′), 117.99 (C8’), 109.84 (C8), 64.68 (C1).

MS (ESI+, m/z): 249.1 [M + H^+], 271.1 [M + Na^+].

1.4 Regioselective acetylation of indazole

Although our initial objective was not to selectively acetylate indazole, but rather to chromatographically separate (1H-indazol-1-yl)(2H-indazol-2-yl)methane (**L3**), which was contaminated with residual starting indazole that was difficult to separate, the unexpected results we observed sparked our curiosity. This was particularly intriguing as these findings had not been documented in the literature.[15,16] Consequently, we conducted additional tests, as shown in Table S1. The data reveal that acidic conditions or the presence of an amine with a low pK_aH value promote the formation of the thermodynamic **Ind-N1-Ac** indazole isomer. Conversely, the use of highly basic, sterically hindered amines (high pK_aH values) favors the formation of the kinetic **Ind-N2-Ac** indazole isomer. Additionally, even trace amounts of acid in a solution containing the acetylated isomers can induce complete isomerization of **Ind-N2-Ac** indazole to **Ind-N1-Ac** indazole at room temperature.

General procedure for the preparation of indazole acetates: In a flask equipped with magnetic stirring, 0.6 g (5 mmol) of indazole was dissolved in 10 mL of dichloromethane (CH_2Cl_2). To this solution, 1.38 mL (10 mmol) of triethylamine (or the corresponding amine, as indicated in Table S1) was added. It is worth noting that the use of DMPA as a catalyst — at least in the case of triethylamine — does not affect the ratio of indazole acetates formed. Subsequently, 0.75 mL (8 mmol) of acetic anhydride (Ac_2O) was added. Then, the reaction mixture was stirred at room temperature. The progress was monitored by checking an aliquot via ^{1}H NMR to confirm the absence of unreacted indazole (see Table S1 for the ratio of indazole acetates). Upon completion, the reaction was quenched with 10 mL of saturated ammonium chloride (NH_4Cl) solution, extracted with dichloromethane (3 × 10 mL), washed twice with brine, dried over anhydrous sodium sulphate (Na_2SO_4), and concentrated under reduced pressure using a rotary evaporator, ensuring the temperature did not exceed 50 °C.

The crude product was purified by flash chromatography on a short column of basic silica gel (pre-treated with sodium bicarbonate and oven-dried), using a 1:10 mixture of diethyl ether and n-hexane as the eluent. This afforded 0.75 g of the indazole acetate mixture (see Table S1), corresponding to a 93% yield.

For the comparison of regioselectivity among the indazole acetates listed in Table S1, triethylamine (Et_3N) was the only reagent replaced, being substituted with the corresponding amine.

Table S1. Regioselectivity assays of indazole acetylation using different conditions, mainly amines with different pK_aH.

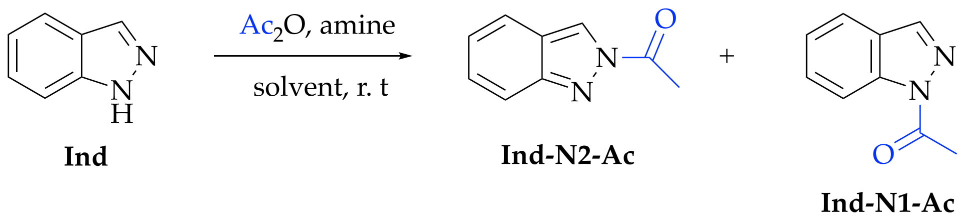

Entry	Ind (eq)	Ac_2O (eq)	Solvent	Amine (eq)	Time (h)	Ind-N2-Ac (%)	Ind-N1-Ac (%)
**1**	1	excess	-	-	4	-	100
**2**	1	1.2	CH_2Cl_2	Py (1.5)	12	20	80
**3**	1	1.2	CH_2Cl_2	Et_3N (1.5)	12	40	60
**4**	1	1.2	CH_2Cl_2	Cy_2NMe (1.5)	12	70	30
**5**	1	1.2	CH_2Cl_2	PMP (1.5)	12	80	20

The isomeric ratio of indazole acetylation products (**Ind-N2-Ac** and **Ind-N1-Ac**) was determined from an aliquot of the crude reaction mixture. This ratio was calculated by integration non-overlapping ^1H NMR (400 MHz, $CDCl_3$) signals specific to each isomer. The protons selected for analysis were H3 (s, 1H) and H8 (d, 1H) for **Ind-N1-Ac**, and H3' (s, 1H) and H6' (dd, 1H) for **Ind-N2-Ac** (Figure S2). For reference, the spectrum of indazole is also shown in Figure 2, which confirms that no unreacted compound remains after the acetylation reaction.

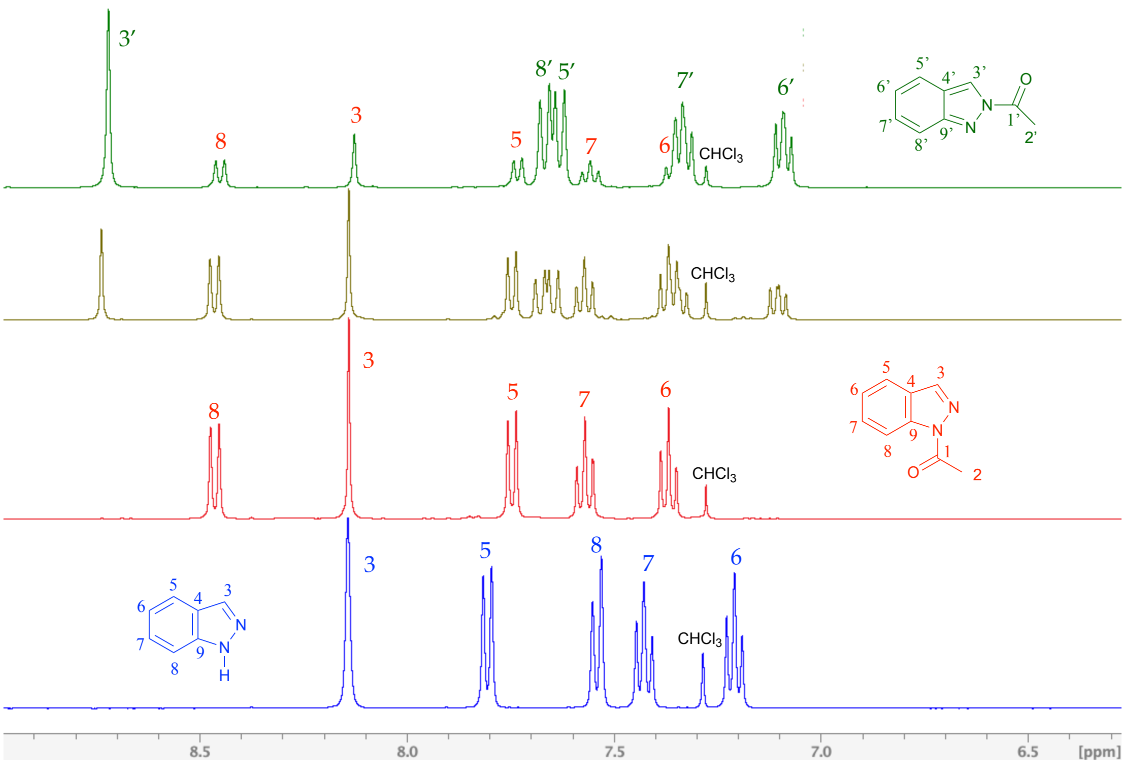

Figure S2. Comparative analysis of the **1H NMR** spectra (400 MHz, $CDCl_3$) of indazole (blue spectrum), **Ind-N1-Ac** (red spectrum), **Ind-N2-Ac/Ind- N1-Ac** ratio of 2:3 (Table S1, entry 3, brown spectrum), and **Ind-N2-Ac/Ind-N1-Ac** ratio of 4:1 (Table S1, entry 5, green spectrum). The N-H signals at 10.62 ppm (broad) for indazole, as well as the methyl signals at 2.79 ppm (s) for **Ind-N1-Ac** and 2.90 ppm (s) for **Ind-N2-Ac**, were omitted.

1.4.1 1-(2H-indazol-2-yl)ethan-1-one (Ind-N2-Ac)[15–17]

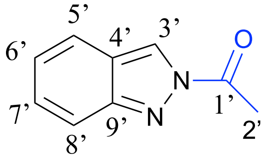

This indazole acetate **Ind-N2-Ac** isomer is highly unstable and readily isomerizes in solution to the thermodynamically favored **Ind-N1-Ac** isomer. Although it can be crystallized in pure form (colorless needles) through slow evaporation from a mixture of isomers in dichloromethane, with its crystal structure determined by SC-XRD (see below), it undergoes isomerization upon dissolution for spectroscopic analysis. Consequently, its spectroscopy was determined from the mixture of both isomers by subtracting the known signals of the **Ind-N1-Ac** isomer.

^{1}H NMR (400 MHz, $CDCl_3$): d 8.71 (s, 1H, H3’), 7.66 (d, J = 8.6 Hz, 1H, H8’), 7.62 (d, J = 8.9 Hz, 1H, H5’), 7.32 (dd, J = 8.6, 7.0 Hz, 1H, H7’), 7.08 (dd, J = 8.9, 7.0 Hz, 1H, H6’), 2.90 (s, 3H, H2’).

^{13}C NMR (100 MHz, $CDCl_3$): d 171.24 (C1’), 151.10 (C9’), 129.46 (C7’), 124.42 (C6’), 122.40 (C4’), 121.72 (C5’), 121.65 (C3’), 119.08 (C8’), 22.26 (C2’).

1.4.2 1-(1H-indazol-1-yl)ethan-1-one (Ind-N1-Ac)

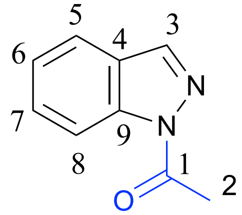

This compound was obtained through the complete isomerization of the mixture of both indazole acetate isomers. The solution was gently heated at 60 °C for 18 h in $CHCl_3$, and after concentration, the product appears as a colorless oil.

Elem. Anal. Calcd. (%) for $C_9H_9N_2O$: C, 67.48; H, 5.03; N, 17.49; **Found:** C, 66.65; H, 6.14; N, 17.26.

FTIR (ATR, cm^{-1}): 1707 (vs), 1427 (s), 1377 (vs), 1336 (vs), 1270 (w), 1194 (m), 1104 (w), 937 (vs), 888 (m), 762 (vs), 685 (m), 624 (s), 607 (m), 589 (w), 552 (w).

^{1}H NMR (400 MHz, $CDCl_3$): d 8.44 (dd, J = 8.4, 0.6 Hz, 1H, H8), 8.12 (s, 1H, H3), 7.72 (dt, J = 8.0, 0.8 Hz, 1H, H5), 7.55 (td, J = 8.4, 0.8 Hz, 1H, H7), 7.35 (ddd, J = 8.4, 8.0, 0.8 Hz, 1H, H6), 2.79 (s, 3H, H1).

^{13}C NMR (100 MHz, $CDCl_3$): d 171.25 (C1), 139.88 (C3), 139.15 (C9), 129.62 (C7), 126.42 (C4), 124.61 (C6), 120.99 (C5), 115.70 (C8), 23.20 (C2).

1.4.3 Theoretical Calculations of Indazole acetates

Given the tendency of **Ind-N2-Ac** indazole to readily isomerize to **Ind-N1-Ac** indazole, we performed theoretical calculations to confirm that **Ind-N1-Ac** indazole is indeed the thermodynamic isomer (Table S2).

From an energetic perspective, the **Ind-N1-Ac** isomer is the most stable (thermodynamic isomer). However, the **Ind-N2-Ac** isomer is only slightly destabilized, with relative ΔG energy difference of 7.5 kcal·mol^{-1}.

Table S2. Results of theoretical calculations of indazole acetates

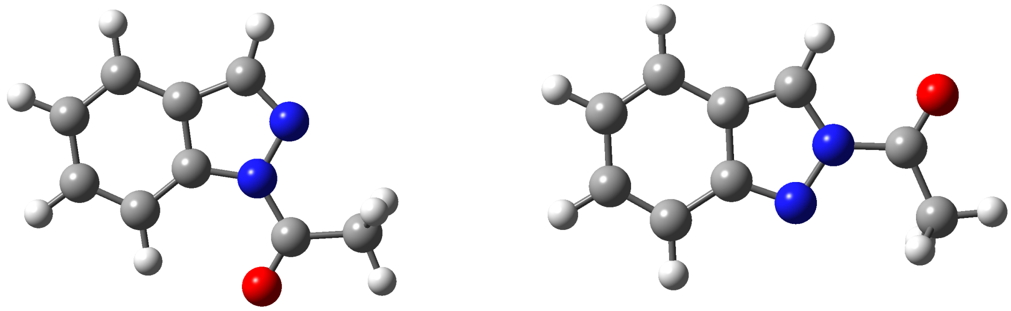

	**Ind-N1-Ac**	**Ind-N2-Ac**
Electronic energy	-532.645154550	-532.632705623
Zero-point Energies	-532.490434	-532.478400
Thermal Energies	-532.480881	-532.468794
Thermal Enthalpies	-532.479937	-532.467849
Thermal Free Energies	-532.525730	-532.513846

ΔE (relative, kcal/mol)	**0**	**7.8**
ΔG (relative, kcal/mol)	**0**	**7.5**

1.5 Regioselective Syntheses of BINDM isomers on Demand

1.5.1 General method used for the syntheses of bis(indazolyl)methane isomers as detailed in Table 1 of main manuscript

The experiments were conducted in a sealed Fischer-Porter vessel equipped with a pressure gauge and subjected to magnetic stirring. One equivalent of anhydrous indazole was dissolved in the appropriate volume of anhydrous, degassed solvent and introduced into the vessel at room temperature under a nitrogen atmosphere. To this solution, the required volume of the selected amine was added, followed by the appropriate equivalents of the methylenating agent. Once the vessel was sealed, the mixture was stirred at the specified temperature for the designated time, as detailed in Table 1.

The progress of the reaction was monitored by cooling the Fischer-Porter vessel to room temperature and sampling aliquots that were as representative as possible of the reaction mixture, which was often heterogeneous due to the formation of ammonium salts. After hydrolyzing the sample with distilled water, extracting with dichloromethane, and drying over anhydrous Na_2SO_4, the solvent was removed under reduced pressure using a rotary evaporator. The reaction progress was assessed through ^1H NMR analysis, which indicated the presence of unreacted indazole and the ratio of isomers of bis-(indazolyl)methane formed (See Figure S1 for comparative analysis of 1H NMR spectra).

1.5.2 Regioselective synthesis of di(1H-indazol-1-yl)methane (L1)

A solution of 2.36 g (20 mmol) of dry indazole in 80 mL of anhydrous toluene was prepared in a 250 mL Fischer–Porter flask under an inert atmosphere of nitrogen or argon, followed by the addition of 3.9 mL (48 mmol) of dry pyridine. After magnetic stirring for 5 minutes at room temperature, 5.6 mL (80 mmol) of CH_2Br_2 was added. The flask was then sealed, and the reaction mixture, maintained under an inert atmosphere with continuous stirring, was heated at 150 °C for 48 hours.

After cooling the Fischer–Porter vessel to room temperature, the reaction was quenched by the addition of 100 mL of distilled water and extracted with three 100 mL portions of ethyl acetate. The combined organic layers were washed with two 50 mL portions of brine, dried over anhydrous Na_2SO_4, filtered, and concentrated under reduced pressure using a rotary evaporator. The resulting residue was purified by flash chromatography using a 1:10 mixture of diethyl ether and n-hexane as eluent (see Section 1.3, page S9), affording 2.16 g of purified **L1** (87% yield).
For spectroscopic data, see section 1.3.1.

1.5.3 Regioselective synthesis of di(2H-indazol-2-yl)methane (L2)

A solution of 2.36 g (20 mmol) of dry indazole in 80 mL of anhydrous toluene was prepared in a 250 mL Fischer–Porter flask under an inert atmosphere of nitrogen or argon, followed by the addition of 8.7 mL (48 mmol) of dry PMP. After magnetic stirring for 5 minutes at room temperature, 11.22 mL (160 mmol) of CH_2Br_2 was added. The flask was then sealed, and the reaction mixture, maintained under the inert atmosphere with constant stirring, was heated at 150 °C for 48 hours.

After cooling the Fischer–Porter vessel to room temperature, the reaction was quenched by the addition of 100 mL of distilled water and extracted with three 100 mL portions of ethyl acetate. The combined organic layers were washed with two 50 mL portions of brine, dried over anhydrous Na_2SO_4,

filtered, and concentrated under reduced pressure using a rotary evaporator. The resulting residue was purified by flash chromatography, eluting with a 1:3 mixture of diethyl ether and *n*-hexane (see Section 1.3, page S10), affording 1.9 g of purified **L2** (77% yield).
For spectroscopic data see section 1.3.2.

1.5.4 Regioselective synthesis of (1H-indazol-1-yl)(2H-indazol-2-yl)methane (L3)

A total of 4.80 g (24 mmol) of $[(C_6H_7N)_2CH_2]Br_2$ (**2-Br**) was mixed with 70 mL of anhydrous tetrahydrofuran (THF), followed by the addition of 17.1 mL (80 mmol) of dry Cy_2NMe. The mixture was prepared in a 250 mL Fischer–Porter flask under an inert atmosphere of nitrogen or argon. After magnetic stirring for 5 minutes at room temperature, a solution of 2.36 g (20 mmol) of dry indazole in 10 mL of THF was added. The flask was then sealed, and the reaction mixture, under the inert atmosphere and constant stirring, was heated at 120 °C for 48 hours.

After cooling the Fischer–Porter vessel to room temperature, the reaction was quenched by the addition of 100 mL of distilled water and extracted with three 100 mL portions of ethyl acetate. The combined organic layers were washed with two 50 mL portions of brine, dried over anhydrous Na_2SO_4, filtered, and concentrated under reduced pressure using a rotary evaporator. The resulting residue was purified by flash chromatography, eluting with a 1:5 mixture of diethyl ether and *n*-hexane (see Section 1.3, page S10), affording 1.8 g of purified **L3** (73% yield).

For spectroscopic data, see Section 1.3.3.

2 Crystallographic Data

A summary of the crystallographic structure refinement data for the fourteen crystalline compounds discussed in this article is provided in Tables S3–S13 of this Supporting Information. Crystals of suitable size for X-ray diffraction analysis were coated with dry perfluoropolyether and mounted on glass fibers, then positioned on the goniometer head under a cold nitrogen stream (T = 193 K). Data were collected using either a Bruker-Nonius X8 Apex-II diffractometer equipped with a CCD area detector or a Bruker-AXS D8 QUEST ECO diffractometer equipped with a PHOTON II area detector. Monochromatic Mo K_α radiation (λ = 0.71073 Å) was employed, and data were acquired through ω and φ scans with a step width of 0.5°.

Data reduction was performed using the SAINT software, and absorption corrections were applied using the multi-scan method (SADABS), both integrated within Bruker's APEX5[18] crystallographic software suite. Structure solution was achieved using intrinsic phasing (SHELXT),[19] also included in the APEX5 package, and refinement was conducted against all F^2 data by full-matrix least-squares methods (SHELXL-2018/3),[20] minimizing $w[F_o^2\text{-}F_c^2]^2$, with the aid of the OLEX2-1.5[21] crystallographic software package. All non-hydrogen atoms were refined anisotropically, whereas hydrogen atoms were placed in calculated positions and refined using a riding model with isotropic displacement parameters.

2.1 X-Ray Crystallographic data of mono- and bis-quaternary ammonium salts used as methylenating agents

2.1.1 Single crystal X-ray structure of compound **1-Cl - CCDC 2431961**

Crystals of compound **1-Cl** were obtained by recrystallization from methanol, allowing the solvent to evaporate slowly.

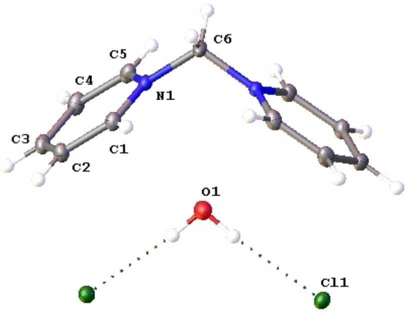

Figure S3. ORTEP representation of **1-Cl** with thermal ellipsoids shown at the 50% probability level.

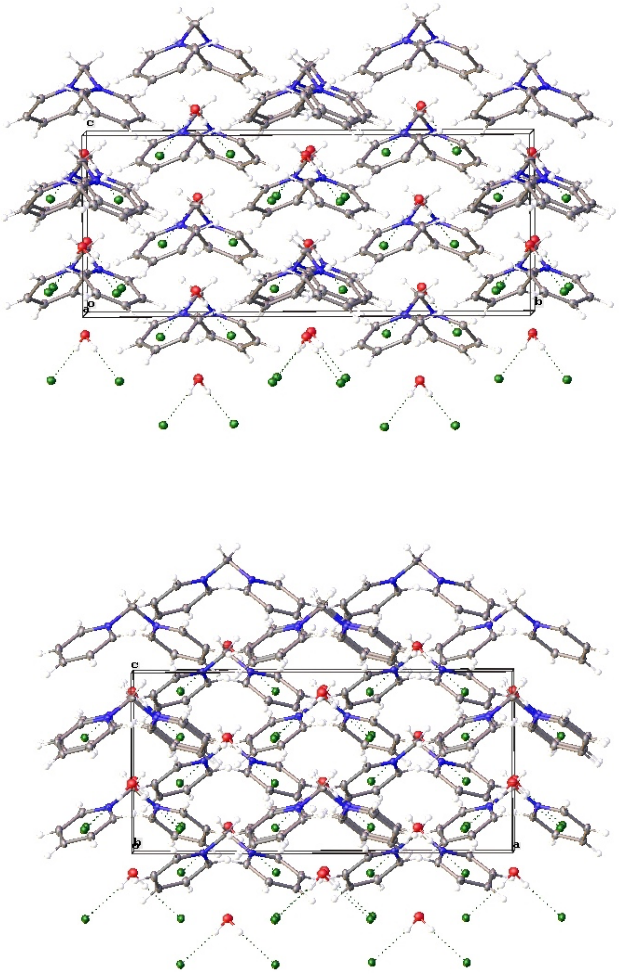

Figure S4. Crystal packing diagrams of **1-Cl** viewed along the *b*-axis (top) and the *c*-axis (bottom).

Table S3. Crystal data and structure refinement details for **1-Cl**

Empirical formula	$C_{11}H_{13}Cl_2N_2O$
Formula weight	260.13
Temperature/K	193.00
Crystal system	orthorhombic
Space group	Fdd2
a/Å	16.3262(5)
b/Å	19.1747(6)
c/Å	7.8092(2)
α/°	90
β/°	90
γ/°	90
Volume/Å^3	2444.67(12)
Z	8
ρ_{calc}g/cm^3	1.414
μ/mm^{-1}	0.511
F(000)	1080.0
Crystal size/mm^3	0.5 × 0.4 × 0.2
Radiation	MoKα (λ = 0.71073)
2Θ range for data collection/°	6.162 to 61.032
Index ranges	$-23 \leq h \leq 23$, $-27 \leq k \leq 27$, $-11 \leq l \leq 10$
Reflections collected	14812
Independent reflections	1777 [R_{int} = 0.0223, R_{sigma} = 0.0181]
Data/restraints/parameters	1777/1/78
Goodness-of-fit on F^2	1.119
Final R indexes [I>=2σ (I)]	R_1 = 0.0184, wR_2 = 0.0528
Final R indexes [all data]	R_1 = 0.0186, wR_2 = 0.0531
Largest diff. peak/hole / e Å^{-3}	0.20/-0.15
Flack parameter	-0.009(12)

2.1.2 Single crystal X-ray structure of two polymorphs of **1-Br**

Two crystalline polymorphs of ***1,1'-methylenedi(pyridin-1-ium) dibromide*** (**1-Br**) were isolated from different solvents and are hereafter referred to as **(a)** and **(b)**. Crystals of polymorph **(a)** were obtained by slow evaporation from acetone, whereas crystals of polymorph **(b)** were obtained by recrystallization from methanol under the same conditions. Both polymorphs are chemically identical but display distinct crystallographic symmetries. - **CCDC 2431962 (a)** and **CCDC 2431962 (b)**

(a)

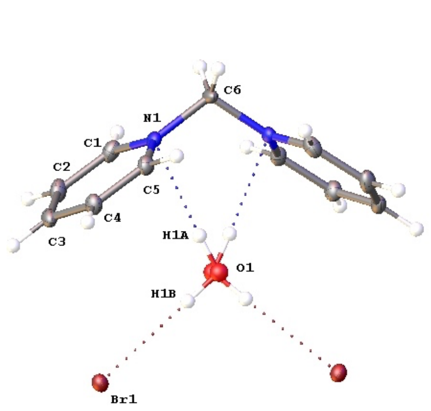

(b)

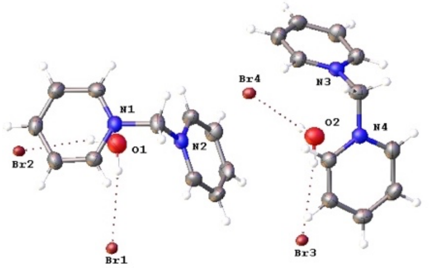

Figure S5. ORTEP representations of the two polymorphs of **1-Br** (forms **a** and **b**), both forms with thermal ellipsoids shown at the 50% probability level

(a)

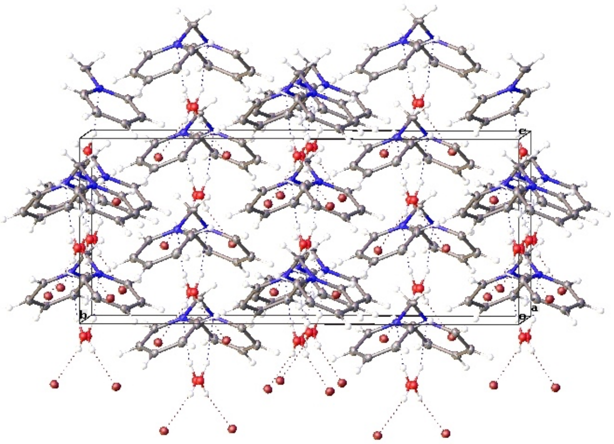

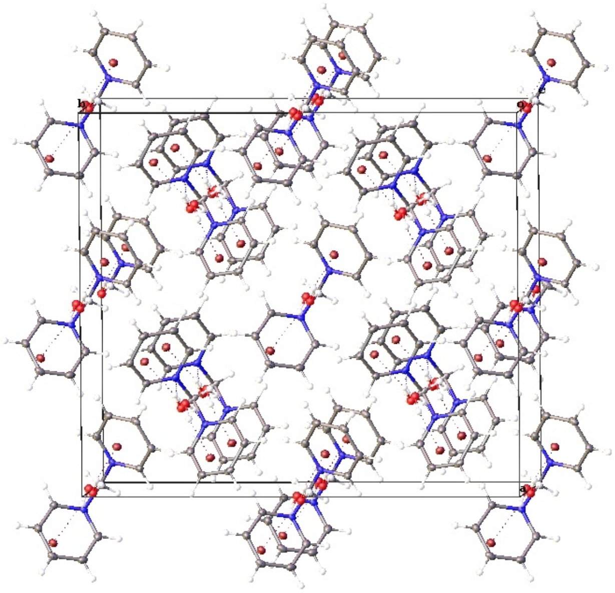

(b)

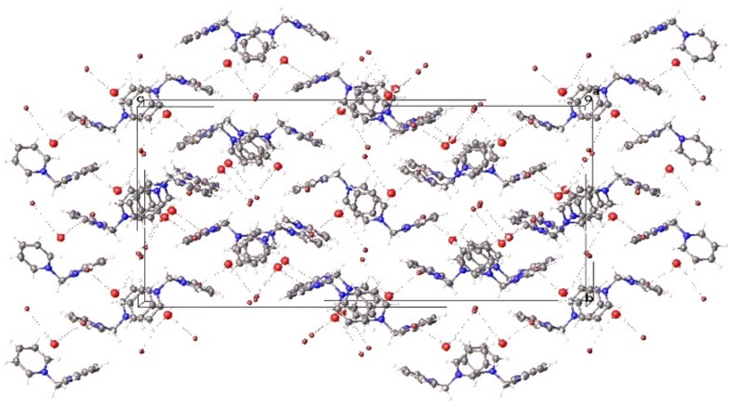

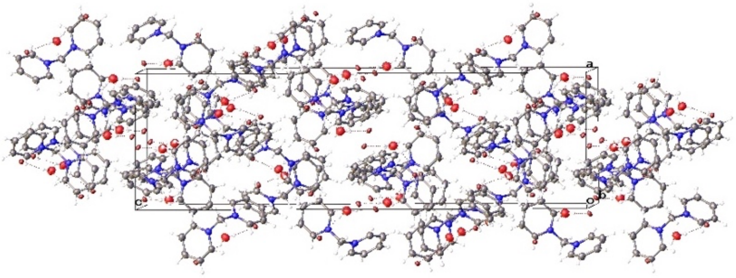

Figure S6. Crystal packing diagrams of the two polymorphs **a** and **b** of **1-Br**; (a) form **a**, viewed along the *a*-axis (top) and *c*-axis (bottom); (b) form **b**, viewed along the *a*-axis (top) and *b*-axis (bottom).

Table S4. Crystal data and structure refinement details for **1-Br** (two polymorphs: forms **a** and **b**)

	**(a)**	**(b)**
Empirical formula	$C_{11}H_{14}Br_2N_2O$	$C_{11}H_{14}Br_2N_2O$
Formula weight	350.06	350.06
Temperature/K	193.00	193.00
Crystal system	orthorhombic	orthorhombic
Space group	Fdd2	Pbca
a/Å	16.8080(6)	10.6055(4)
b/Å	19.2393(7)	15.7753(5)
c/Å	8.1200(3)	35.0228(10)
α/°	90	90
β/°	90	90
γ/°	90	90
Volume/Å^3	2625.80(17)	5859.5(3)
Z	8	16
ρ_{calc}g/cm^3	1.771	1.587
μ/mm^{-1}	6.156	5.518
F(000)	1376.0	2752.0
Crystal size/mm^3	0.4 × 0.35 × 0.25	0.3 × 0.1 × 0.083
Radiation	MoKα (λ = 0.71073)	MoKα (λ = 0.71073)
2Θ range for data collection/°	5.96 to 55.446	4.49 to 50.5
Index ranges	$-22 \leq h \leq 22$, $-24 \leq k \leq 25$, $-10 \leq l \leq 10$	$-12 \leq h \leq 12$, $-18 \leq k \leq 18$, $-42 \leq l \leq 41$
Reflections collected	14028	65994
Independent reflections	1527 [R_{int} = 0.0450, R_{sigma} = 0.0325]	5286 [R_{int} = 0.0227, R_{sigma} = 0.0129]
Data/restraints/parameters	1527/1/76	5286/0/290
Goodness-of-fit on F^2	1.084	1.096
Final R indexes [I>=2σ (I)]	R_1 = 0.0160, wR_2 = 0.0352	R_1 = 0.0362, wR_2 = 0.1199
Final R indexes [all data]	R_1 = 0.0181, wR_2 = 0.0358	R_1 = 0.0366, wR_2 = 0.1203
Largest diff. peak/hole / e Å^{-3}	0.23/-0.36	0.73/-0.82
Flack parameter	0.060(7)	

2.1.3 Single crystal X-ray structure of compound **1-I - CCDC 2431964**

Crystals of compound **1-I** were obtained by recrystallization from acetone, allowing the solvent to evaporate slowly.

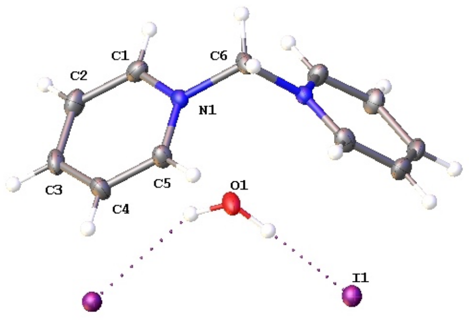

Figure S7. ORTEP representation of **1-I** with thermal ellipsoids shown at the 50% probability level.

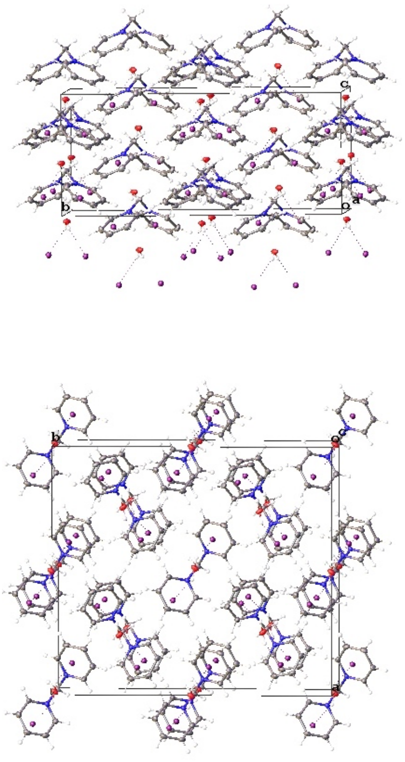

Figure S8. Crystal packing diagrams of **1-I** viewed along *a*-axis (top) and *c*-axis (bottom).

Table S5. Crystal data and structure refinement details for **1-I**

Empirical formula	$C_{22}H_{26}I_4N_4O$
Formula weight	870.07
Temperature/K	193.00
Crystal system	orthorhombic
Space group	Fdd2
a/Å	17.3549(5)
b/Å	19.4102(6)
c/Å	8.4713(2)
α/°	90
β/°	90
γ/°	90
Volume/Å^3	2853.66(14)
Z	4
ρ_{calc}g/cm^3	2.025
μ/mm^{-1}	4.387
F(000)	1624.0
Crystal size/mm^3	0.5 × 0.4 × 0.3
Radiation	MoKα (λ = 0.71073)
2Θ range for data collection/°	5.748 to 61.054
Index ranges	$-24 \leq h \leq 19$, $-27 \leq k \leq 27$, $-12 \leq l \leq 11$
Reflections collected	19070
Independent reflections	2112 [R_{int} = 0.0285, R_{sigma} = 0.0221]
Data/restraints/parameters	2112/7/75
Goodness-of-fit on F^2	1.199
Final R indexes [I>=2σ (I)]	R_1 = 0.0175, wR_2 = 0.0383
Final R indexes [all data]	R_1 = 0.0179, wR_2 = 0.0386
Largest diff. peak/hole / e Å^{-3}	0.49/-0.86
Flack parameter	0.97(4)

2.1.4 Single crystal X-ray structure of compound **2-Cl - CCDC 2431965**

Crystals of compound **2-Cl** were obtained by recrystallization from acetone, allowing the solvent to evaporate slowly.

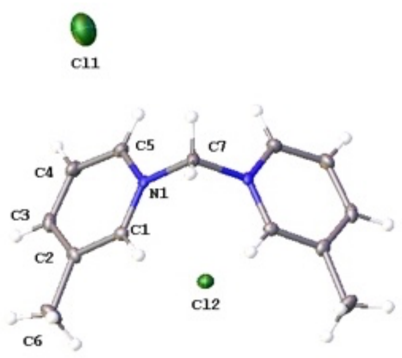

Figure S9. ORTEP representation of **2-Cl** with thermal ellipsoids shown at the 50% probability level.

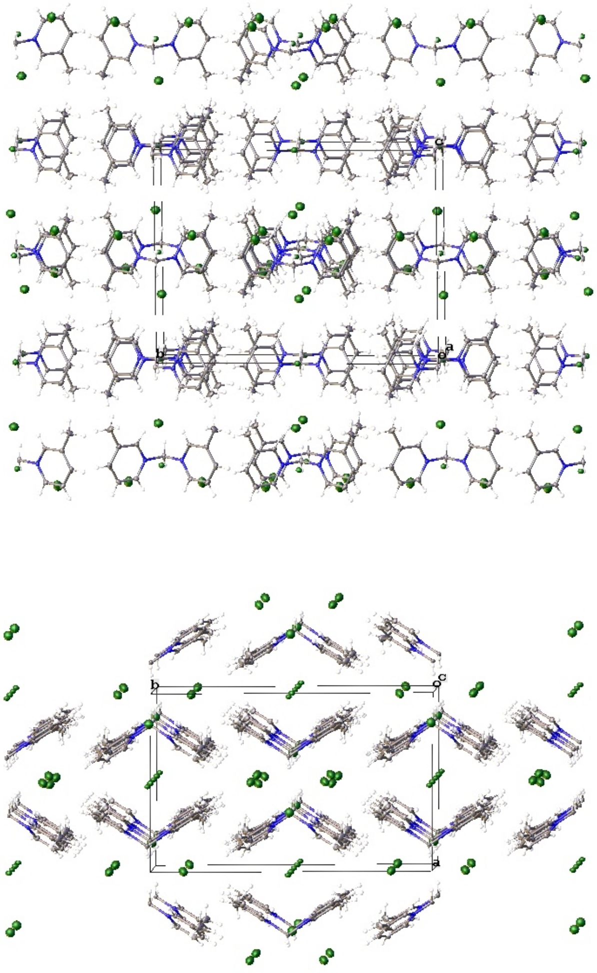

Figure S10. Crystal packing diagrams of **2-Cl**, viewed along *a*-axis (top) and *c*-axis (bottom).

Table S6. Crystal data and structure refinement details for **2-Cl**

Empirical formula	$C_{13}H_{16}Cl_2N_2$
Formula weight	271.18
Temperature/K	173.15
Crystal system	monoclinic
Space group	C2/m
a/Å	12.6408(3)
b/Å	18.5821(4)
c/Å	15.6052(3)
α/°	90
β/°	113.6040(10)
γ/°	90
Volume/Å^3	3358.87(13)
Z	8
ρ_{calc}g/cm^3	1.073
μ/mm^{-1}	0.370
F(000)	1136.0
Crystal size/mm^3	0.5 × 0.48 × 0.42
Radiation	MoKα (λ = 0.71073)
2Θ range for data collection/°	4.154 to 49.998
Index ranges	$-15 \leq h \leq 14$, $-17 \leq k \leq 22$, $-18 \leq l \leq 18$
Reflections collected	17151
Independent reflections	3037 [R_{int} = 0.0171, R_{sigma} = 0.0116]
Data/restraints/parameters	3037/0/164
Goodness-of-fit on F^2	1.074
Final R indexes [I>=2σ (I)]	R_1 = 0.0844, wR_2 = 0.2538
Final R indexes [all data]	R_1 = 0.0919, wR_2 = 0.2626
Largest diff. peak/hole / e Å^{-3}	0.93/-1.59

2.1.5 Single crystal X-ray structure of compound **2-Br - CCDC 2431966**

Crystals of compound **2-Br** were obtained by recrystallization from acetone, allowing the solvent to evaporate slowly.

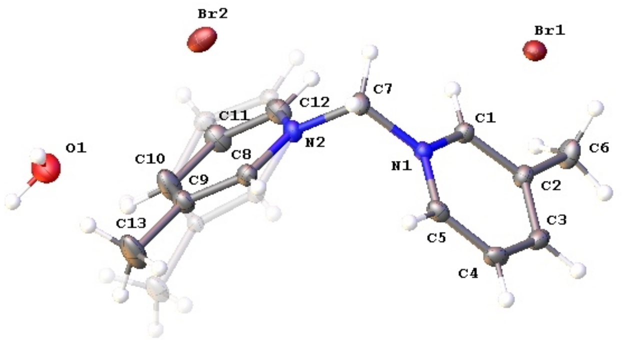

Figure S11. ORTEP representation of **2-Br** with thermal ellipsoids shown at the 50% probability level.

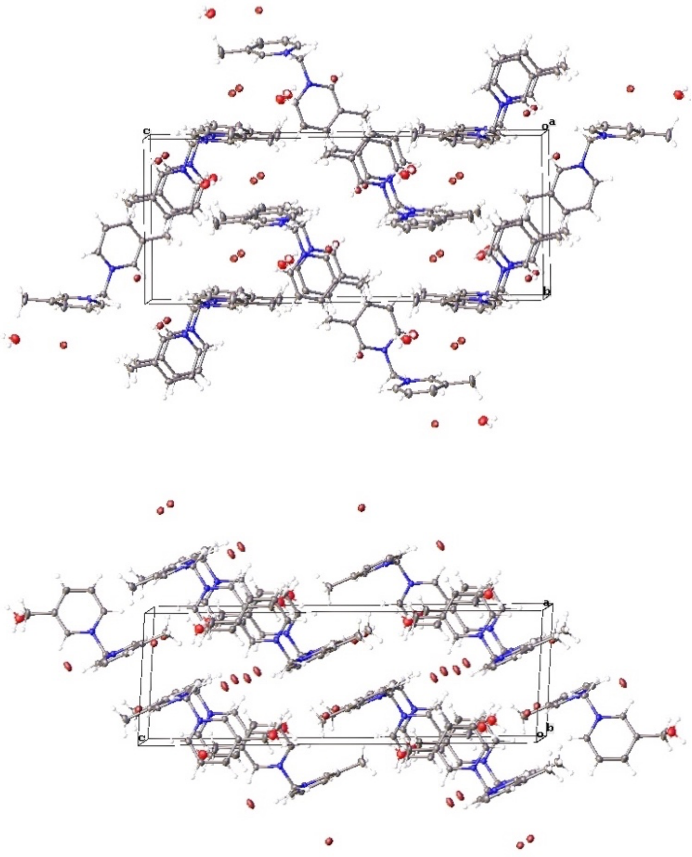

Figure S12. Crystal packing diagrams of **2-Br** viewed along *a* axis (top) and *b* axis (bottom).

Table S7. Crystal data and structure refinement details for **2-Br**

Empirical formula	$C_{13}H_{16.63}Br_2N_2O_{0.31}$
Formula weight	365.73
Temperature/K	173.15
Crystal system	monoclinic
Space group	$P2_1/c$
a/Å	7.3406(2)
b/Å	9.1286(2)
c/Å	22.0679(6)
α/°	90
β/°	93.4220(10)
γ/°	90
Volume/Å^3	1476.12(7)
Z	4
ρ_{calc}g/cm^3	1.646
μ/mm^{-1}	5.476
F(000)	725.0
Crystal size/mm^3	0.32 × 0.26 × 0.19
Radiation	MoKα (λ = 0.71073)
2Θ range for data collection/°	3.698 to 61.076
Index ranges	$-10 \leq h \leq 10$, $-13 \leq k \leq 12$, $-31 \leq l \leq 30$
Reflections collected	20840
Independent reflections	4472 [R_{int} = 0.0228, R_{sigma} = 0.0196]
Data/restraints/parameters	4472/103/216
Goodness-of-fit on F^2	1.012
Final R indexes [I>=2σ (I)]	R_1 = 0.0288, wR_2 = 0.0636
Final R indexes [all data]	R_1 = 0.0407, wR_2 = 0.0686
Largest diff. peak/hole / e Å^{-3}	1.17/-1.21

2.1.6 Single crystal X-ray structure of compound **2-I - CCDC 2431967**

Crystals of compound **2-I** were obtained by recrystallization from acetone, allowing the solvent to evaporate slowly.

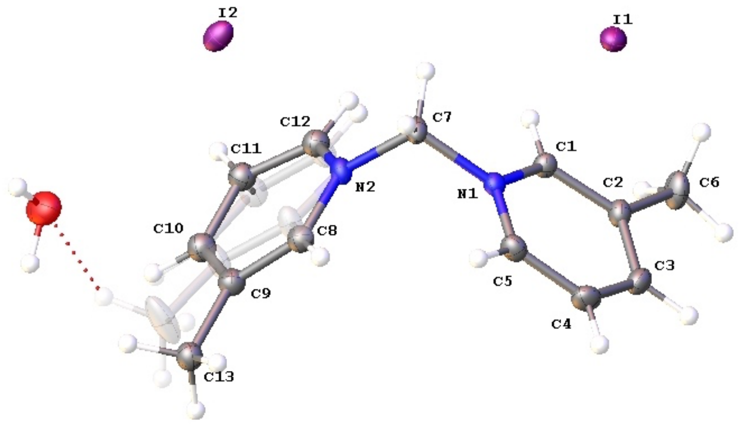

Figure S13. ORTEP representation of **2-I** with thermal ellipsoids shown at the 50% probability level.

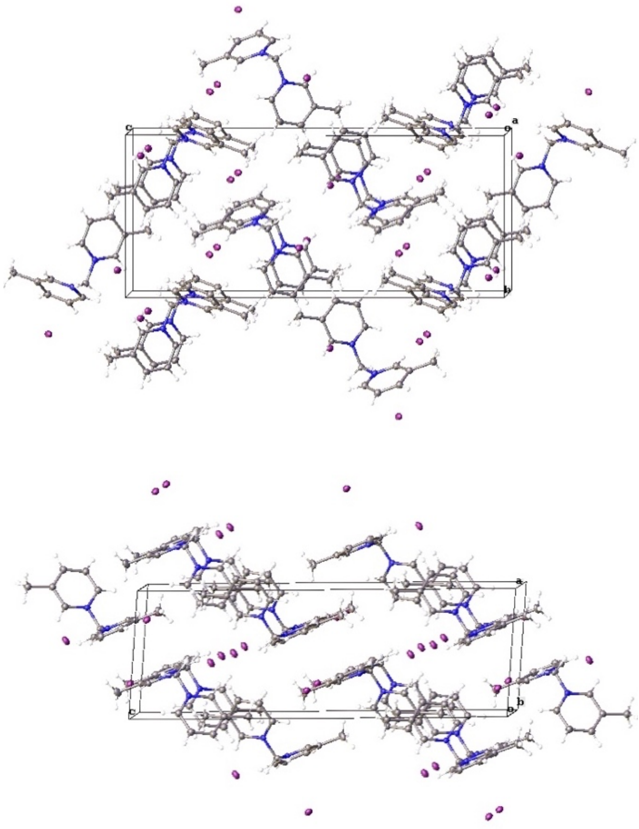

Figure S14. Crystal packing diagrams of **2-I** viewed along *a* axis (top) and *b* axis (bottom).

Table S8. Crystal data and structure refinement details for **2-I**

Empirical formula	$C_{13}H_{16.75}I_2N_2O_{0.37}$
Formula weight	460.79
Temperature/K	173.15
Crystal system	monoclinic
Space group	$P2_1/c$
a/Å	7.6064(3)
b/Å	9.6201(4)
c/Å	22.3880(9)
α/°	90
β/°	94.456(2)
γ/°	90
Volume/Å^3	1633.28(11)
Z	4
ρ_{calc}g/cm^3	1.874
μ/mm^{-1}	3.838
F(000)	871.0
Crystal size/mm^3	0.5 × 0.33 × 0.32
Radiation	MoKα (λ = 0.71073)
2Θ range for data collection/°	3.65 to 61.04
Index ranges	$-9 \leq h \leq 10$, $-13 \leq k \leq 12$, $-31 \leq l \leq 30$
Reflections collected	18112
Independent reflections	4874 [R_{int} = 0.0196, R_{sigma} = 0.0218]
Data/restraints/parameters	4874/109/216
Goodness-of-fit on F^2	1.048
Final R indexes [I>=2σ (I)]	R_1 = 0.0275, wR_2 = 0.0560
Final R indexes [all data]	R_1 = 0.0352, wR_2 = 0.0588
Largest diff. peak/hole / e Å^{-3}	0.83/-0.69

2.1.7 Single crystal X-ray structure of compound **3-Cl - CCDC 2431968**

Crystals of compound **3-Cl** were obtained by recrystallization from acetone, allowing the solvent to evaporate slowly.

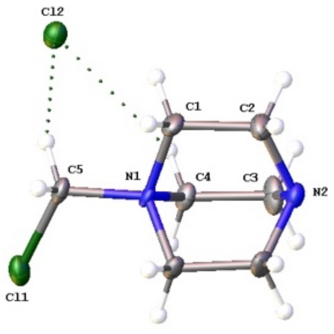

Figure S15. ORTEP representation of **3-Cl** with thermal ellipsoids shown at the 50% probability level.

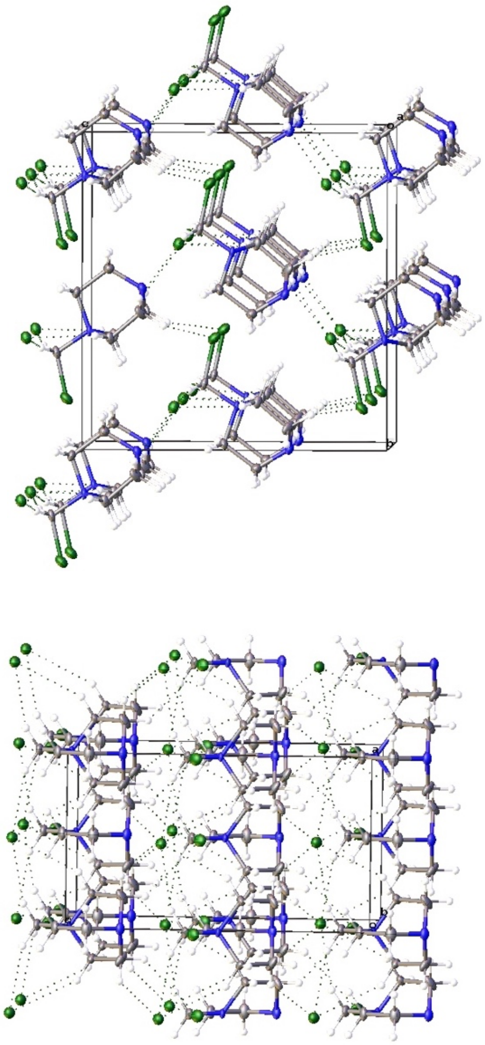

Figure S16. Crystal packing diagrams of **3-Cl** viewed along *a* axis (top) and *b* axis (bottom).

Table S9. Crystal data and structure refinement details for **3-Cl**

Empirical formula	$C_7H_{14}Cl_2N_2$
Formula weight	197.10
Temperature/K	193.00
Crystal system	orthorhombic
Space group	$Cmc2_1$
a/Å	6.6409(6)
b/Å	12.0168(15)
c/Å	11.5351(11)
α/°	90
β/°	90
γ/°	90
Volume/Å^3	920.53(17)
Z	4
ρcalcg/cm3	1.422
μ/mm-1	0.645
F(000)	416.0
Crystal size/mm3	0.3 × 0.2 × 0.1
Radiation	MoKα (λ = 0.71073)
2Θ range for data collection/°	6.782 to 50.388
Index ranges	-7 ≤ h ≤ 7, -14 ≤ k ≤ 14, -13 ≤ l ≤ 13
Reflections collected	8958
Independent reflections	894 [Rint = 0.0582, Rsigma = 0.0388]
Data/restraints/parameters	894/1/62
Goodness-of-fit on F2	1.134
Final R indexes [I>=2σ (I)]	R1 = 0.0366, wR2 = 0.0892
Final R indexes [all data]	R1 = 0.0368, wR2 = 0.0893
Largest diff. peak/hole / e Å-3	0.30/-0.25
Flack parameter	0.16(16)

2.1.8 Single crystal X-ray structure of compound **3-Br**[11] **- CCDC 2431969**

Crystals of compound **3-Br** were obtained by recrystallization from acetone, allowing the solvent to evaporate slowly.

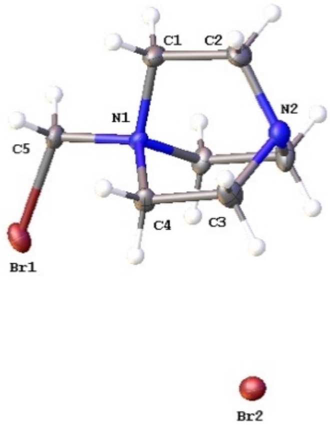

Figure S17. ORTEP representation of **3-Br** with thermal ellipsoids shown at the 50% probability level.

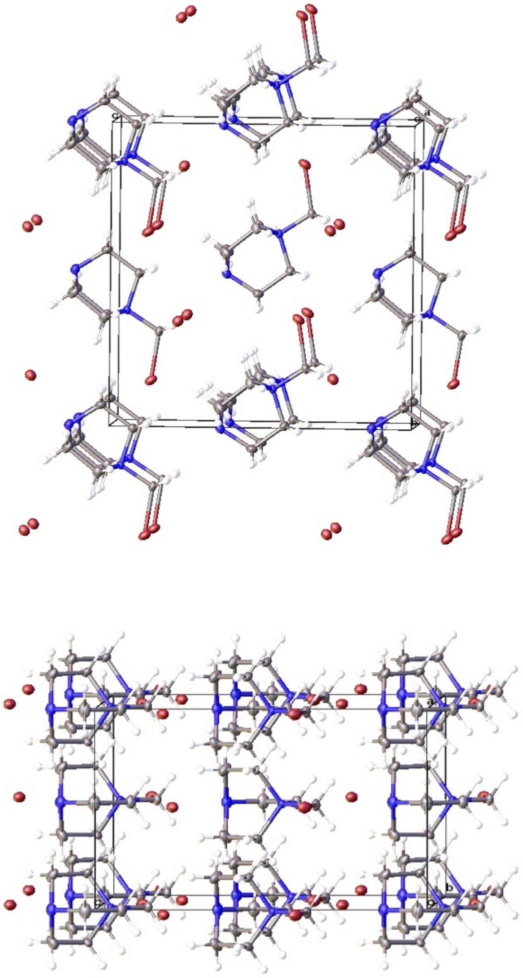

Figure S18. Crystal packing diagrams of **3-Br** viewed along *a* axis (top) and *b* axis (bottom).

Table S10. Crystal data and structure refinement details for **3-Br**

Empirical formula	$C_7H_{14}Br_2N_2$
Formula weight	286.02
Temperature/K	193.00
Crystal system	orthorhombic
Space group	$Cmc2_1$
a/Å	7.1082(3)
b/Å	11.8041(4)
c/Å	11.7651(4)
α/°	90
β/°	90
γ/°	90
Volume/Å^3	987.16(6)
Z	4
ρ_{calc}g/cm^3	1.925
μ/mm^{-1}	8.156
F(000)	560.0
Crystal size/mm^3	0.4 × 0.2 × 0.1
Radiation	MoKα (λ = 0.71073)
2Θ range for data collection/°	6.692 to 50.486
Index ranges	$-8 \leq h \leq 8$, $-14 \leq k \leq 14$, $-14 \leq l \leq 14$
Reflections collected	10321
Independent reflections	958 [R_{int} = 0.0564, R_{sigma} = 0.0429]
Data/restraints/parameters	958/1/61
Goodness-of-fit on F^2	1.294
Final R indexes [I>=2σ (I)]	R_1 = 0.0311, wR_2 = 0.0580
Final R indexes [all data]	R_1 = 0.0314, wR_2 = 0.0584
Largest diff. peak/hole / e Å^{-3}	0.77/-0.74
Flack parameter	0.092(11)

2.1.9 Single crystal X-ray structure of compound **3-I - CCDC 2431970**

Crystals of compound **3-I** were obtained by recrystallization from acetone, allowing the solvent to evaporate slowly.

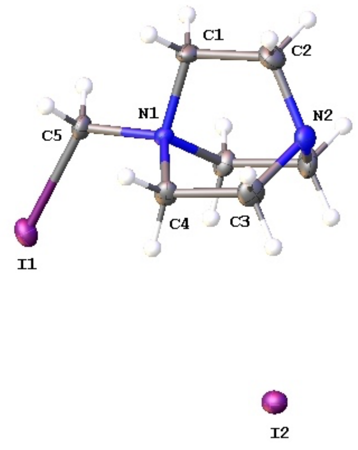

Figure S19. ORTEP representation of **3-I** with thermal ellipsoids shown at the 50% probability level.

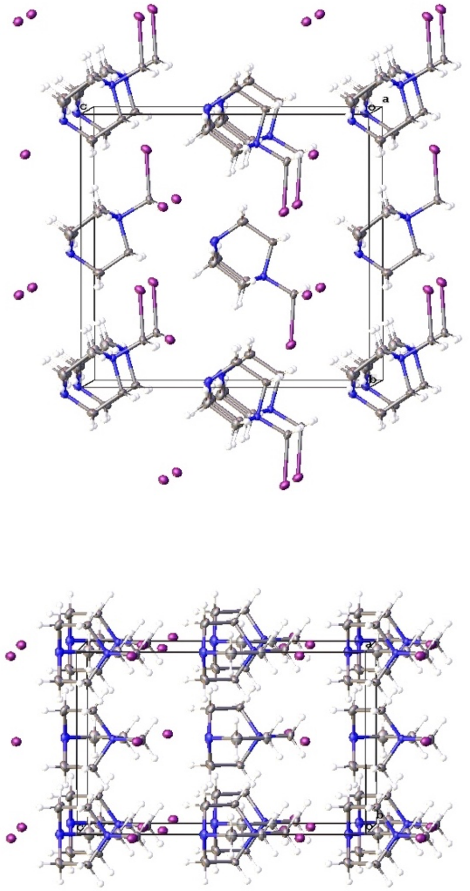

Figure S20. Crystal packing diagrams of **3-I** viewed along *a* axis (top) and *b* axis (bottom).

Table S11. Crystal data and structure refinement details for **3-I**

Empirical formula	$C_7H_{14}I_2N_2$
Formula weight	380.00
Temperature/K	193.00
Crystal system	orthorhombic
Space group	$Cmc2_1$
a/Å	7.7522(3)
b/Å	11.6193(3)
c/Å	12.2632(3)
α/°	90
β/°	90
γ/°	90
Volume/Å^3	1104.61(6)
Z	4
ρ_{calc}g/cm^3	2.285
μ/mm^{-1}	5.645
F(000)	704.0
Crystal size/mm^3	0.1 × 0.08 × 0.05
Radiation	MoKα (λ = 0.71073)
2Θ range for data collection/°	6.318 to 50.488
Index ranges	$-9 \leq h \leq 9$, $-13 \leq k \leq 13$, $-14 \leq l \leq 14$
Reflections collected	12698
Independent reflections	1086 [R_{int} = 0.0221, R_{sigma} = 0.0124]
Data/restraints/parameters	1086/1/61
Goodness-of-fit on F^2	1.070
Final R indexes [I>=2σ (I)]	R_1 = 0.0081, wR_2 = 0.0195
Final R indexes [all data]	R_1 = 0.0081, wR_2 = 0.0195
Largest diff. peak/hole / e Å^{-3}	0.16/-0.28
Flack parameter	0.028(11)

2.2 Single crystal X-ray structures of di(1H-indazol-1-yl)methane (**L1 - CCDC 2431971**), di(2H-indazol-2-yl)methane (**L2 - CCDC 2431972**) and (1H-indazol-1-yl)(2H-indazol-2-yl)methane (**L3 - CCDC 2431973**).

Crystals of indazole isomers were obtained by slow diffusion of *n*-hexane into a dichloromethane solution of the isomer, using closed, interconnected vessels.

The ORTEP representations of the **bis(indazolyl)methane isomers** are shown in Figure 3 of the main manuscript.

Table S12. Crystallographic data and structure refinement parameters for compounds **L1**, **L2**, and **L3**

	**L1**	**L3**	**L2**
Empirical formula	$C_{15}H_{12}N_4$	$C_{15}H_{12}N_4$	$C_{15}H_{12}$N4
Formula weight	248.29	248.29	248.29
Temperature/K	173.15	173.15	173.15
Crystal system	monoclinic	monoclinic	monoclinic
Space group	C2	$P2_1$	$P2_1/c$
a/Å	23.6048(7)	4.5053(3)	10.0876(5)
b/Å	4.10040(10)	11.5398(8)	8.1834(4)
c/Å	13.9516(4)	11.9930(8)	14.7602(7)
α/°	90	90	90
β/°	117.8300(10)	99.550(2)	98.168(2)
γ/°	90	90	90
Volume/Å^3	1194.18(6)	614.88(7)	1206.11(10)
Z	4	2	4
ρcalcg / cm^3	1.381	1.341	1.367
μ / mm^{-1}	0.087	0.084	0.086
F(000)	520.0	260.0	520.0
Crystal size / mm^3	0.5 × 0.26 × 0.25	0.11 × 0.1 × 0.03	0.22 × 0.2 × 0.19
Radiation	MoK$_α$ (λ = 0.71073)	MoK$_α$ (λ = 0.71073)	MoK$_α$ (λ = 0.71073)
2θ range for data collection/°	3.754 to 50.5	3.444 to 50.448	5.576 to 50.496
Index ranges	-26 ≤ h ≤ 28, -4 ≤ k ≤ 4, -16 ≤ l ≤ 16	-4 ≤ h ≤ 5, -13 ≤ k ≤ 13, -14 ≤ l ≤ 14	-12 ≤ h ≤ 12, -8 ≤ k ≤ 9, -17 ≤ l ≤ 17
Reflections collected	12437	11473	19460
Independent reflections	2154, [R_{int} = 0.0423,	2055, [R_{int} = 0.0259,	2179, [R_{int} = 0.0235,

	R_{sigma} = 0.0394]	R_{sigma} = 0.0201]	R_{sigma} = 0.0138]
Data/restraints/parameters	2154/1/173	2055/1/172	2179/0/172
Goodness-of-fit on F^2	1.078	1.088	1.093

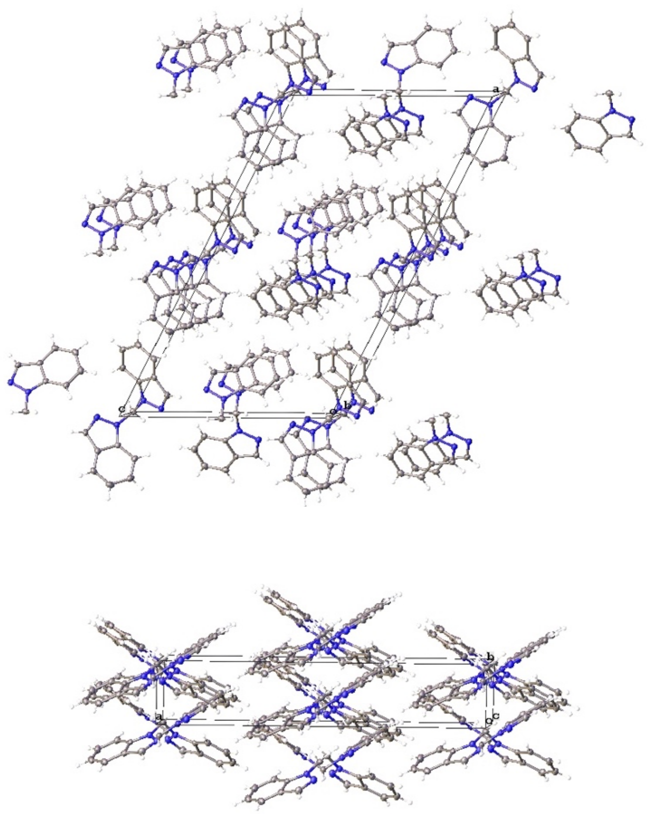

Figure S21. Crystal packing diagrams of **L1** viewed along *b* axis (top) and *c* axis (bottom).

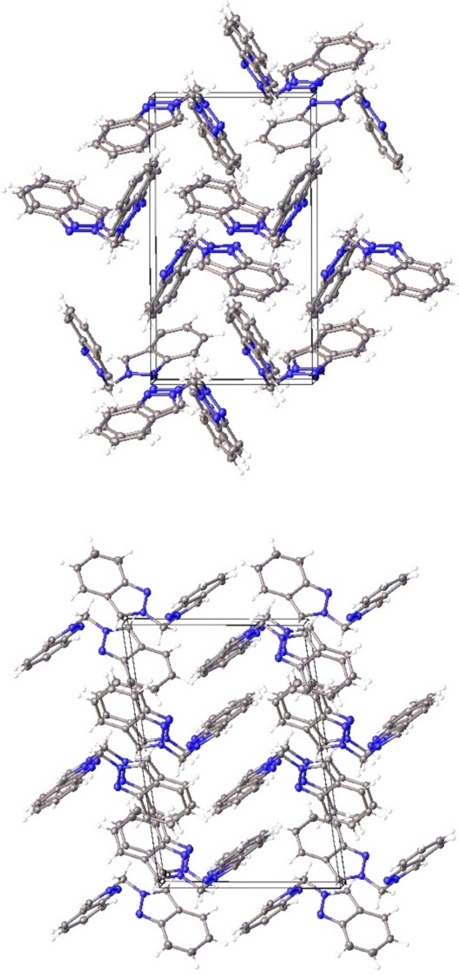

Figure S22. Crystal packing diagrams of **L2** viewed along *a* axis (top) and *b* axis (bottom).

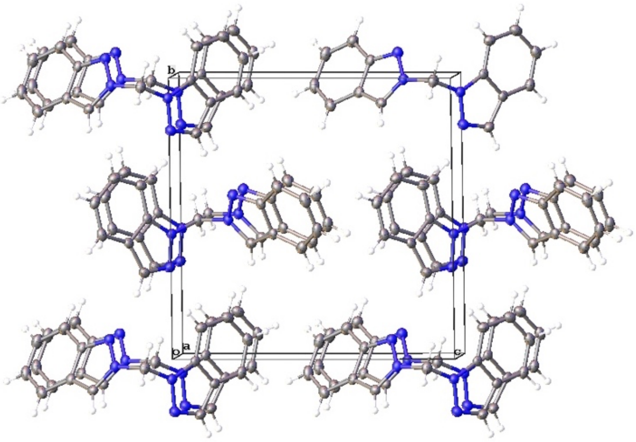

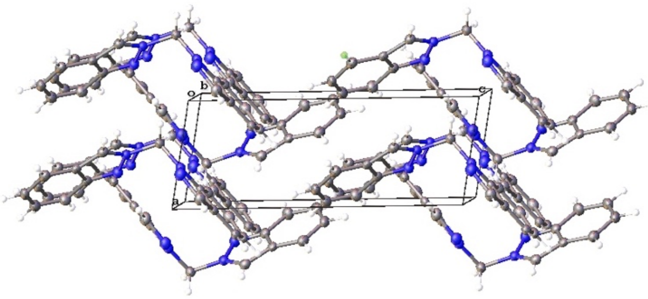

Figure S23. Crystal packing diagrams of **L3** viewed along *a* axis (top) and *b* axis (bottom).

2.3 Single crystal X-ray structure of 1-(2H-indazol-2-yl)ethan-1-one (**Ind-N2-Ac**) - **CCDC 2431974**

Crystals of compound **Ind-N2-Ac** were obtained in pure form (colorless needles) through slow evaporation from a mixture of isomers in dichloromethane.

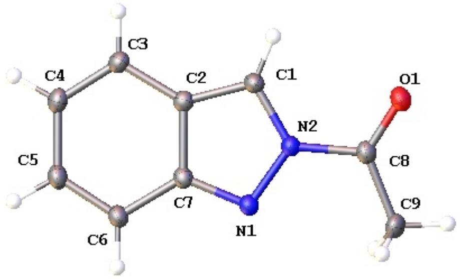

Figure S24. ORTEP representation of **Ind-N2-Ac** with thermal ellipsoids shown at the 50% probability level.

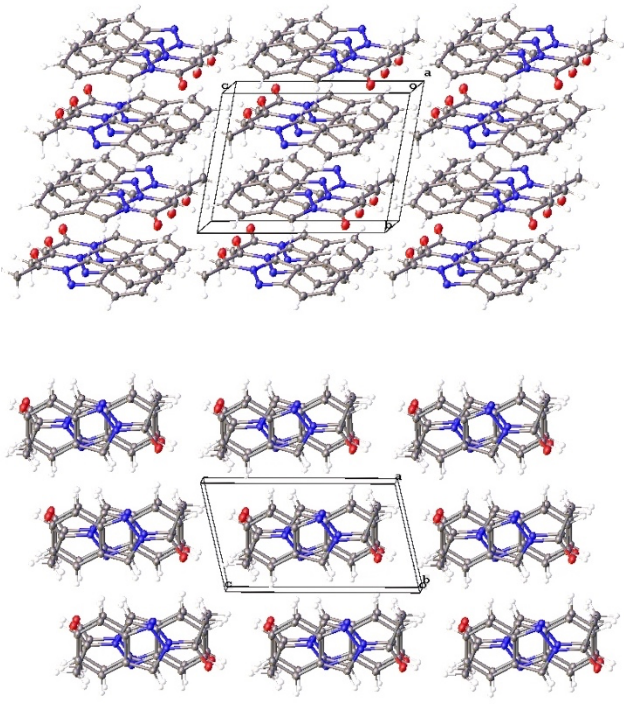

Figure S25. Crystal packing diagrams of **Ind-N2-Ac** viewed along *a* axis (top) and *b* axis (bottom).

Table S13. Crystal data and structure refinement details for **Ind-N2-Ac**

Empirical formula	$C_9H_8N_2O$
Formula weight	160.17
Temperature/K	193.00
Crystal system	triclinic
Space group	P-1
a/Å	5.5536(12)
b/Å	7.3423(19)
c/Å	10.005(2)
α/°	74.112(9)
β/°	74.916(8)
γ/°	79.635(9)
Volume/Å^3	376.27(16)
Z	2
ρ_{calc}g/cm^3	1.414
μ/mm^{-1}	0.096

F(000)	168.0
Crystal size/mm^3	0.5 × 0.2 × 0.15
Radiation	MoKα (λ = 0.71073)
2Θ range for data collection/°	5.808 to 50.492
Index ranges	$-6 \le h \le 6$, $-8 \le k \le 8$, $-11 \le l \le 11$
Reflections collected	11992
Independent reflections	1354 [R_{int} = 0.0638, R_{sigma} = 0.0372]
Data/restraints/parameters	1354/0/110
Goodness-of-fit on F^2	1.105
Final R indexes [I>=2σ (I)]	R_1 = 0.0616, wR_2 = 0.1727
Final R indexes [all data]	R_1 = 0.0712, wR_2 = 0.1830
Largest diff. peak/hole / e Å^{-3}	0.33/-0.30

3 NMR Spectra

Spectra are displayed at the maximum acquisition window available for each dataset. Where original FIDs permitted, spectra were re-exported to extended ranges; otherwise, the processed plots contain all observed resonances used for complete assignment. Display windows were limited to resonance regions to maximize digital resolution and avoid inclusion of baseline noise, ensuring accurate determination of chemical shifts and coupling constants.

3.1 NMR spectra of di(1H-indazol-1-yl)methane (***L1***)

3.1.1 ^{1}H NMR (500 MHz, $CDCl_3$) spectrum of ***L1***

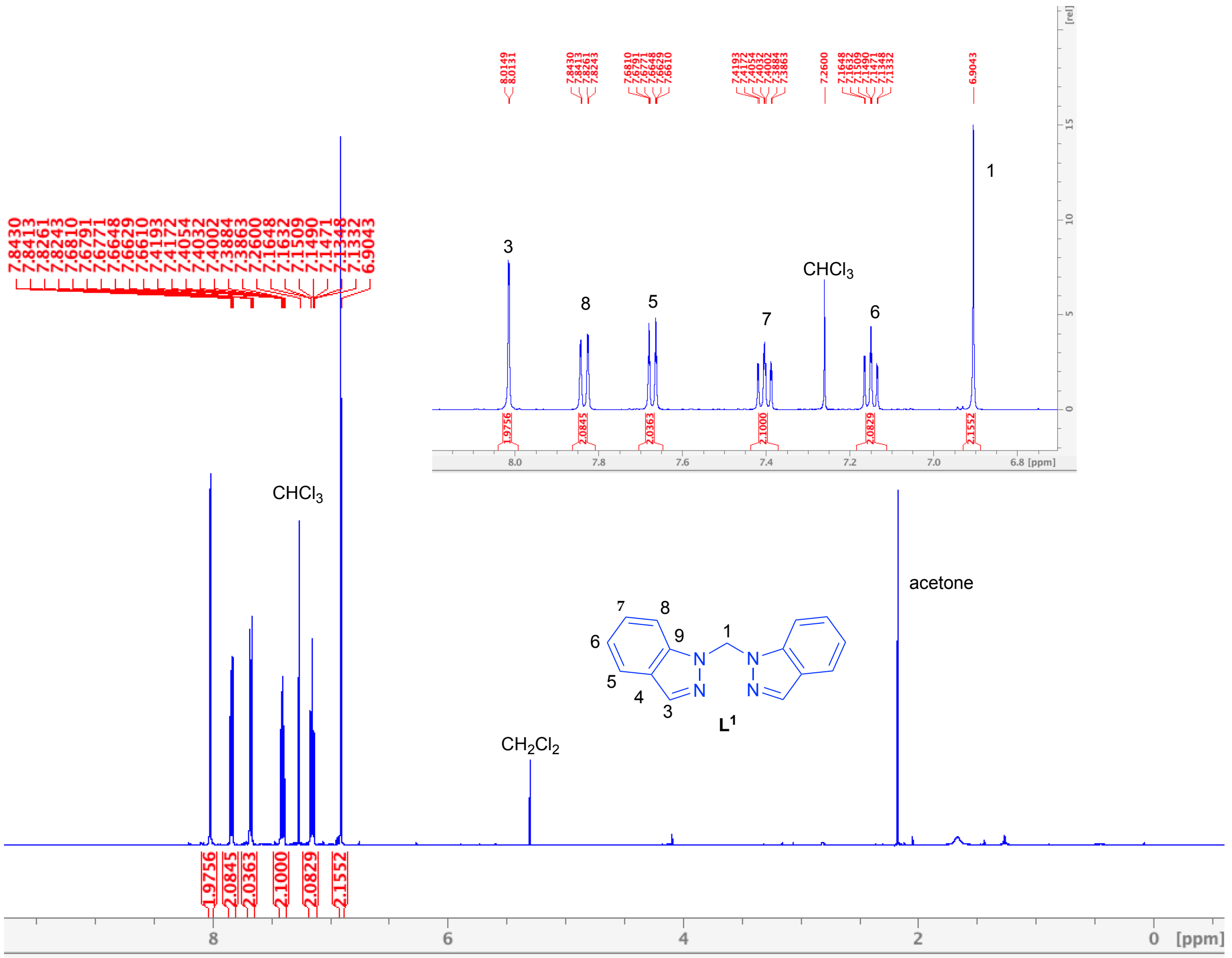

3.1.2 Selective 1D NOESY (500 MHz, $CDCl_3$) spectrum of ***L1***

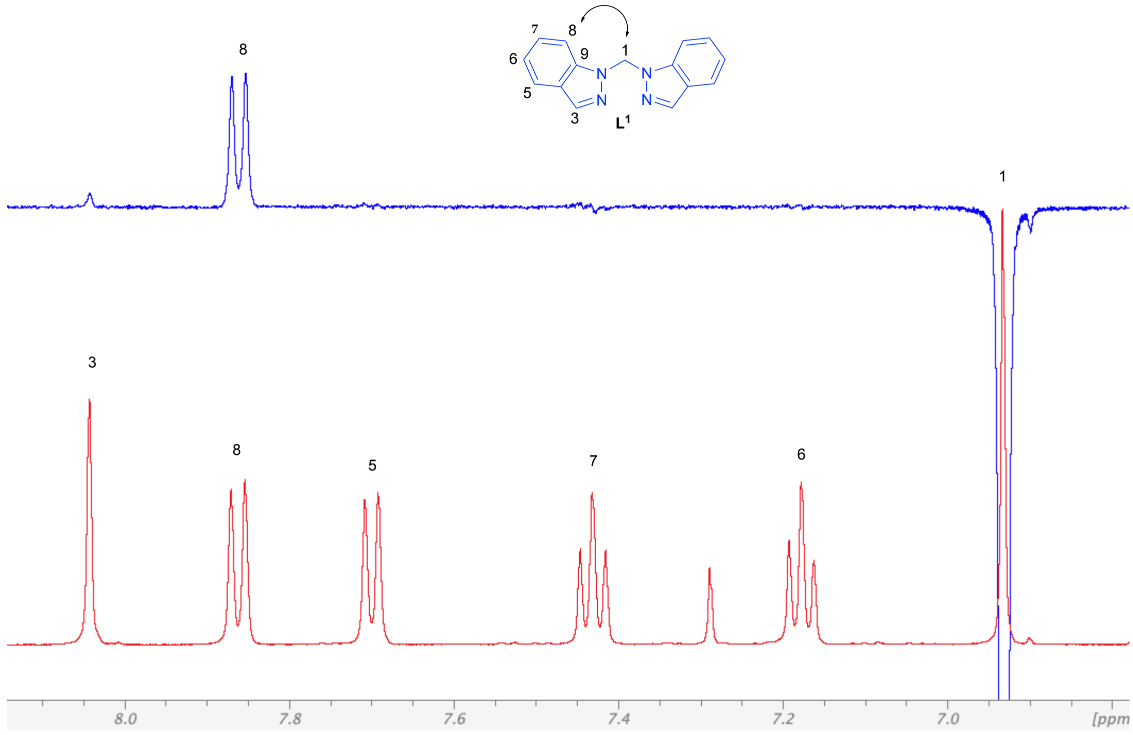

3.1.3 ^{1}H-^{1}H COSY NMR (500 MHz, $CDCl_3$) spectrum of ***L1***

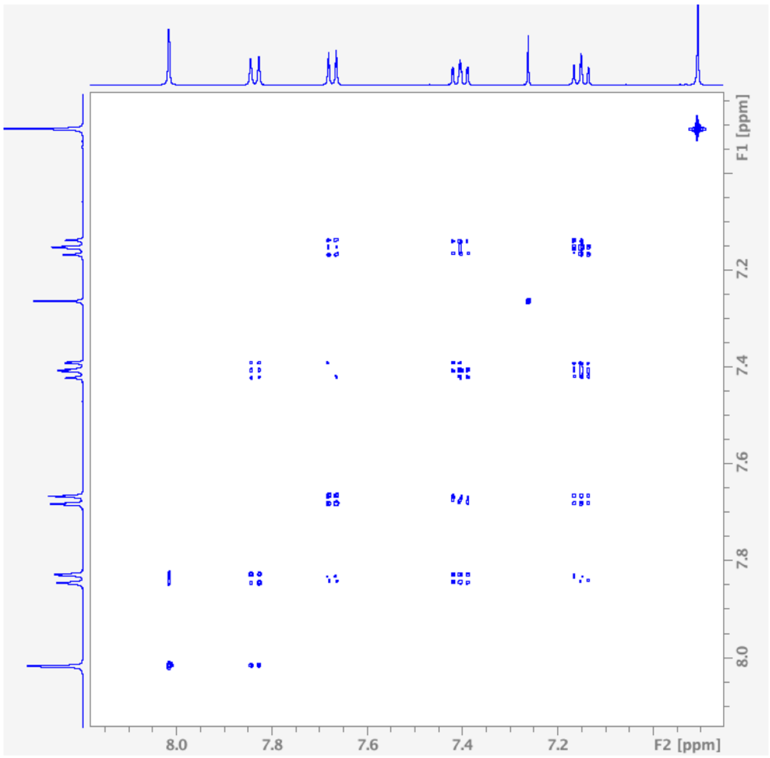

3.1.4 HMQC NMR (500 MHz, $CDCl_3$) spectrum of ***L1***

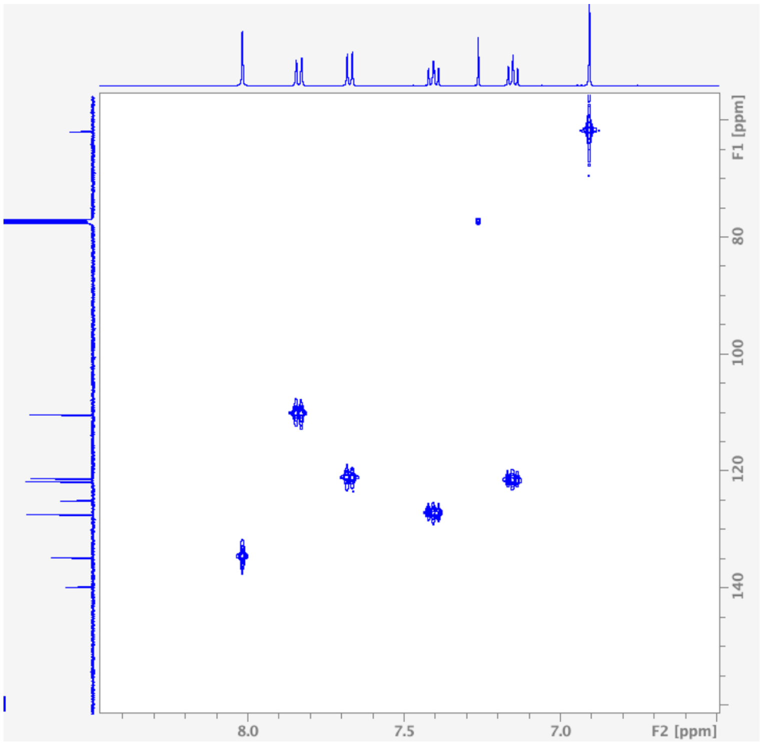

3.1.5 ^{13}C NMR (125 MHz, $CDCl_3$) spectrumof ***L1***

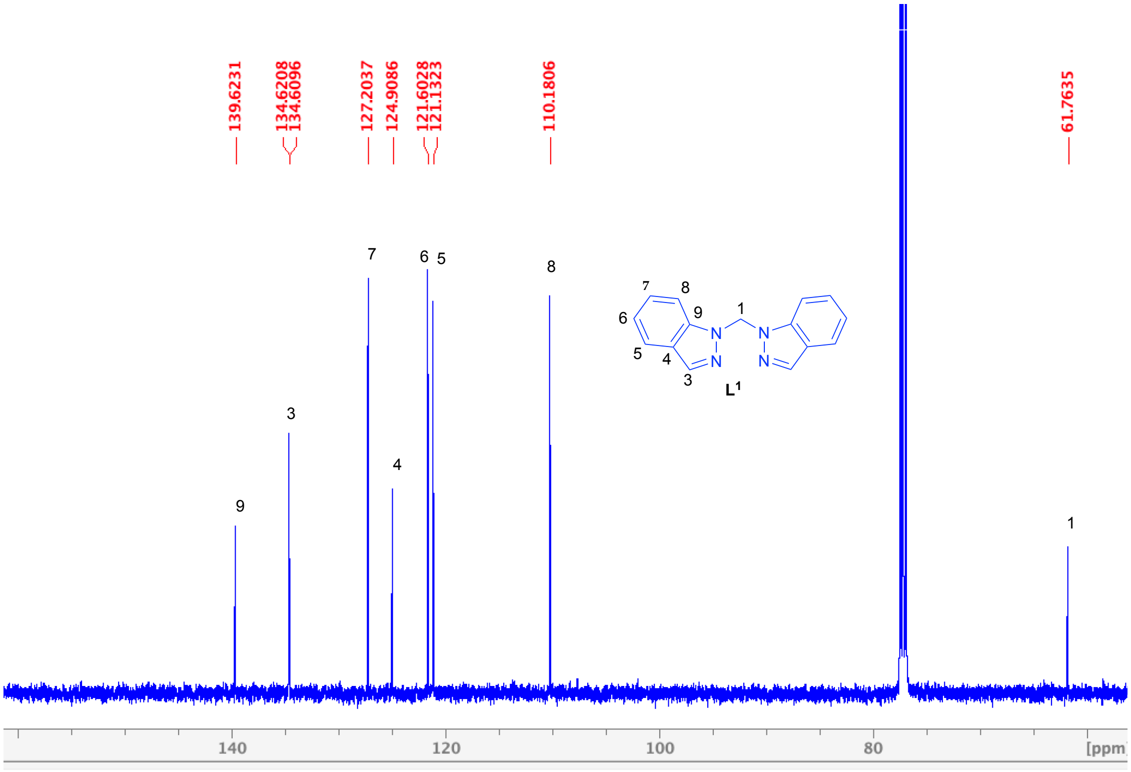

3.2 NMR spectra of di(2H-indazol-2-yl)methane (***L2***)

3.2.1 ^{1}H NMR (500 MHz, $CDCl_3$) spectrum of ***L2***

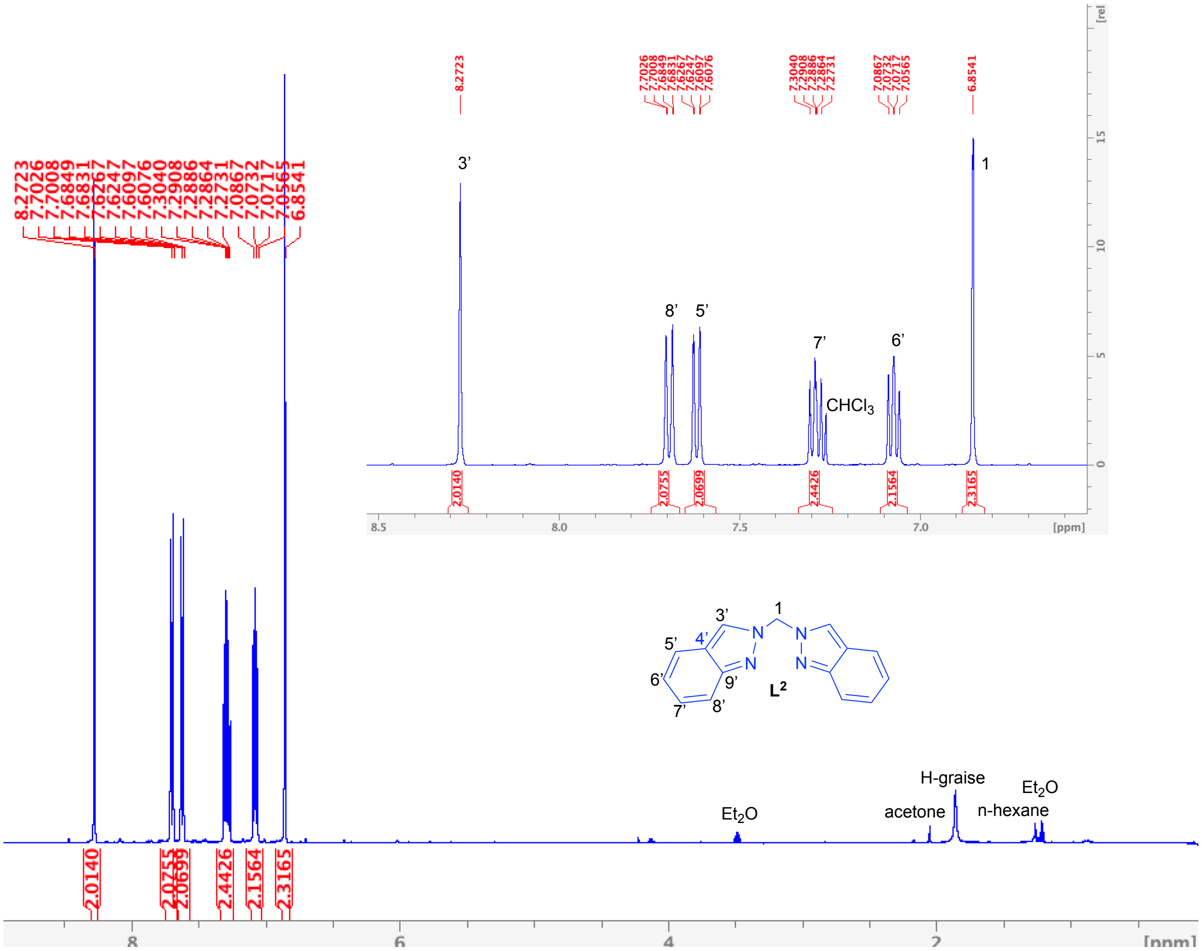

3.2.2 Selective 1D NOESY (500 MHz, $CDCl_3$) spectrum of ***L2***

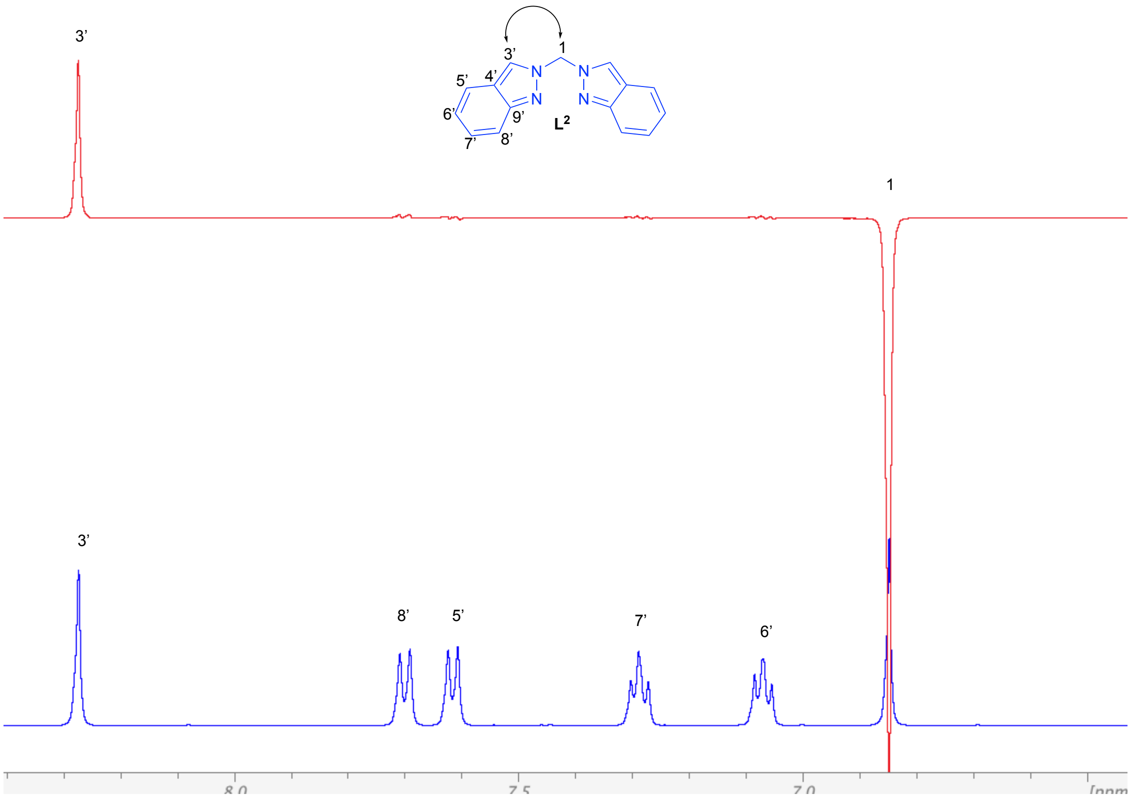

3.2.3 ^{1}H-^{1}H COSY NMR (500 MHz, $CDCl_3$) spectrum of ***L2***

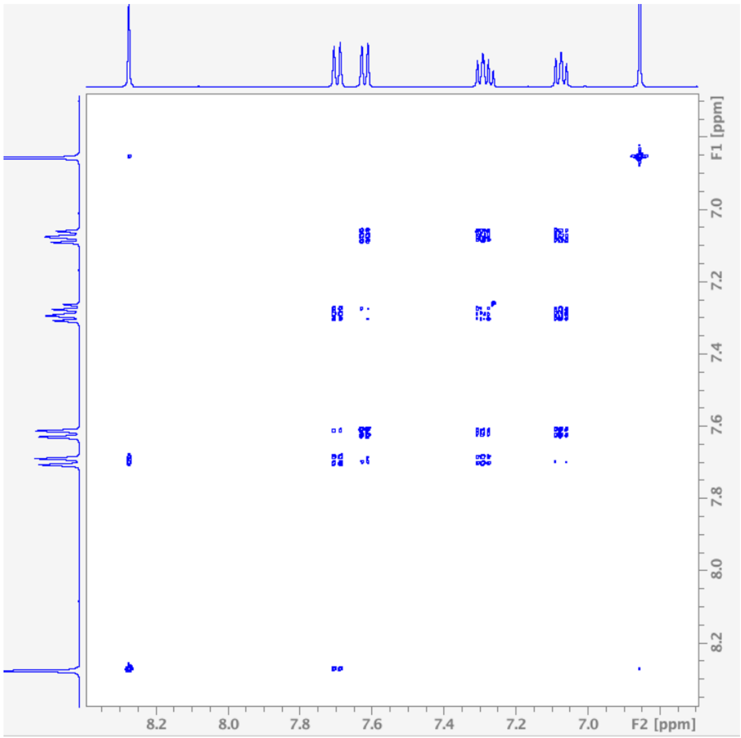

3.2.4 HMQC NMR (500 MHz, $CDCl_3$) spectrum of ***L2***

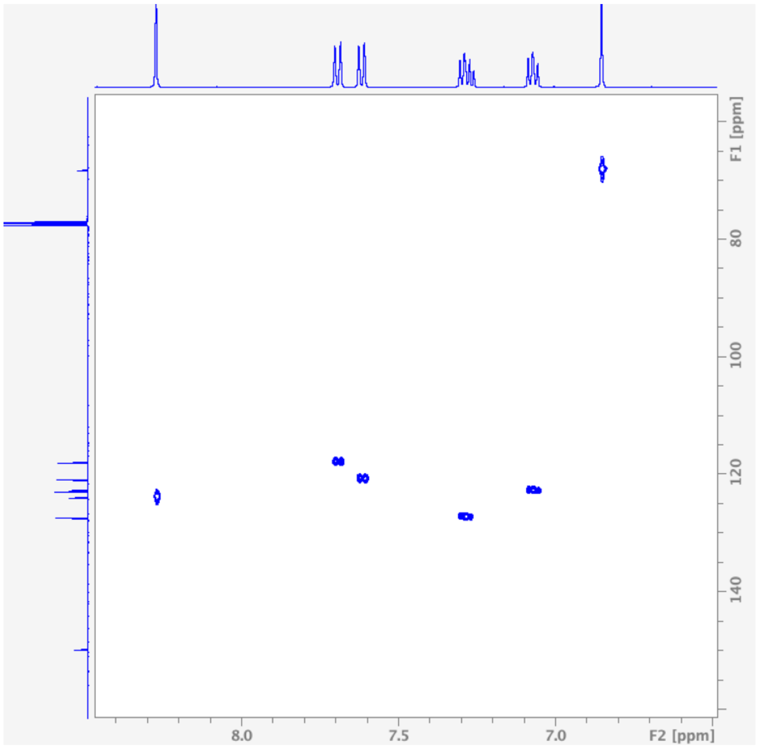

3.2.5 ^{13}C NMR (125 MHz, $CDCl_3$) spectrum of ***L2***

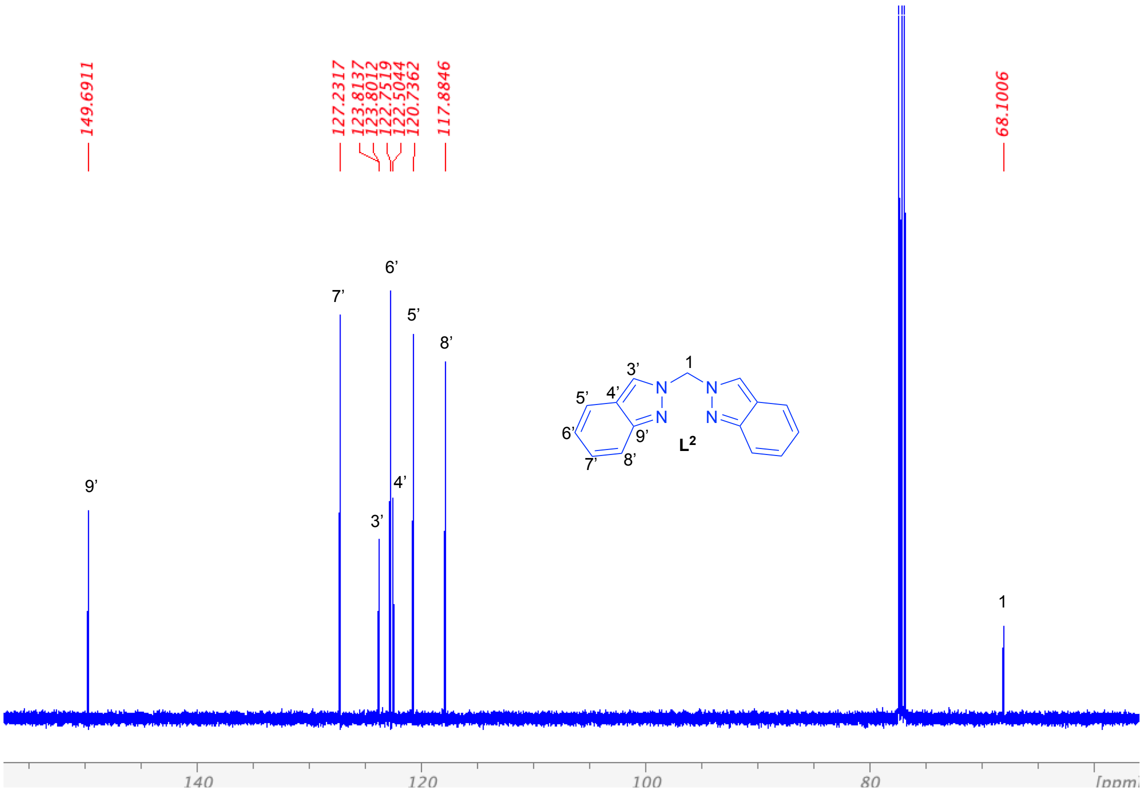

3.3 NMR spectra of (1H-indazol-1-yl)(2H-indazol-2-yl)methane (***L3***)

3.3.1 ^{1}H NMR (500 MHz, $CDCl_3$) spectrum of ***L3***

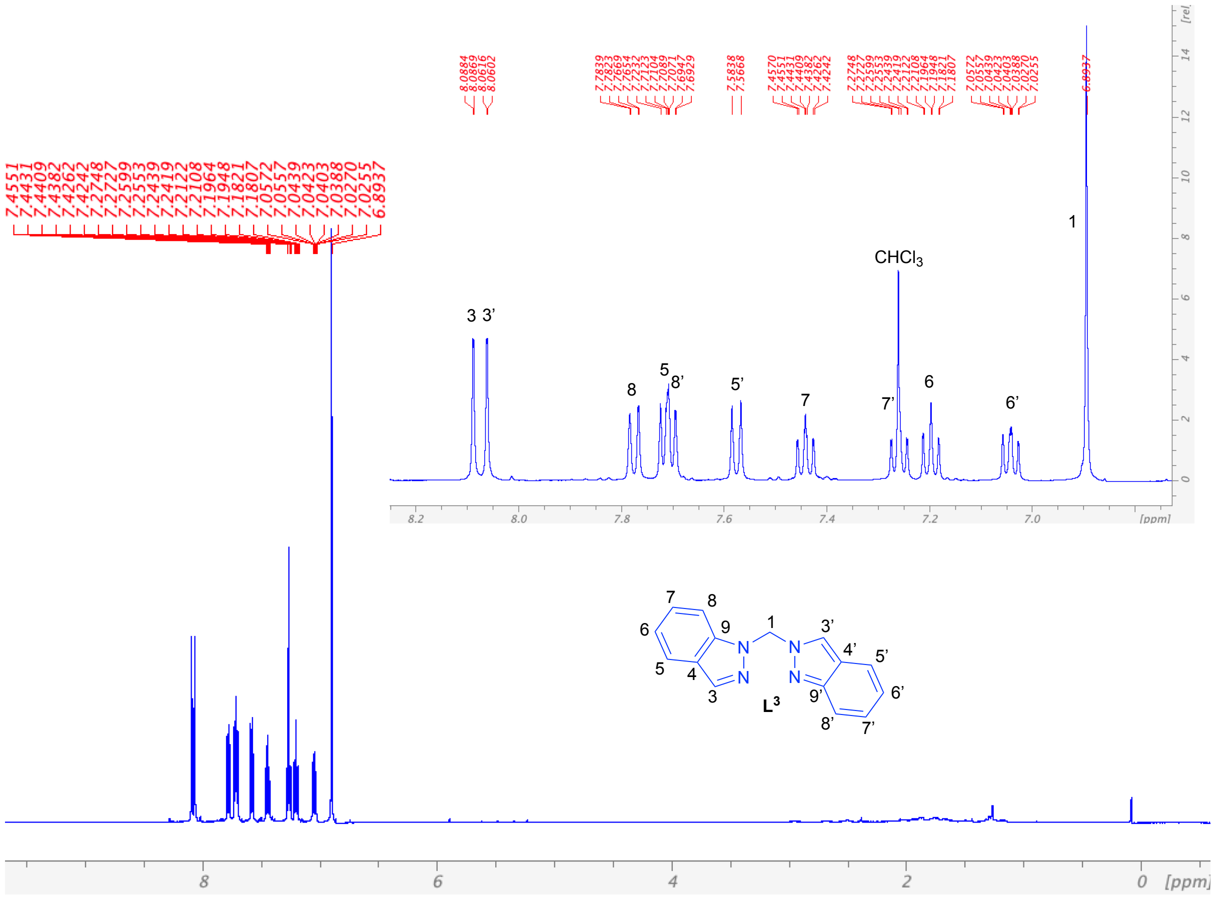

3.3.2 Selective 1D NOESY (500 MHz, $CDCl_3$) spectrum of ***L3***

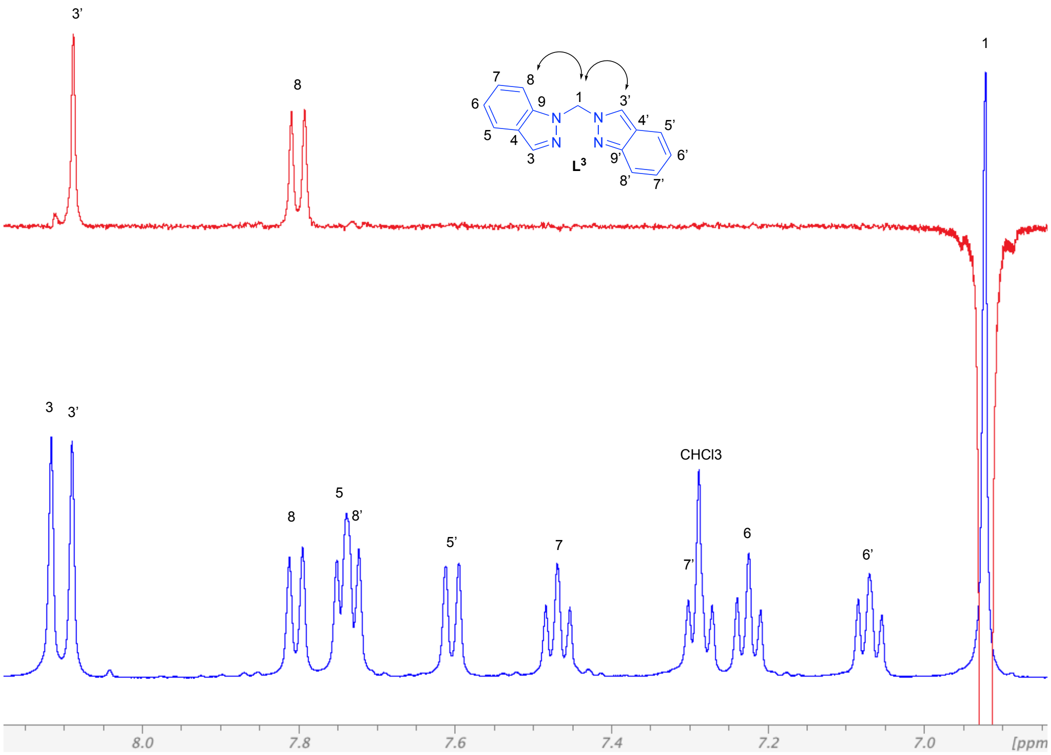

3.3.3 ${}^1H$-${}^1H$ COSY NMR (500 MHz, $CDCl_3$) spectrum of ***L3***

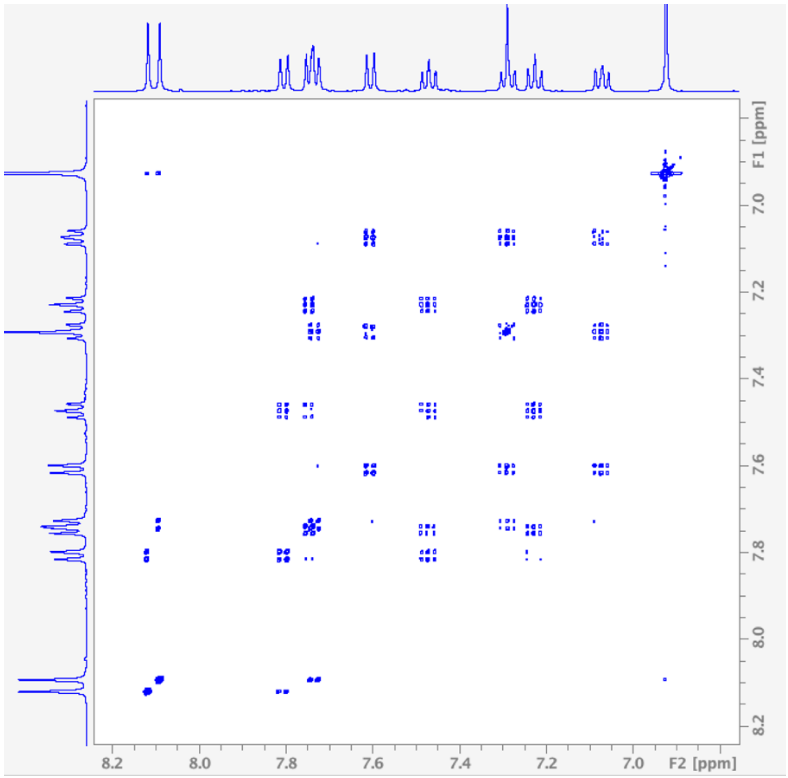

3.3.4 HMQC NMR (500 MHz, $CDCl_3$) spectrum of ***L3***

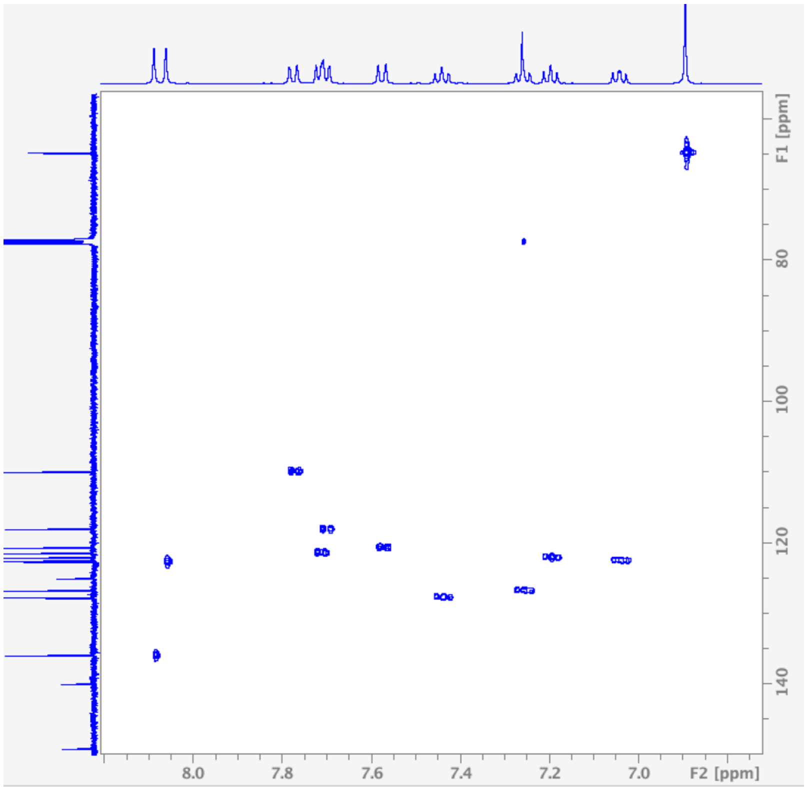

3.3.5 ^{13}C NMR (125 MHz, $CDCl_3$) spectrum of ***L3***

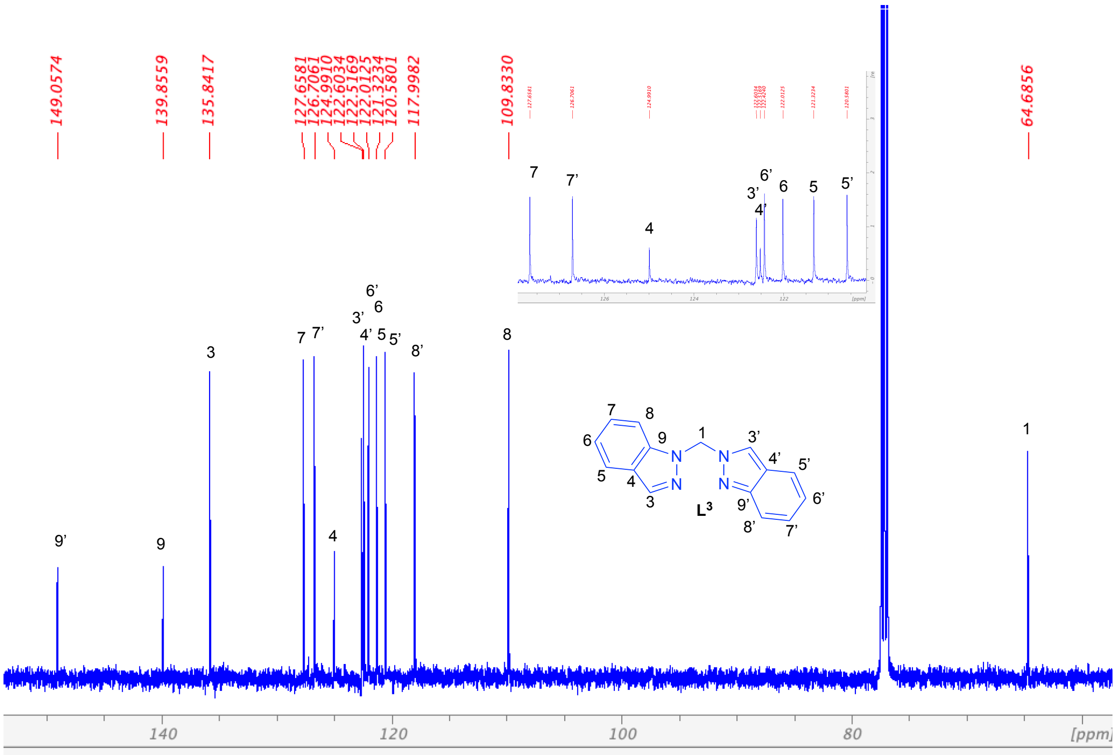

3.4 NMR spectra of **Indazole acetate isomers**

3.4.1 ^{1}H NMR (400 MHz, $CDCl_3$) spectrum of 1-(1H-indazol-1-yl)ethan-1-one (**Ind-N1-Ac**).

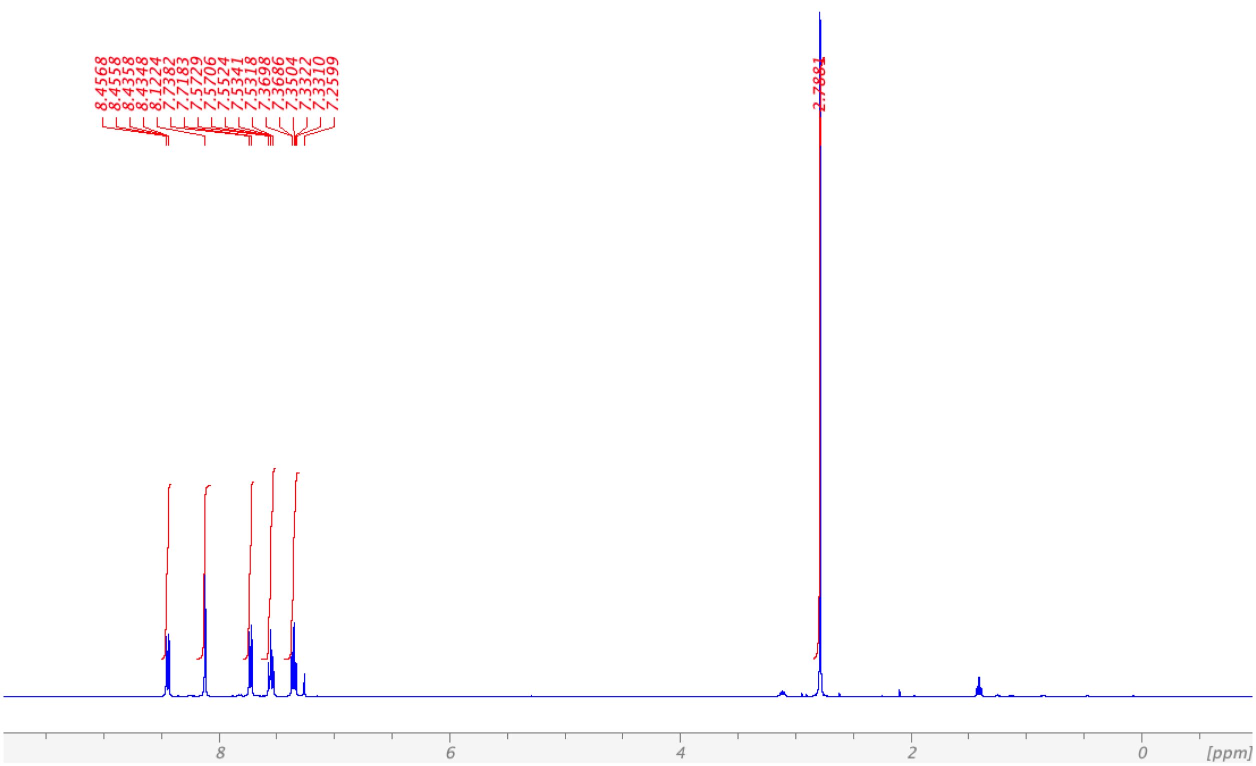

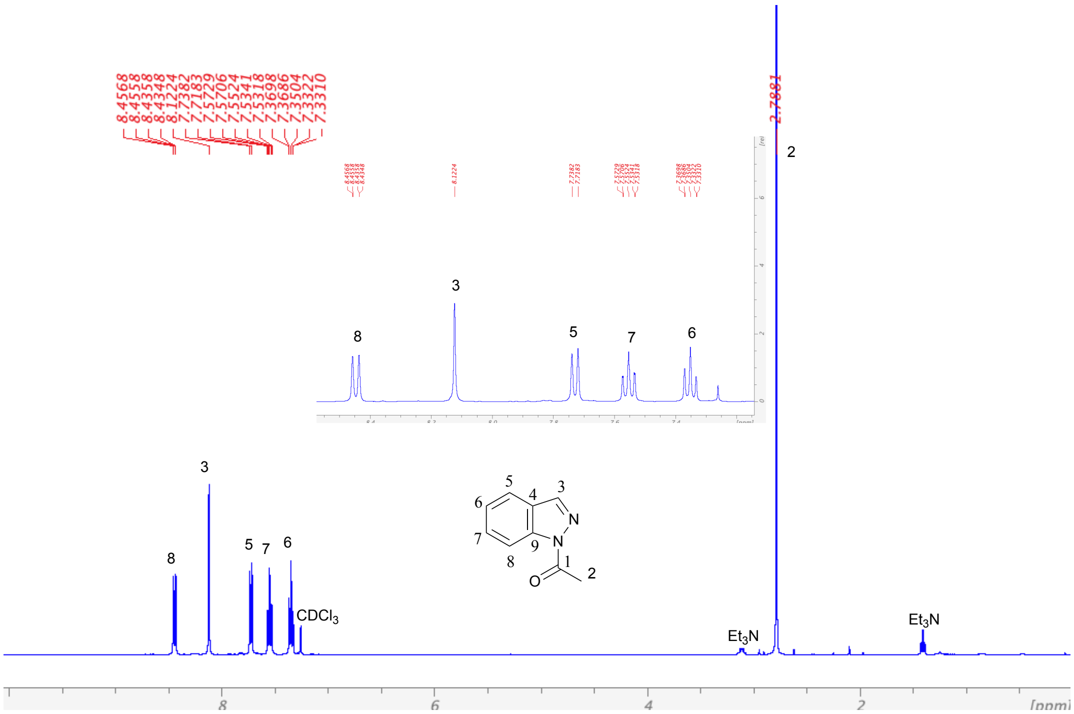

3.4.2 ^{1}H-^{1}H COSY NMR (400 MHz, $CDCl_3$) spectrum of **Ind-N1-Ac**

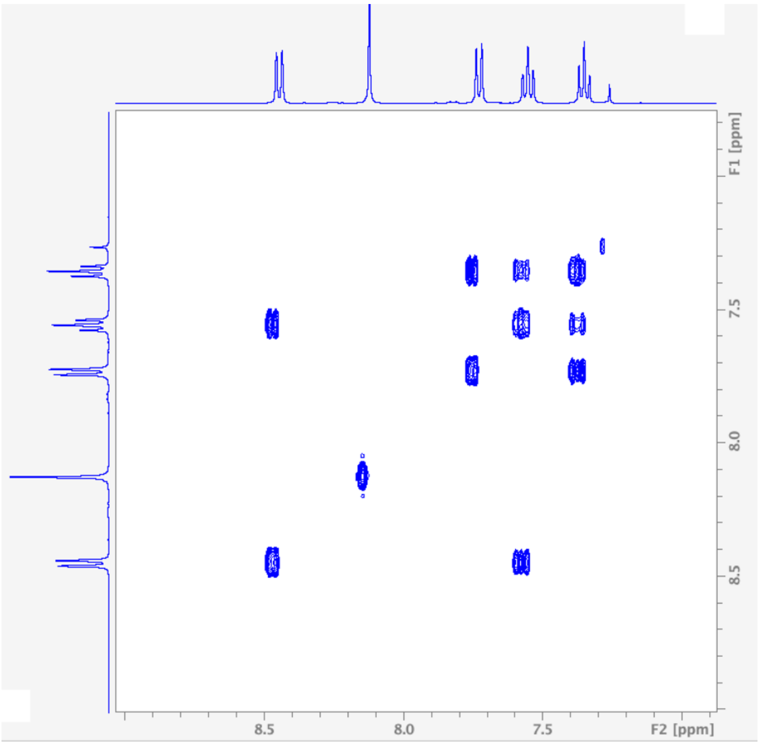

3.4.3 HSQC NMR (400 MHz, $CDCl_3$) spectrum of **Ind-*N1-Ac***

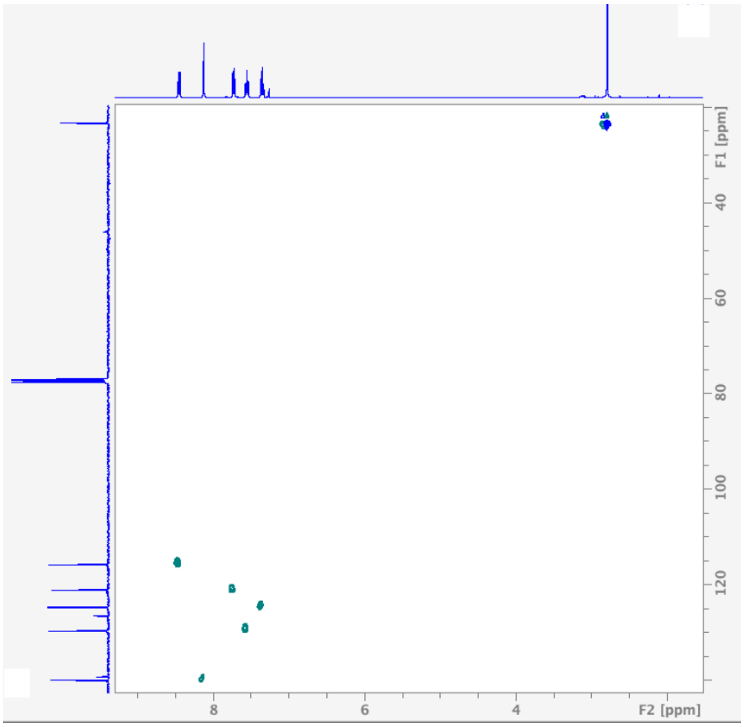

3.4.4 ^{13}C NMR (100 MHz, $CDCl_3$) spectrum of **Ind-N1-Ac**

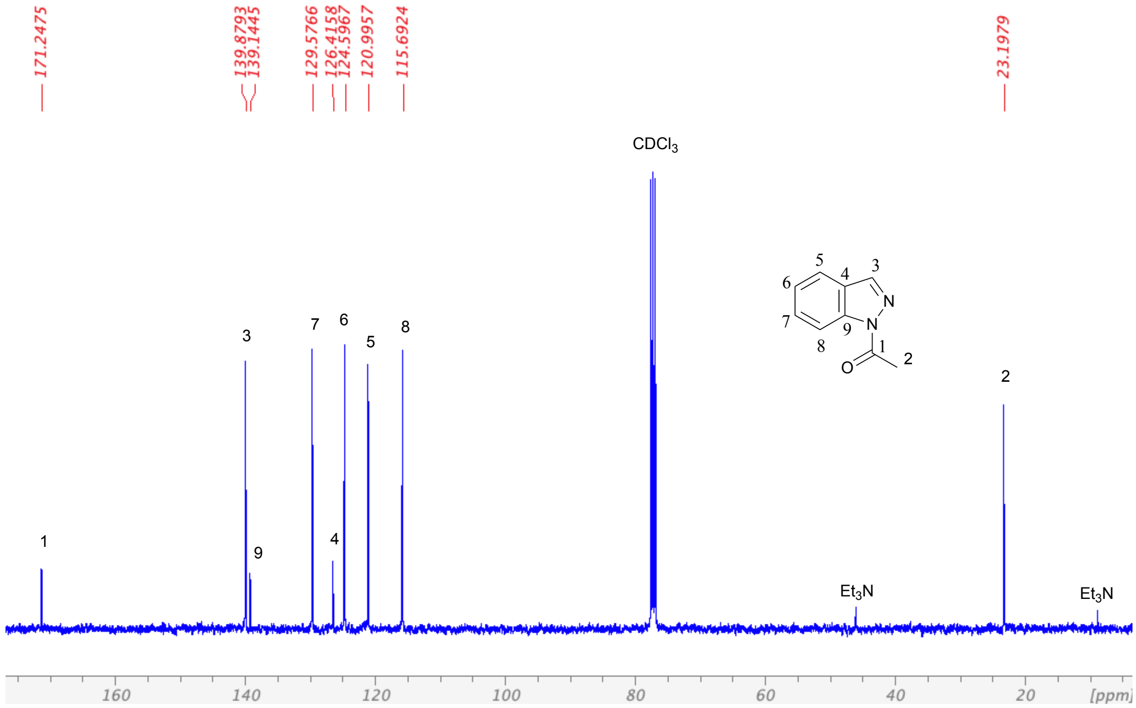

3.4.5 ^{1}H NMR (400 MHz, $CDCl_3$) spectrum of 1-(2H-indazol-2-yl)ethan-1-one (**Ind-N2-Ac,** major) mixed with 1-(1H-indazol-1-yl)ethan-1-one (**Ind-N1-Ac**, minor)

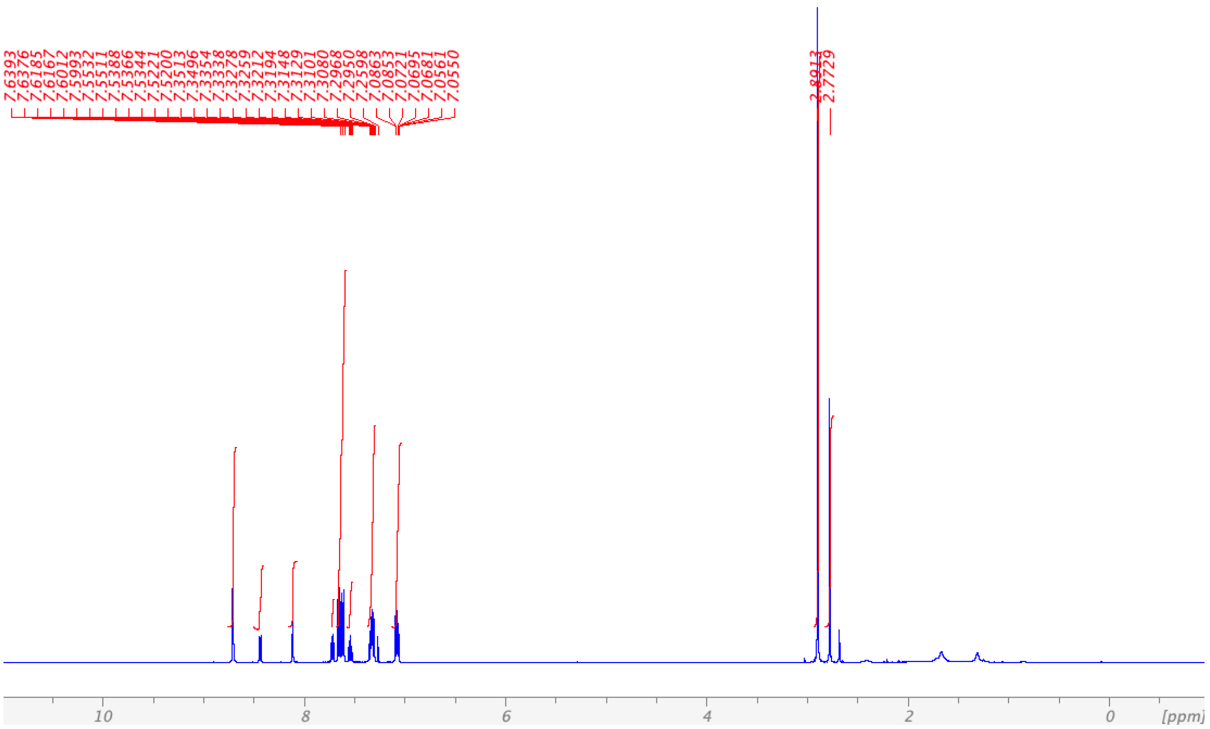

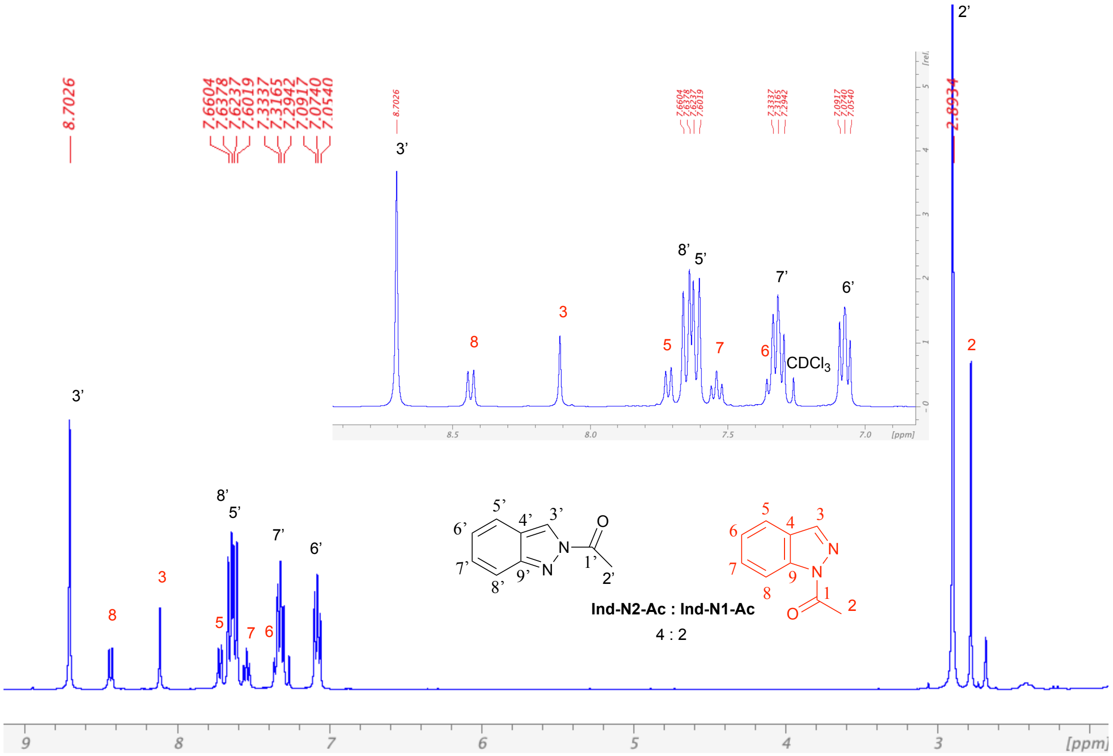

3.4.6 ^{1}H-^{1}H COSY NMR (400 MHz, $CDCl_3$) spectrum of **Ind-N2-Ac**

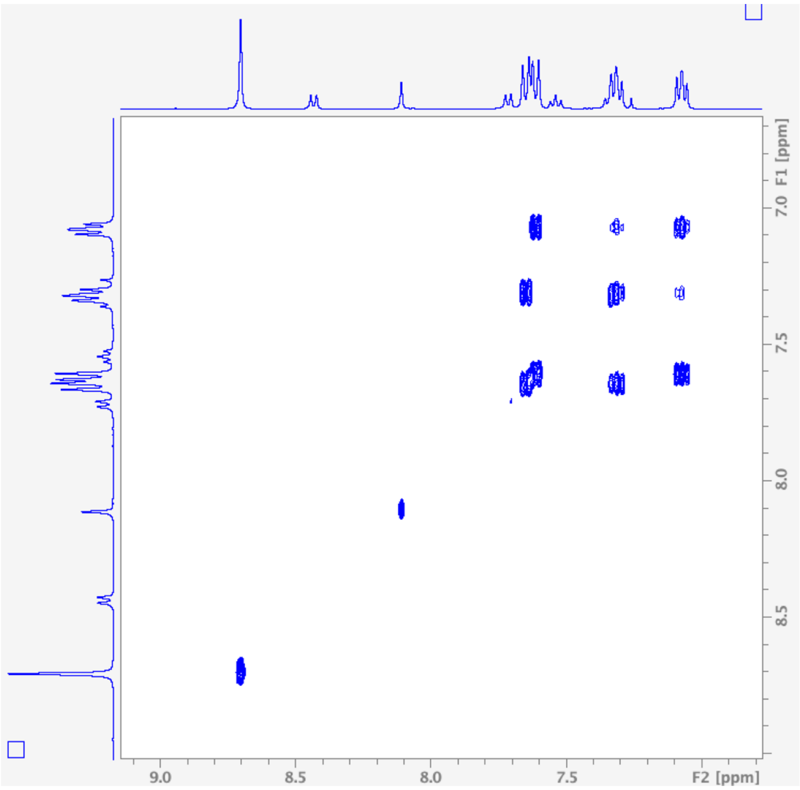

3.4.7 HSQC NMR (400 MHz, $CDCl_3$) spectrum of **Ind-N2-Ac**

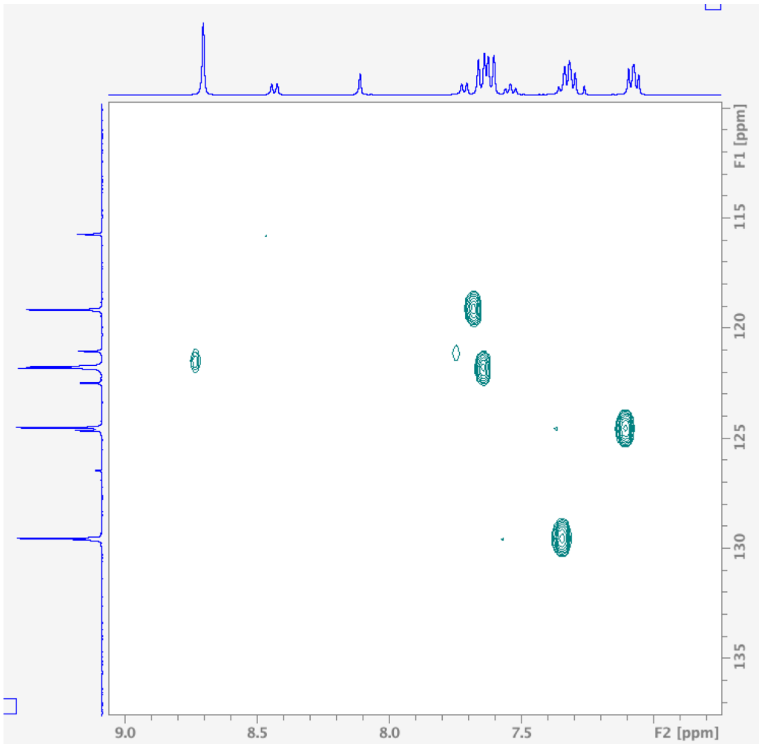

3.4.8 ^{13}C NMR (100 MHz, $CDCl_3$) spectrum of **Ind-N2-Ac**

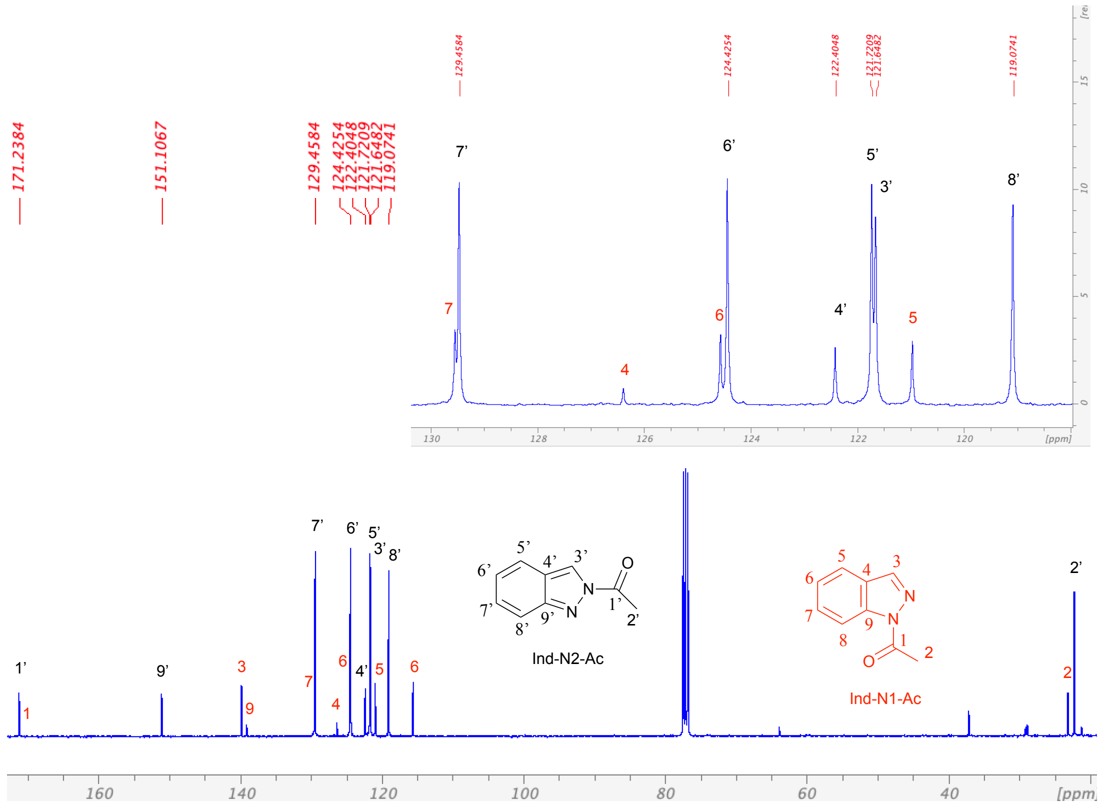

3.5 NMR spectra of **mono- and bis quaternary ammonium salts**

3.5.1 ^{1}H NMR (500 MHz, CD_3OD) spectrum of **1-Cl**

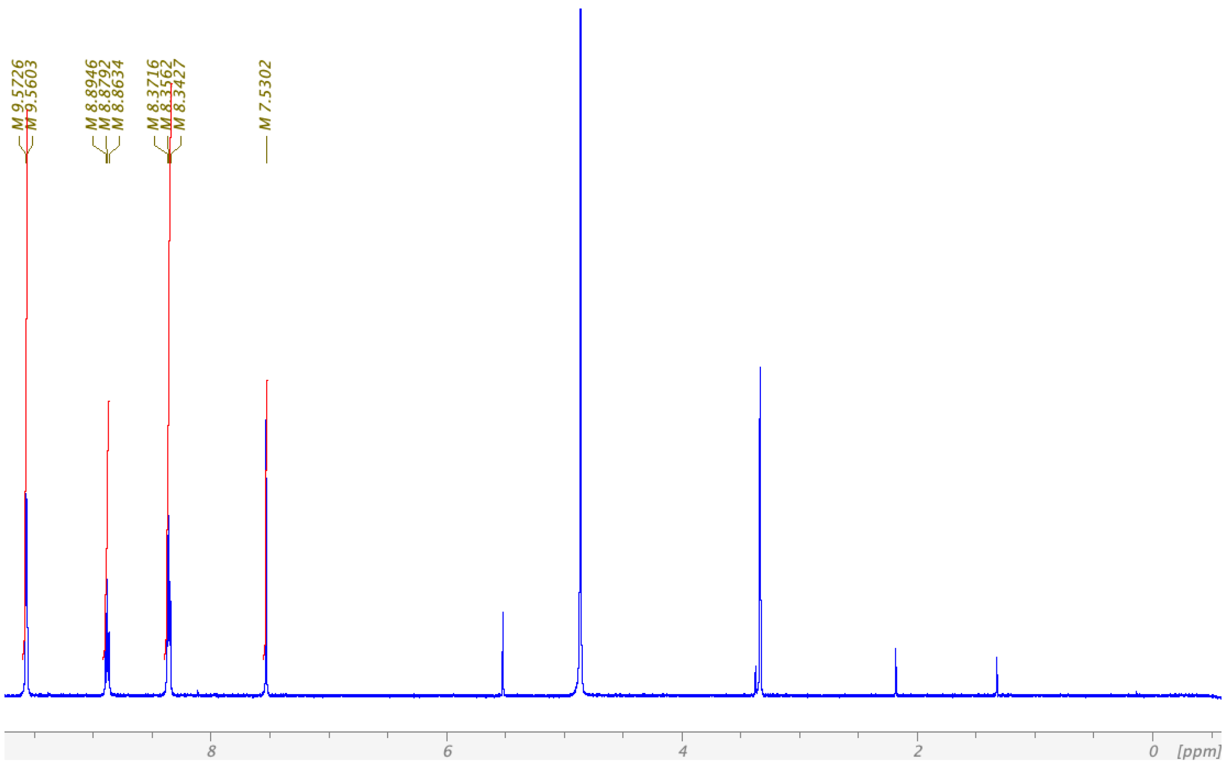

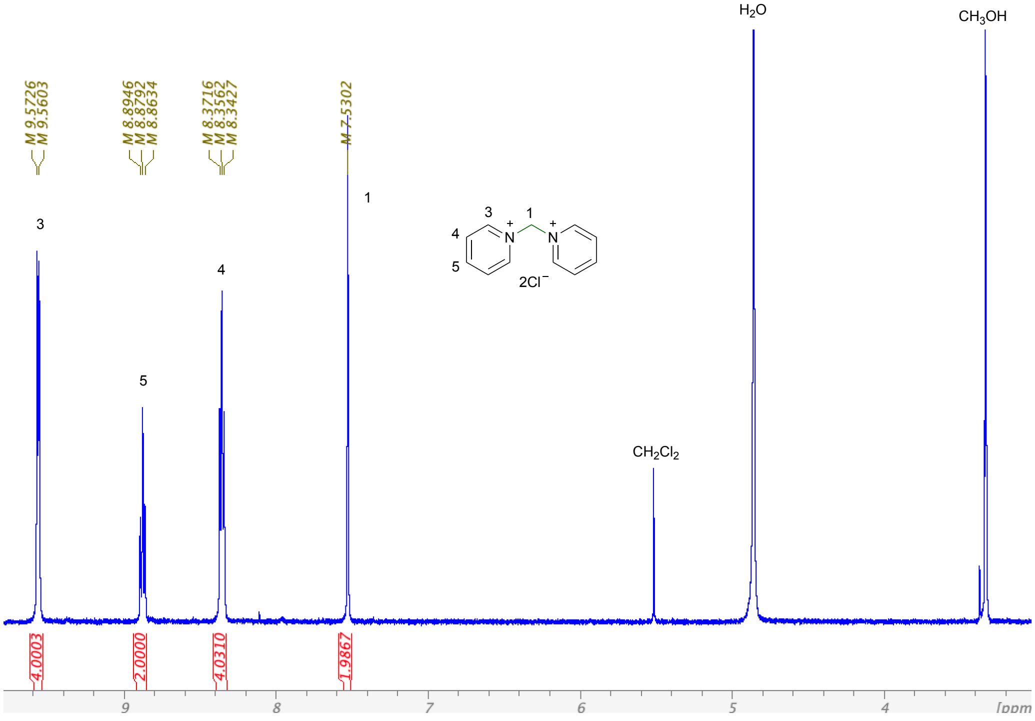

3.5.2 ^{13}C NMR (125 MHz, CD_3OD) spectrum of **1-Cl**

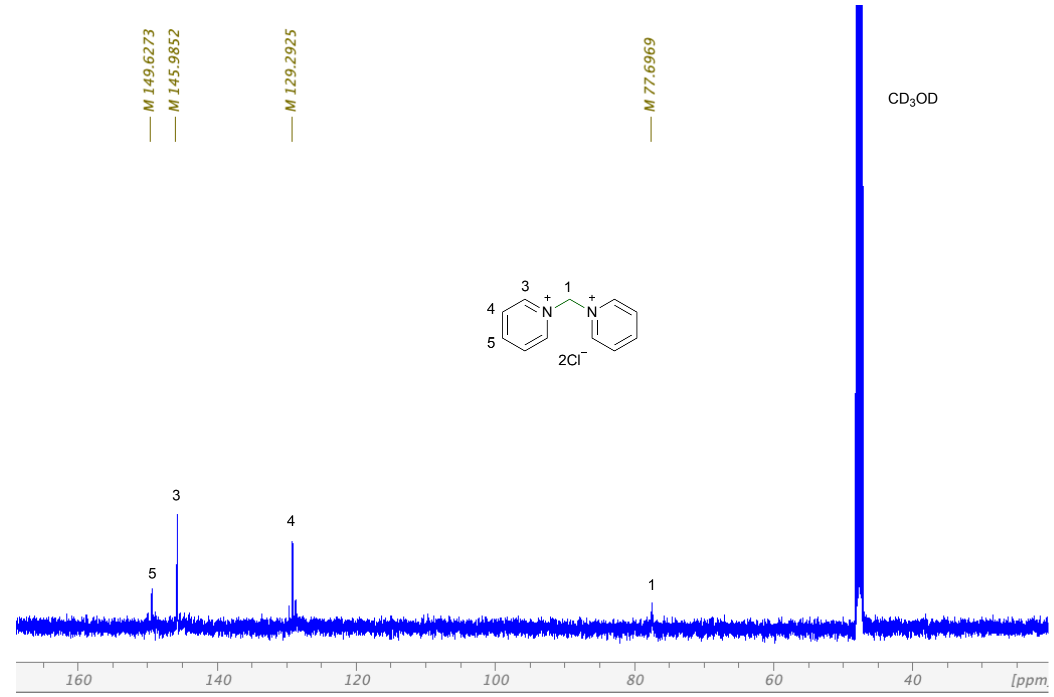

3.5.3 ^{1}H NMR (500 MHz, CD_3OD) spectrum of **1-Br**

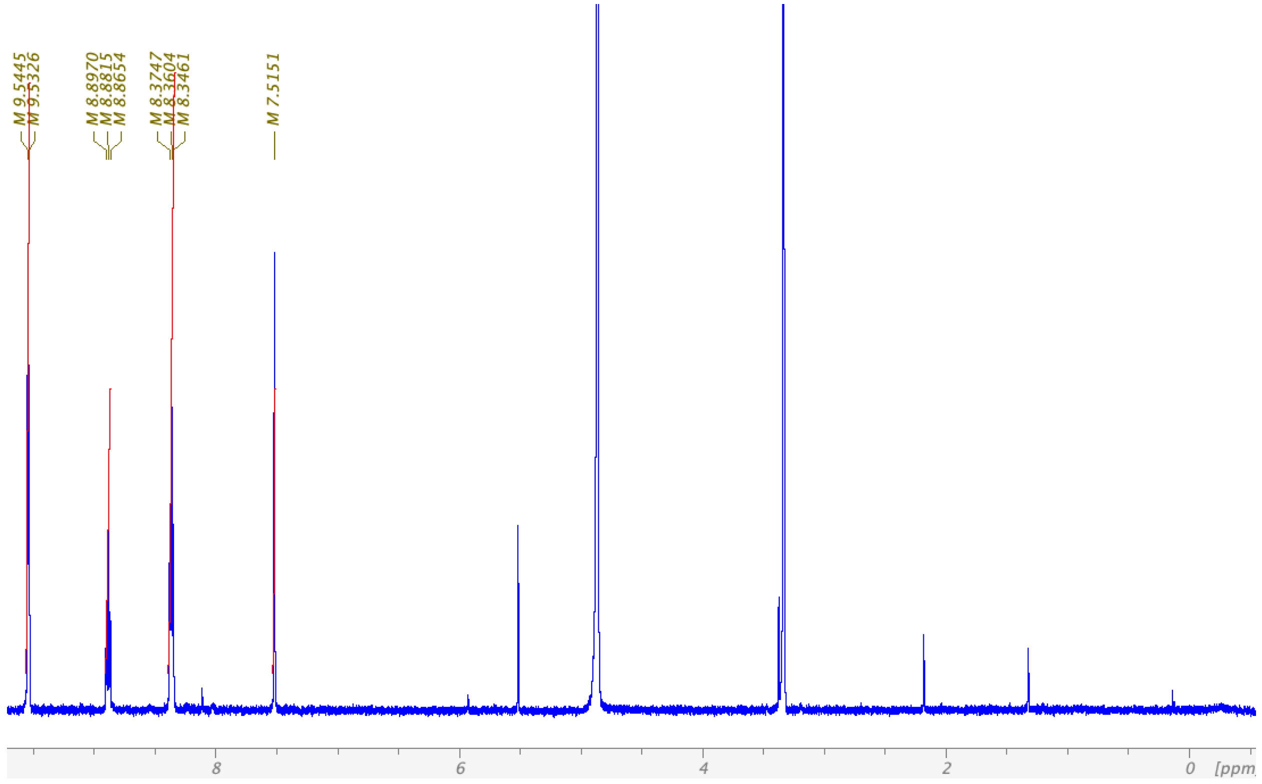

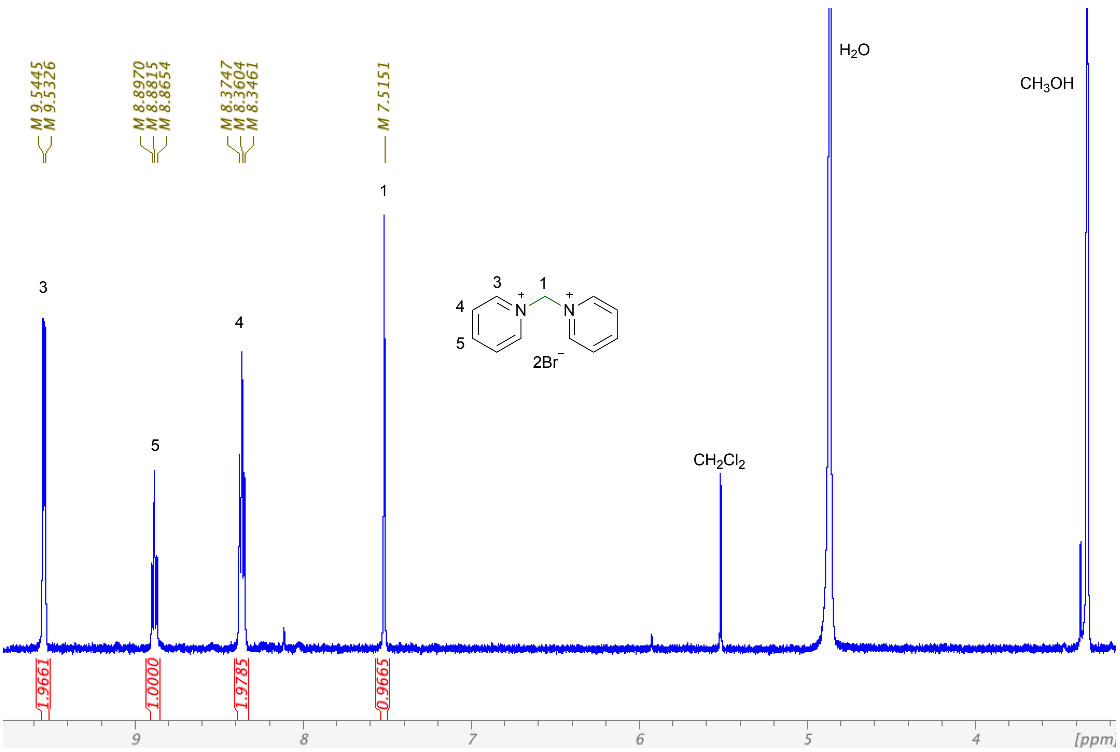

3.5.4 HSQC NMR (500 MHz, CD_3OD) spectrum of **1-Br**

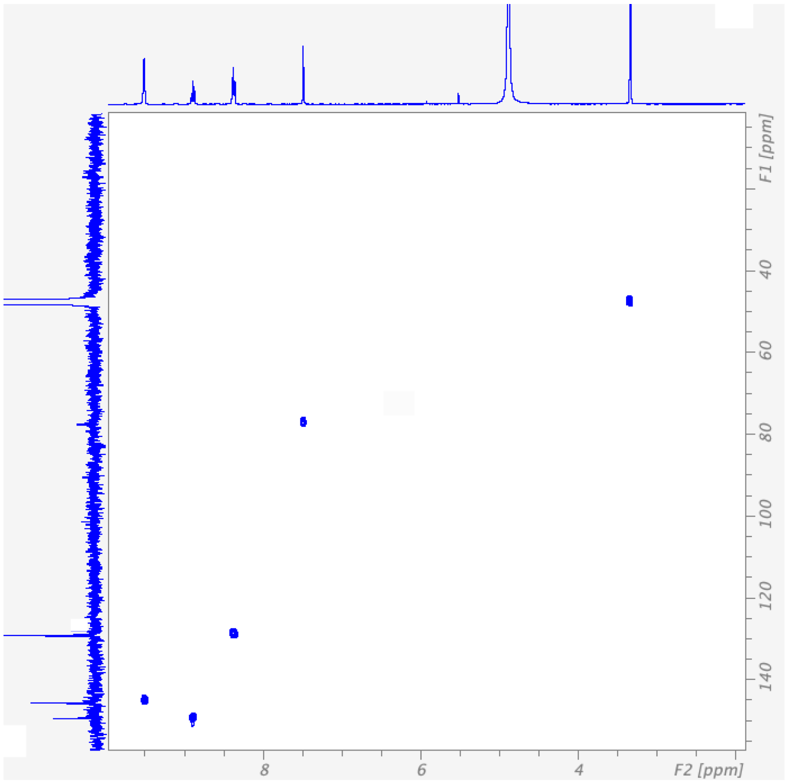

3.5.5 ^{13}C NMR (125 MHz, CD_3OD) spectrum of **1-Br**

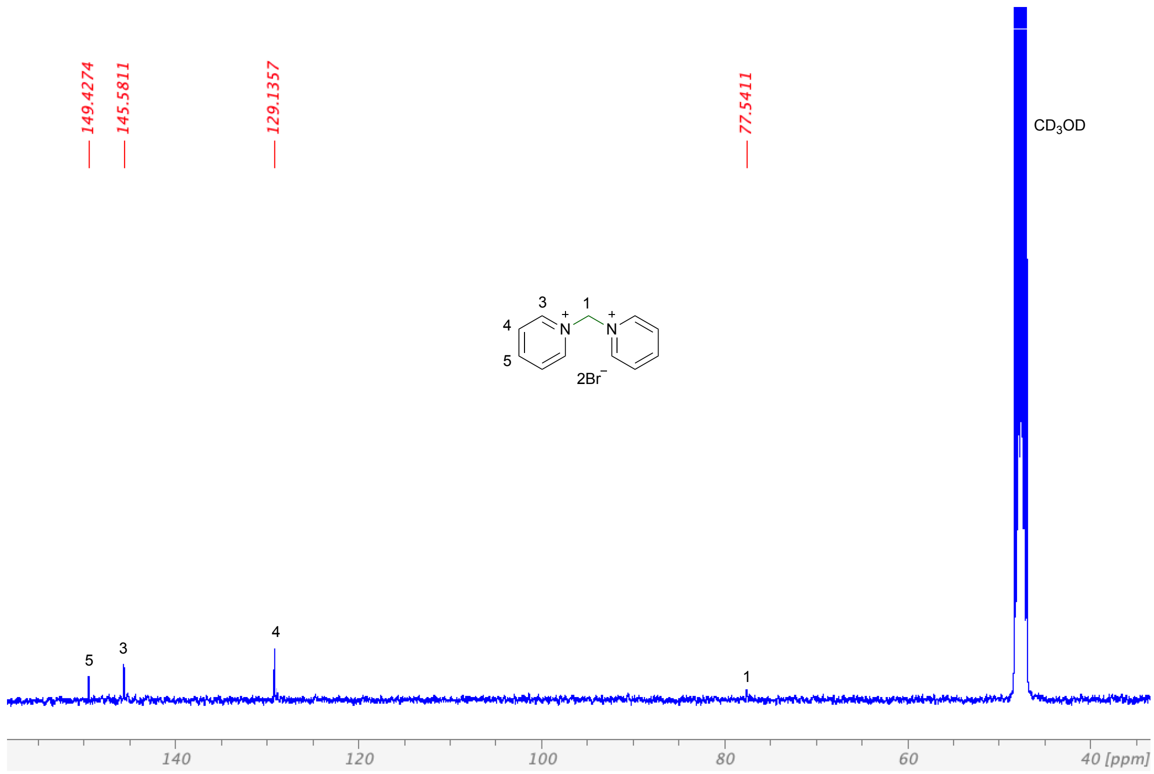

3.5.6 ^{1}H NMR (500 MHz, CD_3OD) spectrum of **1-I**

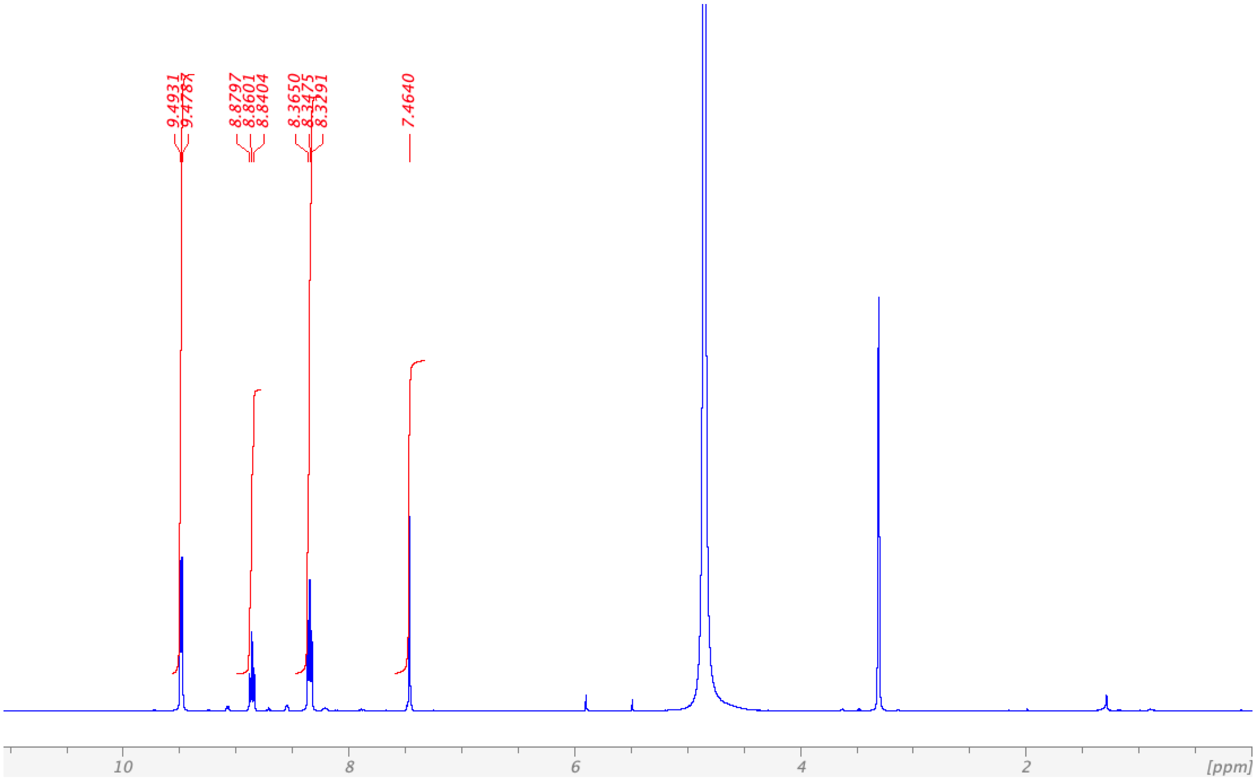

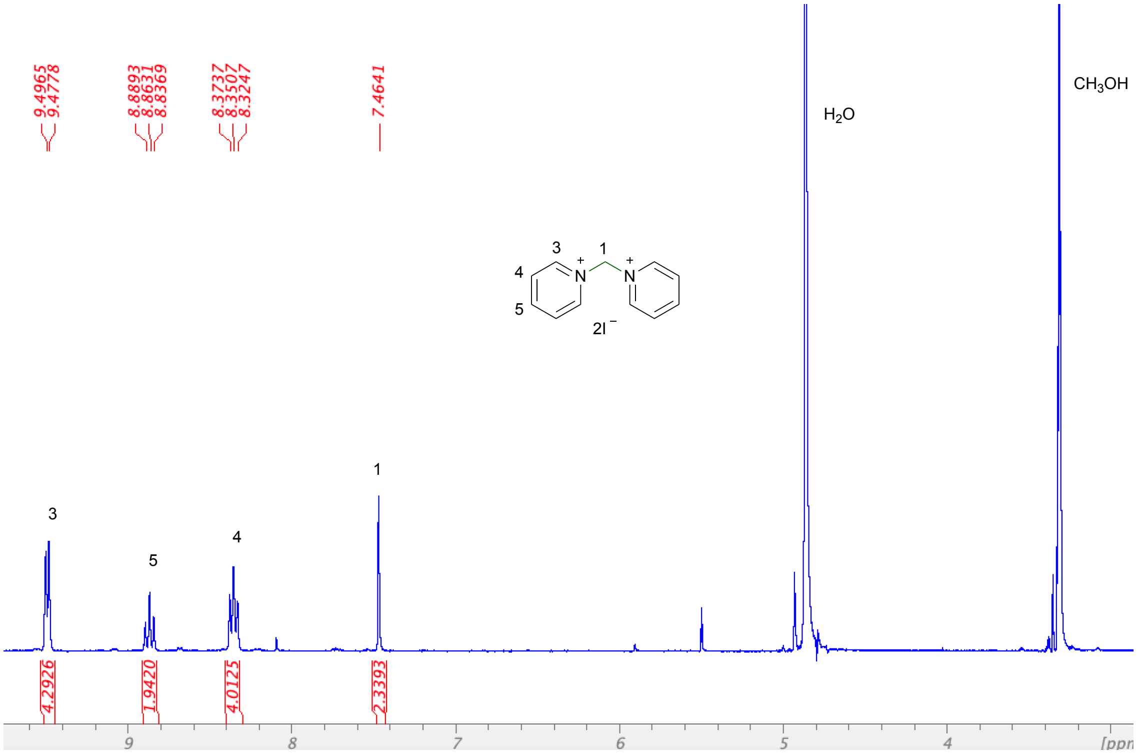

3.5.7 ^{13}C NMR (125 MHz, $(CD_3)_2SO$) spectrum of **1-I**

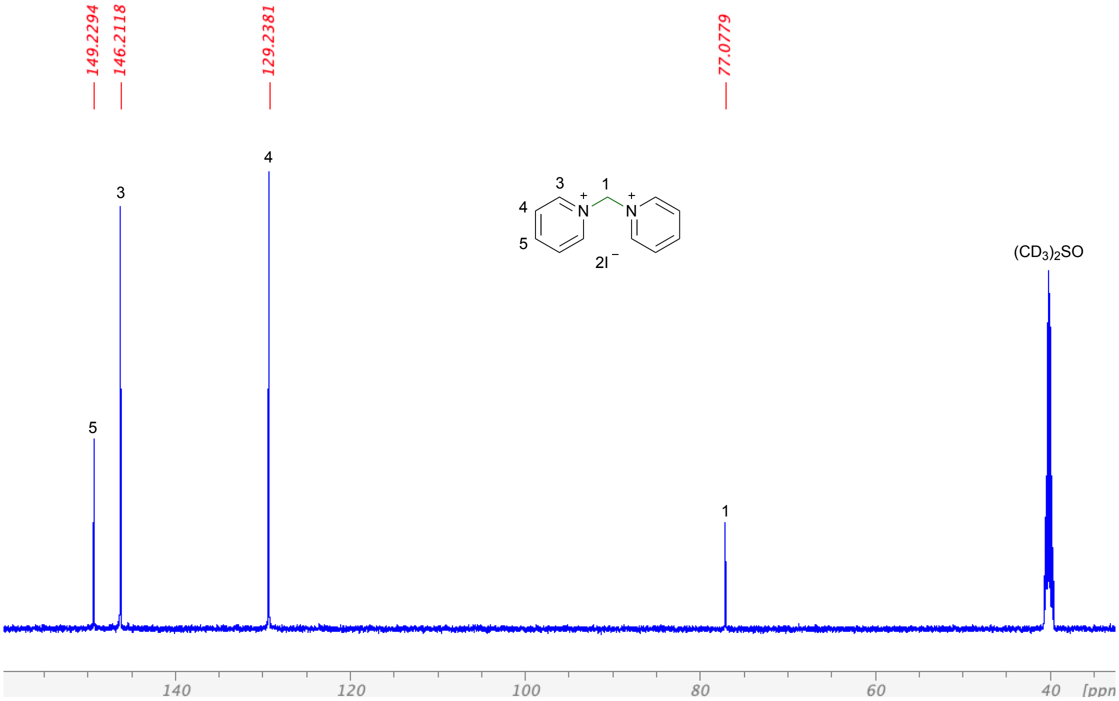

3.5.8 ^{1}H NMR (500 MHz, CD_3OD) spectrum of **2-Cl**

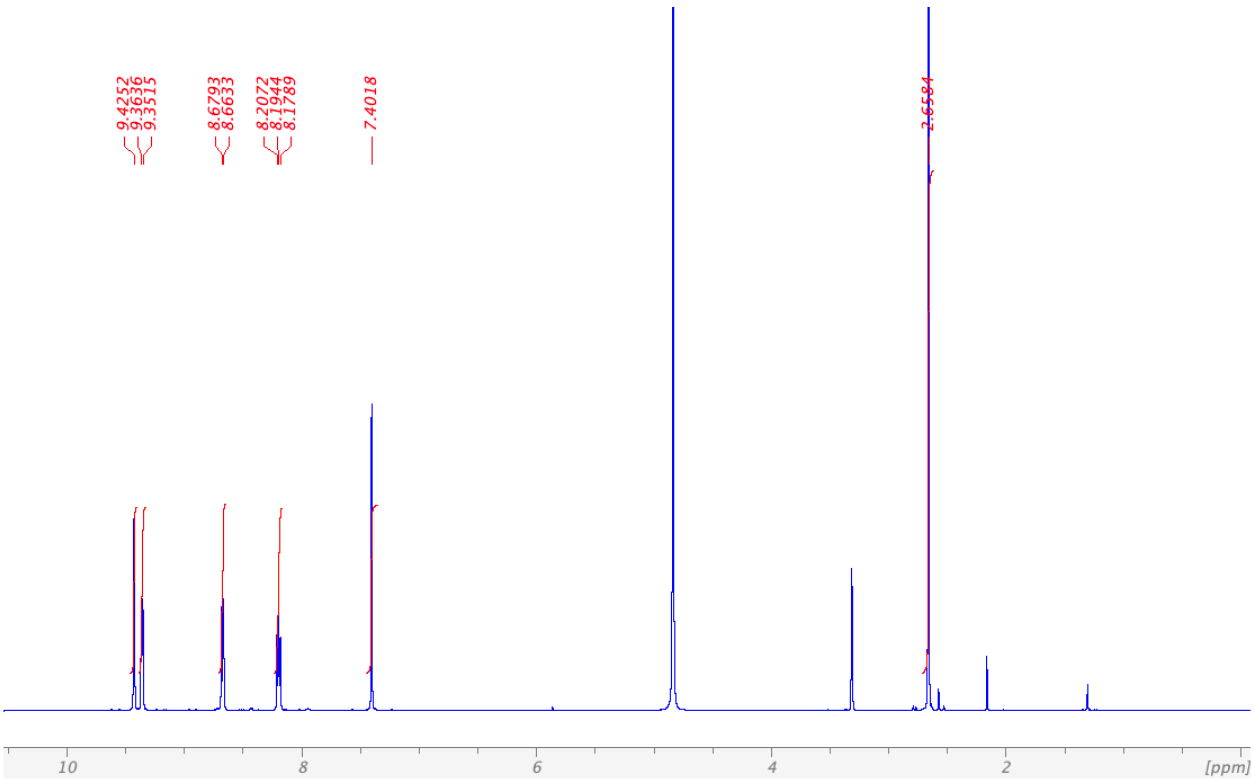

9.4252
9.3636
9.3515
8.6793
8.6633
8.2072
8.1944
8.1789
7.4018
2.6584
10
8
6
4
2
[ppm]

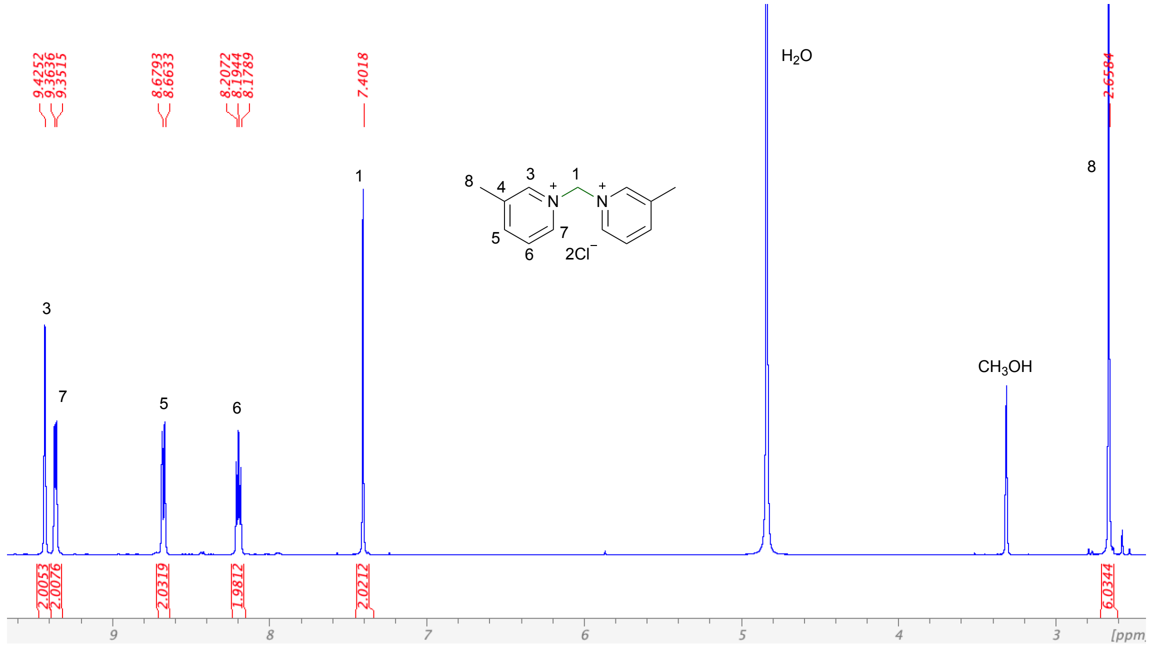

9.4252
9.3636
9.3515
8.6793
8.6633
8.2072
8.1944
8.1789
7.4018
2.6584
H_2O
CH_3OH
3
7
5
6
1
8
4
2Cl⁻
2.0053
2.0076
2.0319
1.9812
2.0212
6.0344
9
8
7
6
5
4
3
[ppm]

3.5.9 ^{1}H-^{1}H COSY NMR (500 MHz, CD_3OD) spectrum of **2-Cl**

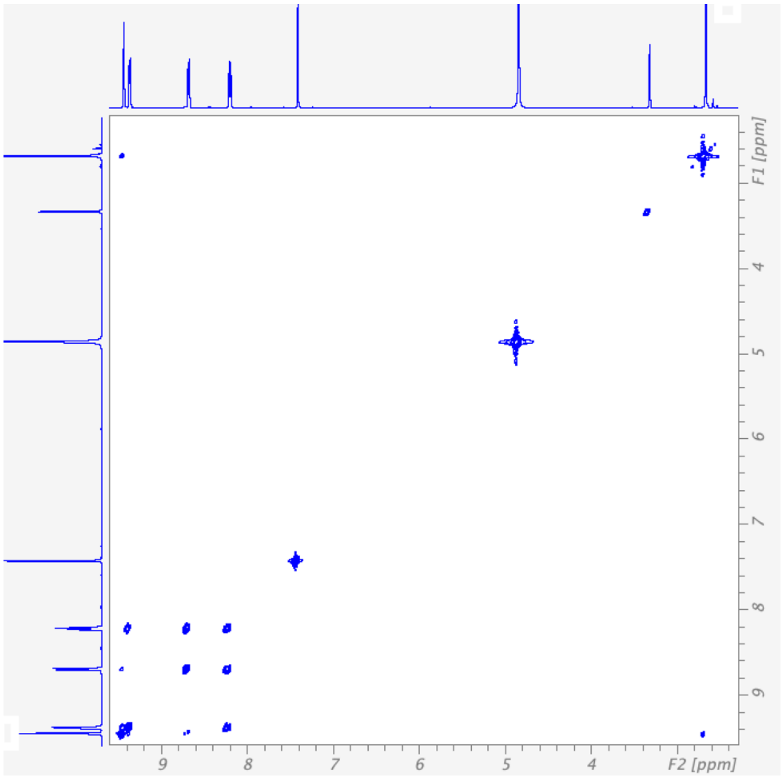

3.5.10 HMQC NMR (500 MHz, CD_3OD) spectrum of **2-Cl**

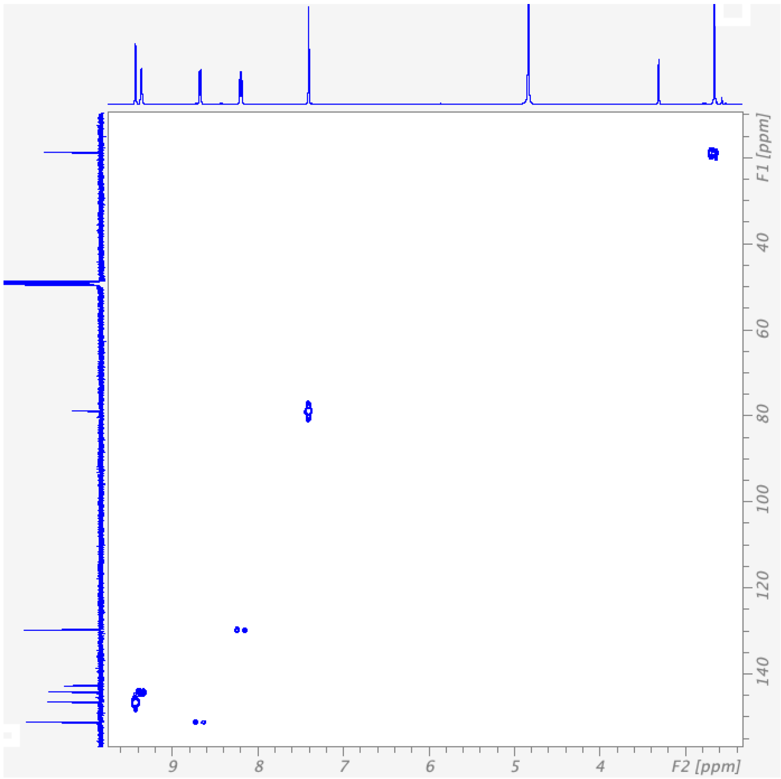

3.5.11 ^{13}C NMR (125 MHz, CD_3OD) spectrum of **2-Cl**

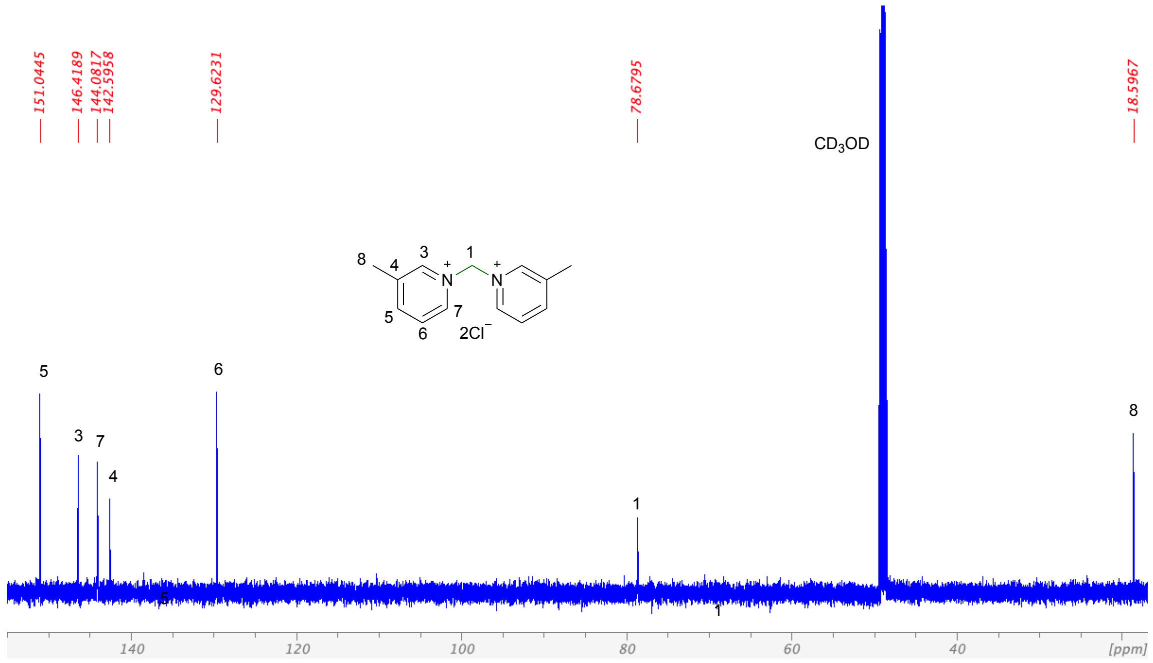

3.5.12 ^1H NMR (500 MHz, CD_3OD) spectrum of **2-Br**

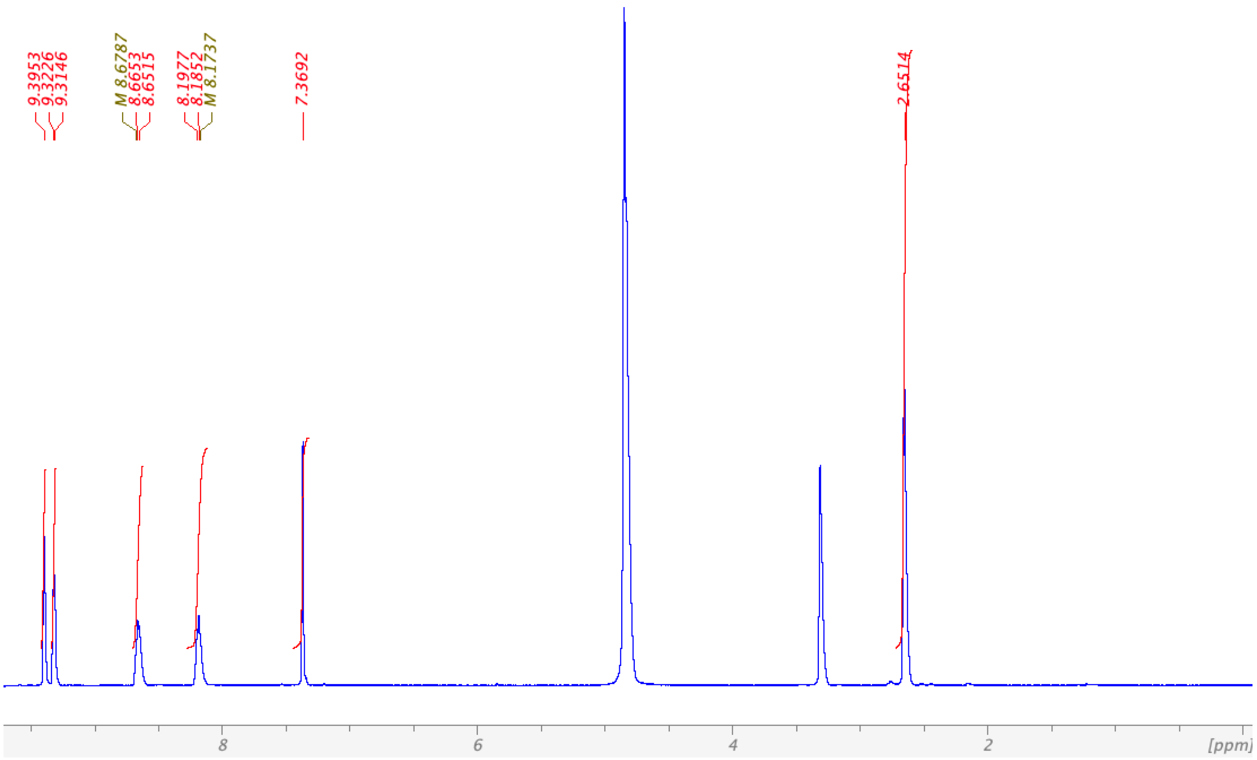

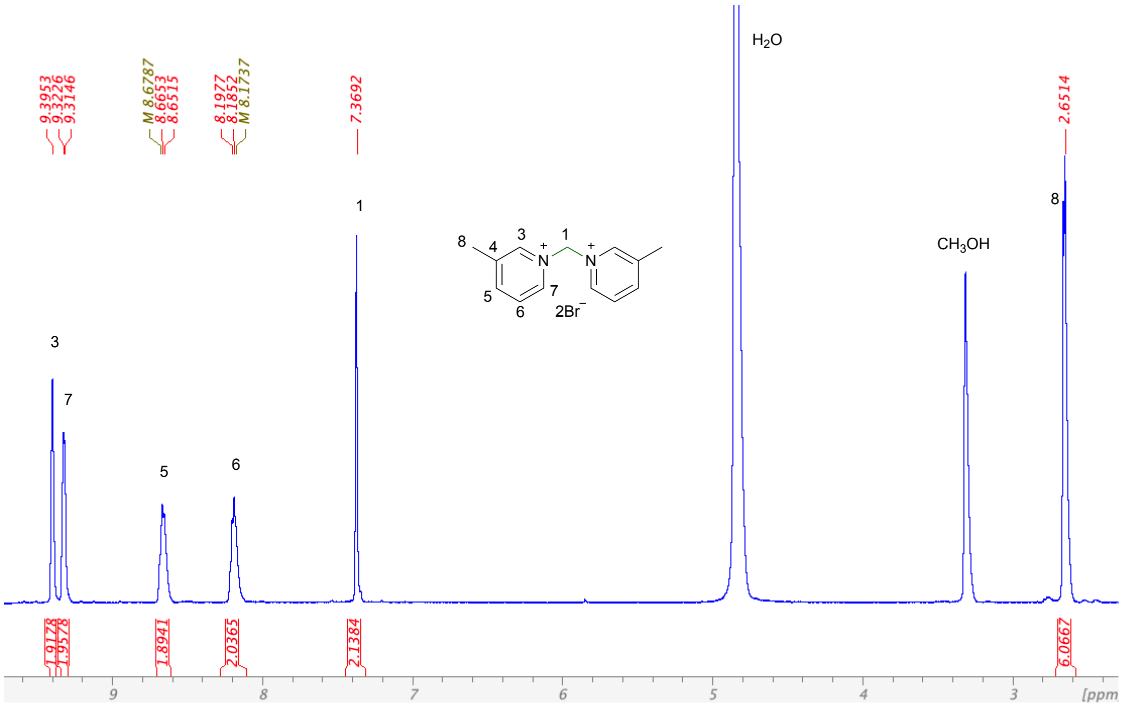

3.5.13 ^{1}H-^{1}H COSY NMR (500 MHz, CD_3OD) spectrum of **2-Br**

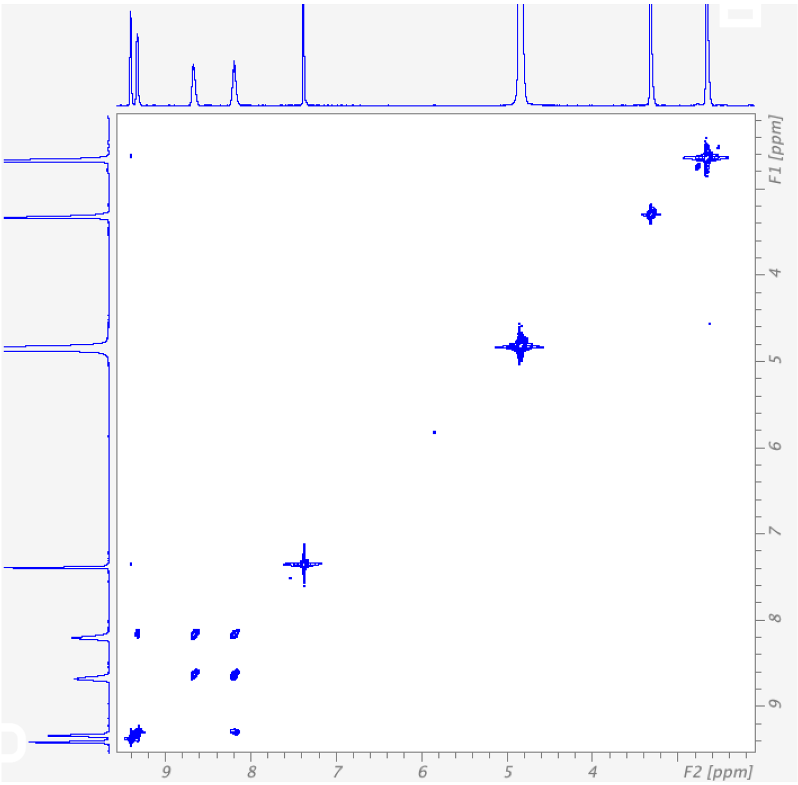

3.5.14 HMQC NMR (500 MHz, CD_3OD) spectrum of **2-Br**

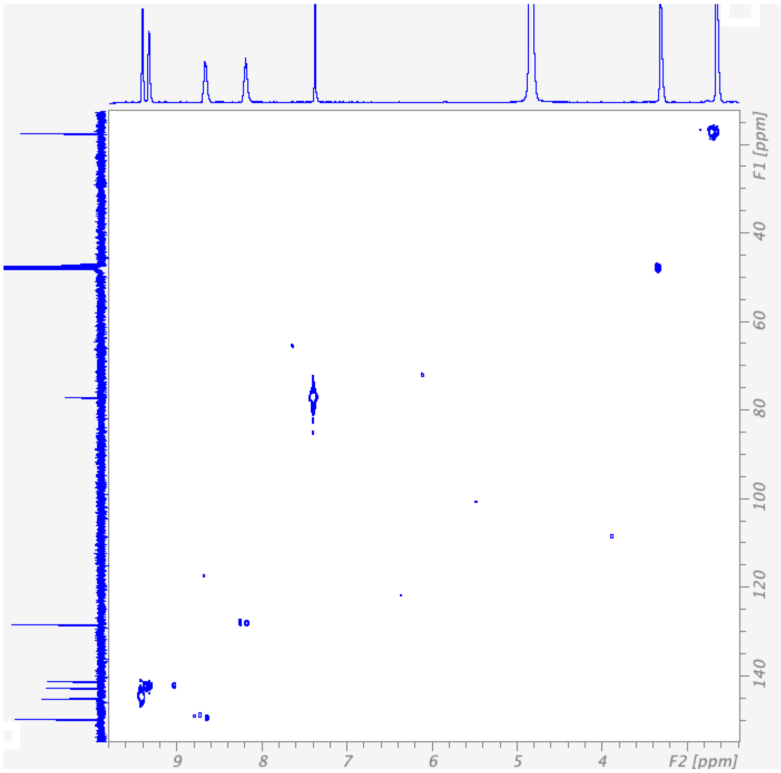

3.5.15 ^{13}C NMR (125 MHz, CD_3OD) spectrum of **2-Br**

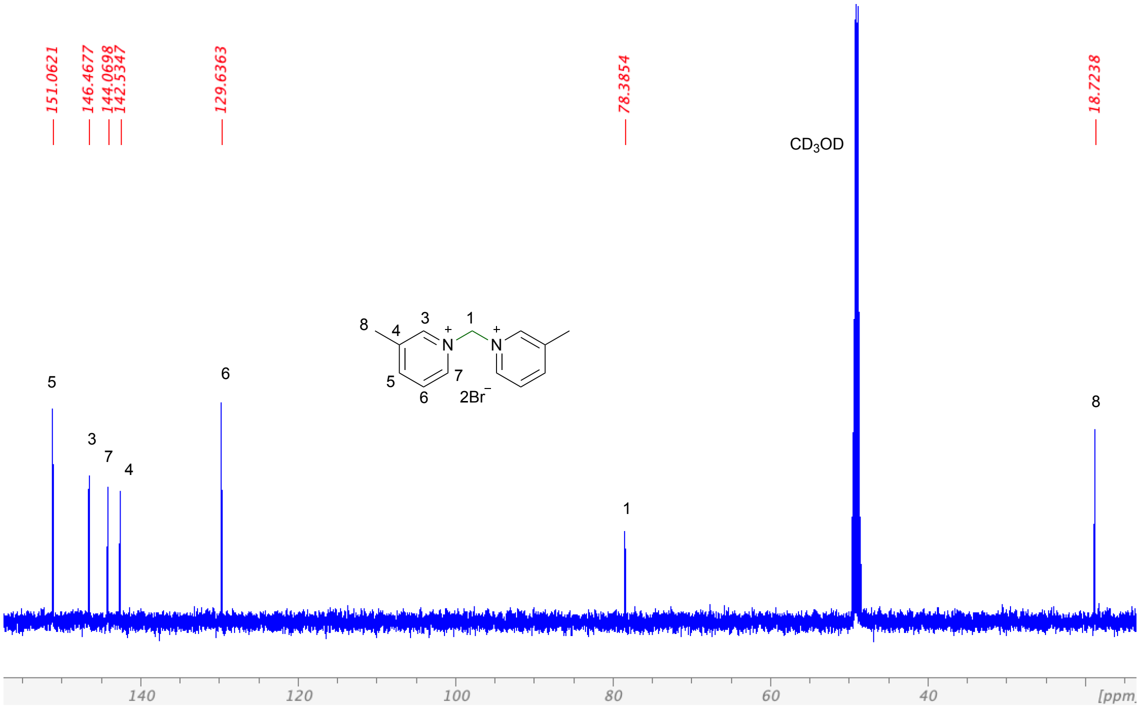

3.5.16 ^{1}H NMR (500 MHz, CD_3OD) spectrum of **2-I**

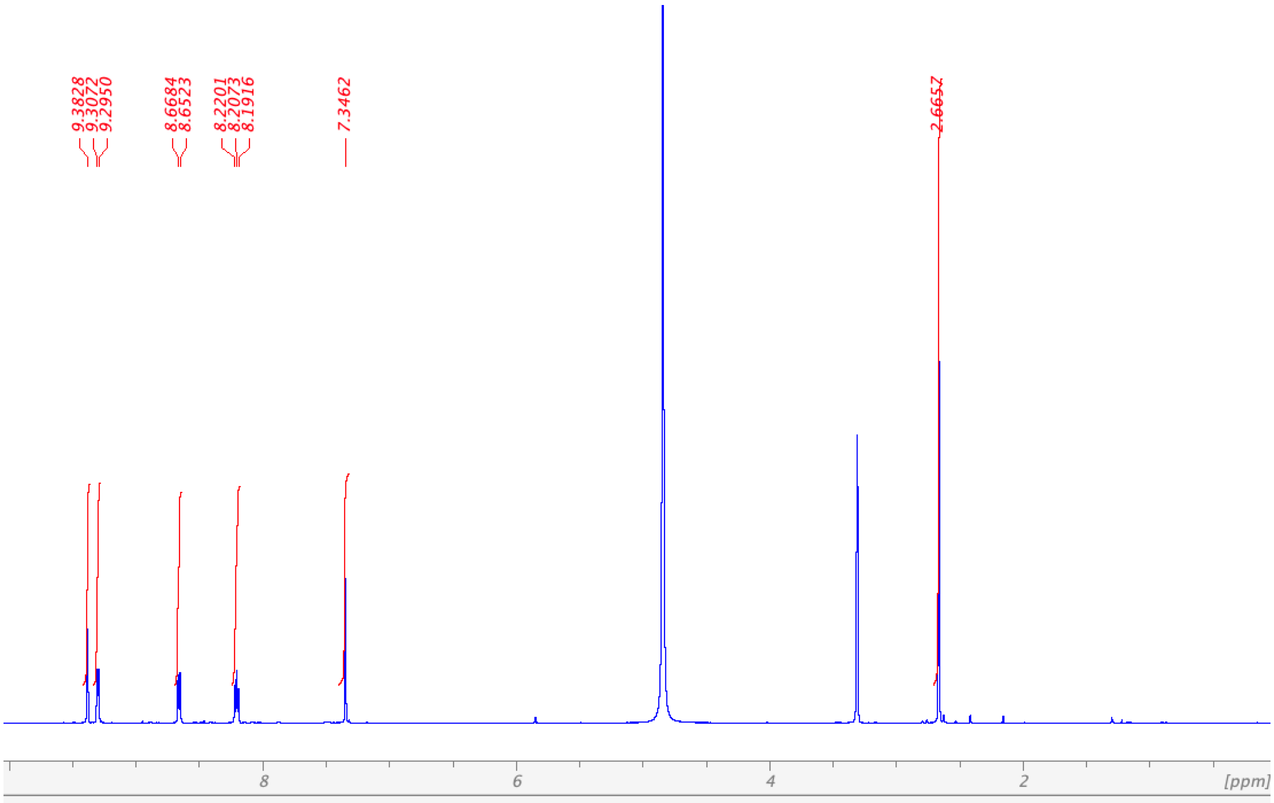

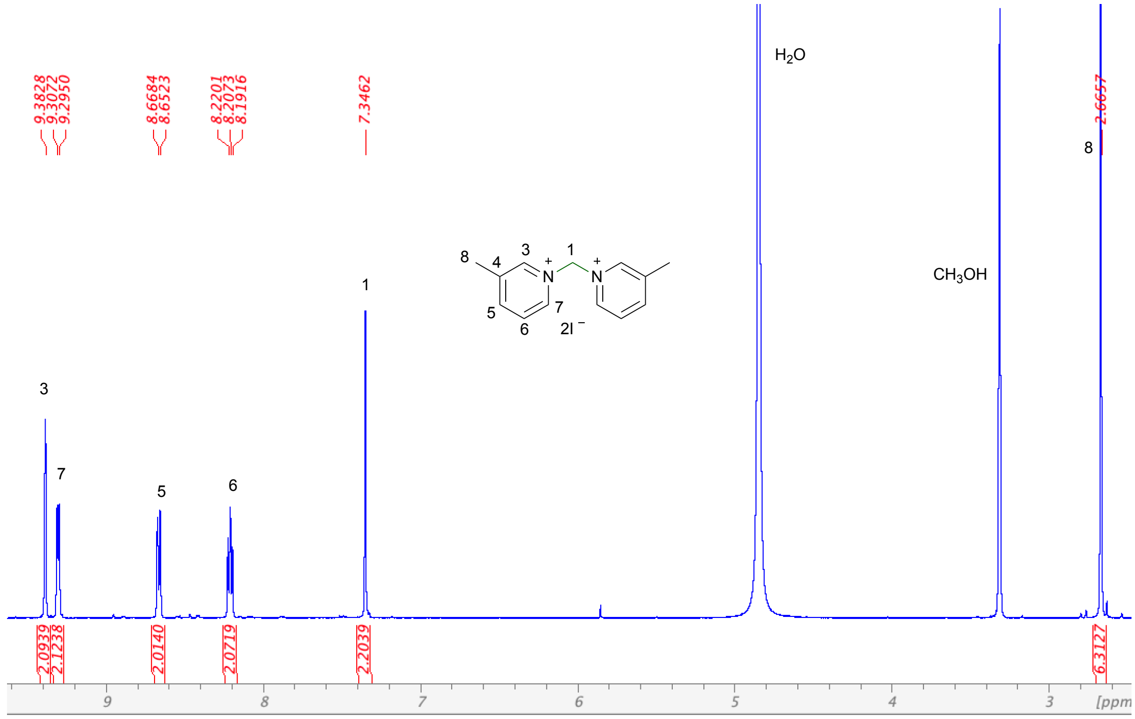

3.5.17 ^{1}H-^{1}H COSY NMR (500 MHz, CD_3OD) spectrum of **2-I**

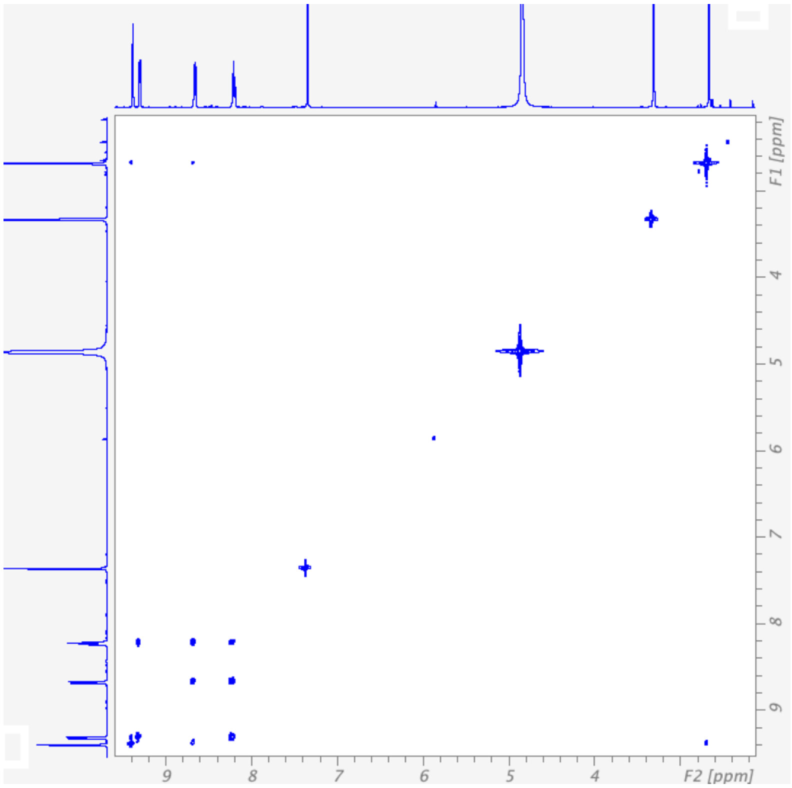

3.5.18 HMQC NMR (500 MHz, CD_3OD) spectrum of **2-I**

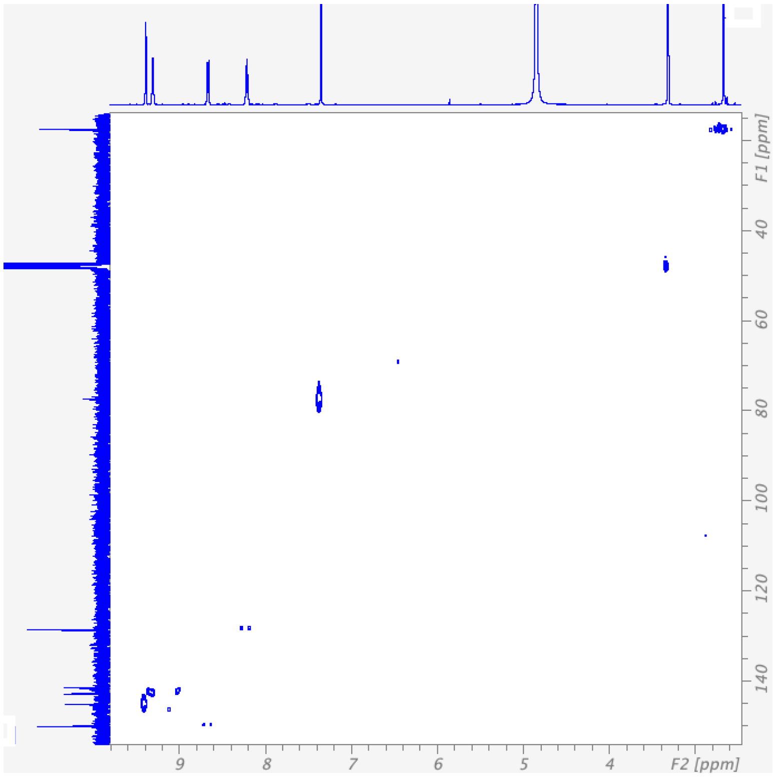

3.5.19 ^{13}C NMR (125 MHz, CD_3OD) spectrum of **2-I**

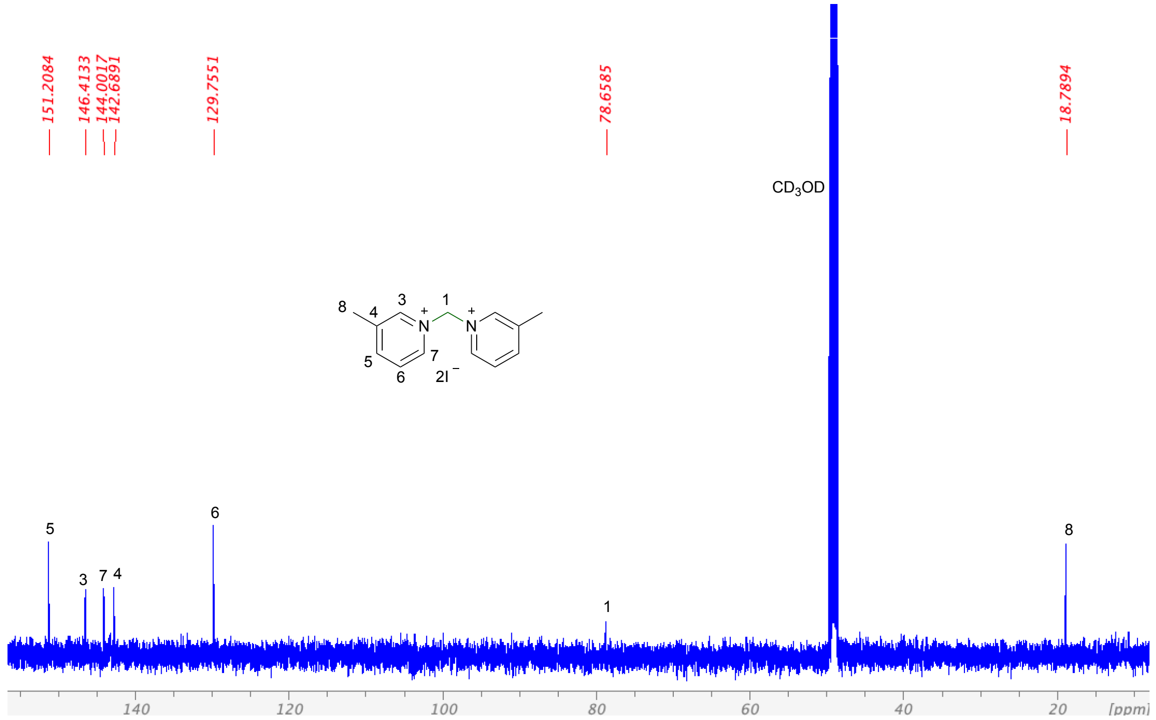

3.5.20 ^{1}H NMR (500 MHz, CD_3OD) spectrum of **3-Cl**

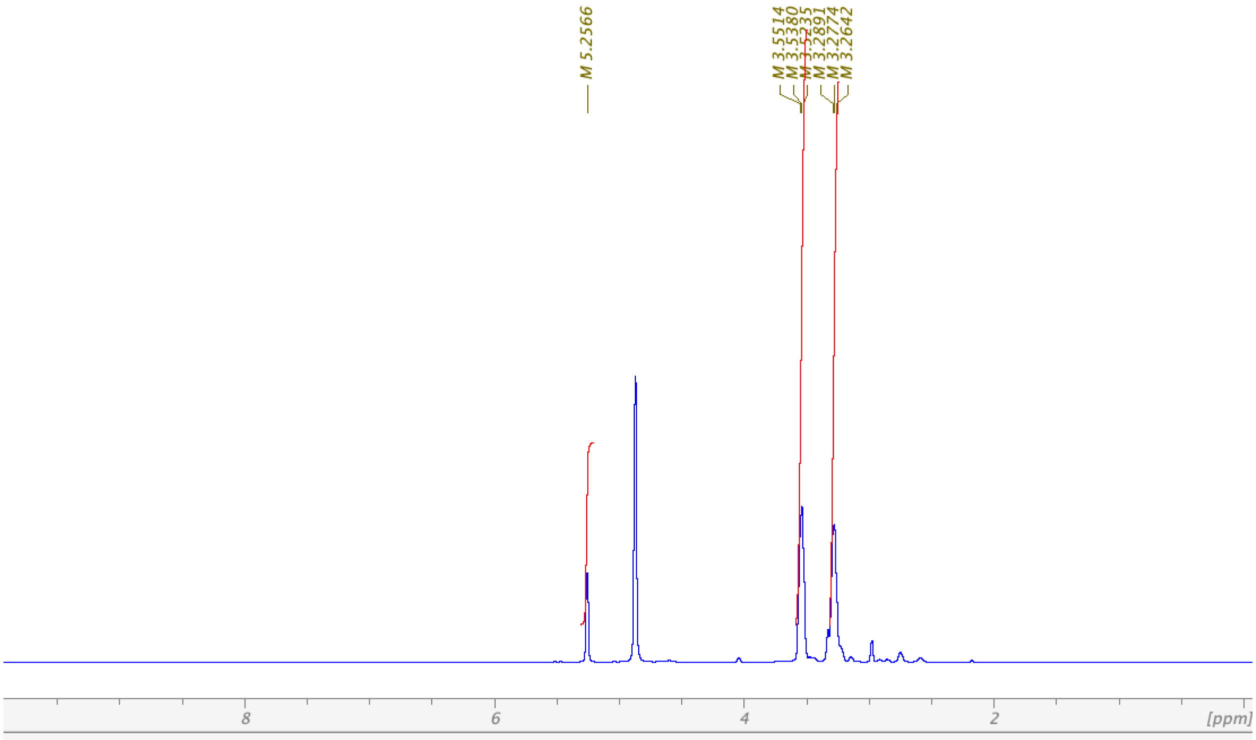

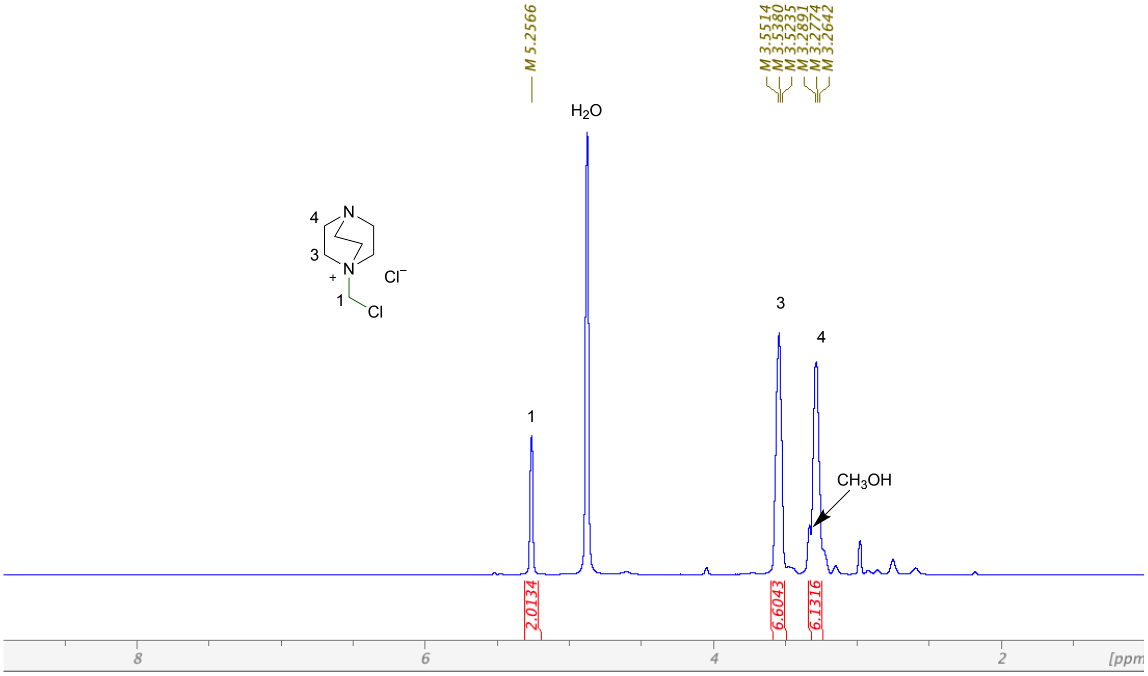

3.5.21 ^{1}H-^{1}H COSY NMR (400 MHz, CD_3OD) spectrum of **3-Cl**

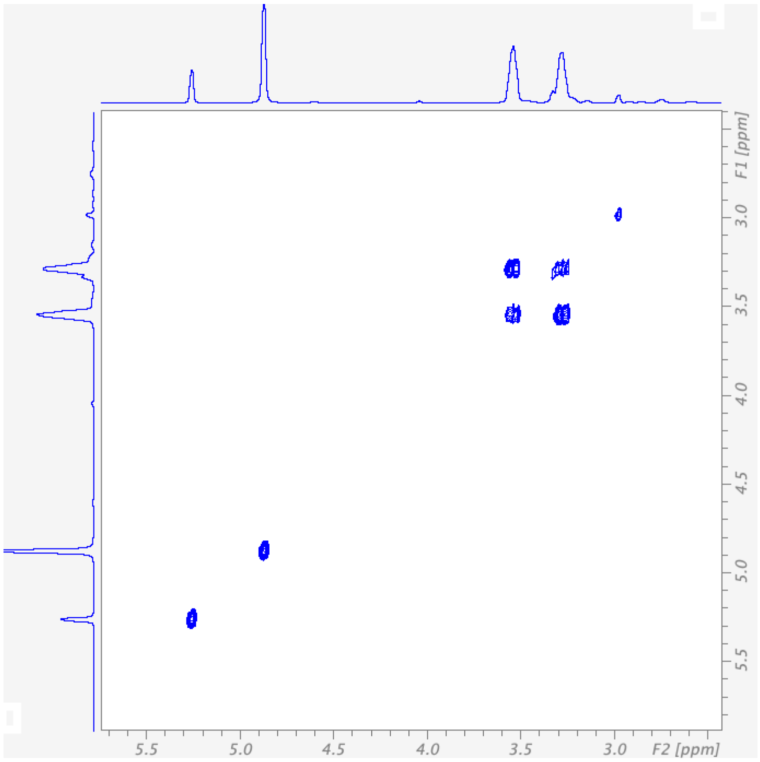

3.5.22 HSQC NMR (400 MHz, CD_3OD) spectrum of **3-Cl**

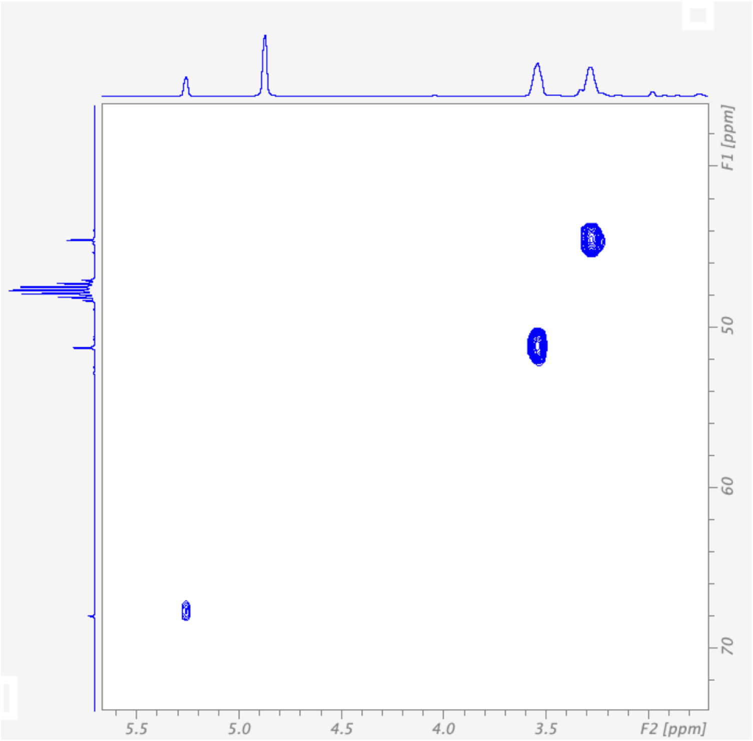

3.5.23 ^{13}C NMR (100 MHz, CD_3OD) spectrum of **3-Cl**

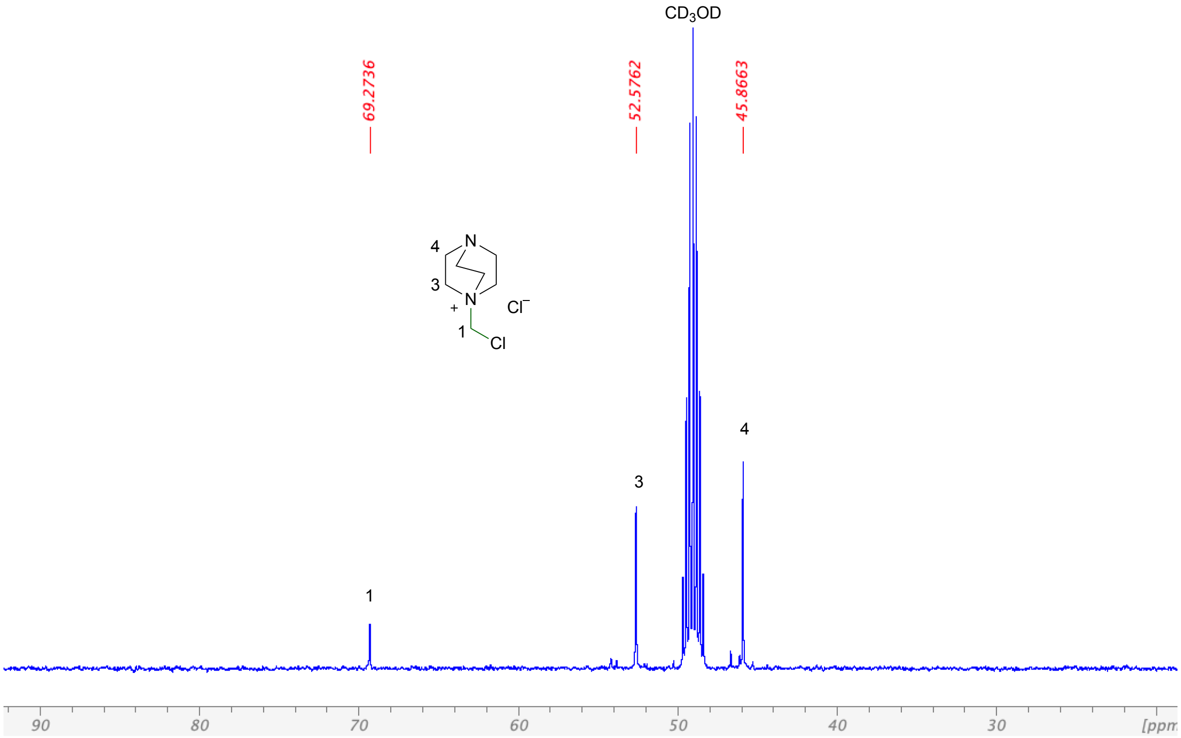

3.5.24 ^{1}H NMR (500 MHz, CD_3OD) spectrum of **3-Br**

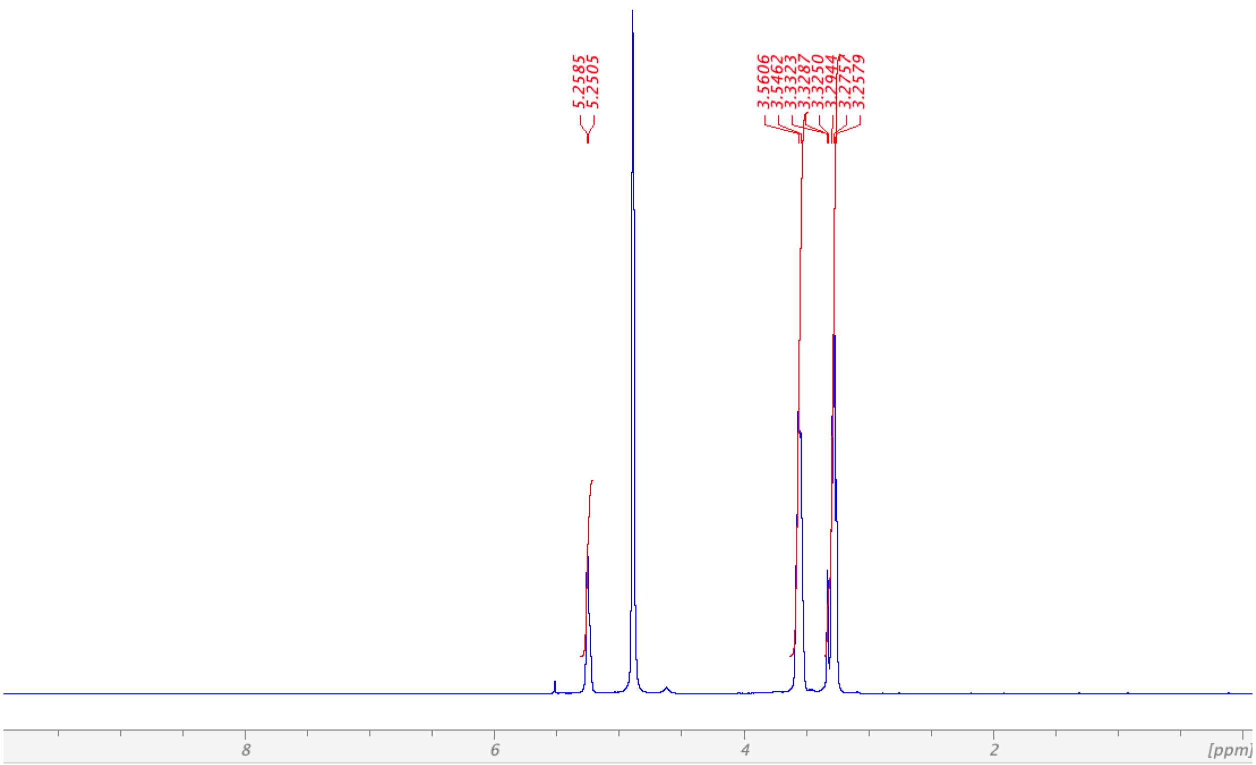

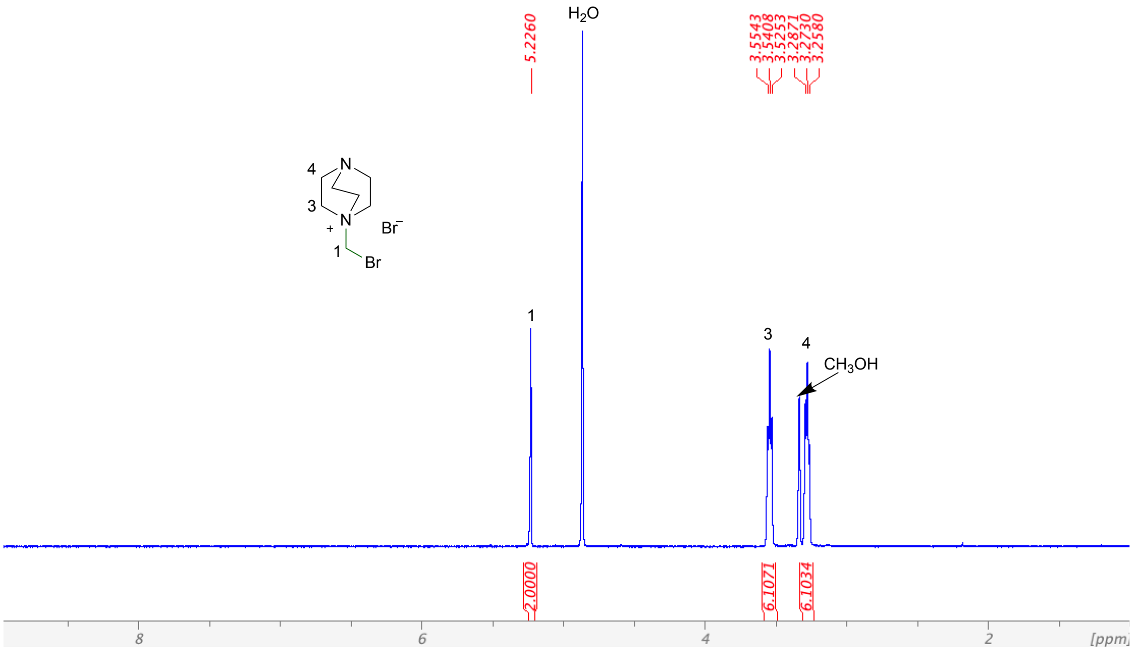

3.5.25 ^{1}H-^{1}H COSY NMR (500 MHz, CD_3OD) spectrum of **3-Br**

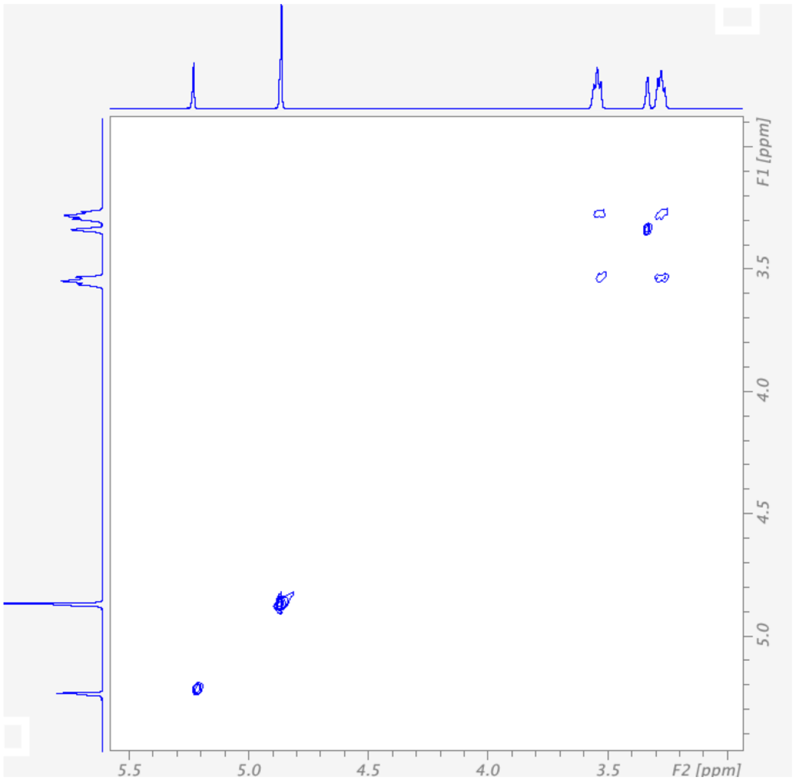

3.5.26 HMQC NMR (500 MHz, CD_3OD) spectrum of **3-Br**

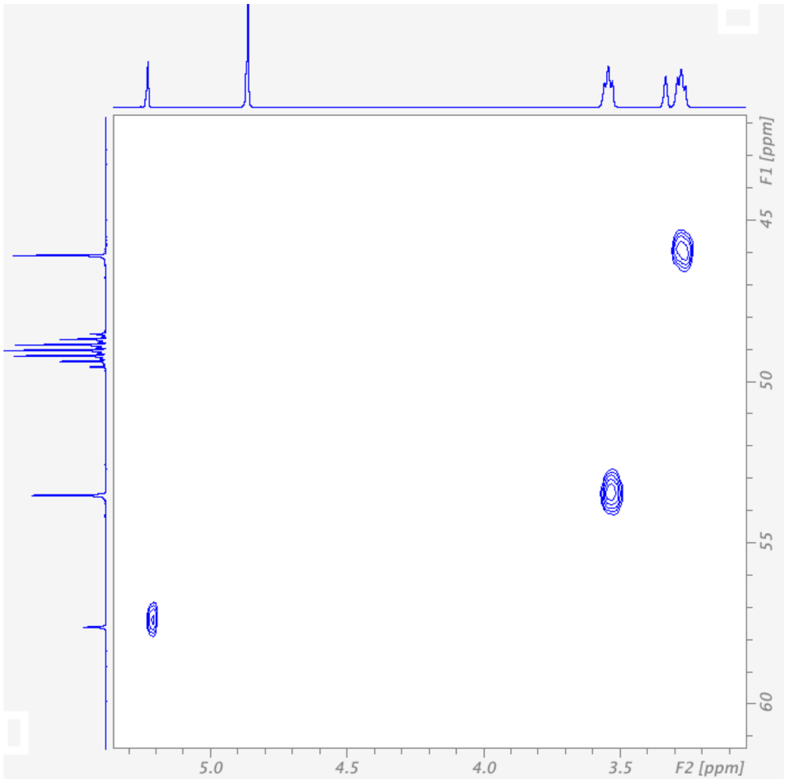

3.5.27 ^{13}C NMR (125 MHz, CD_3OD) spectrum of **3-Br**

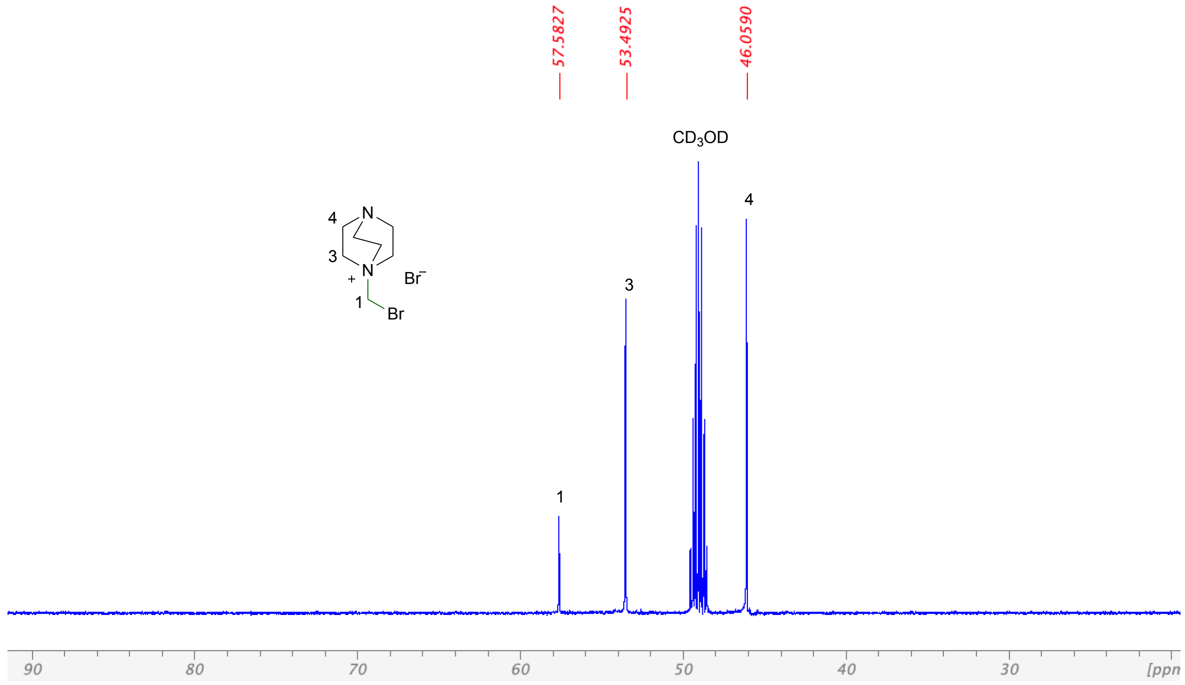

3.5.28 ^{1}H NMR (500 MHz, CD_3OD) spectrum of **3-I**

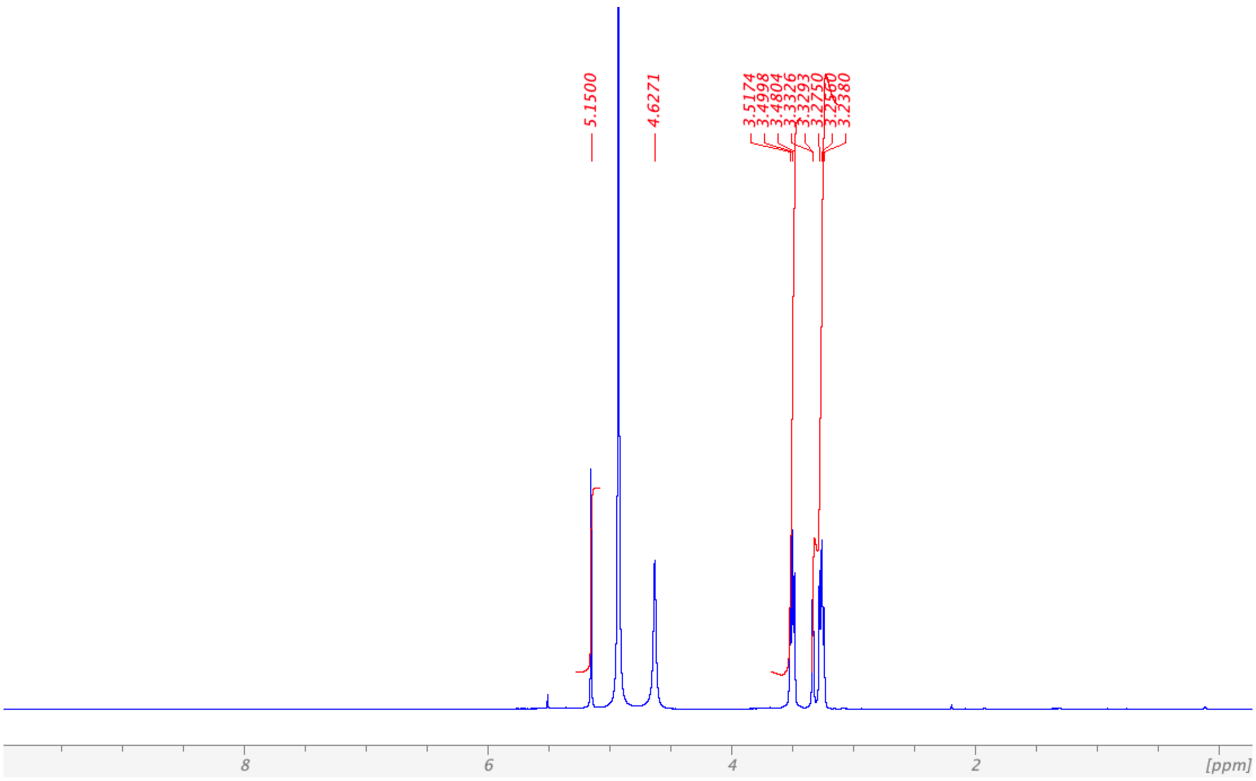

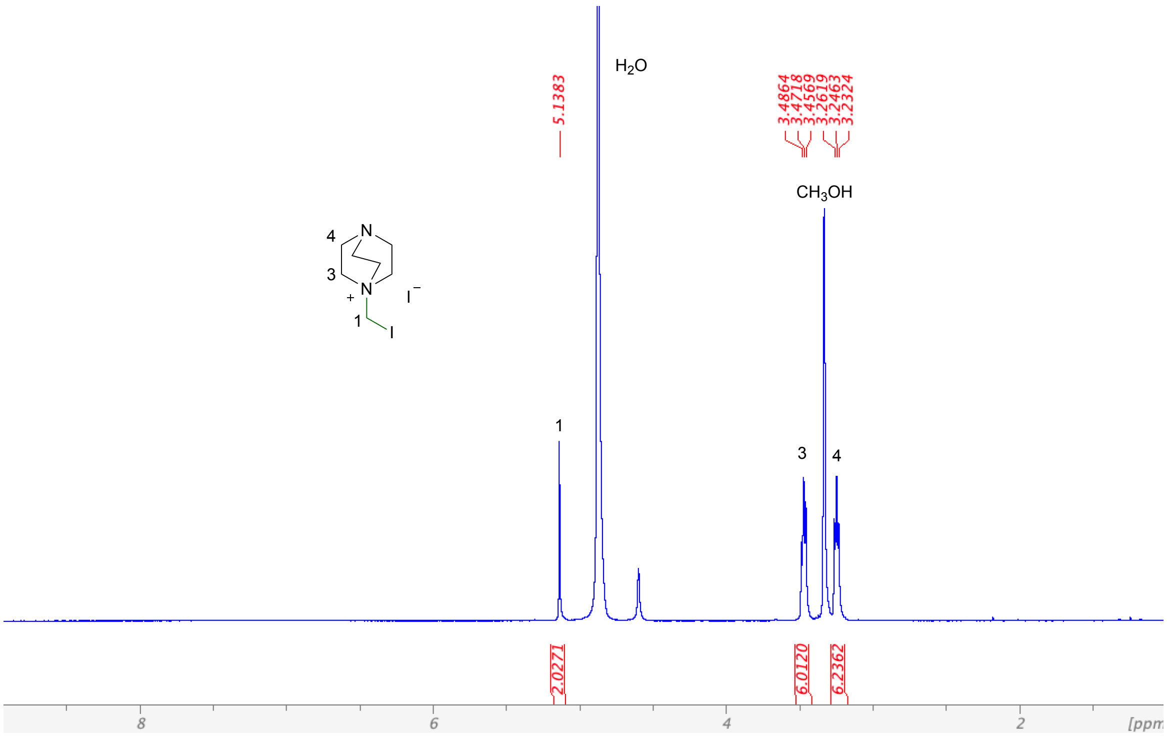

3.5.29 ^{1}H-^{1}H COSY NMR (500 MHz, CD_3OD) spectrum of **3-I**

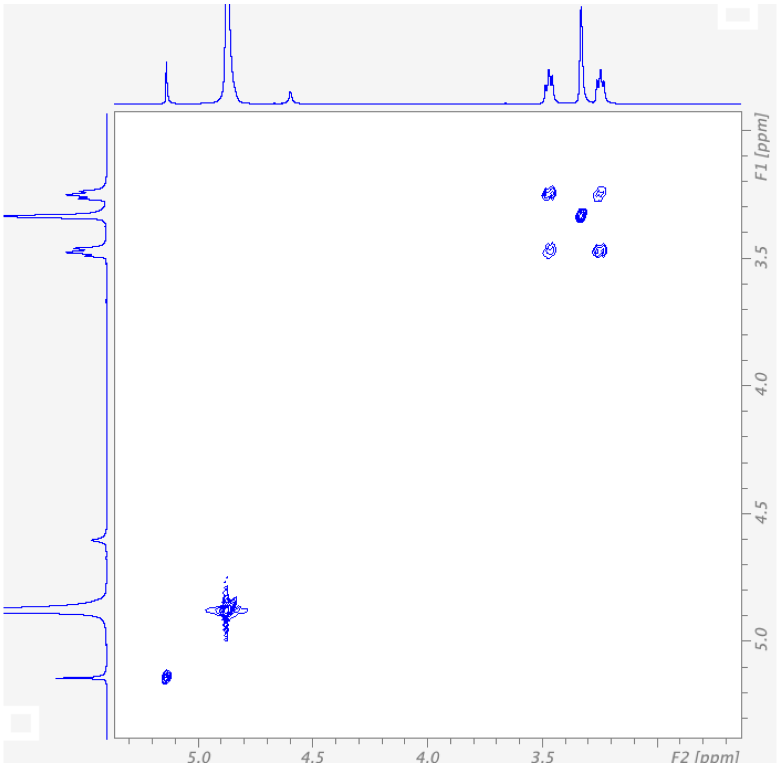

3.5.30 HMQC NMR (500 MHz, CD_3OD) spectrum of **3-I**

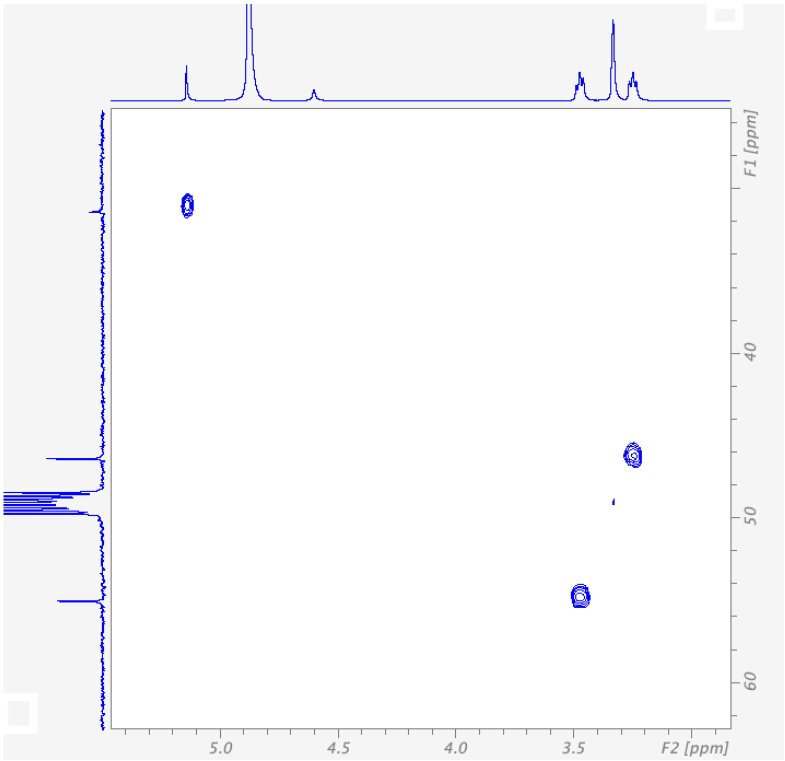

3.5.31 ^{13}C NMR (125 MHz, CD_3OD) spectrum of **3-I**

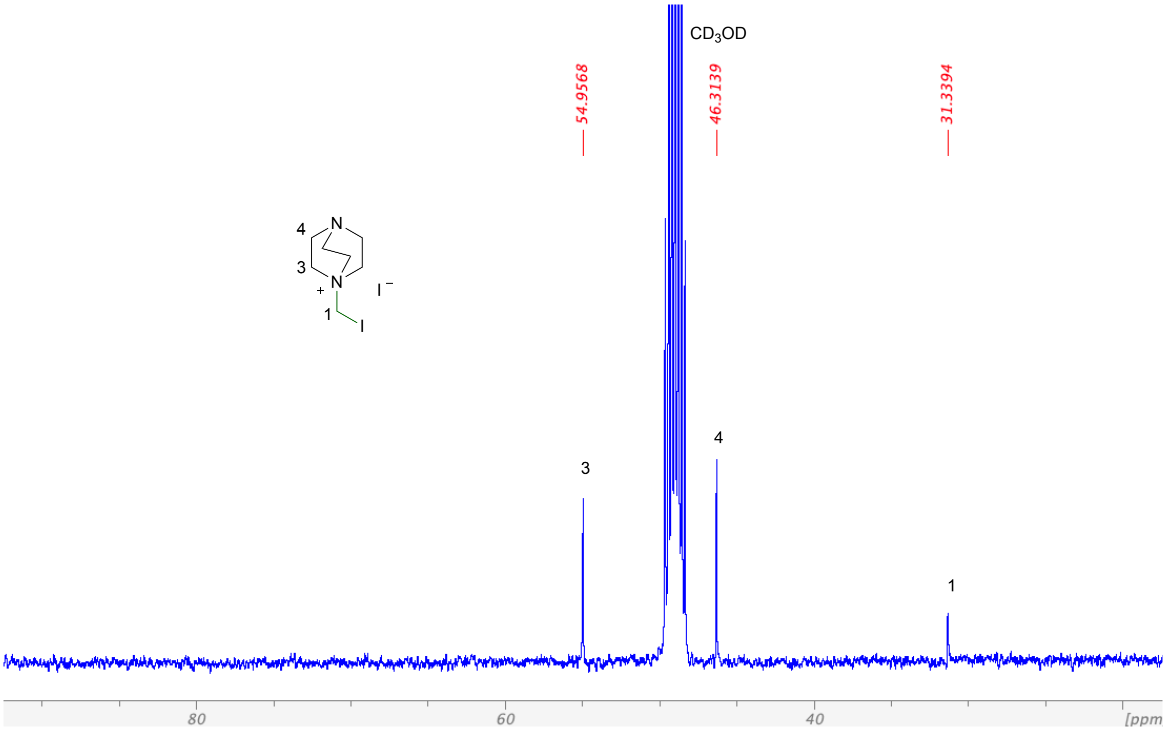

4 Theoretical Study

The electronic structure and geometries of all compounds were calculated using density functional theory (DFT) at the **B3LYP** level,[22,23] with the **6-311+G**** basis set for all atoms. Molecular geometries of all model complexes were optimized without symmetry constraints. Frequency calculations were performed at the same level of theory to identify all of the stationary points as either **transition states** (one imaginary frequency) or **minima** (zero imaginary frequencies) and to provide the thermal correction to free energies at **298.15 K** and **1 atm**. In some cases, a structure was considered a minimum despite a very low imaginary frequency (< 10 cm^{-1}), possibly due to the use of an insufficiently large integration grid.[24,25]

For certain cases, solution-phase **SCF** energies for intermediates and transition states were obtained via **single point calculation** on the gas-phase optimized structure using the **PCM solvation model** in toluene or THF.[26]In these cases, the **Gibbs free energies in solution** were estimated using the equation $G_{solv} = E_{solv} + (G_{gas} - E_{gas})$. Including solvent effects in DFT calculations using the **PCM model** for various reaction mechanisms investigated for us and others did not significantly alter the energetic reaction profile (1–3 $kcal\cdot mol^{-1}$ deviations between gas-phase and solvent-phase free energies).[27–29] Therefore, most calculations were performed in the gas phase, and an in-solution description was not attempted. The energy profiles are presented in terms of **relative free energies** derived from thermochemical analysis. DFT calculations were carry out using the Gaussian 09 software suite.[30] The coordinates of all optimized compounds and other energy profiles are reported in the Table S14.

4.1 Index of figures and schemes corresponding to the mechanism described in the main manuscript:

Figure S26. Comparison of selected structural bond lengths (Å) for the three isomers of the bis(indazolyl)methane compound, **L1-L3** (experimental values in red, italics).

Figure S27. Relative energy profiles for the isomerization of **1*H*-indazole** to **2*H*-indazole** in the presence of base (**py**, **PMP** and **NMeCy₂**) and in the absence of a base (with the highest $\mathbf{TS_{Ind}}$ barrier at 50.0 $kcal\cdot mol^{-1}$).

Figure S28. Comparison of the relative energy profiles for the formation of the intermediates **1-bromomethylindazole** and **2-bromometilindazole** from the reaction of CH_2Br_2 with **1*H*-indazole** in the presence of **PMP**.

Figure S29. Relative energy profile for the formation of **L1** from the reaction of CH_2Br_2 with **1*H*-indazole** in the presence of **pyridine**.

Figure S30. Relative energy profile for the isomerization process toward compound **L1** from the isomers **L2** and **L3** in the absence of **[Hpy]$^+$ cation**.

Scheme S3. Mechanism for the formation of **L2** compound.

Scheme S4. Mechanism for the formation of **L1** compound.

Scheme S5. Mechanism for the formation of **L3** compound.

Table S14. Coordinates of the optimized compounds.

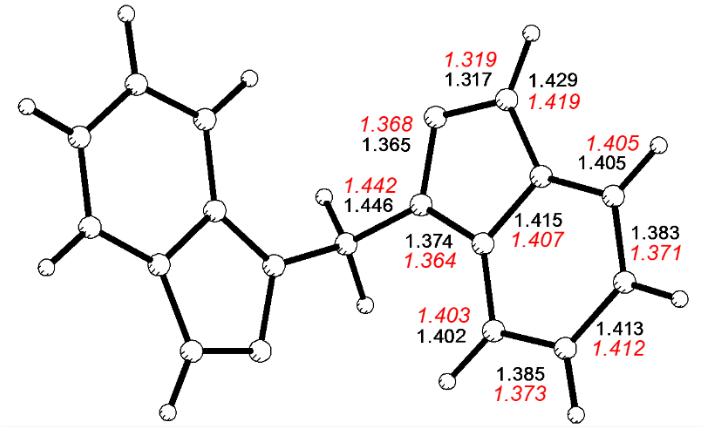

L1

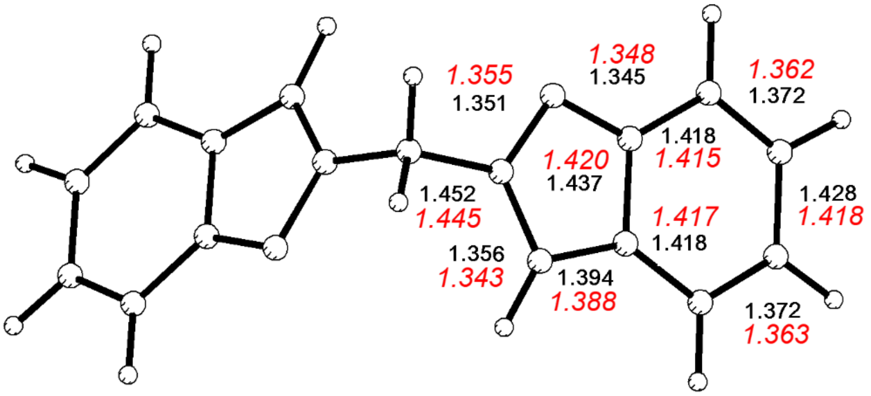

L2

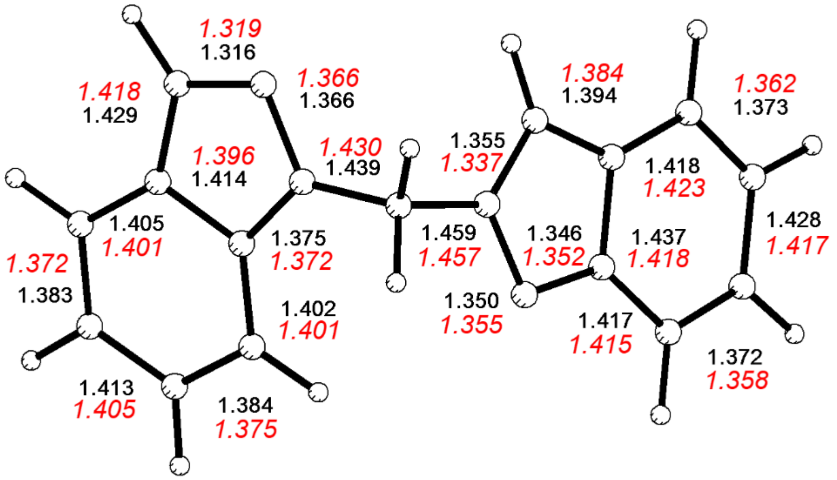

L3

Figure S26. Comparison of selected structural bond lengths (Å) for the three isomers of the bis(indazolyl)methane compound, **L1-L3** (experimental values in red, italics).

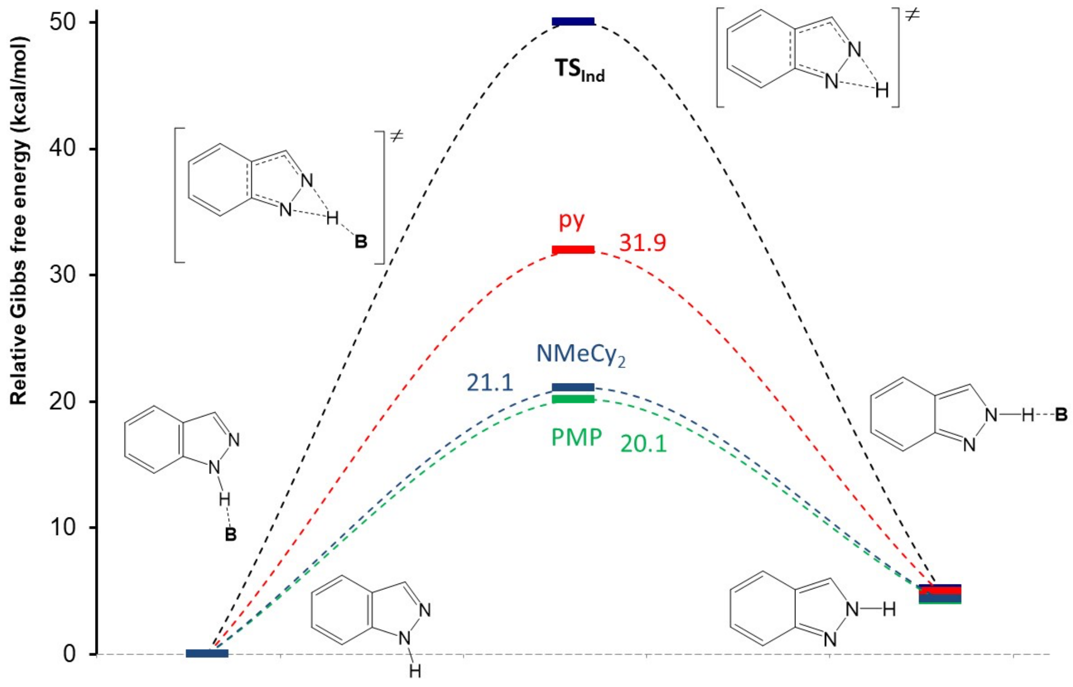

Figure S27. Relative energy profiles for the isomerization of **1*H*-indazole** to **2*H*-indazole** in the presence of base (**py**, **PMP** and **NMeCy₂**) and in the absence de base (with the highest **TS**$_{\mathbf{Ind}}$ barrier at 50.0 kcal·mol^{-1}).

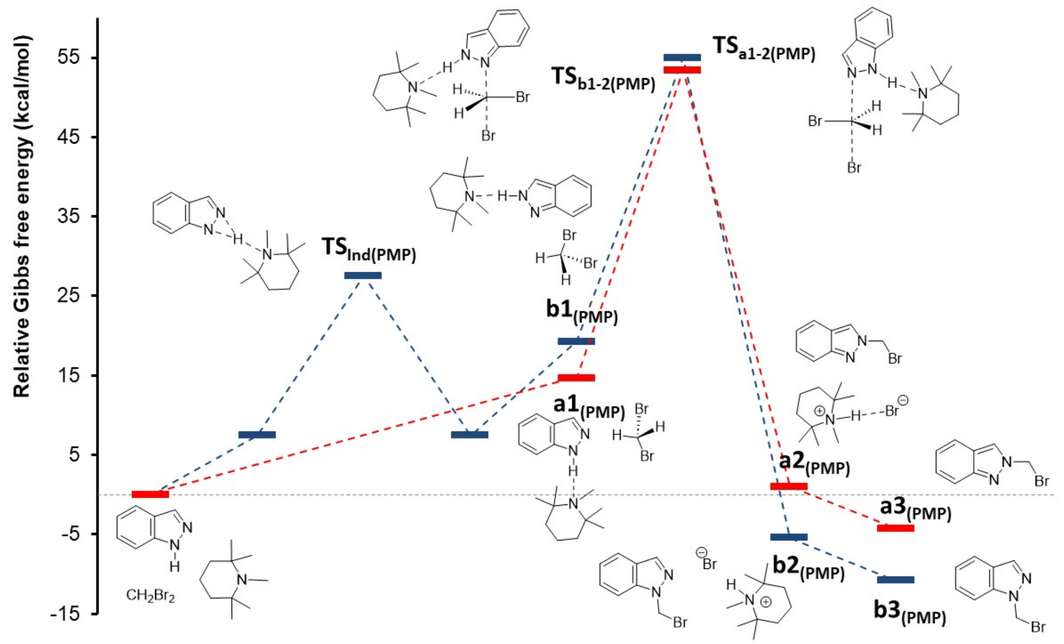

Figure S28. Comparison of the relative energy profiles for the formation of the intermediates **1-bromomethylindazole** and **2-bromomethylindazole** from the reaction of CH_2Br_2 with **1*H*-indazole** in the presence of **PMP**.

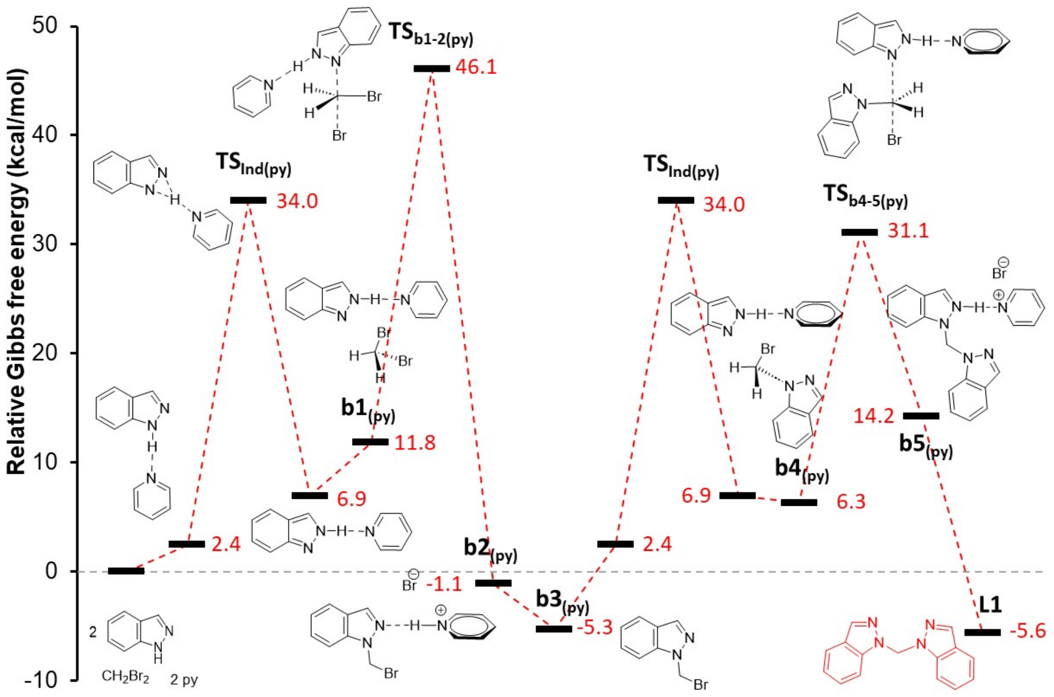

Figure S29. Relative energy profile for the formation of **L1** from the reaction of CH_2Br_2 with **1*H*-indazole** in the presence of **pyridine**.

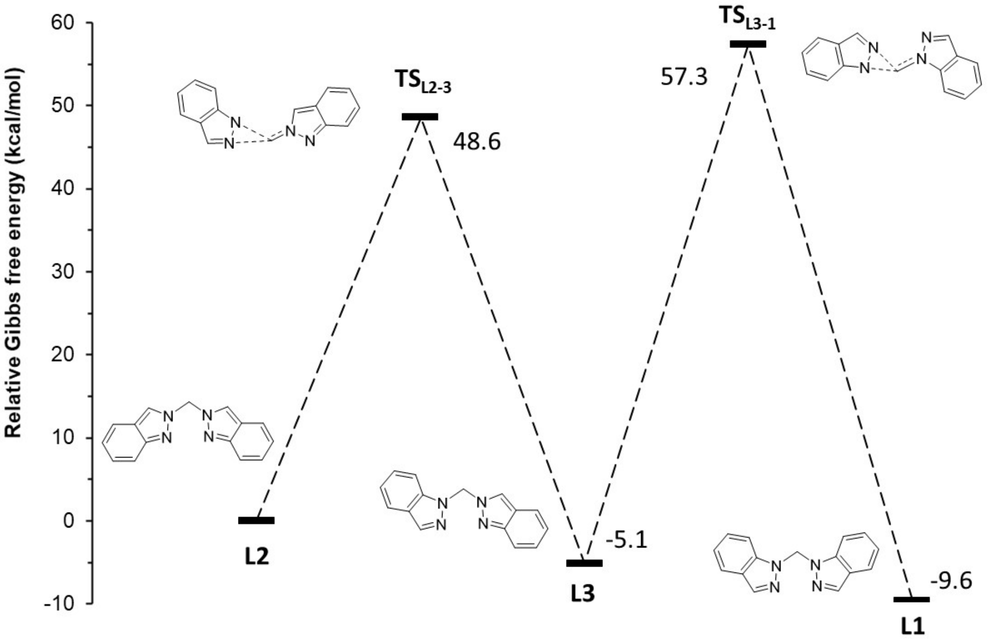

Figure S30. Relative energy profile for the isomerization process toward compound **L1** from the isomers **L2** and **L3** in the absence of **[Hpy]$^+$ cation**.

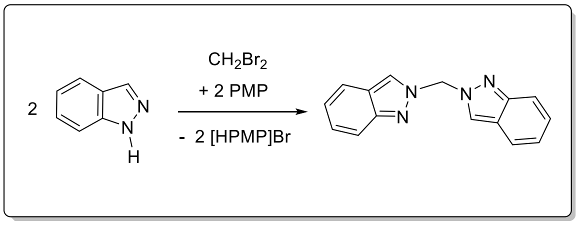

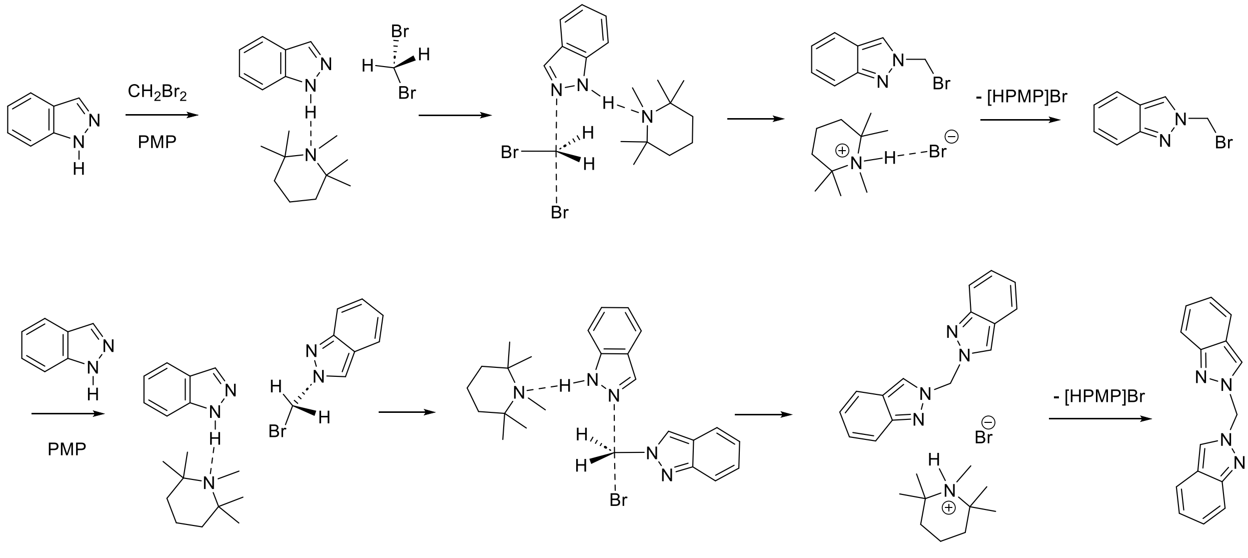

Scheme S3. Mechanism for the formation of **L2** compound.

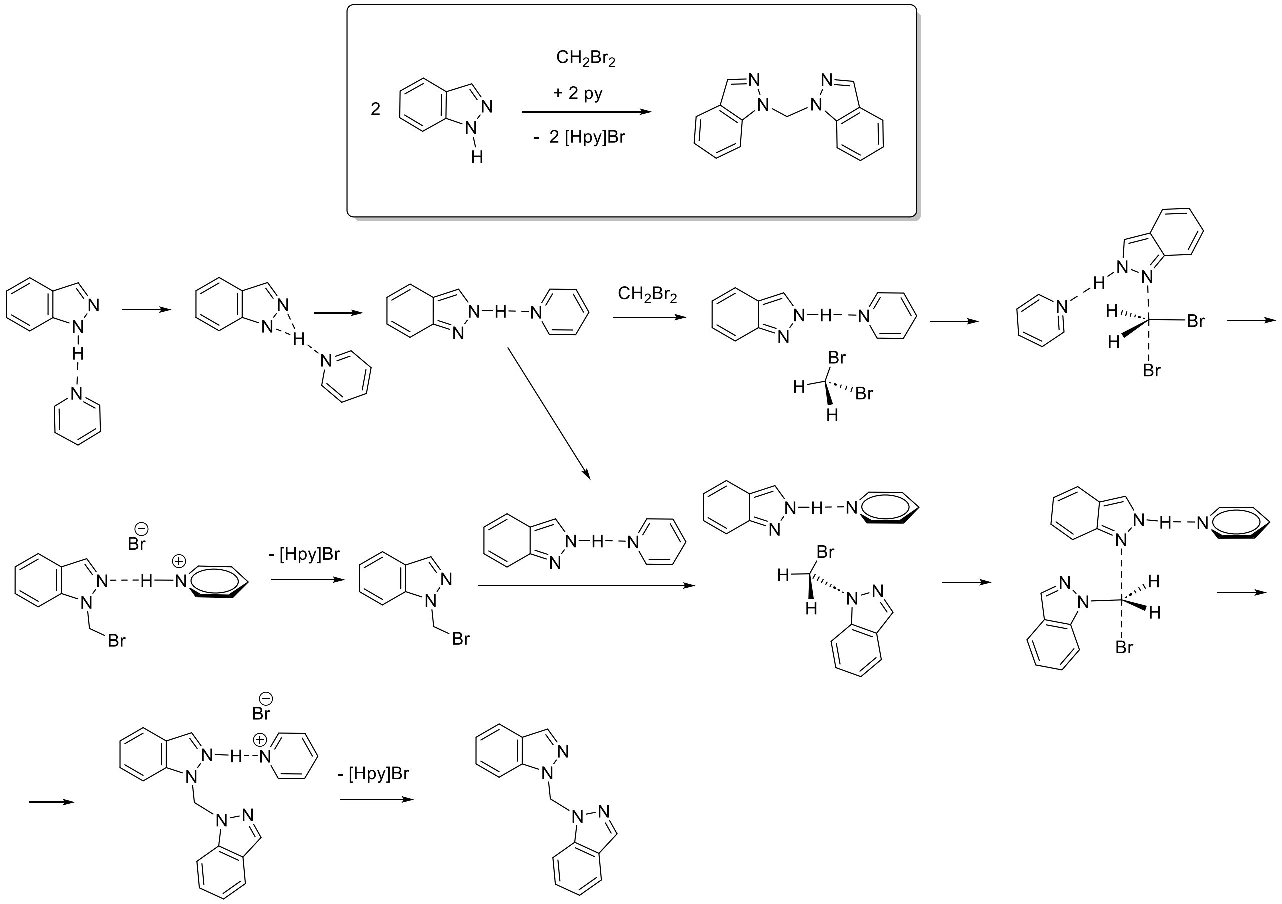

Scheme S4. Mechanism for the formation of **L1** compound.

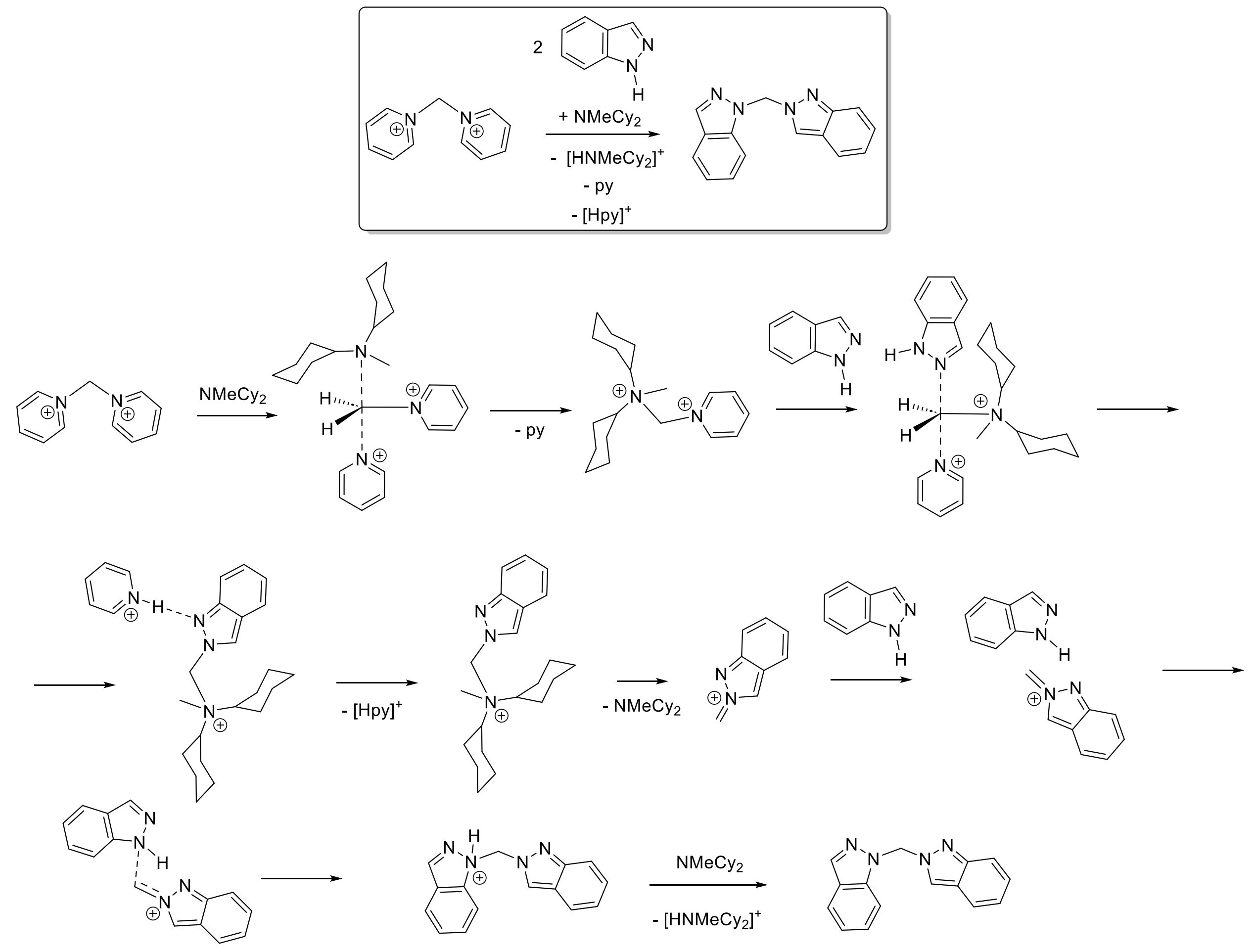

Scheme S5. Mechanism for the formation of **L3** compound.

4.2 **Table S14.** Coordinates of the optimized compounds

L1

N	0.56435600	2.17808000	0.85228200
N	-0.34239400	1.16698800	0.98735800
C	-1.49647900	1.42654700	0.28880100
C	-1.31266800	2.69353100	-0.31392700
C	-2.34107400	3.24955700	-1.09386800
H	0.53613000	3.99573000	-0.15206100
C	0.00000000	3.08909800	0.08735800
C	0.00000000	0.00000000	1.77023600
H	0.84338600	0.28913900	2.39712600
C	1.49647900	-1.42654700	0.28880100
N	0.34239400	-1.16698800	0.98735800
N	-0.56435600	-2.17808000	0.85228200
C	0.00000000	-3.08909800	0.08735800
H	-0.53613000	-3.99573000	-0.15206100
C	1.31266800	-2.69353100	-0.31392700
H	-0.84338600	-0.28913900	2.39712600

C	2.67576600	-0.68531500	0.12658500
C	2.34107400	-3.24955700	-1.09386800
C	3.51085300	-2.52763700	-1.24864900
C	3.67191000	-1.25981500	-0.64497700
H	2.21942200	-4.21930300	-1.56301400
H	4.31914300	-2.93306900	-1.84601200
H	4.59921600	-0.71913700	-0.79715800
H	2.79364800	0.29785100	0.56499600
C	-3.51085300	2.52763700	-1.24864900
C	-3.67191000	1.25981500	-0.64497700
C	-2.67576600	0.68531500	0.12658500
H	-2.21942200	4.21930300	-1.56301400
H	-4.31914300	2.93306900	-1.84601200
H	-4.59921600	0.71913700	-0.79715800
H	-2.79364800	-0.29785100	0.56499600

L2

N	-1.37396400	0.39371600	1.21797000
N	-0.87399200	-0.81721700	0.89196100
C	-1.60883000	-1.49735600	-0.02062700
H	-1.30685300	-2.46987800	-0.37453100
C	-2.69541400	-0.67864300	-0.32831700
C	-3.82625500	-0.77529200	-1.17799900
H	-3.98801000	-1.65389800	-1.79210700
C	-4.70823500	0.27640100	-1.20109300
H	-5.58154700	0.23191800	-1.84183100
C	-4.50537200	1.43886700	-0.39701400
H	-5.23242400	2.24142800	-0.45271300
C	-3.42252200	1.56335400	0.43701200
H	-3.26930500	2.44473400	1.04805800
C	-2.49465100	0.49249800	0.47946300
C	0.38890600	-1.23713800	1.49033700
H	0.51668100	-0.62836800	2.38425700
C	2.22121500	0.05178300	0.29410200
N	1.52351700	-1.08833300	0.61771600
N	1.99289000	-2.17588400	-0.06279200
C	2.99547300	-1.75044500	-0.80136300
H	3.53694600	-2.44816500	-1.42331500
C	3.21152400	-0.34785100	-0.63338300
H	0.32878800	-2.29094100	1.75680300
C	2.08402700	1.38173200	0.71545800
C	4.11137700	0.60276100	-1.14477200
C	3.99023000	1.91422100	-0.72190100
C	2.98521100	2.29569000	0.19545900
H	4.87898400	0.31507600	-1.85413100
H	4.66996200	2.66791900	-1.10193200
H	2.91220900	3.33564600	0.49285200
H	1.29530500	1.68506500	1.39242700

L3

N	1.90457200	0.96561500	-1.02710800
N	1.20546100	1.22254100	0.09992500
C	1.69391300	0.61919200	1.21128200
H	1.21057300	0.72782900	2.16863200
C	2.81850000	-0.09365800	0.79827600
C	3.78283500	-0.91731000	1.43287900
H	3.73008000	-1.11788900	2.49686200
C	4.78165500	-1.45418200	0.65983000
H	5.53232800	-2.08942900	1.11596200
C	4.86093800	-1.19683900	-0.74294900
H	5.67078500	-1.64556600	-1.30736400
C	3.94577200	-0.40318000	-1.38750300
H	4.00623300	-0.20715800	-2.45122200
C	2.90091800	0.16222400	-0.61362000
C	0.00000000	2.02497500	-0.00001600
H	-0.10474400	2.64275800	0.88931200
N	-1.90457100	0.96563000	1.02709400
N	-1.20546000	1.22253900	-0.09994400

C	-1.69391300	0.61917200	-1.21129100
H	-1.21057300	0.72779400	-2.16864200
C	-2.81849900	-0.09367100	-0.79827400
C	-3.78283500	-0.91733200	-1.43286500
H	-3.73008000	-1.11792800	-2.49684500
C	-4.78165600	-1.45419200	-0.65980700
H	-5.53232800	-2.08944600	-1.11593000
C	-4.86093900	-1.19682700	0.74296700
H	-5.67078500	-1.64554500	1.30738900
C	-3.94577200	-0.40315800	1.38750900
H	-4.00623400	-0.20712000	2.45122500
C	-2.90091900	0.16223400	0.61361800
H	0.10474400	2.64274500	-0.88935300

1*H*-indazole

C	0.25912000	-0.67391000	0.00001800
C	0.25738700	0.74220000	-0.00006800
C	-0.96634500	1.43362100	0.00000700
C	-2.13824200	0.69938000	0.00016500
C	-2.11508000	-0.71382700	0.00024900
C	-0.92629700	-1.42235100	0.00017800
C	1.64070100	1.09822700	-0.00020400
H	-0.99031200	2.51735700	-0.00004900
H	-3.09396100	1.21008000	0.00023000
H	-3.05403500	-1.25560200	0.00037300
H	-0.91692400	-2.50597600	0.00024000
H	1.97094000	-1.96475700	-0.00008100
H	2.08649000	2.08170200	-0.00029300
N	1.57623400	-1.03924800	-0.00010000
N	2.41381500	0.03027000	-0.00025600

2*H*-indazole

C	0.27340300	-0.69660000	-0.00001200
C	0.26063900	0.74065900	-0.00010000
C	-0.97059700	1.44354700	0.00003300
C	-2.13214500	0.71181400	0.00023200
C	-2.11567000	-0.71594100	0.00031300
C	-0.94161300	-1.42664900	0.00019500
C	1.61063900	1.09543100	-0.00032400
H	-0.99384900	2.52743700	-0.00004000
H	-3.08868200	1.22200300	0.00030900
H	-3.06167700	-1.24587100	0.00043600
H	-0.92718900	-2.50980600	0.00021400
H	2.11307900	2.04901300	-0.00043700
N	1.53059700	-1.18017600	-0.00026700
N	2.29169800	-0.07300900	-0.00008600
H	3.29431000	-0.18404300	-0.00003300

TS_{Ind}

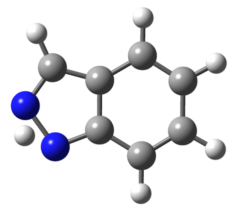

C	-0.26853600	-0.68985300	-0.00871300
C	-0.26269000	0.73379300	-0.00986100
C	0.96488100	1.44045900	-0.01317500
C	2.12958800	0.71070000	0.00337500
C	2.11553100	-0.71355300	0.01679200
C	0.93890000	-1.42486500	0.00985600
C	-1.61987100	1.09528900	0.02469800
H	0.98374900	2.52405200	-0.02204500
H	3.08523100	1.22243800	0.00750600

H	3.06133400	-1.24346700	0.02768200
H	0.92348100	-2.50779100	0.00837400
H	-2.06940900	2.07605600	0.08672400
N	-1.53827900	-1.17014400	-0.08503500
N	-2.39755100	0.00826800	-0.06279100
H	-2.42039500	-0.84996300	0.78871000

$TS_{Ind(py)}$

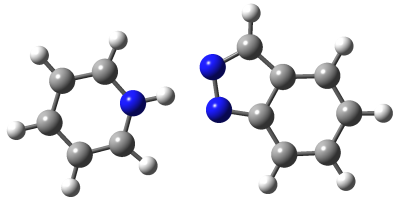

C	1.84441300	0.35457400	-0.00011700
C	2.54896500	-0.89045500	-0.00018100
C	3.95892800	-0.90504100	0.00012600
C	4.63499600	0.29986700	0.00048600
C	3.93162400	1.53348600	0.00054100
C	2.55176900	1.57677100	0.00024200
C	1.51306500	-1.85342300	-0.00056800
H	4.50576800	-1.84291800	0.00008300
H	5.71970000	0.31142300	0.00072000
H	4.49713000	2.45977900	0.00081800
H	2.02007500	2.52287200	0.00026700
H	1.57705600	-2.93271000	-0.00075400
N	0.50758200	0.12535200	-0.00047400
N	0.32978900	-1.23506200	-0.00067400
H	-1.17956700	-0.33786500	-0.00022100
C	-3.09142700	-1.13990100	0.00060300
C	-2.58799600	1.15975000	-0.00066500
C	-4.45100800	-0.87367900	0.00086600
H	-2.65448400	-2.12982800	0.00093700
C	-3.93421900	1.48548800	-0.00044200
H	-1.76902900	1.86758000	-0.00130500
C	-4.87472200	0.45510800	0.00033800
H	-5.15972200	-1.69094200	0.00145900
H	-4.23679000	2.52405800	-0.00088300
H	-5.93333900	0.68663400	0.00051700
N	-2.21638900	-0.12648400	-0.00013800

$TS_{Ind(PMP)}$

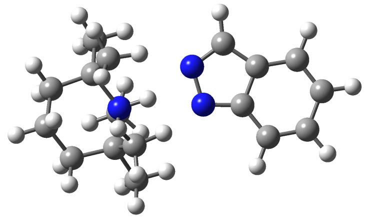

C	2.71030600	0.31872500	0.04644500
C	3.34047400	-0.95875200	-0.08788800
C	4.74367300	-1.04890400	-0.19988200
C	5.48702300	0.11512100	-0.17815800
C	4.85855700	1.38191000	-0.04572600
C	3.48745100	1.49856600	0.06625800
C	2.25172400	-1.85925200	-0.06585000
H	5.23336600	-2.01258800	-0.30195800
H	6.56754000	0.06881700	-0.26270400
H	5.47550700	2.27472400	-0.03263200
H	3.01574400	2.47093200	0.16743300
H	2.25144400	-2.93845800	-0.13979000
N	1.36418000	0.16415300	0.14057000
N	1.10803700	-1.17780900	0.06892100

H	-0.53592600	-0.21271000	0.14707900
C	-2.37794200	-1.21918000	-0.07938100
C	-1.88122800	1.40650800	-0.08855700
C	-3.40192700	1.60920000	0.02490800
C	-4.23814300	0.48707800	-0.59110600
C	-3.86751400	-0.85596000	0.03905200
H	-3.67152500	1.71536700	1.08239000
H	-3.63637700	2.56879400	-0.44516400
H	-5.30008400	0.68880100	-0.42358700
H	-4.10397000	0.45503700	-1.67590800
H	-4.15280700	-0.84583100	1.09774300
H	-4.43752200	-1.66899700	-0.42040700
N	-1.51241100	-0.02254700	0.46194100
C	-1.39057400	-0.03561700	1.95631400
H	-1.01674300	-1.00336000	2.26597700
H	-0.65121300	0.70099600	2.24457800
H	-2.35190900	0.17331400	2.42329000
C	-2.08295100	-2.48265300	0.74928100
H	-2.58783700	-3.32155700	0.26641500
H	-1.01158500	-2.69072800	0.76744000
H	-2.46605800	-2.42359600	1.76871400
C	-1.96570300	-1.53979400	-1.52880900
H	-0.87868000	-1.59744400	-1.61072700
H	-2.37545700	-2.51841000	-1.78674200
H	-2.34466000	-0.83347900	-2.26179300
C	-1.37089100	1.54757200	-1.53403700
H	-1.41592500	2.60429300	-1.80536600
H	-0.32876800	1.22838800	-1.59911400
H	-1.96138800	1.00534200	-2.26633100
C	-1.15243300	2.48054400	0.73914800
H	-0.07872600	2.28632600	0.75785100
H	-1.32174700	3.44359100	0.25331000
H	-1.53274900	2.56517300	1.75788700

a1

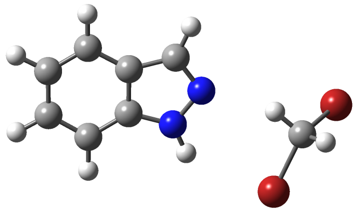

C	3.78059200	1.27519600	-0.57881000
C	2.87153200	0.31841600	-0.10321500
C	5.10995500	0.89808900	-0.64539400
C	3.28164800	-0.97837300	0.29352900
C	5.53915000	-0.39160400	-0.25362000
H	5.84406500	1.60875300	-1.00782200
C	4.64056300	-1.33227200	0.21420000
C	2.07465200	-1.62182600	0.70174800
H	6.59170400	-0.63958400	-0.32415500
H	4.97437000	-2.31945900	0.51287900
H	1.93499000	-2.62530700	1.07541100
N	1.03681300	-0.81746600	0.57346000
N	1.51960000	0.35506000	0.08742900
H	3.45948800	2.26482500	-0.88173300
C	-2.24570800	-0.19482600	0.74942500
H	-1.27244500	-0.67575300	0.75630700
H	-2.71927200	-0.15747100	1.72246500
H	0.87135400	1.10885300	-0.08435400
Br	-1.94715700	1.66857200	0.19731300
Br	-3.42122700	-1.18756700	-0.44481000

TS_{a1-2}

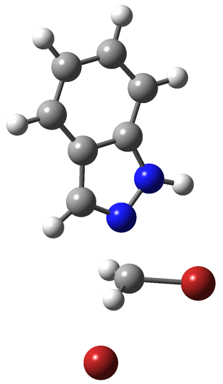

C	-1.15384900	-0.13841700	-0.13992000
H	-1.37714600	-0.71164600	-1.02027100
H	-1.16294900	-0.57170500	0.84356200
C	2.81539700	0.38437000	0.20998400
C	2.81749300	-0.93139100	-0.31944500
C	4.02694000	-1.64794500	-0.41105200
C	5.18140400	-1.02928600	0.02151200
C	5.15909300	0.28777900	0.54212500
C	3.98918800	1.01555200	0.64690800
C	1.46236900	-1.19555400	-0.65293400
H	4.04592900	-2.65531300	-0.80892600
H	6.12705200	-1.55449500	-0.03479500
H	6.08967000	0.73672000	0.86918100
H	3.98104800	2.02146800	1.04781700
H	1.11774200	1.71861700	0.36773100
H	1.00725700	-2.08368400	-1.06459600
N	1.51709600	0.80872300	0.18029700
N	0.72273100	-0.13767100	-0.36826800
Br	-1.58839200	1.71394900	-0.22761200
Br	-3.59148200	-1.20160600	0.27712600

a2

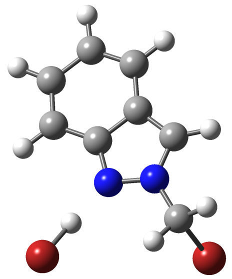

C	2.63143800	1.13354800	-0.76862900
C	1.75178600	0.21344400	-0.14561700
C	3.96821000	0.82635500	-0.77183500
C	2.25481000	-0.98605600	0.46348600
C	4.47434500	-0.36797000	-0.17239400
H	4.66933500	1.50624300	-1.24229400
C	3.64436600	-1.27045900	0.44160500
C	1.12385600	-1.61568400	0.97435400
H	5.54100600	-0.55606600	-0.20849500
H	4.03467400	-2.17396100	0.89505500
H	0.98955100	-2.54819400	1.49836000
N	0.06858100	-0.81759600	0.67561600
N	0.41641400	0.29393000	-0.01564400
H	2.24994600	2.03941300	-1.22292600
C	-1.31030700	-1.08804300	0.93376300
H	-1.41685600	-1.64798700	1.85669600
H	-1.86712000	-0.15781700	0.95289800
H	-0.60545500	1.73222500	-0.02481600
Br	-1.50282500	2.90804500	0.10749900

Br	-2.16063400	-2.21087400	-0.47471800

a3

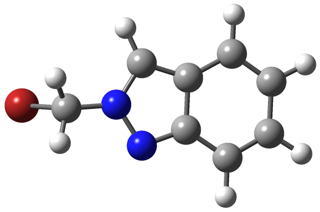

C	2.73123200	-1.44659500	-0.12131300
C	1.58431000	-0.67402700	0.19744100
C	3.88066900	-0.77601900	-0.45051400
C	1.64391600	0.76445600	0.17561000
C	3.94306500	0.65265200	-0.47456200
H	4.77372600	-1.33716800	-0.70185900
C	2.85246300	1.42499300	-0.16769000
C	0.36522500	1.16468600	0.54440000
H	4.87977100	1.12749200	-0.74315400
H	2.90929600	2.50723900	-0.18832300
H	-0.08709800	2.13652300	0.65815200
N	-0.34085000	0.02047000	0.75869300
N	0.36663800	-1.11295500	0.54896400
H	2.68269000	-2.52846200	-0.10284900
C	-1.71585200	-0.07974100	1.11017400
H	-2.01497200	0.75038900	1.74118300
H	-1.89425400	-1.04215900	1.57462500
Br	-2.94685300	-0.00425400	-0.46493300

b1

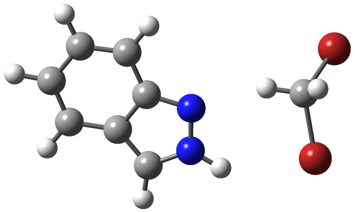

C	-3.11343300	1.49117000	0.68876800
C	-2.55304500	0.27377800	0.22856500
C	-4.46645200	1.66933400	0.54023500
C	-3.38583900	-0.73070200	-0.37170800
C	-5.29722300	0.67344400	-0.05583100
H	-4.92471300	2.59051500	0.88246800
C	-4.77950500	-0.51427000	-0.51018000
C	-2.50156000	-1.76346400	-0.69317600
H	-6.35967500	0.86767200	-0.14798000
H	-5.41762500	-1.26511100	-0.96218400
H	-2.64569800	-2.72545500	-1.15761300
N	-1.28447800	-1.34092800	-0.29290900
N	-1.26385200	-0.11853800	0.26878000
C	1.98948100	0.30896000	0.76125300
H	0.91559700	0.46042200	0.70156700
H	2.39541800	0.43657000	1.75697300
H	-2.48398900	2.24865500	1.14011200
H	-0.40709100	-1.83939300	-0.37099400
Br	2.86586600	1.59464800	-0.40866300
Br	2.31732100	-1.56628000	0.26006300

$TS_{b1\text{-}2}$

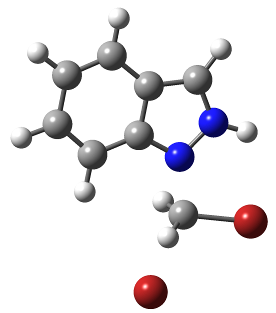

C	0.86089400	0.06896400	0.07153600
H	0.87013700	-0.50318100	0.97820300
H	0.78039300	-0.37658600	-0.90210000
C	-2.05028000	-0.22000000	0.16702200
C	-3.21271500	0.53745400	-0.18495500
C	-4.47416700	-0.09882000	-0.27737700
C	-4.53126100	-1.44846900	-0.03196000
C	-3.36445800	-2.19500600	0.30274200
C	-2.12506700	-1.61024800	0.40759000
C	-2.74736500	1.84560300	-0.37167400
H	-5.36317600	0.46407000	-0.53532100
H	-5.48039800	-1.96693000	-0.09570700
H	-3.46186500	-3.26008000	0.47758800
H	-1.24307300	-2.18789200	0.65504400
H	-3.25182900	2.76051500	-0.63773300
N	-0.97438200	0.60015200	0.21024700
N	-1.42613200	1.81733600	-0.13868000
H	-0.75385800	2.57644000	-0.16806300
Br	1.77478800	1.73864400	0.14835200
Br	2.92732000	-1.61594800	-0.17036500

b2

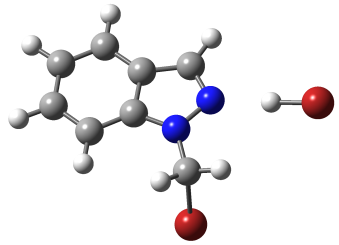

C	2.97881000	0.13488300	0.80382700
C	1.79946900	-0.45766500	0.33859000
C	4.15945000	-0.54602500	0.55443300
C	1.80017000	-1.68024000	-0.36798500
C	4.17851600	-1.77612600	-0.13862800
H	5.09575600	-0.12047900	0.89641800
C	3.01025000	-2.35162900	-0.60546600
C	0.42458800	-1.91695300	-0.67557700
H	5.12775100	-2.26996000	-0.30868800
H	3.02747100	-3.29293800	-1.14208900
H	-0.02127700	-2.74884600	-1.20080000
N	-0.33726400	-0.95305300	-0.21249300
N	0.48036100	-0.06514500	0.43408000
C	-0.03973400	1.15054600	0.92620500
H	0.51399800	1.48706000	1.79625800
H	-1.09889500	1.04665900	1.13141600
H	2.97873700	1.08483300	1.32331800
H	-2.19412900	-0.98559300	-0.08131800
Br	0.09443500	2.68536700	-0.37872100
Br	-3.64587000	-1.04005500	0.12220600

b3

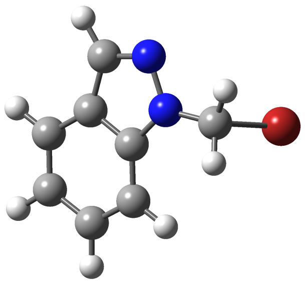

C	-1.23966500	-1.38793100	0.56243100
C	-0.99787900	-0.02514400	0.36018200
C	-2.49125900	-1.86211300	0.19973500
C	-1.96507300	0.83549600	-0.20197400
C	-3.47531000	-1.01469500	-0.35169100
H	-2.71793600	-2.91310800	0.33746000
C	-3.22503700	0.33181500	-0.55829300
C	-1.31612900	2.11239500	-0.25490800
H	-4.43889700	-1.43109500	-0.62060800
H	-3.98111600	0.97903300	-0.98758700
H	-1.70796100	3.05254800	-0.61507000
N	-0.09407300	2.05601300	0.21749000
N	0.10764400	0.76066200	0.62007700
C	1.37601700	0.36243800	1.07350000
H	1.30627400	-0.43735800	1.80308800
H	1.91830100	1.22243900	1.44720900
H	-0.48602200	-2.05108700	0.96875200
Br	2.57195300	-0.40719000	-0.37628800

b4

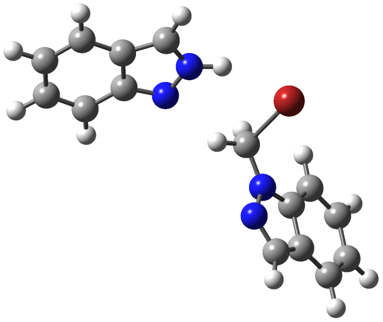

C	-4.01517100	-1.92090100	-0.66892200
C	-3.66979000	-0.61205600	-0.25043900
C	-5.34226300	-2.27211100	-0.64522800
C	-4.68599100	0.30582400	0.18337800
C	-6.35473800	-1.36246700	-0.21604400
H	-5.63737200	-3.26726500	-0.95898000
C	-6.04682400	-0.08902200	0.19562800
C	-3.98106100	1.46686900	0.51236900
H	-7.38729500	-1.69267100	-0.21730500
H	-6.82244500	0.59509400	0.52106100
H	-4.29669400	2.42787000	0.88489200
N	-2.68370200	1.18858500	0.27024600
N	-2.44502400	-0.05153200	-0.19101100
C	0.89516500	0.13332300	-0.48153900
H	-0.06553600	-0.28425500	-0.19807300
H	1.01640700	0.22939700	-1.55432100
H	-3.24705300	-2.61310800	-0.99248300
H	-1.88766100	1.80385600	0.39749900
C	3.29563600	-0.53878000	-0.26529900
C	3.98140800	-1.27727600	0.72161600
C	5.36596700	-1.47173100	0.61271300
C	6.02297500	-0.91355600	-0.47193100
C	5.32230400	-0.16746700	-1.44216000
C	3.95261100	0.03544300	-1.35757500

C	2.95921800	-1.65992900	1.65258100
H	5.90804100	-2.04093300	1.35904600
H	7.09308000	-1.04573400	-0.57991900
H	5.86956300	0.26266500	-2.27306100
H	3.42699000	0.62114500	-2.10152400
H	3.05768800	-2.24141800	2.55786300
N	1.96278300	-0.54897500	0.10537600
N	1.78036800	-1.21719000	1.29084000
Br	0.75641800	2.10606200	0.12435000

TS$_{b4-5}$

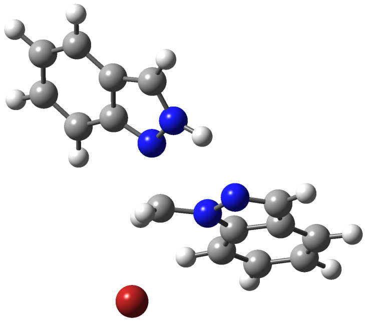

C	2.48436200	-0.14600300	1.51153800
C	2.06618100	-0.74441200	0.32038300
C	3.58533400	-0.71237500	2.13354000
C	2.74428700	-1.83844500	-0.25505200
C	4.26458400	-1.82337200	1.58732900
H	3.94933100	-0.27583200	3.05632900
C	3.86275200	-2.38937400	0.39073000
C	2.04069300	-2.10096600	-1.47147300
H	5.12574500	-2.22298400	2.10965000
H	4.39916900	-3.22645700	-0.04047100
H	2.23295500	-2.86650500	-2.20947600
N	1.03974200	-1.26697700	-1.64369600
N	1.04480600	-0.42201600	-0.55440200
C	0.04176600	0.51050100	-0.41819600
H	0.01376400	1.06739700	0.49900000
H	-0.30638700	0.99174200	-1.31644800
H	2.00588900	0.74298700	1.89975600
C	-2.73063100	-0.22406100	0.34319300
C	-3.75508100	-0.93282200	-0.36047000
C	-5.11096000	-0.78199900	0.01387800
C	-5.40038600	0.07033400	1.05168800
C	-4.37459300	0.78215200	1.73598700
C	-3.04632800	0.65075500	1.40481400
C	-3.07489900	-1.66491000	-1.34541200
H	-5.89438300	-1.32066500	-0.50578000
H	-6.42899200	0.21222100	1.36086100
H	-4.65458900	1.44980700	2.54245900
H	-2.27519500	1.20011600	1.92986500
H	-3.42473800	-2.34108300	-2.10890800
N	-1.51904700	-0.55027900	-0.17301700
N	-1.77301100	-1.39409600	-1.19559900
H	-0.96116100	-1.69413600	-1.74325000
Br	1.51681900	2.90819900	-0.58175500

b5

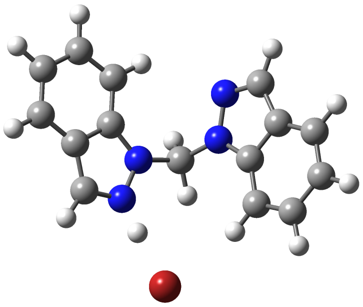

N	1.09268600	1.40967700	0.31239500
N	1.14090400	0.29769600	-0.46195800
C	2.30206700	-0.37419400	-0.18786500
C	3.00092600	0.37856800	0.79312500
C	4.25097300	-0.06930900	1.26709400
H	2.31124400	2.33854900	1.72149700

C	2.17779800	1.49721200	1.05945600
C	0.04424300	-0.02558000	-1.39563100
H	-0.56795300	0.87535700	-1.47540000
C	-1.85565000	-1.17890300	-0.15995400
N	-0.75050200	-1.14453700	-0.98746900
N	-0.36166900	-2.40389800	-1.35920400
C	-1.20985500	-3.23681100	-0.79696500
H	-1.10802800	-4.29932300	-0.96416400
C	-2.18888400	-2.54547400	-0.01904500
H	0.48841100	-0.26148000	-2.36164500
C	-2.59439500	-0.15849900	0.45435000
C	-3.29760400	-2.91722800	0.76057300
C	-4.02995200	-1.91662200	1.37284000
C	-3.67977200	-0.55659500	1.21755200
H	-3.57046800	-3.95992300	0.87623600
H	-4.88993400	-2.17340000	1.98034700
H	-4.27920900	0.20179700	1.70789100
H	-2.34299800	0.89074200	0.33756500
C	4.75553900	-1.24166400	0.74863800
C	4.03997700	-1.98258300	-0.22569800
C	2.81184100	-1.57558500	-0.70755700
H	4.79406200	0.49505600	2.01543200
H	5.71391800	-1.61327100	1.09067500
H	4.47005500	-2.90491300	-0.59868300
H	2.25110300	-2.16036900	-1.42434200
H	0.26843900	2.17544000	0.15046900
Br	-1.10148800	3.39150800	-0.30479100

a4

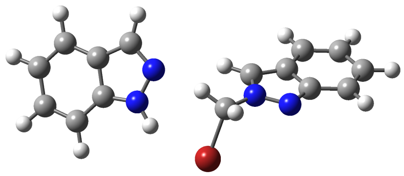

C	4.66745800	0.32497200	-1.09988800
C	3.65912800	-0.17031900	-0.25912000
C	5.87916300	-0.34173800	-1.08471600
C	3.85903000	-1.30054000	0.57166300
C	6.09807800	-1.47212300	-0.26231100
H	6.68442700	0.00976500	-1.71972400
C	5.10289200	-1.95791000	0.56465400
C	2.61442700	-1.46511900	1.24930200
H	7.06525800	-1.96028700	-0.28634400
H	5.27583100	-2.82410900	1.19309200
H	2.33412500	-2.21013200	1.97923200
N	1.74283100	-0.54401900	0.88052000
N	2.37515700	0.23584000	-0.03470600
H	4.50697400	1.18987200	-1.73249500
C	-1.06465200	1.41903100	1.19024000
H	-0.18656900	0.90818200	1.57044600
H	-1.51713300	2.11266000	1.88834700
H	1.87558500	1.02345800	-0.42481000
C	-3.94485700	-0.23157200	0.17740900
C	-3.00930400	-1.29315000	-0.09510600
C	-3.46501400	-2.52933600	-0.62427400
C	-4.80715300	-2.67195900	-0.86480000
C	-5.73311500	-1.61567900	-0.59295400
C	-5.32958200	-0.41044700	-0.07969000
C	-1.77771100	-0.77249800	0.28128700
H	-2.77005800	-3.33459000	-0.83309400
H	-5.18545000	-3.60311400	-1.27096800
H	-6.78383500	-1.78231700	-0.80283900
H	-6.03037800	0.38938500	0.12629700
H	-0.77330800	-1.16579100	0.27715100
N	-3.33307800	0.85031000	0.67931600
N	-2.02932200	0.48760900	0.73598200
Br	-0.35078000	2.59911900	-0.29206500

TS_{a4-5}

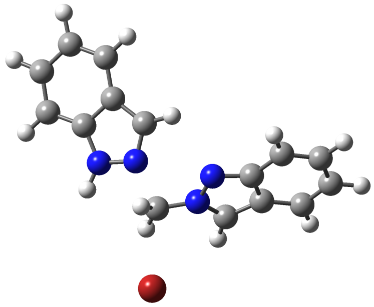

C	-4.99157800	0.53925400	0.59416700
C	-3.65002500	0.22028900	0.33957000
C	-5.92715700	-0.42987400	0.28532700
C	-3.25931400	-1.03021200	-0.20235300
C	-5.55840900	-1.68470300	-0.25760500
H	-6.97633000	-0.22431800	0.46276600
C	-4.23762400	-1.99753400	-0.50439200
C	-1.84327500	-0.96484500	-0.30840600
H	-6.33371700	-2.40738500	-0.48105300
H	-3.95679400	-2.95786300	-0.91951700
H	-1.12700600	-1.67304200	-0.69593800
N	-1.42271700	0.20184000	0.14483500
N	-2.49917400	0.93375500	0.52349500
H	-5.28269800	1.49665500	1.00824300
C	0.21502000	1.10986900	-0.10448600
H	0.12900700	1.80017700	0.71453000
H	0.00396800	1.44731400	-1.10458500
C	2.28275900	-1.60582600	-0.54286100
C	2.74091700	-1.22326700	0.77056600
C	3.77048600	-1.96186200	1.41363800
C	4.29454100	-3.04166800	0.75426600
C	3.83198400	-3.42323900	-0.54635700
C	2.84595900	-2.73254100	-1.19990300
C	1.98526400	-0.10819800	1.09088900
H	4.13004900	-1.67390000	2.39447900
H	5.08290000	-3.62452200	1.21625900
H	4.28801700	-4.28302700	-1.02439300
H	2.50678200	-3.01464800	-2.18914900
H	2.00637800	0.57380400	1.92460900
N	1.32634900	-0.78479400	-0.99270700
N	1.17310600	0.11728000	0.01727900
H	-2.37160100	1.86485200	0.89084800
Br	1.79602300	3.41158500	-0.27256500

a5

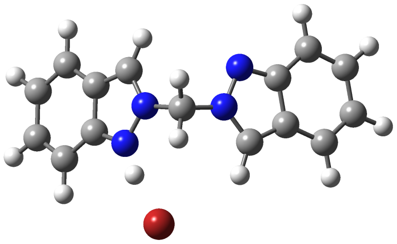

C	3.72034800	-0.50594400	-1.15385200
C	2.65910300	-0.65682400	-0.23881700
C	4.47307000	-1.62757200	-1.41307700
C	2.37965000	-1.90564000	0.39249000
C	4.20689300	-2.88262400	-0.79460700
H	5.29856700	-1.55861700	-2.11192000
C	3.17679000	-3.03777400	0.10078800
C	1.27621400	-1.65978900	1.22318200
H	4.83632300	-3.72891400	-1.04187900
H	2.97625100	-3.99351500	0.56931800
H	0.69856800	-2.30436300	1.86583000
N	0.94873100	-0.36822700	1.08849600
N	1.76241200	0.24970200	0.20259300
H	3.92180800	0.44826000	-1.62298300
C	-0.18517100	0.35758800	1.69279700
H	-0.28538800	0.00905600	2.71834800
H	0.05290900	1.42251300	1.64800600
H	1.62309400	1.33149600	0.01703700
C	-3.11885600	-1.01974800	0.40746600
C	-3.11849200	0.18264900	-0.38179300
C	-4.15208400	0.42211200	-1.32342100
C	-5.13729500	-0.52348900	-1.45326800
C	-5.13498700	-1.71643700	-0.66651400
C	-4.15274700	-1.97881500	0.25460900
C	-1.98385500	0.87625900	0.03302400
H	-4.15884600	1.32633600	-1.92071900
H	-5.94072600	-0.37040300	-2.16450500
H	-5.93897600	-2.43046700	-0.80697700
H	-4.15558800	-2.88231100	0.85234400
H	-1.53427200	1.81436700	-0.26101800
N	-2.06618500	-1.06071200	1.24293300
N	-1.41324100	0.09996100	0.99048600
Br	1.10093600	3.21508000	-0.07104300

b6

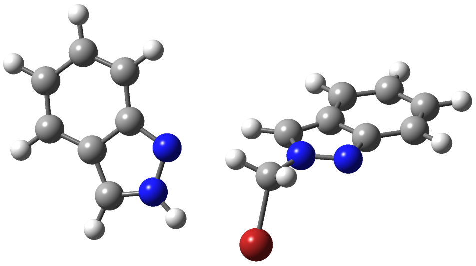

C	-0.96573200	1.44646500	1.21135000
H	0.03464900	1.07655900	1.40941700
H	-1.43064800	1.95920900	2.04441700
C	-3.62560900	-0.59725200	0.32472900
C	-2.56662000	-1.42062300	-0.20215100
C	-2.86923500	-2.65125100	-0.84261100
C	-4.18599600	-3.01928000	-0.94220600
C	-5.23474900	-2.19892400	-0.41829300
C	-4.97993800	-1.00708700	0.20890200
C	-1.40423300	-0.72091900	0.09539800
H	-2.08047300	-3.27846900	-1.24185000

H	-4.44921600	-3.95198600	-1.42788300
H	-6.25908900	-2.53826600	-0.52512000
H	-5.77367200	-0.38617900	0.60606000
H	-0.35658400	-0.90953300	-0.08063500
N	-3.14808000	0.51605400	0.89790900
N	-1.80581000	0.40592400	0.74947300
C	3.34188900	-1.81555500	1.10834100
C	3.11704100	-0.66675100	0.31000100
C	4.55799200	-2.44355000	0.99801800
C	4.13652600	-0.18802100	-0.58044900
C	5.57324600	-1.97078000	0.11387100
H	4.76025500	-3.32435900	1.59714600
C	5.38056300	-0.86018900	-0.67041000
C	3.56560300	0.93625700	-1.18362100
H	6.51467000	-2.50597700	0.06698600
H	6.15735700	-0.50731400	-1.33919400
H	3.93574600	1.63292400	-1.91824500
N	2.33025900	1.04883200	-0.65682800
N	2.01170100	0.10544800	0.25012100
H	2.57343400	-2.17584400	1.78189000
H	1.63912900	1.76353200	-0.85797100
Br	-0.68647300	2.90504700	-0.17099900

TS_{b6-7}

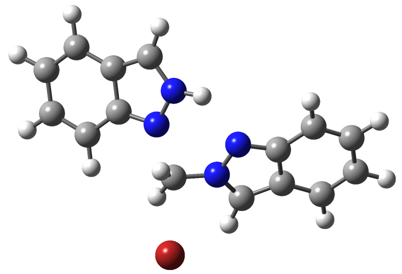

C	-3.53614600	0.44017600	1.10243900
C	-2.81489800	-0.46073000	0.28976900
C	-4.89513600	0.25030100	1.19886600
C	-3.47752400	-1.51913800	-0.40828500
C	-5.56189200	-0.80734200	0.51863500
H	-5.48126800	0.92595900	1.81090300
C	-4.87523500	-1.69135600	-0.27856100
C	-2.45808200	-2.17696700	-1.11548900
H	-6.63408400	-0.90922500	0.63620800
H	-5.38648000	-2.49524800	-0.79475100
H	-2.47591900	-3.03030000	-1.77427200
N	-1.31762000	-1.54174700	-0.82752900
N	-1.48339400	-0.51882500	0.03551800
H	-3.04051800	1.24928600	1.62404200
C	-0.18936500	0.89681200	-0.13622800
H	-0.53447900	1.49579300	0.68271200
H	-0.46497500	1.13980600	-1.14600700
C	2.49330900	-1.28151600	-0.39011300
C	2.87653700	-0.64010200	0.83762400
C	4.07354500	-1.01526800	1.49989800
C	4.83391000	-2.00966000	0.94041800
C	4.44419300	-2.65093800	-0.27566300
C	3.29711000	-2.30821600	-0.94608700
C	1.87249600	0.29037100	1.07601800
H	4.37589200	-0.52570500	2.41801800
H	5.75507500	-2.31908400	1.41999500
H	5.08496500	-3.42676200	-0.67960500
H	3.01496200	-2.78753600	-1.87572800
H	1.75314500	1.05620600	1.82412900
N	1.34404600	-0.77347300	-0.86832300
N	1.00006200	0.18293000	0.04480800
H	-0.35105900	-1.67464800	-1.16082400
Br	0.88817600	3.37030600	-0.43301700

b7

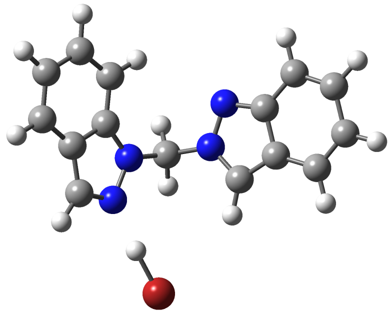

C	4.11714700	-1.49276400	0.25448800
C	2.94814000	-0.70411700	0.40318400
C	5.14482200	-0.97682700	-0.49434200
C	2.85938600	0.59063300	-0.21413200
C	5.06014100	0.30988800	-1.10872000
H	6.05150100	-1.55635500	-0.62822900
C	3.94196000	1.09482600	-0.97863100
C	1.59809700	1.06095000	0.14843100
H	5.90348300	0.66658900	-1.68885900
H	3.88495800	2.07050000	-1.44732900
H	1.07248500	1.97813900	-0.06759300
N	1.04420600	0.08750000	0.91313100
N	1.82842000	-0.99970100	1.08789300
H	4.18293000	-2.46814600	0.72128400
C	-0.29100100	0.07669600	1.48682700
H	-0.24639500	-0.54562800	2.37934100
H	-0.57344000	1.09287400	1.75541200
H	-1.93548000	2.12856700	-0.08215000
C	-1.61163900	-1.74591300	0.29442900
C	-2.66746300	-1.70269200	-0.64680200
C	-3.21772700	-2.90058000	-1.13792200
C	-2.69522800	-4.09392200	-0.67876100
C	-1.63055500	-4.11600800	0.25254100
C	-1.06711500	-2.95516300	0.75108400
C	-2.90700100	-0.31427800	-0.85537500
H	-4.02770400	-2.88495500	-1.85775700
H	-3.09759300	-5.03281800	-1.04022400
H	-1.23694300	-5.07278400	0.57606900
H	-0.22516900	-2.97649100	1.43057600
H	-3.62963100	0.16747300	-1.49717400
N	-1.30655600	-0.43995000	0.59441000
N	-2.09351000	0.41494000	-0.11974500
Br	-1.71207700	3.60597500	-0.02117100

a1$_{(PMP)}$

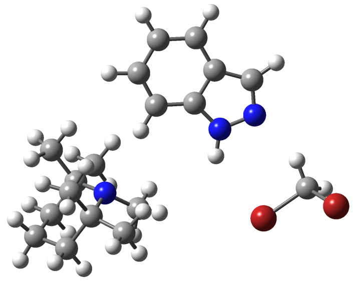

C	4.26251000	-0.69361400	-0.74443200
H	3.90688300	0.32870900	-0.65707100
H	4.85902000	-0.87508600	-1.62971000
C	0.23874900	2.56042100	-0.00643400
C	0.97995600	3.76713800	-0.04631100
C	0.32533600	4.99186200	0.17910300
C	-1.03293300	4.97231700	0.43480400
C	-1.75309600	3.75480000	0.47054500
C	-1.14039400	2.53323600	0.25266400
C	2.31536200	3.35413600	-0.33319100
H	0.87330800	5.92694500	0.15283500
H	-1.56128400	5.90190800	0.61206500
H	-2.81761100	3.78196300	0.67545300
H	-1.70197300	1.60420400	0.27967100
H	3.20188900	3.95994800	-0.44872800
N	1.13929200	1.56405800	-0.26031000
N	2.39446600	2.04269600	-0.45791400
H	0.98537000	0.56912400	-0.31845700
Br	2.66598300	-1.82880800	-0.89834300
Br	5.36811000	-1.09894800	0.80773700
C	-4.13054300	-0.47395900	-1.01466600
C	-3.48858200	-1.34848700	1.31180800
C	-4.40723500	-2.57421200	1.07175500
C	-5.56494000	-2.28564000	0.11387600
C	-5.03138600	-1.72120900	-1.20463700
H	-3.80207200	-3.38959200	0.65632000
H	-4.78519800	-2.93171000	2.03589400
H	-6.12750500	-3.20552700	-0.07588300
H	-6.27513200	-1.58783500	0.56981500
H	-4.44915400	-2.50054600	-1.71178400
H	-5.85772300	-1.46732900	-1.87773600
N	-3.03768000	-0.72619700	0.00482100
C	-1.86906000	-1.40181100	-0.57403600
H	-1.49372800	-0.83467300	-1.42226500
H	-1.06084300	-1.43637000	0.15240800
H	-2.04914700	-2.43479200	-0.91836500
C	-3.52476900	-0.11425100	-2.38890900
H	-4.33542600	0.17477300	-3.06145500
H	-2.83841400	0.73379200	-2.31502500
H	-3.00294100	-0.94952800	-2.85770000
C	-4.98594200	0.75379100	-0.62021900
H	-4.34138000	1.58989000	-0.34243600
H	-5.59150200	1.06173400	-1.47672300
H	-5.67390100	0.56751600	0.19957100
C	-4.19893800	-0.30410900	2.20601500
H	-4.27277700	-0.69157400	3.22560400
H	-3.62185400	0.62236000	2.23598000
H	-5.21048300	-0.06479500	1.88893600
C	-2.27443400	-1.82289600	2.13952800
H	-1.56279400	-1.00978000	2.30907800
H	-2.63004700	-2.15856200	3.11613300
H	-1.74735300	-2.66268200	1.68504500

TS$_{a1\text{-}2(PMP)}$

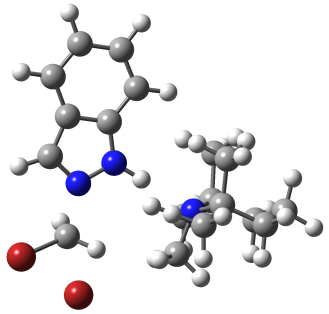

C	-2.22737800	-0.26047100	0.01416300
H	-2.29075100	-0.14621900	-1.05114600
H	-1.49896100	-0.87758400	0.49702200
C	0.46725500	2.72627100	-0.38599500
C	-0.68548200	3.53133400	-0.19359600
C	-0.62464400	4.92355300	-0.40037800
C	0.57919600	5.47074300	-0.79050300
C	1.72279800	4.65684400	-0.97869200
C	1.69305600	3.28964600	-0.78296400
C	-1.69988300	2.62270900	0.19552300
H	-1.50215100	5.54298900	-0.25747800
H	0.65867300	6.53786700	-0.95963400
H	2.65088900	5.12270500	-1.28966900
H	2.57456500	2.68373800	-0.93757800
H	0.71120400	0.59034500	-0.01468200
H	-2.74048600	2.79613900	0.42103000
N	0.10171400	1.43389100	-0.12313100
N	-1.20442500	1.39818200	0.25151800
Br	-3.80684500	0.07448800	0.99780100
Br	-2.68524500	-2.71375000	-0.96642100
C	2.85858400	-0.72327800	1.42908200
C	2.48504100	-1.43801300	-1.03109500
C	3.53112100	-2.55800500	-0.82001100
C	4.57916500	-2.22517300	0.24396600
C	3.89297100	-1.86754500	1.56422000
H	3.00951000	-3.47626900	-0.52804300
H	4.01042500	-2.77330600	-1.78051400
H	5.24153200	-3.08360800	0.39284700
H	5.22248600	-1.40416200	-0.09059200
H	3.39065400	-2.75980600	1.95412500
H	4.63267100	-1.58024000	2.31895400
N	1.85898700	-1.00077200	0.29941600
C	0.84515900	-1.98837200	0.74614900
H	0.29978500	-1.59616700	1.60148300
H	0.11671200	-2.18137100	-0.03538100
H	1.26804300	-2.95678800	1.04342600
C	2.11452900	-0.58195000	2.77601300
H	2.82615300	-0.24582200	3.53318900
H	1.31745900	0.16509400	2.71831900
H	1.68865000	-1.51949400	3.13225300
C	3.58741600	0.62500300	1.23444200
H	2.87739700	1.43016700	1.03966800
H	4.11700900	0.87490200	2.15703600
H	4.33046200	0.62038100	0.44203000
C	3.11843000	-0.24189100	-1.77420800
H	3.41912700	-0.56869900	-2.77247600
H	2.38952300	0.56094600	-1.90464200
H	4.00368700	0.16847400	-1.29804100
C	1.39763200	-1.97125200	-1.98929800
H	0.57043200	-1.26612500	-2.10456600
H	1.84541600	-2.11434300	-2.97534100
H	0.97929900	-2.92959500	-1.68540500

a2$_{(PMP)}$

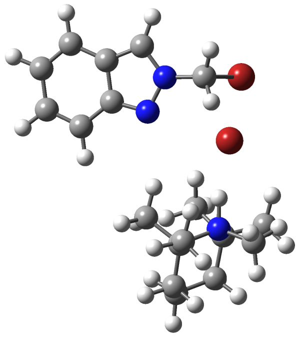

C	-2.14387700	-1.70800900	-0.50106500
H	-2.54615900	-2.30925900	-1.30864800
H	-1.07085100	-1.56848100	-0.60602900
C	-3.14056800	1.57919200	0.08492200
C	-4.33996600	1.13158100	-0.57193500
C	-5.42782200	2.02175100	-0.76148300
C	-5.29939800	3.30674300	-0.29799400
C	-4.10804500	3.74869800	0.35595400
C	-3.03635200	2.91483100	0.55165900
C	-4.07830800	-0.19808800	-0.88409100
H	-6.33491100	1.69445200	-1.25677300
H	-6.11486800	4.00947400	-0.42579700
H	-4.05863100	4.77435300	0.70475000
H	-2.13484700	3.25173300	1.04922300
H	2.47041500	-0.43098000	-0.59334500
H	-4.66697400	-0.95876700	-1.37045200
N	-2.22472400	0.60057500	0.16219100
N	-2.82050700	-0.45374800	-0.43668000
Br	-2.41554300	-2.80608400	1.15242700
Br	1.30644300	-1.62920400	-1.86978400
C	2.73170400	0.04581800	1.47300400
C	3.47445800	1.44576100	-0.66284500
C	4.36012500	2.28093400	0.27930300
C	3.85017300	2.35222600	1.72132200
C	3.66244500	0.94804400	2.30271500
H	5.37620200	1.86962300	0.27797000
H	4.43895900	3.28435700	-0.14974900
H	4.56717800	2.90697100	2.33333300
H	2.91387300	2.91487100	1.76914600
H	4.64124700	0.46485400	2.40291200
H	3.24677300	1.00415400	3.31323700
N	3.23782600	0.03995500	-0.01236900
C	4.40754900	-0.87582900	-0.20918000
H	4.13445900	-1.86818800	0.12772600
H	4.61754700	-0.94986300	-1.26902400
H	5.28097500	-0.51402300	0.33116600
C	2.76235600	-1.39193200	2.02285200
H	2.28242700	-1.38357000	3.00286900
H	2.19914900	-2.07442500	1.38191400
H	3.77303100	-1.77618000	2.16403300
C	1.26386000	0.51319600	1.53557200
H	0.65979100	0.07416300	0.74154200
H	0.84146100	0.18212000	2.48562100
H	1.14566800	1.59224400	1.49808100
C	2.12213200	2.12477700	-0.95220900
H	2.30219900	2.95574200	-1.63712500
H	1.43301500	1.43170700	-1.43865800
H	1.64092900	2.53634800	-0.07112800
C	4.17423400	1.26944400	-2.02373500
H	3.61810300	0.58511900	-2.66897700
H	4.19930500	2.24514400	-2.51246400
H	5.20666100	0.92908300	-1.93643800

a4$_{(PMP)}$

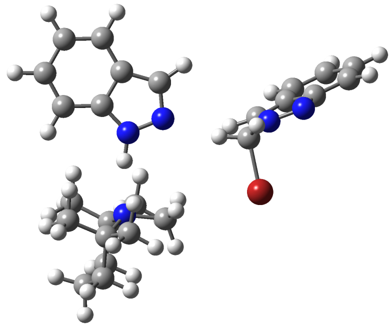

C	3.13518500	3.35508300	-0.49414500
C	1.84235300	2.80432300	-0.46901000
C	3.23904400	4.71437500	-0.72756000
C	0.68990000	3.60523600	-0.67341100
C	2.09836700	5.52567200	-0.93385100
H	4.22129700	5.17280800	-0.75386800
C	0.82665400	4.98546500	-0.90893700
C	-0.39436200	2.68751000	-0.57064500
H	2.23150000	6.58612800	-1.11343800
H	-0.04563300	5.60966800	-1.06699300
H	-1.45393500	2.88009200	-0.65811800
N	0.03900200	1.46129800	-0.33162200
N	1.39450700	1.52862400	-0.27260400
H	4.01644300	2.74564700	-0.33836800
C	-2.43786700	-0.59009400	-1.73158300
H	-1.49842100	-0.08261700	-1.53620300
H	-2.84955300	-0.38395600	-2.71246000
C	-5.30978600	-0.03539800	0.14897100
C	-4.34735200	0.30228700	1.16573000
C	-4.78190600	0.70511600	2.45546900
C	-6.13138700	0.75626200	2.69494900
C	-7.08414400	0.41945800	1.68305900
C	-6.70018700	0.02940500	0.42574200
C	-3.11599300	0.13627300	0.54164900
H	-4.06645700	0.96228300	3.22826100
H	-6.49387600	1.05769000	3.67116200
H	-8.13939500	0.47620100	1.92625000
H	-7.42141100	-0.22607800	-0.34105100
H	-2.09790200	0.28254300	0.86555800
N	-4.71279300	-0.37905100	-1.00199400
N	-3.39282000	-0.25864200	-0.73157100
H	1.93269100	0.67422800	-0.05318700
Br	-1.98775800	-2.54645400	-1.76623800
C	3.28916700	-0.97120600	1.88243200
C	3.69850300	-1.59810400	-0.58687300
C	4.42128000	-2.87982200	-0.10462300
C	5.06883400	-2.72827800	1.27332900
C	4.02793600	-2.26765700	2.29571600
H	3.69522800	-3.70050100	-0.06615500
H	5.16817000	-3.16594400	-0.85291400
H	5.49984500	-3.68390000	1.58812400
H	5.90378300	-2.02141700	1.22929400
H	3.28998500	-3.06711100	2.43217900
H	4.49237000	-2.11023700	3.27504700
N	2.72589700	-1.07544600	0.46693200
C	1.44863500	-1.82225400	0.44579600
H	0.72618000	-1.33930400	1.09658600
H	1.01511100	-1.79701700	-0.54828500
H	1.53520300	-2.87509400	0.75123700
C	2.15076900	-0.72721300	2.89848000
H	2.59526400	-0.52625200	3.87568800
H	1.54541700	0.14125000	2.62469700
H	1.49376800	-1.58904000	3.01607300
C	4.22432900	0.25098400	2.02640800
H	3.74460400	1.15302700	1.64177200
H	4.43265800	0.41556000	3.08661300

H	5.18515200	0.13730200	1.53291200
C	4.73025900	-0.51607700	-0.98242600
H	5.25448200	-0.83795700	-1.88567300
H	4.23183800	0.42842400	-1.20997300
H	5.49114700	-0.32825700	-0.23013100
C	2.94882800	-1.93293800	-1.89486100
H	2.35932600	-1.08294500	-2.24929400
H	3.68540200	-2.16909500	-2.66586900
H	2.29233500	-2.79815100	-1.80395200

TS$_{a4\text{-}5(PMP)}$

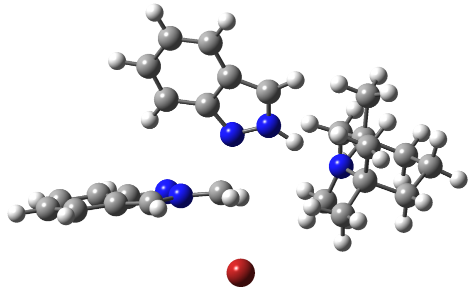

C	2.46647100	2.59756000	0.00269800
C	1.09776800	2.32089900	-0.21135400
C	2.93335900	3.82690000	-0.40379300
C	0.24300700	3.29319100	-0.81581300
C	2.08900700	4.79678400	-1.01238700
H	3.97783000	4.07158600	-0.24754300
C	0.75272300	4.54912600	-1.21800400
C	-1.01935800	2.68313100	-0.84037900
H	2.51291100	5.74946200	-1.30725000
H	0.10618600	5.29306300	-1.66868000
H	-1.97699000	3.04419800	-1.17881900
N	-0.88794000	1.46498600	-0.30263000
N	0.37763200	1.21285600	0.11199300
H	3.11397400	1.88176600	0.48949500
C	0.92539400	-0.68518100	-0.12373700
H	0.56632600	-0.74236900	-1.13274200
H	0.33646400	-1.02632400	0.70407400
C	4.11910700	-0.72378100	1.13499500
C	4.46207800	-0.73175300	-0.26873000
C	5.82504000	-0.76802500	-0.67255000
C	6.78399700	-0.78436300	0.30406500
C	6.44046700	-0.77034900	1.69587400
C	5.14048300	-0.74069600	2.12399700
C	3.24372800	-0.70872600	-0.92104800
H	6.09223200	-0.78623800	-1.72263000
H	7.83205200	-0.81462200	0.02916800
H	7.24270000	-0.79019500	2.42507300
H	4.88064100	-0.73883400	3.17537400
H	2.97093200	-0.76438300	-1.96158200
N	2.79665500	-0.70291000	1.32364000
N	2.28901500	-0.69871800	0.06052600
H	-1.68992000	0.79918700	-0.08370900
Br	0.47640800	-3.32069200	-0.91445900
C	-3.96414000	-0.57763600	-0.87068200
C	-3.92762900	0.49160500	1.49074200
C	-5.25496800	-0.24133600	1.80011700
C	-6.10877900	-0.49790100	0.55685400
C	-5.29666100	-1.26535200	-0.48759000
H	-5.02988800	-1.20051900	2.27991900
H	-5.81241400	0.34508800	2.53818100
H	-6.99739300	-1.07489200	0.83126800
H	-6.47975100	0.44530200	0.14177000
H	-5.07576400	-2.26576700	-0.09953700
H	-5.88384900	-1.41532900	-1.39943600
N	-3.14397400	-0.21937200	0.37801300
C	-2.45517200	-1.42381900	0.91231700

H	-1.84057100	-1.89277500	0.14915600
H	-1.78569200	-1.13782200	1.71929700
H	-3.13997400	-2.18680000	1.30289700
C	-3.16999500	-1.54203800	-1.77664900
H	-3.71365500	-1.65830000	-2.71700500
H	-2.17588300	-1.15770100	-2.01770100
H	-3.04366000	-2.53639800	-1.35186100
C	-4.23292600	0.66845000	-1.74377900
H	-3.30102100	1.18897600	-1.97583400
H	-4.66461000	0.34356200	-2.69341400
H	-4.92699100	1.38272000	-1.31150800
C	-4.21478700	1.96938200	1.13835100
H	-4.58476600	2.47650200	2.03257400
H	-3.30494900	2.48556000	0.82745000
H	-4.96844600	2.10594600	0.36883700
C	-3.08871300	0.54357700	2.78700000
H	-2.09964400	0.97716100	2.61367600
H	-3.60441300	1.18024900	3.50915500
H	-2.96301100	-0.43263100	3.25405700

a5$_{(PMP)}$

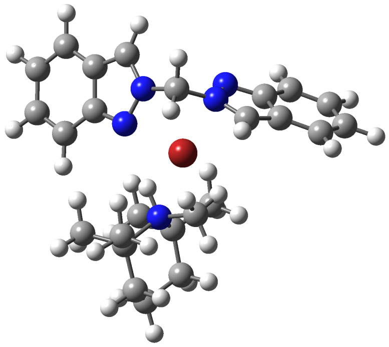

C	-3.36278900	2.18591000	-0.42797400
C	-2.10805100	2.32494100	0.21997900
C	-4.12291600	3.31446600	-0.62299100
C	-1.68412600	3.62215500	0.65996600
C	-3.69103400	4.60245000	-0.19553900
H	-5.08651800	3.22573500	-1.11258900
C	-2.48719900	4.76682400	0.44361600
C	-0.45169600	3.38906700	1.26435900
H	-4.33101700	5.45810600	-0.37594500
H	-2.15841800	5.74441800	0.77702200
H	0.25641700	4.05121000	1.73430200
N	-0.21260100	2.06753000	1.16829700
N	-1.19249400	1.37598800	0.53097300
H	-3.72291300	1.21949200	-0.75204200
C	0.95869100	1.41577300	1.72132500
H	0.69368200	0.47663700	2.22558600
H	1.38329100	2.09990700	2.45701600
C	3.27656500	1.47809100	-0.92563000
C	3.70677100	0.25532600	-0.30664000
C	4.81695000	-0.45698900	-0.82564200
C	5.45636400	0.05531200	-1.92866300
C	5.02476400	1.26980100	-2.54209600
C	3.95339800	1.98432100	-2.06363600
C	2.81817300	0.07245800	0.75684800
H	5.15478500	-1.37513400	-0.35837000
H	6.31250700	-0.46384100	-2.34447400
H	5.56698900	1.63549500	-3.40730800
H	3.63059400	2.90925800	-2.52684100
H	2.71215000	-0.68004400	1.52936200
N	2.21437600	2.00638700	-0.28667400
N	1.96415800	1.12221800	0.70288400
H	-1.08696900	-0.74839200	-0.19119500
Br	0.94232900	-1.71430400	3.40787500

C	-0.92973900	-1.47080500	-2.17038800
C	-2.24627800	-2.51807100	-0.06952100
C	-2.40104400	-3.79053100	-0.92146000
C	-2.41277900	-3.55512200	-2.43267900
C	-1.14060500	-2.82457700	-2.86644300
H	-1.59340900	-4.48691300	-0.67284800
H	-3.32587500	-4.27776500	-0.59955400
H	-2.47425100	-4.51702500	-2.94907200
H	-3.30271600	-2.99363800	-2.73277000
H	-0.27301700	-3.46566700	-2.67780500
H	-1.15046100	-2.63572900	-3.94381000
N	-1.00421900	-1.69397100	-0.60804500
C	0.28216700	-2.26708400	-0.06753500
H	1.11063400	-1.66535100	-0.41744900
H	0.28555200	-2.21990000	1.02328800
H	0.39679200	-3.29305400	-0.41102800
C	0.44817200	-0.89514700	-2.54443400
H	0.41159200	-0.60747300	-3.59690700
H	0.68970200	0.00335700	-1.97136400
H	1.25927600	-1.61380300	-2.43805200
C	-1.96826700	-0.42953700	-2.61641700
H	-1.86298800	0.49704600	-2.05206000
H	-1.77542400	-0.19513400	-3.66500100
H	-2.99664900	-0.76970600	-2.55192500
C	-3.51035500	-1.64396600	-0.09474400
H	-4.28577700	-2.16609000	0.46843800
H	-3.33504100	-0.69120700	0.40729600
H	-3.90976500	-1.45731000	-1.08765300
C	-2.00309500	-2.90363100	1.39846100
H	-1.75842400	-2.04636300	2.02901000
H	-2.92481400	-3.35094700	1.77692800
H	-1.20781200	-3.63520300	1.52742000

b1$_{(PMP)}$

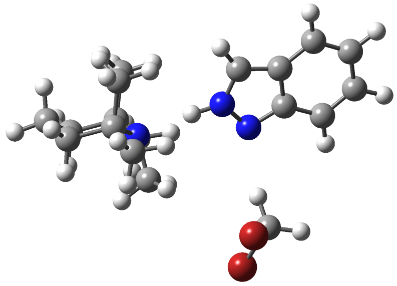

C	2.61669000	-1.49088500	-0.06410800
H	3.67460900	-1.26152800	-0.09234600
H	1.98810900	-0.63771000	-0.30431100
C	1.83938500	2.44512300	-0.44468100
C	1.28043400	3.47831900	0.37883700
C	2.02135500	4.65324200	0.65319700
C	3.27799500	4.76939000	0.10886100
C	3.82935900	3.74277200	-0.71309500
C	3.13562000	2.59108400	-0.99715400
C	0.01462300	2.98962400	0.72048200
H	1.60861500	5.44008700	1.27475200
H	3.86836500	5.65806500	0.30108900
H	4.82285900	3.88372900	-1.12436600
H	3.55421500	1.81781400	-1.63081500
H	-0.78055500	3.40727300	1.31675900
N	0.97974700	1.41245100	-0.58389300
N	-0.09830500	1.78342300	0.13194300
H	-0.93197500	1.15860300	0.14069600
Br	2.28974400	-2.87860800	-1.40200300
Br	2.20060600	-2.04257100	1.76647700
C	-3.13645000	0.28368400	-1.32300800
C	-3.23144100	-0.15527500	1.21895300
C	-4.50854500	-1.00078600	0.98937800

C	-5.31019500	-0.56974600	-0.24059900
C	-4.41579300	-0.57462900	-1.48163900
H	-4.21834000	-2.05141800	0.87141100
H	-5.12915100	-0.95366500	1.89069500
H	-6.15370600	-1.25136000	-0.38915100
H	-5.74924900	0.42150200	-0.08635100
H	-4.12199300	-1.60771100	-1.70192900
H	-4.96963900	-0.22049200	-2.35766200
N	-2.38020000	-0.07820100	-0.04478700
C	-1.55950600	-1.29765300	-0.22336000
H	-0.88309400	-1.16937400	-1.06213100
H	-0.93143000	-1.45471200	0.64853100
H	-2.14483900	-2.21289800	-0.39143300
C	-2.24329900	0.04992200	-2.56151700
H	-2.75672000	0.45147800	-3.43797700
H	-1.28291000	0.56439900	-2.47286800
H	-2.05583300	-1.00617100	-2.75750300
C	-3.49304000	1.78885300	-1.35751400
H	-2.62384500	2.40376000	-1.11573300
H	-3.81228700	2.05247400	-2.36876300
H	-4.30480900	2.06819500	-0.69180100
C	-3.60710100	1.25549200	1.72853000
H	-4.01177100	1.17263700	2.74027900
H	-2.72545300	1.89830800	1.77826000
H	-4.35908100	1.75957100	1.12918000
C	-2.42978100	-0.79985100	2.37100100
H	-1.47775400	-0.28833100	2.53766100
H	-3.01540700	-0.71766800	3.28930900
H	-2.22972100	-1.85940900	2.21169400

$TS_{b1-2(PMP)}$

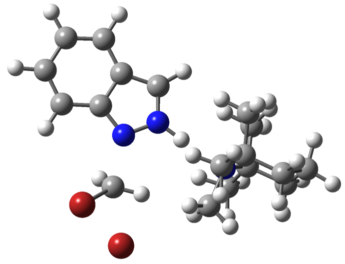

C	1.59886500	1.08884400	0.00005900
H	2.13657300	1.01968100	-0.92580800
H	0.54055500	1.21749700	0.06582900
C	2.37391000	-1.81246200	-0.12239200
C	1.76458300	-2.97066800	-0.69581000
C	2.55255400	-4.08433600	-1.06897200
C	3.91074800	-4.00642800	-0.87436200
C	4.51180300	-2.84468300	-0.31312800
C	3.77272300	-1.74845700	0.06749600
C	0.39887100	-2.65837600	-0.74914100
H	2.09696300	-4.96962300	-1.49702400
H	4.54393800	-4.84101000	-1.15132700
H	5.58761700	-2.82857900	-0.18089400
H	4.23773200	-0.87290200	0.50076800
H	-0.44960400	-3.22958800	-1.08896000
N	1.42158100	-0.88871600	0.16730300
N	0.25176400	-1.42408400	-0.24961400
H	-0.66655700	-0.91779400	-0.10374300
Br	2.59345200	1.57349200	1.53486300
Br	1.10205700	3.42807000	-1.12934800
C	-3.03575200	-0.88879800	1.32078900
C	-3.12477200	-0.02800800	-1.12206600
C	-4.58739700	0.41044400	-0.86867600
C	-5.30189000	-0.42695100	0.19420200
C	-4.49999400	-0.42016600	1.49785700
H	-4.59201400	1.46071200	-0.55654200
H	-5.13036500	0.37283100	-1.81859500

H	-6.30243300	-0.02179300	0.37394600
H	-5.45190600	-1.45217900	-0.16043300
H	-4.50244400	0.59522900	1.90965900
H	-4.98003800	-1.05479500	2.25012000
N	-2.33624300	-0.11693300	0.19336000
C	-1.95146200	1.23976900	0.66153700
H	-1.26919700	1.16276900	1.50418500
H	-1.42997000	1.78494600	-0.11966200
H	-2.80282400	1.85578900	0.97746300
C	-2.29013500	-0.66939000	2.65621600
H	-2.71590400	-1.33919400	3.40626200
H	-1.22560100	-0.90554500	2.57034300
H	-2.39080500	0.34537400	3.03981500
C	-2.99588600	-2.41585300	1.08486900
H	-1.98084600	-2.75759800	0.87458000
H	-3.32338300	-2.91878100	1.99790000
H	-3.64707800	-2.76151100	0.28810000
C	-3.09526600	-1.36860400	-1.89071000
H	-3.50779500	-1.20724100	-2.88927400
H	-2.07002500	-1.72192000	-2.02019000
H	-3.67928100	-2.16238300	-1.43476600
C	-2.46794500	0.99607000	-2.07302500
H	-1.40046400	0.80549400	-2.21086500
H	-2.94447500	0.91498800	-3.05238500
H	-2.58426100	2.02889100	-1.74735300

b2$_{(PMP)}$

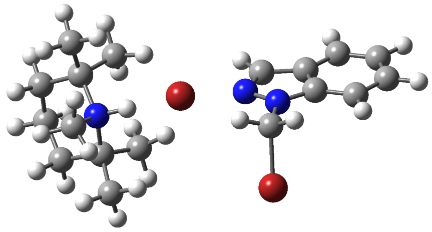

C	2.13716100	-0.99680800	0.48383300
H	2.68951300	-1.47935400	1.28213800
H	1.08063900	-0.91080700	0.72370300
C	4.02457600	0.63133300	0.22605300
C	4.08908500	1.93476300	-0.31279700
C	5.31789100	2.61105900	-0.36254900
C	6.44351900	1.96242200	0.11644400
C	6.36206000	0.65400000	0.63989100
C	5.16001200	-0.03302400	0.70358000
C	2.73996000	2.22348400	-0.69510200
H	5.38416300	3.61440900	-0.76772800
H	7.40575200	2.46023100	0.08842600
H	7.26498600	0.17252100	0.99771100
H	5.11144100	-1.04137800	1.09522100
H	2.34763200	3.12232500	-1.14846200
N	2.69953700	0.25227100	0.16118400
N	1.93334400	1.22361800	-0.42365400
H	-2.58548000	-0.28623000	0.76110700
Br	2.23450200	-2.33589600	-1.04780700
Br	-1.21800600	-1.01490400	2.17545500
C	-3.07580200	-0.42243700	-1.31524200
C	-3.71305200	1.48795800	0.42403000
C	-4.73831900	1.98807600	-0.60891000
C	-4.36369400	1.68165800	-2.06126000
C	-4.13190500	0.18130200	-2.25813200
H	-5.71721700	1.54602800	-0.38914700
H	-4.85277300	3.06616800	-0.46126000
H	-5.16861300	2.01509700	-2.72263600
H	-3.47668900	2.24904000	-2.35618800
H	-5.08144000	-0.35035800	-2.12598600
H	-3.80920700	-0.02475400	-3.28309000
N	-3.43743200	-0.03186400	0.16100300

C	-4.51195200	-0.90662800	0.73145000
H	-4.19713300	-1.94006800	0.65492500
H	-4.61656600	-0.68704400	1.78669500
H	-5.45634800	-0.75306800	0.21146100
C	-3.07154700	-1.95671400	-1.44800800
H	-2.69488700	-2.20286900	-2.44218500
H	-2.40443900	-2.41785200	-0.71593900
H	-4.06539300	-2.39730300	-1.36345400
C	-1.64974200	0.05356600	-1.65608000
H	-0.95636400	-0.14303800	-0.83812200
H	-1.30115100	-0.50980000	-2.52304300
H	-1.58659200	1.10612100	-1.91612700
C	-2.39156500	2.27842700	0.36894200
H	-2.57258400	3.26550200	0.79911100
H	-1.61846100	1.78768400	0.96343900
H	-2.01272700	2.43194300	-0.63601100
C	-4.26878200	1.66877200	1.84915200
H	-3.60886600	1.21313600	2.59128000
H	-4.31501900	2.73980200	2.05454400
H	-5.27911000	1.27651200	1.97046200

$\mathbf{b1_{(py)}}$

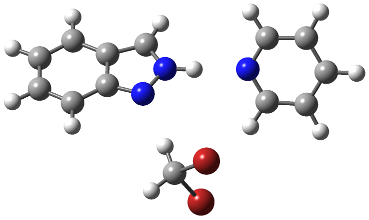

C	-0.58061700	-2.19560000	-0.20564800
H	-0.15167600	-3.17896700	-0.35334300
H	0.14728900	-1.39013500	-0.27520200
C	3.08243700	0.07862200	-0.21418800
C	3.69711300	1.32688800	0.13790100
C	5.10510200	1.42225800	0.25508100
C	5.85358500	0.29341000	0.02273200
C	5.24013500	-0.94536800	-0.32803900
C	3.87727300	-1.07008700	-0.44926900
C	2.61818600	2.20529100	0.28441000
H	5.57982100	2.36037100	0.52060800
H	6.93373100	0.33670700	0.10479000
H	5.87354900	-1.80802100	-0.50311300
H	3.41591900	-2.01292400	-0.71866800
H	2.56699000	3.25042000	0.54451600
N	1.73894700	0.19634500	-0.27429900
N	1.50863200	1.48550600	0.03058500
H	0.53650000	1.83643200	0.04479000
Br	-1.91763500	-1.93209200	-1.60588700
Br	-1.32327500	-2.17005100	1.60842000
C	-1.60942000	3.59760200	-0.62642000
C	-2.14927500	1.92105100	0.84575200
C	-2.91245600	4.08591700	-0.62912100
H	-0.84061900	4.07124400	-1.22956100
C	-3.47699500	2.33585600	0.90646200
H	-1.81285400	1.05760200	1.41065800
C	-3.86540400	3.44075300	0.15496800
H	-3.16915300	4.94729300	-1.23365600
H	-4.18424500	1.79776300	1.52543400
H	-4.89119800	3.79085600	0.17665400
N	-1.22617300	2.53768900	0.09653100

$\mathbf{TS_{b1\text{-}2(py)}}$

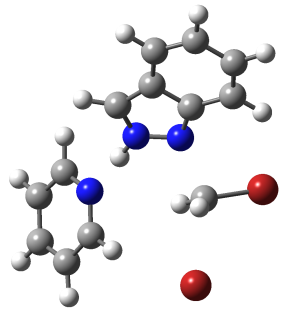

C	-0.16749600	-1.10705800	0.01642100
H	-0.48869800	-1.20665000	-1.00159100
H	0.70796400	-0.57476700	0.32694900
C	-2.60003600	0.77916300	0.00580000
C	-2.76026600	2.10321900	-0.51125900
C	-4.04457200	2.58991100	-0.84788900
C	-5.11857700	1.74994000	-0.67479500
C	-4.95041400	0.43036700	-0.16908800
C	-3.71640700	-0.07014600	0.17660100
C	-1.45778700	2.62266700	-0.55846300
H	-4.17480700	3.59499800	-1.23191200
H	-6.11598600	2.09118500	-0.92567100
H	-5.82680100	-0.19681400	-0.05160700
H	-3.59833500	-1.07243100	0.56571600
H	-1.07978900	3.58273300	-0.87234200
N	-1.28844600	0.54764500	0.27264800
N	-0.63723400	1.66934500	-0.10266900
H	0.40930000	1.73259500	-0.00190400
Br	-0.86177900	-2.34242000	1.26271800
Br	1.80563600	-2.44510100	-1.13524600
C	2.64647100	2.93946200	0.90097800
C	3.05562700	1.10405100	-0.42987400
C	4.00344700	3.18992800	1.06372300
H	1.90596600	3.57464300	1.37819000
C	4.43269300	1.27778700	-0.31598000
H	2.65417600	0.26698000	-0.99477900
C	4.91539300	2.34008200	0.44020800
H	4.33301800	4.02666300	1.66728000
H	5.09950500	0.58116500	-0.80826900
H	5.98187500	2.50201600	0.54832200
N	2.17270400	1.92082100	0.16742000

b2$_{(py)}$

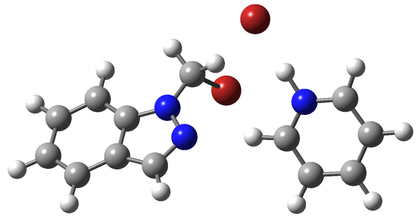

C	-0.93368600	-1.10814900	0.41030100
H	-1.50607000	-2.02628100	0.34444300
H	0.06973100	-1.24049800	0.01163200
C	-2.97331700	0.11105300	-0.39147100
C	-3.15399200	1.37797700	-0.98820200
C	-4.44594500	1.82266500	-1.31197900
C	-5.51523700	0.99458300	-1.01970100
C	-5.31658900	-0.26464800	-0.41201900
C	-4.05204100	-0.72799500	-0.08760200
C	-1.82969100	1.90265100	-1.10694000
H	-4.60094700	2.78933900	-1.77726500
H	-6.52381200	1.31218900	-1.25657600
H	-6.17814000	-0.88430800	-0.19138800
H	-3.91350300	-1.69196700	0.38565400

H	-1.51694300	2.85162700	-1.51812800
N	-1.61381800	-0.04882200	-0.22436800
N	-0.93279400	1.06063300	-0.64339200
H	2.79720600	-0.42593000	-0.77624300
Br	-0.69333400	-0.81207900	2.40507900
Br	2.19211300	-2.15278200	-1.38795100
C	2.36142600	1.54848600	-0.12324600
C	4.52079800	0.62084400	-0.18132100
C	2.86124200	2.73916100	0.38527000
H	1.30727300	1.37014200	-0.32426200
C	5.07564800	1.78496100	0.32225300
H	5.09027700	-0.26651600	-0.42975900
C	4.23103700	2.85796200	0.60920500
H	2.18209900	3.55248100	0.60446600
H	6.14308400	1.84677000	0.48791600
H	4.63991100	3.77917400	1.00763300
N	3.20013300	0.53770400	-0.38865600

b4$_{(py)}$

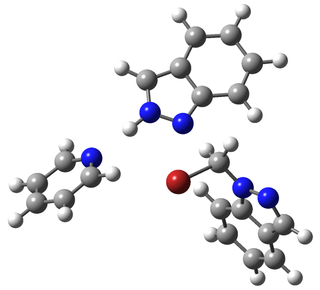

C	-1.78506700	-0.35758500	1.62590300
C	-2.76261300	-0.47331700	0.63202800
C	-2.19530500	-0.59593600	2.92923000
C	-4.10266100	-0.80447700	0.92311600
C	-3.52961200	-0.93313500	3.24188800
H	-1.47101100	-0.51498900	3.73189200
C	-4.49218300	-1.03552600	2.25119800
C	-4.75400700	-0.80517200	-0.35407400
H	-3.80128900	-1.10814100	4.27646100
H	-5.51810500	-1.28736100	2.49453900
H	-5.79187000	-1.00369200	-0.58007000
N	-3.92234900	-0.51310700	-1.32463200
N	-2.70292900	-0.28566400	-0.73826700
C	-1.56636700	-0.12085400	-1.54044900
H	-0.76176200	0.38336200	-1.01240300
H	-1.83079100	0.34230400	-2.48294200
H	-0.76662700	-0.07147900	1.39223000
C	0.76698900	2.92332900	0.04640500
C	2.04221800	3.55577300	-0.13390100
C	2.12711500	4.95985700	-0.29633100
C	0.96114200	5.68700100	-0.27418600
C	-0.30452900	5.05594000	-0.09244000
C	-0.42026900	3.69602900	0.06741400
C	2.95363700	2.49468700	-0.09595500
H	3.08546600	5.44810600	-0.43447100
H	0.99540800	6.76374200	-0.39551900
H	-1.19618100	5.67298100	-0.07884600
H	-1.38421500	3.22231000	0.21199300
H	4.02757800	2.46123500	-0.18655000
N	0.89958400	1.58516700	0.18006500
N	2.22588300	1.37728200	0.08815600
H	2.59755400	0.41750900	0.18758400
Br	-0.71820400	-1.89931000	-2.12268600
C	2.84648600	-2.35201700	-0.19817800
C	4.41311200	-1.50954500	1.25467700
C	3.33475400	-3.64752000	-0.04636900
H	1.99855700	-2.15086700	-0.84514800
C	4.96964300	-2.76696400	1.46732700

H	4.81366100	-0.63598200	1.76057100
C	4.41779400	-3.85857300	0.80141700
H	2.86946100	-4.46607000	-0.58163900
H	5.81151000	-2.88286500	2.13902700
H	4.82326500	-4.85403700	0.94290900
N	3.37336300	-1.29684800	0.43783000

$TS_{b4\text{-}5(py)}$

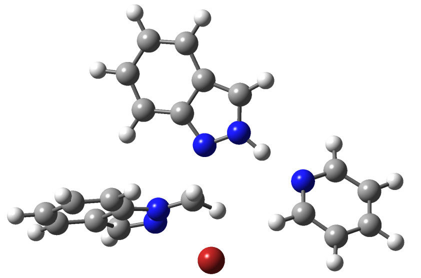

C	2.75098700	-1.34363900	-1.16720800
C	2.45164500	-1.22873600	0.18839700
C	4.04789800	-1.72600800	-1.48458700
C	3.38787900	-1.50928600	1.19856200
C	5.00766500	-1.99336900	-0.48911200
H	4.32368100	-1.83628500	-2.52677300
C	4.69017500	-1.89563400	0.85750700
C	2.65875700	-1.32706500	2.42722100
H	6.00526000	-2.29534100	-0.78556200
H	5.42395900	-2.12222300	1.62215500
H	3.01502900	-1.45070600	3.44022100
N	1.41884800	-0.97566900	2.21921900
N	1.26536200	-0.90263200	0.84636900
C	0.08459000	-0.52778000	0.31517000
H	-0.02363200	-0.58153700	-0.75191700
H	-0.77538800	-0.61953100	0.95392000
H	2.00691000	-1.17931500	-1.93534100
C	0.91584200	2.44380600	0.08995000
C	0.30330400	3.55157600	-0.57664700
C	1.07079400	4.68380400	-0.93521000
C	2.41106400	4.67877800	-0.62982000
C	3.01374000	3.57311800	0.03320600
C	2.29423100	2.45964800	0.40080200
C	-1.04363900	3.18035300	-0.70767800
H	0.61304900	5.53007000	-1.43426900
H	3.02698100	5.53101000	-0.89184100
H	4.07336300	3.61636600	0.25810900
H	2.76237400	1.63082200	0.91517300
H	-1.89004900	3.69314500	-1.13638200
N	-0.01743500	1.49414300	0.35181900
N	-1.17330200	1.96672900	-0.15946000
H	-2.07109400	1.41572400	-0.05490800
Br	-1.00586300	-2.94043100	-0.88782500
C	-3.86453200	-0.63115900	-0.22856900
C	-4.64110600	1.33357400	0.69479300
C	-5.10921800	-1.23855000	-0.07565600
H	-3.02907700	-1.19217600	-0.64371500
C	-5.91338900	0.80737400	0.87920600
H	-4.41506600	2.35178000	0.99838100
C	-6.15143300	-0.50813800	0.48351100
H	-5.23747100	-2.26729500	-0.38821200
H	-6.69291900	1.41316900	1.32478000
H	-7.13110900	-0.95367500	0.61518300
N	-3.63220600	0.63672600	0.14945700

$b5_{(py)}$

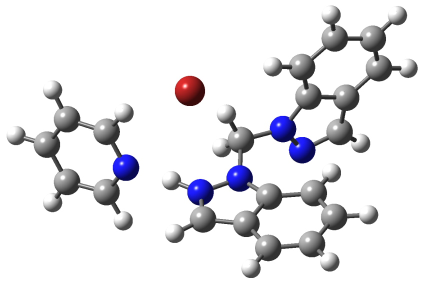

C	-2.47084800	-1.75582100	0.91484700
C	-2.45494100	-1.42782200	-0.44776900
C	-3.64174000	-2.31897200	1.39449000
C	-3.56181500	-1.64397900	-1.29853500
C	-4.76135000	-2.54949300	0.56234500
H	-3.69899600	-2.59100500	2.44223400
C	-4.73632600	-2.21809400	-0.78001500
C	-3.12659100	-1.17717100	-2.57909500
H	-5.65234600	-2.99480100	0.98987000
H	-5.59576100	-2.39678000	-1.41615100
H	-3.66781400	-1.17074900	-3.51433100
N	-1.89290800	-0.72250400	-2.53830400
N	-1.47470400	-0.86315100	-1.24215200
C	-0.15051600	-0.50598800	-0.87411500
H	0.13147000	-0.98254900	0.07704600
H	0.53881600	-0.78439300	-1.67143600
H	-1.61376000	-1.57666400	1.55813700
C	-0.85638600	1.95629500	-0.31316100
C	-0.08285700	3.05335000	0.16255900
C	-0.70847700	4.25059900	0.56813000
C	-2.07955300	4.32098400	0.49233700
C	-2.83936100	3.22350200	0.01346500
C	-2.25940200	2.04176500	-0.39624500
C	1.25413600	2.62132200	0.10789400
H	-0.11958200	5.08332400	0.93323500
H	-2.59376200	5.22277700	0.80187500
H	-3.91832100	3.31647200	-0.03173200
H	-2.86312900	1.22610200	-0.76221500
H	2.17149900	3.11297100	0.38878100
N	0.01898400	0.96130000	-0.65692700
N	1.27187400	1.38682800	-0.36791400
H	2.14230400	0.74485600	-0.49065000
Br	1.00139100	-0.73527200	2.38519000
C	3.92187400	-0.93008800	0.26964500
C	4.31483700	0.07516100	-1.77654500
C	5.11627700	-1.64200400	0.18552000
H	3.24009400	-1.02662200	1.11497000
C	5.51936600	-0.59728000	-1.93622700
H	3.95883100	0.76659200	-2.53465600
C	5.92708800	-1.47427400	-0.93181300
H	5.39301700	-2.31419300	0.98814500
H	6.11827600	-0.43706300	-2.82415300
H	6.86135900	-2.01692400	-1.02282000
N	3.53084600	-0.08515600	-0.69956800

[**L2**-Hpy]$^+$

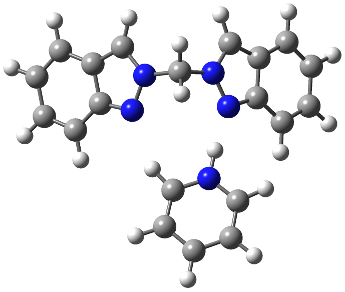

C	-2.47431900	-1.14357800	-0.18117700
C	-2.32638400	-2.52405900	0.17006100
C	-3.19713200	-3.50354200	-0.36962500
C	-4.17416500	-3.08535600	-1.23646300
C	-4.31536100	-1.71065000	-1.58889100
C	-3.49008800	-0.73786600	-1.08052100
C	-1.24905600	-2.54346500	1.05436800
H	-3.09002300	-4.54881100	-0.10622100
H	-4.85570400	-3.80683800	-1.67017000
H	-5.09899100	-1.43264100	-2.28405800
H	-3.60493500	0.29969600	-1.37096000
H	-0.77134100	-3.35323700	1.58264800
N	-1.55189100	-0.38594700	0.45340700
N	-0.82836600	-1.26706400	1.19040100
C	0.32189500	-0.81440300	1.96317200
H	0.45955000	-1.50065600	2.79717300
H	0.10701300	0.18109100	2.34612100
C	2.97739500	0.01318500	-0.17990700
C	3.46388600	-1.27128900	0.23912800
C	4.69362300	-1.77384500	-0.25873200
C	5.39043000	-0.99769000	-1.14804500
C	4.90411900	0.27881800	-1.56504800
C	3.72092000	0.79440300	-1.10057500
C	2.49936600	-1.73069600	1.13068400
H	5.06791100	-2.74114500	0.05441300
H	6.33252000	-1.35153700	-1.54913800
H	5.49461500	0.85145200	-2.27085900
H	3.36265400	1.76398600	-1.42507000
H	2.42829200	-2.63752300	1.71002300
N	1.80520000	0.31229200	0.41488500
N	1.54964100	-0.76278800	1.19888200
H	-1.33279800	1.29405600	0.32274700
C	-0.08740100	2.94160300	0.04281200
C	-2.43151800	3.06265700	0.28706400
C	-0.00964700	4.31830600	-0.09465100
H	0.75916100	2.26103800	0.03305800
C	-2.40757800	4.43838800	0.15442500
H	-3.33883400	2.49422600	0.44499400
C	-1.17962400	5.07340100	-0.04083300
H	0.95492000	4.78635100	-0.23944800
H	-3.33112100	4.99943000	0.20445300
H	-1.13766000	6.15093600	-0.14718800
N	-1.28621400	2.35839200	0.22585700

TS$_{L2-3}$

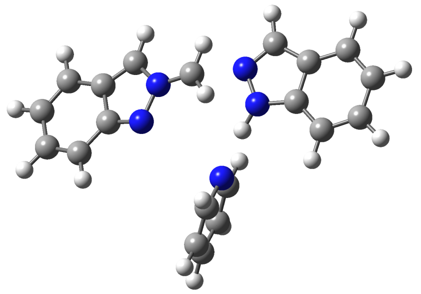

C	3.17258500	-0.24708900	-0.11702800
C	3.78343700	-1.33906100	0.54933700
C	5.17026800	-1.53975300	0.44416100
C	5.89958700	-0.64734200	-0.31948300
C	5.27653500	0.44326500	-0.97405000
C	3.91532800	0.66410900	-0.88765600
C	2.70726400	-1.97633200	1.23897500
H	5.65492900	-2.36699900	0.94884300
H	6.97027400	-0.77677200	-0.42127900
H	5.88826400	1.12294400	-1.55553400
H	3.44591900	1.50313800	-1.38627600
H	2.74051300	-2.84450200	1.88197300
N	1.83366600	-0.31763700	0.15385900
N	1.56413100	-1.35514500	1.02795200
C	0.03646500	-2.08795100	-1.04391200
H	0.56475900	-2.88616800	-0.54886500
H	0.45089000	-1.54333100	-1.87985400
C	-3.09892600	-0.88064400	-0.79208300
C	-3.15769200	-1.85414800	0.31189800
C	-4.35836700	-2.00367500	1.07837400
C	-5.41886400	-1.22458400	0.74177500
C	-5.35822800	-0.27102000	-0.34964300
C	-4.24272400	-0.08677200	-1.10733300
C	-1.93201300	-2.43710600	0.33781700
H	-4.40886200	-2.71601600	1.89187600
H	-6.34703900	-1.30776000	1.29392300
H	-6.24737400	0.31166400	-0.56078100
H	-4.20530900	0.62274900	-1.92328600
H	-1.47590400	-3.18211900	0.96861600
N	-1.92626900	-0.86352300	-1.39182600
N	-1.19449300	-1.83391600	-0.70559700
H	1.15674900	0.47184800	0.09984600
C	0.09262900	2.62218600	1.35111700
C	-0.44133500	2.61841000	-0.88356000
C	-0.50391800	3.86448400	1.54297700
H	0.56545400	2.10169400	2.17825400
C	-1.05745300	3.86215400	-0.78877500
H	-0.40082000	2.09254600	-1.83215900
C	-1.08904900	4.49781000	0.45000100
H	-0.50183000	4.32210000	2.52441100
H	-1.49464300	4.32041200	-1.66735200
H	-1.55709100	5.46911100	0.56026500
N	0.12695900	2.00219700	0.16274300

[**L3**-Hpy]$^+$

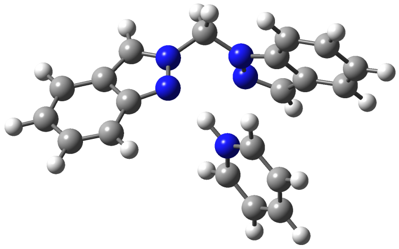

C	2.03561400	-1.46777500	-0.21447200
C	2.91716500	-1.03767700	0.80438500
C	4.23011400	-0.65520500	0.47958100
C	4.62159700	-0.71053300	-0.84628500
C	3.72840600	-1.14289200	-1.85122300
C	2.42809400	-1.52591900	-1.55942900
C	2.15167700	-1.14375300	2.00863700
H	4.92239400	-0.33884400	1.25119000
H	5.63188400	-0.43423200	-1.12283200
H	4.07356200	-1.18974200	-2.87762900
H	1.76390100	-1.86955700	-2.34360100
H	2.45614200	-0.92885200	3.02284500
N	0.84666400	-1.76903800	0.41951900
N	0.93626700	-1.58588900	1.77213300
C	-0.31079600	-2.41778200	-0.12638000
H	-0.52619000	-3.33534300	0.42201700
H	-0.10659900	-2.66020500	-1.16960500
C	-2.82291900	0.06381100	-0.45911300
C	-3.62748600	-0.97074300	0.11239000
C	-5.01487100	-0.77629000	0.31707300
C	-5.55447900	0.43123600	-0.05121500
C	-4.75020500	1.45839600	-0.62353400
C	-3.40028300	1.30003800	-0.83241600
C	-2.73087900	-2.01603000	0.34960000
H	-5.62906400	-1.55787900	0.74762400
H	-6.61309700	0.61376300	0.08710500
H	-5.22561700	2.38979700	-0.90862500
H	-2.80680900	2.08448600	-1.28699200
H	-2.87304800	-3.00181500	0.76321500
N	-1.52879300	-0.33346200	-0.56040400
N	-1.51876700	-1.59297700	-0.05403500
H	-0.30955200	0.77752300	-0.35030700
C	0.32502200	2.10938600	1.11313800
C	1.20608600	2.03909600	-1.06878700
C	1.16098400	3.15953000	1.44920200
H	-0.38395700	1.66410000	1.79855200
C	2.06530400	3.08612400	-0.78741200
H	1.16525200	1.54278800	-2.02888600
C	2.04018500	3.65398500	0.48628400
H	1.12050100	3.57982300	2.44517000
H	2.73894700	3.44731200	-1.55273900
H	2.70228600	4.47728100	0.72683000
N	0.36592600	1.58387800	-0.12342400

TS$_{L3-1}$

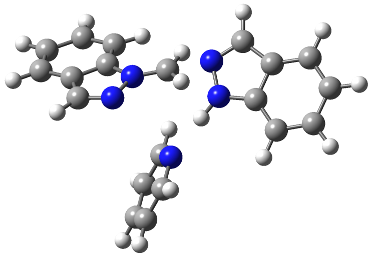

C	-2.97662800	-0.18564200	0.00289100
C	-3.54101300	-1.23918200	-0.75356900
C	-4.89968300	-1.56560500	-0.60132400
C	-5.65034100	-0.82635200	0.29480800
C	-5.07388500	0.23020500	1.03593600
C	-3.73737400	0.56836400	0.90801400
C	-2.45926300	-1.71151700	-1.55902600
H	-5.34815300	-2.36863500	-1.17419800
H	-6.70084000	-1.05219500	0.43189500
H	-5.69845600	0.79147800	1.72105400
H	-3.30620900	1.38334100	1.47663300
H	-2.46149400	-2.50722600	-2.29049100
N	-1.64692200	-0.12272900	-0.34155100
N	-1.35163600	-1.03107000	-1.32791300
C	0.10020300	-1.94973400	1.18890300
H	-0.37428100	-2.15497900	0.23688900
H	-0.42705600	-1.97225100	2.13421800
C	2.29921600	-1.60521900	0.14071100
C	3.53944000	-1.37033000	0.74200100
C	4.68177300	-1.24657800	-0.04206500
C	4.52710600	-1.35942000	-1.42548300
C	3.27029500	-1.57477200	-2.00365100
C	2.11351000	-1.69928600	-1.22667100
C	3.26364200	-1.29841600	2.16766100
H	5.65447900	-1.06786400	0.39910400
H	5.39525900	-1.27075000	-2.06701200
H	3.18696600	-1.64157600	-3.08154900
H	1.13409400	-1.82598000	-1.67284600
H	3.97156700	-1.12266200	2.96769900
N	1.36268600	-1.68729600	1.21731500
N	2.01309300	-1.46731700	2.46172600
H	-1.00779400	0.68115700	-0.20158000
C	0.33716300	2.91103500	1.02918800
C	0.46538100	2.72602200	-1.25633700
C	0.96695100	4.15151300	1.01479500
H	0.01382200	2.46950100	1.96718600
C	1.10120500	3.95848800	-1.36990600
H	0.24092200	2.13522500	-2.13904400
C	1.35649400	4.68587900	-0.21064700
H	1.13921300	4.68472300	1.94155100
H	1.38020100	4.33788800	-2.34508500
H	1.84463300	5.65217000	-0.26124500
N	0.08820400	2.20154300	-0.08108500

[**L1**-Hpy]$^+$

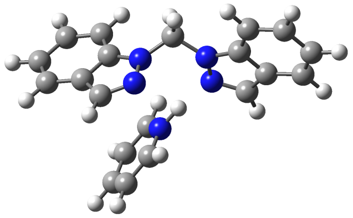

C	-3.18646900	-0.38802900	-0.12319900
C	-3.59910100	0.94762700	-0.34842800
C	-4.95343200	1.30531300	-0.20118000
C	-5.84868800	0.32050400	0.15949000
C	-5.42122600	-1.01133300	0.37657700
C	-4.09913800	-1.39110300	0.24020000
C	-2.40747300	1.63605900	-0.69173900
H	-5.28002500	2.32481600	-0.36773200
H	-6.89756500	0.56168600	0.28008200
H	-6.15493200	-1.75669900	0.65996100
H	-3.79638900	-2.41538900	0.41592600
H	-2.27432400	2.68215900	-0.92742800
N	-1.82731000	-0.41550200	-0.32436000
N	-1.36674000	0.81659800	-0.68872100
C	-0.97667100	-1.58714800	-0.43338600
H	-0.75209600	-1.79836800	-1.48082100
H	-1.53716500	-2.42542900	-0.01879000
C	1.54919100	-1.65310100	-0.23488300
C	2.40972100	-1.64604100	0.88795500
C	3.79200800	-1.83454800	0.71434600
C	4.27275800	-2.02335400	-0.56878900
C	3.39960400	-2.03104600	-1.67896600
C	2.03303900	-1.84779900	-1.53709100
C	1.53839800	-1.44393100	2.00373800
H	4.46154800	-1.84521300	1.56662700
H	5.33181600	-2.18353400	-0.73065100
H	3.80861300	-2.19904600	-2.66854900
H	1.37946700	-1.87592900	-2.40079500
H	1.78722400	-1.39208500	3.05409300
N	0.27904100	-1.43569900	0.26106800
N	0.28308000	-1.33507600	1.62422600
H	0.20579000	1.37355500	-0.43690100
C	1.40841900	2.07571800	1.10289500
C	1.97928200	2.19306100	-1.17990800
C	2.58903200	2.70384200	1.45905000
H	0.66546200	1.74163000	1.81498300
C	3.17381000	2.82174600	-0.88004500
H	1.66554400	1.95680300	-2.18792600
C	3.48033100	3.08204700	0.45592400
H	2.80235800	2.89141100	2.50283700
H	3.84785100	3.10217700	-1.67822000
H	4.40933600	3.57695900	0.71281800
N	1.13580400	1.84247800	-0.19291100

TS$_{L3-1}$ (without Hpy$^+$ cation)

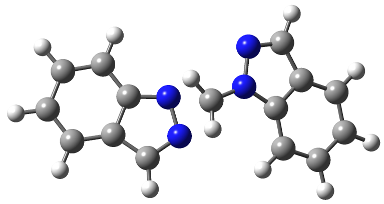

C	-2.53711300	0.31508700	-0.30570500
C	-3.05741700	-0.98244200	-0.00324200
C	-4.45051000	-1.19833200	0.01390600
C	-5.28166400	-0.12196500	-0.22504800

C	-4.76196800	1.17316900	-0.50130000
C	-3.40550900	1.40589500	-0.54866300
C	-1.89481600	-1.77147500	0.16843700
H	-4.86299400	-2.18076900	0.21751200
H	-6.35730900	-0.25970600	-0.20378100
H	-5.45529500	1.98666400	-0.68483900
H	-3.00232000	2.38678100	-0.77318300
H	-1.81085700	-2.82733600	0.38649800
N	-1.18575500	0.29171700	-0.32861700
N	-0.81063600	-1.02746900	-0.04762000
C	0.26603700	0.18064400	1.46406900
H	0.31693900	-0.79150900	1.92062600
H	-0.41435900	0.92433700	1.84415100
C	2.45526600	-0.07640800	0.36550500
C	3.31852100	0.87388600	-0.20261900
C	4.50383200	0.46233900	-0.81926000
C	4.78123200	-0.89826800	-0.85257600
C	3.89350800	-1.83495900	-0.29313700
C	2.70957800	-1.44316100	0.32267100
C	2.66033000	2.13874900	0.01994600
H	5.18170300	1.18113700	-1.26419500
H	5.69038500	-1.24963000	-1.32584400
H	4.13200300	-2.89034000	-0.35392000
H	2.01204000	-2.16807600	0.72019300
H	2.99862500	3.12495100	-0.26582900
N	1.38078100	0.64723200	0.89598400
N	1.52640900	2.00665500	0.64664000

TS$_{\mathbf{L2\text{-}3}}$ (without Hpy^+ cation)

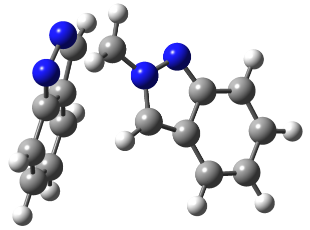

C	-2.37387900	-0.02066400	-0.33433500
C	-1.99794400	0.21565400	1.02607200
C	-1.95940100	1.53050200	1.52986100
C	-2.24956600	2.56834500	0.66644500
C	-2.60231200	2.33451300	-0.69229100
C	-2.67408400	1.05835500	-1.20308800
C	-1.79272200	-1.08689300	1.54429600
H	-1.69887200	1.72699500	2.56394300
H	-2.21241900	3.59071900	1.02637800
H	-2.83083100	3.18376900	-1.32665900
H	-2.96848900	0.87124700	-2.22979100
H	-1.49592500	-1.38721500	2.53889700
N	-2.40358900	-1.34193900	-0.59571900
N	-2.04332900	-1.99393300	0.60986600
C	-0.45594300	-2.47234200	-0.83007200
H	-0.90307900	-2.52130500	-1.80631000
H	-0.38327300	-3.35810100	-0.22392200
C	2.21749700	-0.66717800	0.30198400
C	1.87848800	0.17399900	-0.82757800
C	2.64369500	1.34119300	-1.10756700
C	3.69719200	1.62942300	-0.28513600
C	4.03296800	0.79159900	0.83156400
C	3.32188700	-0.33628700	1.13602400
C	0.78316800	-0.43621300	-1.39782300
H	2.39378300	1.97958900	-1.94658400
H	4.29983700	2.51106200	-0.46983400
H	4.87928600	1.07197900	1.44859700
H	3.57454600	-0.96665400	1.97947400
H	0.16207300	-0.18686000	-2.24132600
N	1.39576100	-1.70821400	0.41669500

N	0.53245700	-1.56438200	-0.63984300

[$CH_2py_2]^{2+}$

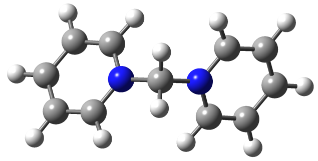

C	2.10586700	1.01031600	0.61715600
C	1.52930300	-1.14679800	-0.17226100
C	3.29406800	0.97310000	-0.08445000
H	1.82596100	1.84833800	1.24240600
C	2.70318700	-1.22234600	-0.89056400
H	0.80765300	-1.95289000	-0.15279100
C	3.60174900	-0.15226700	-0.84989700
H	3.97006300	1.81662100	-0.02009200
H	2.91506500	-2.11631300	-1.46364500
H	4.53355900	-0.20010700	-1.40209900
N	1.23658400	-0.03668300	0.56140300
C	0.00001400	-0.00029000	1.37834500
H	-0.03431700	-0.88475500	2.01146500
H	0.03424100	0.88393900	2.01180900
C	-2.10593300	-1.01051300	0.61681800
C	-1.52917900	1.14676800	-0.17204700
C	-3.29417400	-0.97296900	-0.08469600
H	-1.82610400	-1.84872900	1.24184600
C	-2.70312700	1.22264200	-0.89023500
H	-0.80742700	1.95277000	-0.15248300
C	-3.60180300	0.15266000	-0.84979600
H	-3.97026200	-1.81643600	-0.02054800
H	-2.91492600	2.11680500	-1.46304500
H	-4.53368700	0.20075700	-1.40185500
N	-1.23653400	0.03642200	0.56128100

TS_{L3a-b}

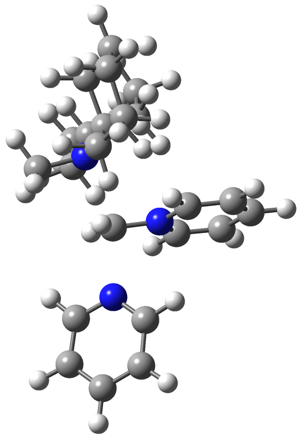

C	1.06861700	-0.12597100	-0.38396600
H	0.99566600	-1.15594000	-0.66151600
H	1.12018800	0.68216300	-1.08481300
N	-1.10590300	-0.15284300	-1.02948600
C	-0.84780000	-0.48277300	-2.44985400
H	-0.19396000	0.25796600	-2.90588500
H	-0.36931300	-1.45590400	-2.53698000
H	-1.77716100	-0.50415700	-3.02635300
C	-1.79193400	1.20658500	-0.92090400
C	-2.22088300	1.57755700	0.50990900
C	-0.97499400	2.36185800	-1.53350500
H	-2.70155200	1.11851600	-1.52817400
C	-3.02878400	2.88884700	0.52082200

H	-1.33520500	1.70241300	1.14381500
H	-2.82774100	0.79361600	0.96317400
C	-1.78840800	3.67013800	-1.53039600
H	-0.05591700	2.52776400	-0.95506000
H	-0.67794900	2.14726500	-2.56115300
C	-2.26177700	4.04581300	-0.12411000
H	-3.30232300	3.13191800	1.55074800
H	-3.97144600	2.72999500	-0.01561200
H	-1.18225800	4.46986700	-1.96303200
H	-2.65436000	3.54916100	-2.19115900
H	-2.89009500	4.93853500	-0.16472200
H	-1.39560200	4.30912500	0.49796200
C	-1.87944600	-1.26092200	-0.33276100
C	-3.37437400	-1.34007800	-0.73482300
C	-1.26074800	-2.66393800	-0.50144900
H	-1.84217400	-1.00579300	0.73101300
C	-4.11729200	-2.37260100	0.13163300
H	-3.45149700	-1.62687600	-1.78927400
H	-3.86659800	-0.37302000	-0.63042800
C	-1.98040300	-3.69157100	0.39257800
H	-1.35344000	-2.99590800	-1.53897300
H	-0.18729300	-2.67975400	-0.27438700
C	-3.47511000	-3.76009700	0.06075600
H	-5.16038300	-2.41788800	-0.19076300
H	-4.13391800	-2.02544100	1.17266700
H	-1.51269700	-4.67043100	0.26054800
H	-1.85115700	-3.42324600	1.45000400
H	-3.98046200	-4.44751800	0.74306900
H	-3.60224900	-4.17365500	-0.94644700
C	3.92714400	-0.86251800	0.37831600
C	3.65882400	0.09011600	-1.71991800
C	5.27995400	-1.07962000	0.15222800
H	3.46517900	-1.15821300	1.31444700
C	5.00296500	-0.09002700	-2.01957400
H	2.98242700	0.54278900	-2.43813800
C	5.82736800	-0.68589400	-1.06766600
H	5.88535000	-1.54992300	0.91671000
H	5.38779600	0.22939700	-2.97977000
H	6.87946300	-0.84326000	-1.27340800
N	3.14013900	-0.28378700	-0.54103400
C	1.32455900	1.46249600	1.40727600
C	0.65596900	-0.75185600	1.91314700
C	1.29648300	1.79984500	2.74186900
H	1.61132100	2.15791200	0.63174500
C	0.61209500	-0.45172700	3.25895300
H	0.42656300	-1.73223300	1.52208300
C	0.93196400	0.83735100	3.68675200
H	1.56272000	2.80711800	3.03573200
H	0.33485400	-1.22724400	3.96146400
H	0.90469900	1.08740800	4.74100500
N	0.99670400	0.20197300	1.00023100

L3b

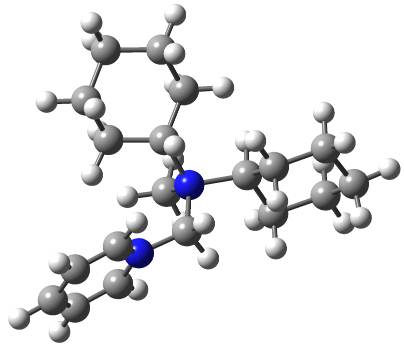

C	0.30939100	-1.24953000	-0.36794300
H	-0.11355700	-2.20537900	-0.07155700
H	0.04580500	-1.05088400	-1.40117600
N	-0.35500700	-0.18793800	0.48118000
C	-0.05234400	-0.37718300	1.94214600
H	-0.31004600	-1.38700200	2.24761900
H	0.99874200	-0.18644500	2.13484100
H	-0.65239300	0.33019300	2.51099700
C	-1.94234000	-0.42503100	0.26017300
C	-2.37066700	-0.23630700	-1.19993700
C	-2.47098300	-1.75882400	0.80835100
H	-2.35388500	0.37379500	0.87364600
C	-3.91396500	-0.21366600	-1.27747600
H	-2.01882200	-1.07214600	-1.81499800
H	-1.97515500	0.68381500	-1.63324000
C	-4.01783600	-1.74888200	0.72515500
H	-2.10367300	-2.60871600	0.22192300
H	-2.19259500	-1.92076700	1.85017700
C	-4.52049300	-1.49433200	-0.69815000
H	-4.20679300	-0.08189000	-2.32124800
H	-4.29200500	0.66127900	-0.73597500
H	-4.38455500	-2.70604300	1.10200300
H	-4.40539000	-0.98041500	1.40362900
H	-5.61045300	-1.42595700	-0.69976600
H	-4.26389600	-2.34589000	-1.33950300
C	0.06141600	1.29246300	0.02625800
C	-0.97908900	2.36531700	0.38753200
C	1.43270300	1.72411500	0.57788300
H	0.11267800	1.20374000	-1.06131000
C	-0.55735800	3.72833300	-0.21010000
H	-1.05835600	2.45720400	1.47646900
H	-1.96894700	2.13072600	0.00189800
C	1.86352700	3.05960700	-0.06440000
H	1.36154200	1.88095700	1.65770300
H	2.21075200	0.97818300	0.41407300
C	0.84193500	4.16044400	0.22799000
H	-1.30237800	4.47078500	0.08370600
H	-0.60200700	3.66561000	-1.30412100
H	2.84770400	3.32990100	0.32527300
H	1.97947200	2.93337500	-1.14811600
H	1.12536000	5.08391500	-0.28138400
H	0.84363900	4.38438900	1.30086800
C	2.35750400	-2.16003500	0.65585100
C	2.54479600	-0.87032900	-1.31274800
C	3.71912200	-2.38223300	0.66837300
H	1.69502100	-2.59620800	1.39019900
C	3.90863400	-1.07421800	-1.34549100
H	2.02530500	-0.30405500	-2.07354500
C	4.51154300	-1.83388300	-0.34158800
H	4.14557600	-2.99712000	1.45083800
H	4.48535000	-0.65098800	-2.15815200
H	5.58095100	-2.01083400	-0.35561900
N	1.78415600	-1.39378700	-0.31224500

TS$_{L3b-c}$

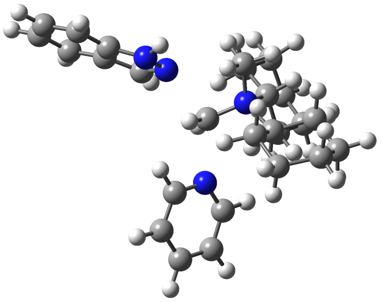

C	0.04517200	-0.13987000	0.03668400
H	0.33105100	0.20425800	1.00812300
H	0.42086300	-1.03069900	-0.42219500
N	-0.86156600	0.68962400	-0.75594900
C	-0.23386700	0.99763400	-2.10756400
H	0.63812400	1.61979600	-1.95918600
H	0.04197000	0.06532400	-2.59137300
H	-0.97834800	1.51351200	-2.70625900
C	-1.19687600	2.06811600	-0.04028500
C	-2.05463500	1.89798700	1.21517500
C	0.04035800	2.91918800	0.27591400
H	-1.77385600	2.58010500	-0.80949900
C	-2.50452600	3.27985600	1.73766100
H	-1.46825800	1.40343300	1.99824700
H	-2.94095300	1.29224700	1.02354500
C	-0.41097600	4.30758900	0.77957300
H	0.63635000	2.44336700	1.06077400
H	0.68586800	3.05074300	-0.59161700
C	-1.31403100	4.20180600	2.01121900
H	-3.09958500	3.13433400	2.64213500
H	-3.17192200	3.74119900	1.00041700
H	0.47889600	4.90129900	1.00048200
H	-0.93715900	4.83033400	-0.02747400
H	-1.66772300	5.19274900	2.30404800
H	-0.73637700	3.81638700	2.86030200
C	-2.24935700	-0.15045800	-1.08149000
C	-3.32255900	0.71873300	-1.73766400
C	-1.98081100	-1.40415100	-1.91474800
H	-2.56610800	-0.43875900	-0.08605400
C	-4.62807900	-0.10951900	-1.83965500
H	-3.02317400	1.02021800	-2.74557800
H	-3.53594900	1.62175700	-1.16707800
C	-3.28142300	-2.23603900	-2.00147500
H	-1.68383000	-1.13383400	-2.93147800
H	-1.19172000	-2.02452600	-1.48625400
C	-4.42548800	-1.41816100	-2.60677500
H	-5.38469700	0.51228600	-2.32314800
H	-5.00066300	-0.32134900	-0.83051100
H	-3.08130000	-3.12756200	-2.60011800
H	-3.55824100	-2.58656400	-1.00042800
H	-5.34873300	-2.00189600	-2.59973400
H	-4.20526000	-1.20008400	-3.65820200
C	4.13601800	-0.39509400	-1.00839600
C	4.25921400	0.23553600	0.25317500
C	5.50816600	0.27016500	0.90943200
C	6.58050000	-0.31620300	0.27343700
C	6.43837400	-0.92901000	-0.99688700
C	5.22549600	-0.98111800	-1.66125200
C	2.96572100	0.73620200	0.53498500
H	5.61941100	0.74512400	1.87634500
H	7.55591000	-0.30882600	0.74351300
H	7.31104900	-1.37255800	-1.46143900
H	5.13324800	-1.45341300	-2.63135200
H	2.63208900	1.26710600	1.41488100

N	2.81020200	-0.28648700	-1.37389700
N	2.12210700	0.47581700	-0.45917400
H	2.48195700	-0.30349000	-2.32875200
C	-1.88330900	-1.53521100	2.24958900
C	-0.33744300	-2.85050400	1.14292100
C	-2.18733600	-2.56573200	3.13067300
H	-2.37622600	-0.57609400	2.33513900
C	-0.58645900	-3.92953800	1.98111800
H	0.40162600	-2.93633600	0.35213400
C	-1.52964700	-3.78558300	2.99530200
H	-2.92439900	-2.40492100	3.90742500
H	-0.04646500	-4.85690900	1.83787300
H	-1.74494000	-4.60632700	3.66905700
N	-0.97870000	-1.67769200	1.26605900

L3c

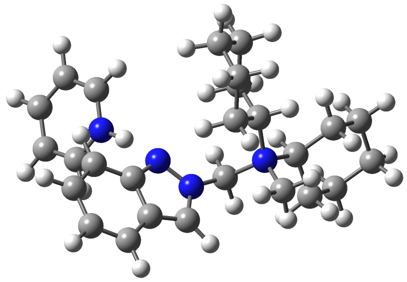

C	0.82048400	-0.08582200	-1.07793200
H	0.59399800	0.90668200	-0.70300800
H	1.13006300	-0.02376300	-2.11651900
N	2.05167300	-0.58818500	-0.30472700
C	2.47926100	-1.93390700	-0.81925100
H	1.70549100	-2.66615400	-0.62013400
H	2.66205600	-1.87453800	-1.88840500
H	3.39309200	-2.22873900	-0.30781900
C	1.82288900	-0.68552700	1.25428200
C	1.33965200	0.63883500	1.85640900
C	0.94353100	-1.85699300	1.72511500
H	2.82977000	-0.89304700	1.61178600
C	1.37577600	0.56132000	3.39653100
H	0.30989600	0.82774500	1.53651500
H	1.95353500	1.48071200	1.52860200
C	0.99479300	-1.92923000	3.26662300
H	-0.09172400	-1.72336100	1.40908800
H	1.29848000	-2.81060300	1.33384600
C	0.55310200	-0.61841600	3.92285500
H	1.01186000	1.50613400	3.80842900
H	2.41574000	0.46379400	3.72921700
H	0.36289500	-2.75756200	3.59535600
H	2.01458100	-2.17555900	3.58514100
H	0.65259500	-0.68987500	5.00850000
H	-0.51123500	-0.44714200	3.71854400
C	3.19956900	0.49184300	-0.63526600
C	4.41690500	0.40045600	0.29608400
C	3.66258700	0.44894000	-2.10353200
H	2.69219900	1.44152400	-0.45623100
C	5.40499300	1.55011300	-0.00463700
H	4.92886400	-0.55787000	0.15688000
H	4.13540300	0.47660400	1.34515200
C	4.61677300	1.62809600	-2.38951400
H	4.20488200	-0.47996000	-2.30023400
H	2.83200900	0.49902200	-2.81124700
C	5.83816900	1.58659400	-1.47022900
H	6.26876800	1.43649700	0.65425400
H	4.93456200	2.50342100	0.26535500
H	4.91670100	1.58167800	-3.43889300
H	4.08073600	2.57598700	-2.25990700
H	6.47678600	2.45414600	-1.65133800

H	6.44256200	0.70192600	-1.70220700
C	-2.36794600	-1.41496800	-0.37786400
C	-1.92911200	-2.42587100	-1.29619100
C	-2.76868500	-3.52161400	-1.62810200
C	-4.00276100	-3.57999300	-1.03654100
C	-4.43340900	-2.57969500	-0.11239200
C	-3.64793800	-1.50535500	0.22593300
C	-0.65450000	-2.02185900	-1.67344400
H	-2.44036700	-4.28581200	-2.32182100
H	-4.67079500	-4.40236100	-1.26059600
H	-5.41167800	-2.68556500	0.34192300
H	-3.98353300	-0.77409300	0.95151800
H	0.04829900	-2.45581500	-2.36648900
N	-1.42698800	-0.45467700	-0.21701600
N	-0.38391700	-0.86992500	-1.00439400
H	-2.32915900	1.19652200	-0.11715800
C	-3.04973800	2.81434900	0.95725200
C	-3.76471000	2.19467100	-1.20814000
C	-3.92923500	3.88049500	0.97173200
H	-2.39204900	2.57304700	1.78141300
C	-4.65956200	3.24789200	-1.24274800
H	-3.64643100	1.48359800	-2.01474500
C	-4.74301100	4.10090600	-0.14116700
H	-3.97724400	4.52364900	1.84053900
H	-5.28190200	3.39277500	-2.11593900
H	-5.44028900	4.93048000	-0.14815100
N	-2.98743400	2.00768900	-0.12192000

L3d

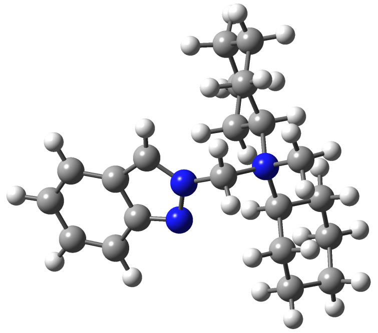

C	0.17911800	0.12438400	1.85237600
H	0.41998900	-0.72706000	2.48290900
H	0.05504000	1.00474700	2.47600400
N	1.42226600	0.37154000	0.98168900
C	2.53251100	0.62983200	1.97009900
H	2.26926300	1.46770900	2.60892900
H	2.68128900	-0.25431500	2.58355100
H	3.44521900	0.85964100	1.42888400
C	1.27897100	1.64897400	0.06816800
C	0.43775900	1.44709100	-1.20206700
C	0.79736000	2.89211200	0.83475100
H	2.31226400	1.82981000	-0.23152700
C	0.56182900	2.69460800	-2.09971000
H	-0.61114700	1.28931000	-0.94392000
H	0.76522200	0.57682800	-1.76947900
C	0.92937300	4.13855100	-0.06334600
H	-0.25360500	2.78227800	1.11461900
H	1.36908900	3.05876900	1.75001200
C	0.14809000	3.97586300	-1.37072700
H	-0.05177000	2.54402900	-2.99123800
H	1.59744800	2.78838700	-2.44955300
H	0.57727800	5.01090400	0.49279400
H	1.98896800	4.31656900	-0.28234300
H	0.30808500	4.84473000	-2.01405600
H	-0.92620700	3.94281200	-1.15294600
C	1.78250300	-0.90719800	0.11877100

C	3.03404900	-0.69376400	-0.74661800
C	1.92571500	-2.18827700	0.95479100
H	0.90452400	-1.03060900	-0.50960700
C	3.22932500	-1.89972800	-1.68756200
H	3.92378500	-0.59930600	-0.11498400
H	2.96578800	0.21188200	-1.35029700
C	2.11812900	-3.39350600	0.01142200
H	2.79096800	-2.12821900	1.62300900
H	1.03483000	-2.37503800	1.55387800
C	3.32686600	-3.21508800	-0.91052300
H	4.12909500	-1.73477900	-2.28557600
H	2.39083300	-1.94726800	-2.39251200
H	2.22506300	-4.29591300	0.61841700
H	1.20826500	-3.52450400	-0.58473100
H	3.39970200	-4.05708700	-1.60364900
H	4.24842100	-3.22115700	-0.31551400
C	-2.50301400	-1.22561900	0.06501500
C	-3.15564100	-0.02475200	0.51405200
C	-4.52097700	0.21195100	0.19948300
C	-5.17835600	-0.73475900	-0.54002500
C	-4.52531600	-1.92733300	-0.98589200
C	-3.21093300	-2.18831600	-0.70074000
C	-2.17613900	0.65692500	1.22324400
H	-5.02414800	1.11017300	0.53666500
H	-6.22064900	-0.58806700	-0.79667600
H	-5.09780800	-2.64322500	-1.56411500
H	-2.72197600	-3.09520700	-1.03393000
H	-2.20096400	1.58528000	1.77052200
N	-1.22467400	-1.27535100	0.47561400
N	-1.05037500	-0.11530500	1.15936800

L3e

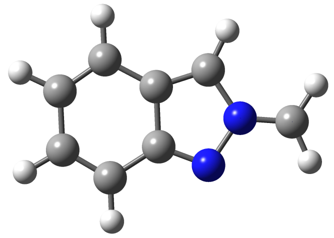

C	-0.07834800	-0.70116900	0.00003100
C	-0.14096500	0.78308700	-0.00004500
C	-1.41109300	1.45013500	-0.00007300
C	-2.52185000	0.67289000	-0.00002800
C	-2.45324100	-0.78300800	0.00004800
C	-1.27986300	-1.47009600	0.00007700
C	1.14576200	1.19701900	-0.00007100
H	-1.46696900	2.53093200	-0.00012700
H	-3.50289900	1.13204300	-0.00004900
H	-3.38938500	-1.32923200	0.00007800
H	-1.23459700	-2.55105400	0.00013000
H	1.61891500	2.16585900	-0.00012300
N	1.15320400	-1.15849800	0.00004400
N	1.93481200	0.00457300	-0.00001100
C	3.22007800	-0.08475000	-0.00000200
H	3.81641800	0.81981900	0.00007100
H	3.65952400	-1.07553900	0.00016500

L3f

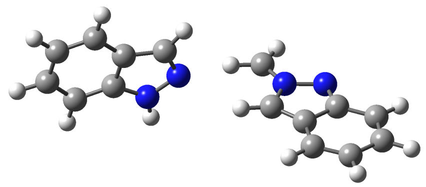

C	-3.67690900	0.14048200	-0.66715300
C	-3.50578600	-0.14263500	0.70900700
C	-4.56695400	-0.70517600	1.44536100
C	-5.75080900	-0.96295100	0.78522100
C	-5.90174100	-0.67252200	-0.59255400
C	-4.87918300	-0.12069000	-1.34096000
C	-2.17268100	0.26335600	0.99259600
H	-4.45598200	-0.92777700	2.49990700
H	-6.58534100	-1.39444000	1.32418400
H	-6.84880800	-0.88969100	-1.07230600
H	-5.00547900	0.09877000	-2.39416900
H	-1.64423800	0.22328600	1.93440700
N	-2.48866100	0.66742800	-1.09327500
N	-1.57375700	0.74554600	-0.08736400
C	1.12582200	2.30400900	0.17093200
H	0.07936100	1.99630000	0.05535100
H	1.46422400	3.31902900	0.34714600
C	3.98113000	0.52934900	0.10428400
C	3.02608800	-0.57937600	-0.12746600
C	3.50727200	-1.92125000	-0.29393400
C	4.84749100	-2.11724100	-0.22953900
C	5.77970900	-1.02376100	-0.00131100
C	5.38266200	0.26663700	0.16338700
C	1.80087800	-0.00684400	-0.12802500
H	2.81646300	-2.73701500	-0.46351900
H	5.25502000	-3.11361500	-0.34941400
H	6.83603800	-1.26354600	0.03701100
H	6.08104300	1.07536700	0.33308100
H	0.78786400	-0.35973300	-0.24986600
N	3.38760700	1.69513000	0.23710500
N	2.03051700	1.38651800	0.09665200
H	-2.25205100	1.00238100	-2.01272200

$TS_{L3f\text{-}g}$

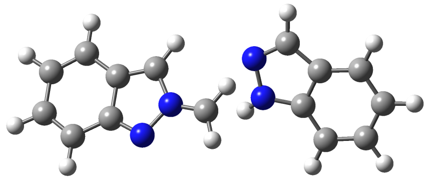

C	3.07136200	-0.26711400	-0.56130800
C	3.61897400	0.83079800	0.13627200
C	4.97738100	0.83042800	0.49042200
C	5.74387600	-0.26287200	0.12409200
C	5.18139500	-1.34674500	-0.58410800
C	3.84091300	-1.37374900	-0.93618600
C	2.52496400	1.74459700	0.28248300
H	5.41499700	1.66378000	1.02679600
H	6.79633800	-0.29085300	0.37822200
H	5.81662900	-2.18004200	-0.86003900
H	3.41876800	-2.21064100	-1.47947400
H	2.52227400	2.71693900	0.75483200
N	1.72669500	0.01225800	-0.72299700
N	1.42009200	1.27668000	-0.25603400
C	-0.24545000	-0.65619400	1.39687100
H	0.45832100	0.15704500	1.50588300
H	-0.04002800	-1.67578500	1.69918800
C	-3.41733900	-0.80201100	0.28736100
C	-3.17273500	0.63707200	0.05087500
C	-4.20590500	1.46106300	-0.50593000

C	-5.39188200	0.86805700	-0.79418100
C	-5.62712300	-0.54661800	-0.55639700
C	-4.68437000	-1.37575400	-0.03220300
C	-1.89810300	0.85776000	0.45352500
H	-4.03502700	2.51515000	-0.68258400
H	-6.20079500	1.45303300	-1.21453000
H	-6.60206100	-0.94485900	-0.81254700
H	-4.86546400	-2.42785800	0.14361700
H	-1.23630300	1.70906700	0.44258800
N	-2.37066200	-1.41997700	0.79131000
N	-1.41835000	-0.40163600	0.91058400
H	1.15218700	-0.34856300	-1.47150700

L3g

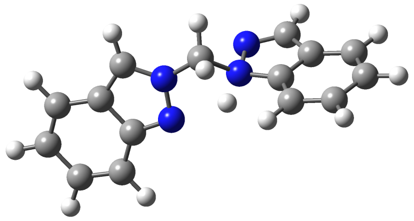

C	2.48515700	-0.20700600	0.11394200
C	3.35316400	0.57747700	-0.64309400
C	4.71018100	0.26637500	-0.69820100
C	5.14564800	-0.84325000	0.02508800
C	4.25244900	-1.61944700	0.77258400
C	2.88675900	-1.31608300	0.83042500
C	2.52525300	1.61364600	-1.23993200
H	5.40187100	0.86074800	-1.28217100
H	6.19388900	-1.11470300	0.00698100
H	4.62250000	-2.47923100	1.31759800
H	2.20058500	-1.93275600	1.39758700
H	2.85361100	2.41751100	-1.88887400
N	1.14518100	0.35537200	-0.06499900
N	1.27522600	1.54135000	-0.93759400
C	0.34183400	0.69497300	1.17329700
H	0.53863600	1.72341700	1.46686500
H	0.64368500	-0.00656800	1.95235800
C	-2.59230400	-0.54934900	-0.22895200
C	-3.21122600	0.49047100	0.54485200
C	-4.61860500	0.66243000	0.52014700
C	-5.35144900	-0.19202700	-0.26242500
C	-4.72941200	-1.22218500	-1.03057300
C	-3.37055000	-1.41623100	-1.03090400
C	-2.15374500	1.14644500	1.17708500
H	-5.09525100	1.44219500	1.10147200
H	-6.42912100	-0.09140600	-0.30492200
H	-5.35803500	-1.86932700	-1.63101100
H	-2.90485100	-2.20013900	-1.61459900
H	-2.12355200	1.98909100	1.84966600
N	-1.25228200	-0.53474600	-0.06220800
N	-1.03159300	0.50220500	0.78459600
H	0.46137200	-0.30552400	-0.54956600

5 References (Supporting Information)

(1) Almarzoqi, B.; George, A. V; Isaacs, N. S. The Quarternisation of Tertiary Amines with Dihalomethane. *Tetrahedron* **1986**, *42* (2), 601–607. https://doi.org/10.1016/S0040-4020(01)87459-7.

(2) Fu, W.-Z.; Wang, W.-J.; Niu, Y.-Y.; Ng, S. W. 1,1^-Methylenedipyridinium Dichloride Monohydrate. *Acta Crystallographica Section E* **2010**, *66* (5), o1211. https://doi.org/10.1107/S1600536810015096.

(3) Penn, K. R.; Anders, E. J.; Lindsay, V. N. G. Expedient Synthesis of Bis(Imidazolium) Dichloride Salts and Bis(NHC) Complexes from Imidazoles Using DMSO as a Key Polar Additive. *Organometallics* **2021**, *40* (23), 3871–3875. https://doi.org/10.1021/acs.organomet.1c00592.

(4) Rudine, A. B.; Walter, M. G.; Wamser, C. C. Reaction of Dichloromethane with Pyridine Derivatives under Ambient Conditions. *J Org Chem* **2010**, *75* (12), 4292–4295. https://doi.org/10.1021/jo100276m.

(5) Musilek, K.; Komloova, M.; Zavadova, V.; Holas, O.; Hrabinova, M.; Pohanka, M.; Dohnal, V.; Nachon, F.; Dolezal, M.; Kuca, K.; Jung, Y.-S. Preparation and in Vitro Screening of Symmetrical Bispyridinium Cholinesterase Inhibitors Bearing Different Connecting Linkage—Initial Study for Myasthenia Gravis Implications. *Bioorg Med Chem Lett* **2010**, *20* (5), 1763–1766. https://doi.org/10.1016/j.bmcl.2010.01.034.

(6) Zhang, Z.-F. Crystal Structure of 1,1'-Methylenedipyridinium Dibromide Monohydrate, (C11H12N2)Br2) H2O. **2011**, *226* (4), 553–554. https://doi.org/doi:10.1524/ncrs.2011.0246.

(7) Brüdgam, I.; Hartl, H. Dipyridiniomethan-Diiodid. *Acta Crystallographica Section C* **1986**, *42* (7), 866–868. https://doi.org/10.1107/S0108270186094258.

(8) Tcyrulnikov, N. A.; Varadharajan, R.; Tikhomirova, A. A.; Pattabiraman, M.; Ramamurthy, V.; Wilson, R. M. Modulation of Reduction Potentials of Bis(Pyridinium)Alkane Dications through Encapsulation within Cucurbit[7]Uril. *J Org Chem* **2019**, *84* (13), 8759–8765. https://doi.org/10.1021/acs.joc.9b01049.

(9) Sarie, J. C.; Thiehoff, C.; Mudd, R. J.; Daniliuc, C. G.; Kehr, G.; Gilmour, R. Deconstructing the Catalytic, Vicinal Difluorination of Alkenes: HF-Free Synthesis and Structural Study of p-TolIF2. *J Org Chem* **2017**, *82* (22), 11792–11798. https://doi.org/10.1021/acs.joc.7b01671.

(10) Laali, K. K.; Jamalian, A.; Zhao, C. Reaction of Selectfluor (F-TEDA-BF4) with Chloromethylated-DABCO Monocation Salts (X=BF4, NTf2) and Other Nitrogen Bases (Et3N; Piperidine; Basic Ionic Liquid); Unexpected Formation of Symmetrical [N−H−N]+ Trication Salts. *Tetrahedron Lett* **2014**, *55* (49), 6643–6646. https://doi.org/10.1016/j.tetlet.2014.10.071.

(11) Finke, A. D.; Gray, D. L.; Moore, J. S. 1-Bromomethyl-4-Aza-1-Azoniabicyclo-[2.2.2]Octane Bromide. *Acta Crystallogr Sect E Struct Rep Online* **2010**, *66* (2), o377–o377. https://doi.org/10.1107/S1600536810000292/PK2223ISUP2.HKL.

(12) Kvasovs, N.; Fang, J.; Kliuev, F.; Gevorgyan, V. Merging of Light/Dark Palladium Catalytic Cycles Enables Multicomponent Tandem Alkyl Heck/Tsuji–Trost Homologative Amination Reaction toward Allylic Amines. *J Am Chem Soc* **2023**, *145* (33), 18497–18505. https://doi.org/10.1021/jacs.3c04968.

(13) Gustafsson, B.; Håkansson, M.; Jagner, S. Copper(I)-Mediated Quaternisation of 1,4-Diazabicyclo[2.2.2]Octane (DABCO). Crystal Structure of Bis{(1-Chloromethyl-4-Aza-1-Azoniabicyclo[2.2.2]Octane)-μ-Chloro-Chlorocopper(I)}. *Inorganica Chim Acta* **2005**, *358* (4), 1309–1312. https://doi.org/10.1016/j.ica.2004.10.026.

(14) Hassner, A.; Krepski, L. R.; Alexanian, V. Aminopyridines as Acylation Catalysts for Tertiary Alcohols. *Tetrahedron* **1978**, *34* (14), 2069–2076. https://doi.org/10.1016/0040-4020(78)89005-X.

(15) DIssanayake, D. M. M. M.; Vannucci, A. K. Selective N1-Acylation of Indazoles with Acid Anhydrides Using an Electrochemical Approach. *Org Lett* **2019**, *21* (2), 457–460. https://doi.org/10.1021/acs.orglett.8b03683.

(16) Kim, M.; Na, H.-N.; Shen, L.; Jeong, J. H. Direct Introduction of an Acetyl Group at the α-Carbon Atom of an Arene Ring through an Amide Photo-Fries Rearrangement upon Exposure to UV Light. *European J Org Chem* **2024**, *27* (26), e202400281. https://doi.org/10.1002/ejoc.202400281.

(17) Karl von Auwers. Über Cumaranon Und Hydrindon. *Chem Ber* **1919**, *52*, 1332–1337.

(18) Bruker (2012). APEX5. Bruker AXS Inc., Madison, Wisconsin, USA.

(19) Sheldrick, G. M. SHELXT – Integrated Space-Group and Crystal-Structure Determination. *Acta Crystallographica Section A* **2015**, *71* (1), 3–8. https://doi.org/10.1107/S2053273314026370.

(20) Sheldrick, G. M. Crystal Structure Refinement with SHELXL. *Acta Crystallographica Section C* **2015**, *71* (1), 3–8. https://doi.org/10.1107/S2053229614024218.

(21) Dolomanov, O. V; Bourhis, L. J.; Gildea, R. J.; Howard, J. A. K.; Puschmann, H. OLEX2: A Complete Structure Solution, Refinement and Analysis Program. *J Appl Crystallogr* **2009**, *42* (2), 339–341. https://doi.org/10.1107/S0021889808042726.

(22) Becke, A. D. Density-functional Thermochemistry. III. The Role of Exact Exchange. *J Chem Phys* **1993**, *98* (7), 5648–5652. https://doi.org/10.1063/1.464913.

(23) Lee, C.; Yang, W.; Parr, R. G. Development of the Colle-Salvetti Correlation-Energy Formula into a Functional of the Electron Density. *Phys Rev B* **1988**, *37* (2), 785–789. https://doi.org/10.1103/PhysRevB.37.785.

(24) Lignell, A.; Khriachtchev, L.; Räsänen, M.; Pettersson, M. A Study on Stabilization of HHeF Molecule upon Complexation with Xe Atoms. *Chem Phys Lett* **2004**, *390* (1), 256–260. https://doi.org/10.1016/j.cplett.2004.04.033.

(25) van Slageren, J.; Klein, A.; Záliš, S.; Stufkens, D. J. Resonance Raman Spectra of D6 Metal–Diimine Complexes Reflect Changes in Metal–Ligand Interaction and Character of Electronic Transition. *Coord Chem Rev* **2001**, *219–221*, 937–955. https://doi.org/10.1016/S0010-8545(01)00388-5.

(26) Cossi, M.; Rega, N.; Scalmani, G.; Barone, V. Energies, Structures, and Electronic Properties of Molecules in Solution with the C-PCM Solvation Model. *J Comput Chem* **2003**, *24* (6), 669–681. https://doi.org/https://doi.org/10.1002/jcc.10189.

(27) Herbert, M.; Montilla, F.; Galindo, A. Olefin Epoxidation in Solventless Conditions and Apolar Media Catalysed by Specialised Oxodiperoxomolybdenum Complexes. *J Mol Catal A Chem* **2011**, *338* (1), 111–120. https://doi.org/10.1016/j.molcata.2011.02.004.

(28) Herbert, M.; Montilla, F.; Álvarez, E.; Galindo, A. New Insights into the Mechanism of Oxodiperoxomolybdenum Catalysed Olefin Epoxidation and the Crystal Structures of Several Oxo–Peroxo Molybdenum Complexes. *Dalton Transactions* **2012**, *41* (23), 6942–6956. https://doi.org/10.1039/C2DT12284G.

(29) Costa, P. J.; Calhorda, M. J.; Kühn, F. E. Olefin Epoxidation Catalyzed by H5-Cyclopentadienyl Molybdenum Compounds: A Computational Study. *Organometallics* **2010**, *29* (2), 303–311. https://doi.org/10.1021/om9002522.

(30) M. J. Frisch, G. W. Trucks, H. B. Schlegel, G. E. Scuseria, M. A. Robb, J. R. Cheeseman, G. Scalmani, V. Barone, B. Mennucci, G. A. Petersson, H. Nakatsuji, M. Caricato, X. Li, H. P. Hratchian, A. F. Izmaylov, J. Bloino, G. Zheng, J. L. Sonnenberg, M. Hada, M. Ehara, K. Toyota, R. Fukuda, J. Hasegawa, M. Ishida, T. Nakajima, Y. Honda, O. Kitao, H. Nakai, T. Vreven, J. A. Montgomery, Jr., J. E. Peralta, F. Ogliaro, M. Bearpark, J. J. Heyd, E. Brothers, K. N. Kudin, V. N. Staroverov, T. Keith, R. Kobayashi, J. Normand, K. Raghavachari, A. Rendell, J. C. Burant, S. S. Iyengar, J. Tomasi, M. Cossi, N. Rega, J. M. Millam, M. Klene, J. E. Knox, J. B. Cross, V. Bakken, C. Adamo, J. Jaramillo, R. Gomperts, R. E. Stratmann, O. Yazyev, A. J. Austin, R. Cammi, C. Pomelli, J. W. Ochterski, R. L. Martin, K. Morokuma, V. G. Zakrzewski, G. A. Voth, P. Salvador, J. J. Dannenberg, S. Dapprich, A. D. Daniels, O. Farkas, J. B. Foresman, J. V. Ortiz, J. Cioslowski, and D. J. Fox, Gaussian 09, Revision B.01. Gaussian, Inc., Wallingford CT **2016**.
